# A systematic fluorescently tagged protein toolkit for dissecting apical extracellular matrix patterning in *C. elegans*

**DOI:** 10.1371/journal.pbio.3004059

**Published:** 2026-10-14

**Authors:** James Matthew Ragle, Murugesan Pooranachithra, Guinevere E. Ashley, Emma Cadena, Batia Blank, Katelyn Kang, Cincy Chen, Aditree R. Bhowmick, Soraya H. Mercado, Tabatha E. Wells, John C. Clancy, Andrew D. Chisholm, Jordan D. Ward

**Affiliations:** 1 Department of Molecular, Cell and Developmental Biology, University of California Santa Cruz, Santa Cruz, California, United States of America; 2 Department of Cell and Developmental Biology, School of Biological Sciences, University of California San Diego, La Jolla, California, United States of America; University of Notre Dame, Center for Stem Cells and Regenerative Medicine, UNITED STATES OF AMERICA

## Abstract

Barrier epithelia are shielded from the external environment by their apical extracellular matrices (aECMs). The molecular complexity of aECMs has challenged understanding of their organization in vivo. To define the molecular architecture of a model aECM we generated a toolkit of 105 fluorescently tagged aECM components using gene editing in *C. elegans*, focusing on proteins secreted by the epidermis to form the collagen-rich cuticle. We developed efficient pipelines for modular protein tagging and rapid fluorophore swapping. Most tagged collagens were functional and exhibited exquisitely specific patterning across stages, cell types, and matrix substructures. We define multiple reference markers for key substructures including the little-understood cortical layer, as well as the helical crossed fiber arrays that function as a hydrostatic skeleton to maintain organismal shape. We further tagged 30 members of key aECM protein classes including proteases, protease inhibitors, and lipid transporters. Our standardized markers will allow dissection of the mechanistic basis of aECM spatiotemporal patterning in vivo.

## Abstract 2

Barrier epithelia are protected by apical extracellular matrices (aECMs), but their complex organization remains poorly understood in vivo. This study generates a toolkit of 105 fluorescently tagged aECM components and defines reference markers for key substructures, enabling analysis of spatiotemporal extracellular matrix patterning.

## Introduction

Extracellular matrix (ECM) comprises a wide variety of compartments including those formed by epithelial tissues such as basement membranes (BMs) and apical extracellular matrices (aECMs). ECMs play myriad roles in organismal development [1], and are key determinants of disease pathology [2] and aging [3]. The molecular complexity and diversity of such ECMs is increasingly appreciated. Estimates of the total number of protein species of the ECM, i.e., the matrisome [4,5], range from the hundreds to over 1,000, many of which are subject to complex processing and post-translational modification. In short, the ECM is one of the most complex aspects of metazoan development and physiology.

One major type of ECM is the basement membrane of epithelia, formed around a conserved set of proteins such as laminins and type IV collagens [6]. Recent studies in *C. elegans* and mammals have systematically tagged 46 BM components including tissue- or stage-specific proteins [7,8]. These large-scale resources have revealed the molecular diversity of BMs in vivo. While previous work had suggested that BMs turned over on a scale of weeks, analysis of endogenously tagged proteins revealed BMs are far more dynamic than appreciated [7]. Such resources have also established the importance of studying ECM components using endogenous knock-in tags, consistent fluorescent protein tags and a modular editing pipeline for stable and reproducible analysis of protein localization.

Here we focus on aECMs, a highly heterogeneous set of complex ECMs [9]. The nematode *C. elegans* generates several distinct aECMs [10,11], of which the largest is the epidermal aECM or cuticle, a critical determinant of organismal morphogenesis [12], a functional permeability barrier [13], skin wound healing [14], mechanosensation [15], host-pathogen interactions [16,17], mate recognition [18], and aging [3]. The main body cuticle is continuous with more specialized interfacial aECMs such as those generated by glial cells [19] or epidermal orifices such as the vulva [20]. Throughout development, the cuticle can be broadly divided into collagen-rich basal layers and cortical layers containing collagens and additional insoluble cuticlins and epicuticlins [10]. The surface of the cortical layer further forms a lipid-rich epicuticle that contributes to the permeability barrier [21] and an outermost glycoprotein-rich surface coat that affects interaction with pathogens [22]. The body cuticle also contains multiple stage- and region-specific specializations, such as longitudinal ridges (alae) formed in L1, dauer, and adult [23] and circumferential ridges (annuli) separated by furrows (Fig 1A). Each of the four larval stages displays a body cuticle that is initially formed in the preceding stage (e.g., L1 cuticle is assembled in the embryo, L2 cuticle is assembled in L1, and so on). Compared to these transient cuticles formed in development, the mature adult cuticle contains several additional compartments, such as a fluid-filled medial layer between the cortical and fibrous layers that contains organized arrays of cylindrical struts [24].

**Fig 1:**
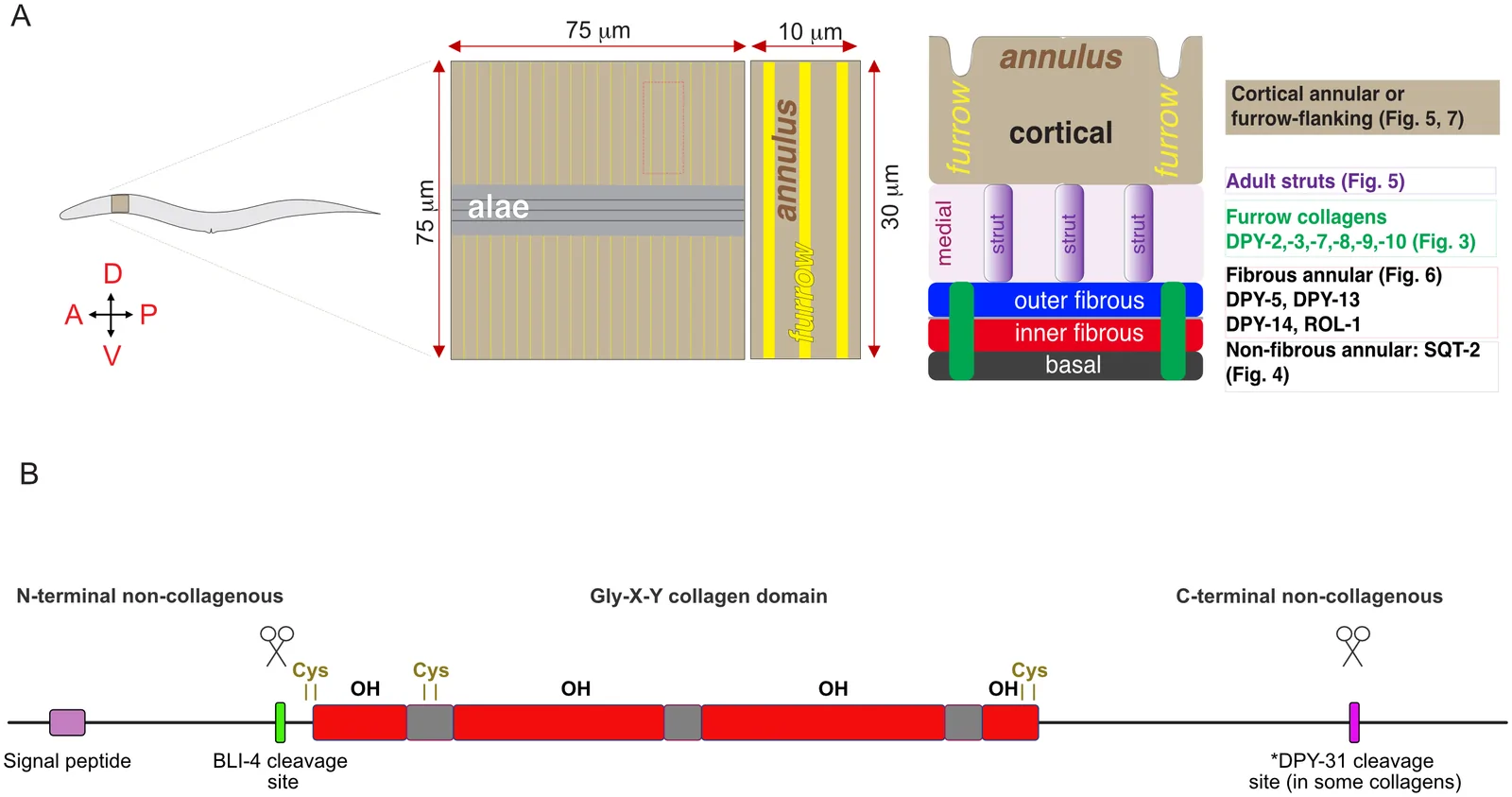
Collagen processing and cuticle structure. **(A)** Standard images of cuticle aECM in this and subsequent figures show 75 x 75 µm panels centered on the lateral alae in the anterior or mid body. Insets show magnified views of the annular and furrow regions. Right, cuticle cross section, longitudinal aspect showing one annulus, two furrows, and three struts, indicating furrow and fibrous/basal collagen localization in adult cuticle. Our analyses suggest furrow collagens localize to the basal layer in adults [24]. **(B)** Generic *C. elegans* collagen structure, based on [30]. An N-terminal propeptide (containing a predicted signal peptide or transmembrane domain) is cleaved by the BLI-4 subtilisin-like protease. A central collagen domain contains Gly-X–Y repeats (red boxes) with a variable number of interruptions (gray boxes). Conserved cysteine-rich domains (Cys) flank the collagen domain and are found between one of the Gly-X-Y sections of the collagen domain. Prolines and lysines in the collagen domain are hydroxylated (OH). Some but not all *C. elegans* collagens are processed in the C-terminal noncollagenous domain by the DPY-31 protease.

The *C. elegans* epidermal aECM is remarkably complex at a molecular level, with at least 175 distinct types of cuticle collagens and a similar number of noncollagen proteins [25] such as cuticlins, epicuticlins, C-type lectins, chondroitin proteoglycans, mucins, and zona pellucida (ZP) domain proteins. The aECM collagens have been classified by sequence and domain architecture [25]; their Gly-X-Y repeats have characteristic sequence interruptions similar to vertebrate FACIT or MACIT collagens [10,11] (Fig 1B). Specific collagen localization correlates with known morphological sub-structures such as annuli [26] (Fig 1A). Transcriptomic studies have revealed that ~20% of *C. elegans* genes oscillate in expression, peaking at a stereotypical point in each larval stage [27–29]. Such gene sets are enriched in aECM components whose oscillation reflects the periodic assembly of the cuticle in the four larval molts. Mutation or inactivation of specific oscillating collagens affects the structural organization of collagens expressed at a similar time, but not collagens from other temporal groups [26]. Moreover, the epidermal aECM of each stage is prefigured by a transient aECM known as precuticle [10]. Super-resolution microscopy has revealed nanoscale organization of collagens within the adult-specific strut compartment [24] and has revealed epicuticlins as novel components of the cortical layer [14]. These studies have reinforced the importance of standardized tagging to build a molecular atlas of aECM components.

Here we report a protein-tagging resource of knock-in strains for 105 aECM components as the basis for such a molecular atlas. We selected genes likely to be informative aECM markers based on several criteria including genetic function, expression pattern, or sequence orthology. We optimized a gene editing pipeline to make endogenously mNeonGreen (mNG) tagged versions of 62 cuticle collagens and 30 noncollagen aECM proteins. For select genes, we generated 13 additional mScarlet (mSc) red FP-tagged versions to facilitate co-localization studies. We tagged 40 proteins previously shown to be cuticle components and validated previously observed patterns. Of the set of collagens identified based on phenotype, most tagged knock-ins retained normal function. While many tagged proteins localized to previously described cuticle substructures, others defined previously unknown substructures or revealed previously unappreciated aspects of known structures. This aECM toolkit provides a resource for further studies of aECM architecture and the mechanistic basis of complex ECM structure and function.

## Results

### A modular pipeline for protein-tagging knock-ins

We first optimized parameters for Cas9 ribonucleoprotein complex (RNP) and linear repair template-based genome editing using a GFP knock-in into the 5′ end of the *glh-1* gene. We chose *glh-1* as a test case due to its use in optimization of editing efficiency and ease of scoring [31,32]. We tested Cas9 source and amount, RNA:Cas9 ratio, repair template, and methods to enrich for edits. IDT Cas9 at 250 ng/µl was most efficient, similar to a previous report (S1A Fig) [31]. While RNA:Cas9 ratios from 1:1–10:1 have been used [33–35], we found that a 1:1 ratio was most efficient with our reagents (S1B Fig). We did not find significant improvements from 5′ modifications [36] (S1C Fig), however, ssDNA templates [37] slightly increased efficiency (S1D Fig). We next tested methods to enrich for edits. In pilot experiments generating collagen knock-ins we were unsuccessful with pRF4 (*rol-6(su1006)*) or *dpy-10* co-conversion [31,38], possibly due to genetic interactions between dominant collagen mutations and the target genes.

We next tested whether an *mlc-1p::NLS::tagRFP* co-injection marker could enrich for successful edits. Among marker-positive animals, 26% contained a *GFP::GLH-1* knock-in, compared with 2.4% among marker-negative animals (S1E Fig). Comparable editing efficiencies were obtained when screening F1 progeny laid either 1 or 2 days post-injection (S1F Fig), although more total knock-ins were recovered from day 1 progeny (S1G Fig). We also tested whether *myo-2::T2A::GFP* could be used as a co-CRISPR marker. However, significantly fewer knock-ins were recovered among *myo-2::T2A::GFP*-positive progeny (S1H Fig). Based on these results, we used the *mlc-1p::NLS::tagRFP* co-injection marker for subsequent experiments, together with unmodified dsDNA repair templates and our optimized Cas9 concentration and RNA ratio. We prioritized speed in our goal of generating 100+ aECM knock-in strains and did not extensively optimize failed edits. We detail these failed edits in S1 Table and plan to revisit them to improve our editing pipeline.

We developed a modular tagging strategy in which an initial insertion allows rapid validation of phenotype, expression, and localization. We designed an mNG::3xFLAG cassette flanked by flexible 13 amino acid linkers that contain four crRNA target sites oriented for optimal editing efficiency (crRNAs 1–4; Fig 2A) [39]. Our initial insertion includes an mNG::3xFLAG flanked by the flexible linker sequences. Re-editing of the knock-in using the internal sgRNA sites (crRNAs #2 + 3) allows for removal of the mNG::3xFLAG and subsequent insertion of a desired cassette using a single, common repair template and two crRNAs (Fig 2A). This approach ensures that our toolkit strains can be modified by any researcher in an efficient, cost-effective manner. Use of different internal and external crRNA pairs allows for removal or retention of the linker sequences during re-editing. We first tested this system by attempting to replace the mNG::3xFLAG tag in *glh-1* with an mSc::2xOLLAS tag using unmodified or biotinylated dsDNA or ssDNA repair templates. While biotinylated dsDNA and ssDNA repair templates did not boost *glh-1* knock-in efficiency (S1C and S1D Fig), they dramatically improved color swap efficiency (Fig 2B), suggesting variation between targets that warrants further exploration. We also validated that the other crRNA pairs (crRNAs 1 + 4 and 2 + 4) allowed efficient tag swapping, except for crRNAs #1 + 3 (Fig 2C). These data may suggest that crRNA 3 is a suboptimal guide and edits using it might benefit from biotinylated dsDNA or ssDNA repair templates. We proceeded to generate knock-ins in aECM genes using either mNG or modular mNG::3xFLAG knock-ins, as mNG has been widely used to tag *C. elegans* BM proteins [7], and is frequently brighter than GFP [40,41]. In most cases, we initially inserted a tag at the C-terminus unless there was evidence for C-terminal processing (e.g., SQT-3) [42]; as discussed further below, C-terminal tagging appeared to be broadly tolerated.

**Fig 2:**
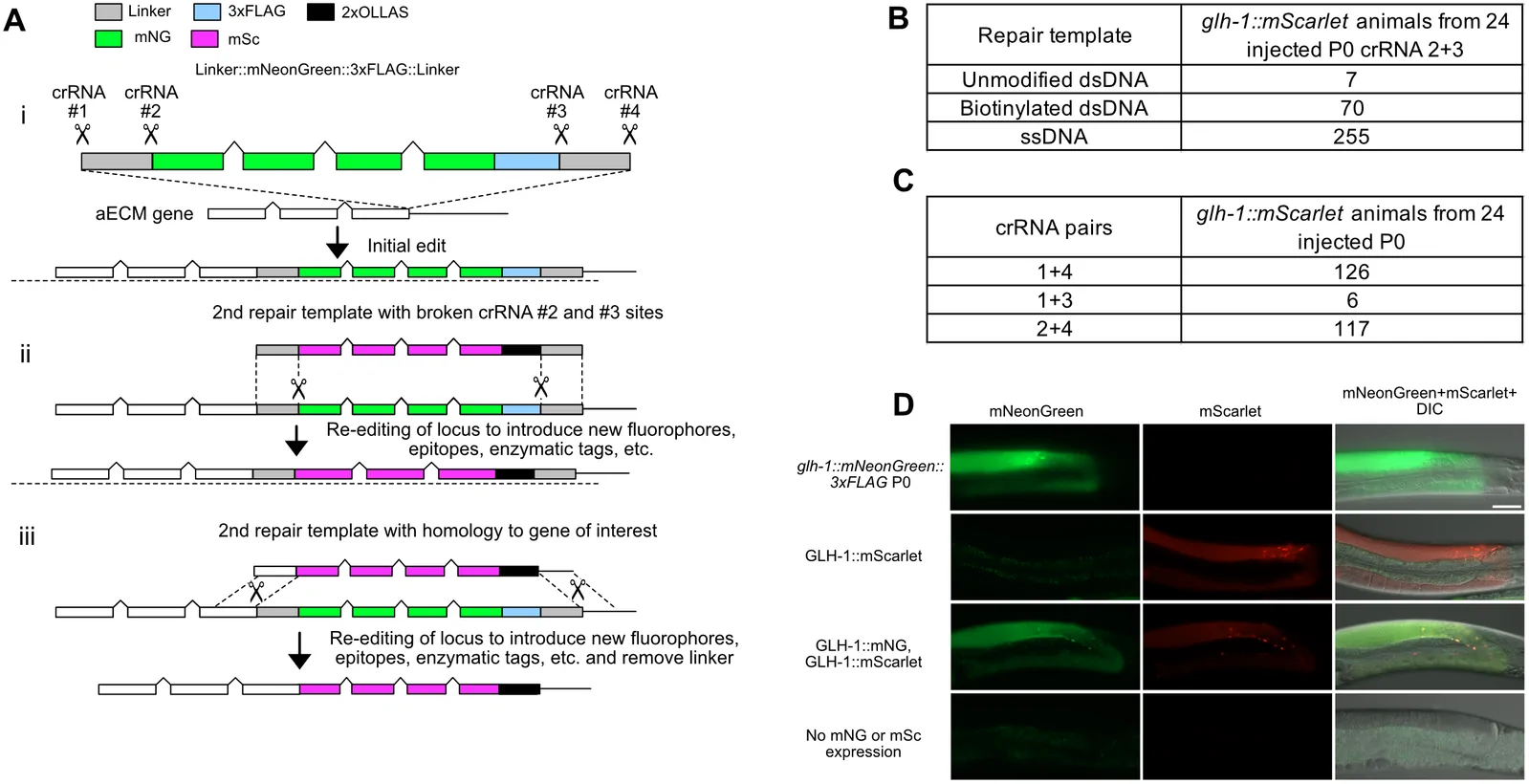
Development of a modular knock-in cassette. **(A)** (i) Initial knock-ins were made using an mNeonGreen::3xFLAG cassette flanked by linkers containing unique, high-efficiency crRNA sites. (ii) Re-editing the initial knock-in with the internal crRNA sites (#2 and #3) allows replacement of the mNeonGreen::3xFLAG with any desired epitope. The linkers act as 35 bp homology arms and thus a single PCR repair template can be used to re-edit any initial knock-in. (iii) Using the external crRNA sites (#1 and #4) allows removal of the linker sequence during re-editing. **(B and C)** A combination of crRNAs allows retention of the N- or C-terminal linkers during re-editing. Number of *glh-1::mSc::2xOLLAS* animals recovered from 24 *glh-1::mNG::3xFLAG* P_0_ animals from two independent experiments injected with the indicated repair templates and crRNAs targeting the internal sites (B) and dsDNA repair templates using the indicated crRNA pairs (C). **(D)** Representative images of parental *glh-1::mNG::3xFLAG* animals, and color-swapped *glh-1::mSc::2xOLLAS* animals with and without GLH-1::mNG::3xFLAG expression. Images are representative of 20 animals imaged in two independent experiments. Scale bars = 50 µm.

We successfully swapped mNG::3xFLAG to mSc::2xOLLAS in 9 strains. In color swap experiments, we also frequently observed mNG-negative animals in the progeny of injected animals, suggesting that the cassette was lost or inactivated (Fig 2D). Further optimization such as inactivation of nonhomologous end-joining [43] may be desirable. We were also able to color-swap three of the nonmodular mNG alleles (*col-63, dpy-10,* and *dpy-13*).

Our first pass knock-in attempts typically used melted PCR products with 35 bp homology arms. For targets that were unsuccessful using this approach, we developed a complementary selection-based strategy. We embedded an *unc-119(+)* selection marker in a synthetic intron in our modular mNG::3xFLAG cassette (S2 and S3 Figs), similar to a previous study [44], generating three strains (S2B Fig). Several *unc-119*-based knock-ins also failed, which are detailed in S1 Table. In the case of *spia-1*, we employed a self-excising cassette [45] due to concerns over the failure of *C. briggsae unc-119(+)* to rescue spore adhesion phenotypes [46]. We also generated a *bli-5::mNG::3xFLAG* knock-in using the SEC method prior to our generation of the *unc-*119-based selection system. Overall, plasmid-based repair templates with long homology arms and a selectable marker provided an effective alternative when simpler repair templates were unsuccessful but required substantially more experimental work to construct.

### Gene families of the aECM and selection for toolkit

We focused on secreted protein families likely to comprise stable components of the aECM [10,47] and which were robustly expressed in public RNAseq datasets. The body cuticle is composed of collagens and noncollagenous proteins. The *C. elegans* genome contains ~180 collagen-encoding genes (see Methods and S1 File). We first focused on those for which phenotypic information existed (‘genetically defined’ collagens; bold face genes in Table 1). We next prioritized collagens based on expression as described above. These included collagens expressed in multiple larval stages (‘oscillating’) as well as stage-specific collagens, especially those upregulated in L4 or adult. These classes overlapped as some collagen genes are both oscillating and display stage specificity. Single-cell transcriptomic datasets such as (www.cengen.org) [48] indicate cuticle collagens are primarily expressed in the epidermis (seam cells or epidermal syncytia) or in interfacial glial cells that generate the overlying aECM.

**Table 1:** Graphical overview of aECM knock-in localization. Genes are sorted by gene or transcription unit name; genes first defined by mutant phenotype are in bold face. Tag: mNeonGreen (mNG) or mScarlet (mSc). Origin: Ward lab (JDW), Chisholm lab (CZ) or SunyBiotech (PHX). DS, visibility under standard dissection microscope conditions. Black box = strong, gray = weak, white = not visible. Notes: overt phenotypes or localization to epidermal puncta consistent with retention in secretory pathway. Expression and localization columns and coding: Stages: Embryo, L1, L2, L3, L4, Adult, Dauer. aECM substructures: Furrows (green) or furrow-flanking (dark green); Annuli cortical (cyan, c) or basal/fibrous (pink); Struts (purple); Alae (gold), Interfacial (sensilla, excretory pore, vulva, rectal, etc); Male (male tail rays, fan, or hook); Other: Gut (g), Pharynx (p), Uterus (u), somatic gonad (s), coelomocyte (c), reproductive system (r), muscle (m). S2 Table is a sortable spreadsheet of this data to facilitate further analysis.

**Gene**	**Tag**	**Origin**	**DS**	**Note**	Em	L1	L2	L3	L4	Ad	Da	Fu	An	St	Al	In	Ma	Ot
*acn-1*	**mNG**	JDW		puncta														
*bli-5*	**mNG**	JDW	no															
C26B2.8	**mNG**	PHX	no															p
*clec-180*	**mNG**	JDW																
*col-10*	**mNG**	JDW											c					
*col-12*	**mNG**	JDW											c					
*col-13*	**mNG**	JDW																
*col-14*	**mNG**	JDW																
*col-17*	**mNG**	PHX																
*col-19*	**mNG**	CZ																
*col-20*	**mNG**	PHX																
*col-38*	**mNG**	PHX																
*col-39*	**mNG**	PHX		puncta														
*col-41*	**mNG**	JDW																
*col-43*	**mNG**	PHX																
*col-46*	**mNG**	PHX	no	puncta														
*col-48*	**mNG**	PHX																
*col-49*	**mNG**	PHX																
*col-51*	**mNG**	PHX																
*col-63*	**mNG mSc**	PHX																
*col-71*	**mNG**	PHX																
*col-75*	**mNG**	JDW																
*col-80*	**mNG**	PHX											c					
*col-81*	**mNG**	PHX																
*col-91*	**mNG**	PHX											c					
*col-92*	**mNG**	PHX																
*col-93*	**mNG**	PHX																
*col-96*	**mNG**	PHX																
*col-97*	**mNG**	JDW																
*col-98*	**mNG**	PHX											c					
*col-103*	**mNG mSc**	JDW																
*col-118*	**mNG**	JDW																
***col-121***	**mNG**	PHX																
*col-124*	**mNG**	PHX																
*col-125*	**mNG mSc**	JDW		Mlt?									c					
*col-128*	**mNG**	PHX	no	puncta														
*col-130*	**mNG**	PHX											c					
*col-135*	**mNG**	PHX	no															u
*col-138*	**mNG**	PHX																
*col-149*	**mNG**	PHX											c					
*col-159*	**mNG**	JDW																
*col-166*	**mNG**	JDW																
*col-169*	**mNG**	PHX											c					
*col-174*	**mNG**	PHX																
*col-178*	**mNG**	PHX																
*cpg-7*	**mNG**	JDW																
*cpz-1*	**mNG mSc**	JDW																g
*crim-1*	**mNG**	JDW																p
*ctsa-1.1*	**mNG mSc**	JDW																cgmr
*cut-2*	**mNG**	JDW																
***dpy-1***	**mNG**	PHX	no	puncta														
***dpy-2***	**mNG**	PHX																
***dpy-3***	**mNG**	PHX																
***dpy-4***	**mNG**	JDW		Dpy														
***dpy-5***	**mNG mSc**	PHX																
***dpy-6***	**mNG**	PHX																
***dpy-8***	**mNG**	PHX		Dpy														
***dpy-9***	**mNG**	PHX																
***dpy-10***	**mNG mSc**	PHX																
***dpy-13***	**mNG mSc**	PHX																
***dpy-14***	**mNG mSc**	JDW																
***dpy-17***	**mNG**	PHX																
F40A3.3	**mNG**	PHX		diffuse														
*gmap-1*	**mNG**	PHX																
*kund-6*	**mNG**	JDW																
*kund-7*	**mNG mSc**	JDW																p
***lon-3***	**mNG**	PHX																
***lon-8***	**mNG mSc**	JDW																
*lrp-1*	**mNG**	JDW																
*mlt-8*	**mNG**	JDW																
*mlt-9*	**mNG**	JDW		puncta														
*mlt-10*	**mNG**	JDW		puncta														
*nas-37*	**mNG mSc**	JDW		head														
*noah-1*	**mNG**	JDW		puncta														
*noah-2*	**mNG**	JDW																
*npa-1*	**mNG**	JDW	no															c
*piit-1*	**mNG mSc**	JDW																p
*qua-1*	**mNG**	JDW		puncta														
***ram-1***	**mNG**	PHX																
***ram-2***	**mNG**	JDW		Ram									c					
***ram-4***	**mNG**	PHX		Ram														
***rol-1***	**mNG**	PHX																
***rol-6***	**mNG**	PHX																
***rol-8***	**mNG**	JDW																
***skpo-1***	**mNG**	JDW																s
***spia-1***	**mNG**	JDW	no															
***sqt-1***	**mNG**	PHX																
***sqt-2***	**mNG**	JDW																
***sqt-3***	**mNG**	PHX																
*srap-1*	**mNG**	JDW																
ZK470.6	**mNG**	PHX	no															p
*wrt-2*	**mNG**	JDW																

While our imaging largely focused on collagens, we also tagged selected members of major noncollagen secreted protein families likely to be components of aECMs. We tagged the ZP domain protein DPY-1, the cysteine cradle domain proteins DPY-6 [49] and SPIA-1 [50], and the novel secreted protein LON-8 implicated in body cuticle morphology [51]. We also tagged the ZP domain proteins NOAH-1 and NOAH-2, components of the pre-cuticle [52]. We tagged the non-ZP cuticlin CUT-2 as a representative of the BME-insoluble cuticle fraction [53]. As aECM remodeling is important in molting, we tagged the extracellular peroxidase SKPO-1, astacin metalloproteases (NAS-8, NAS-37) [54], cathepsins (CPZ-1, CPZ-2, and CTSA-1.1), and putative protease inhibitors (BLI-5, PIIT-1, F30H5.3 (KUND-6), K10D3.4 (KUND-7)). As *C. elegans* has a number of poorly characterized putative protease inhibitors with Kunitz domains, we annotated this group of genes, classifying them as **KUN**itz protease inhibitor **D**omain containing (*kund)* (S3 Table). We tagged CLEC-180 as a representative of C-type lectins [55] and CPG-7 as a representative of chondroitin proteoglycans [56]. We also tagged the mucin SRAP-1, and proteins implicated in molting (ACN-1, QUA-1, MLT-8, MLT-9, MLT-10, and WRT-2) [57]. We also tagged several genes that may regulate extracellular lipids, including secreted lipid binding proteins such GM2AP/GMAP-1 [21], the nematode polyprotein allergen-related NPA-1 [58], and the low-density lipoprotein receptor LRP-1 [59]. GMAP-1::mNG was expressed in epidermis and localized to the cuticle, consistent with prior transgenic studies [21]. Other noncollagen proteins localized to a variety of tissues or compartments (Table 1; see S4 Fig), broadly consistent with their transcriptomic data [48].

### Localization of genetically defined cuticle components

To test whether aECM components tolerate mNG tagging we first examined a set of 27 cuticle genes initially defined by mutant phenotypes [10,51,60,61]. Knock-ins for some have been previously reported (e.g., *bli-1, bli-2, bli-6*, *dpy-13, dpy-17,* and *sqt-3*) [24,62]; we generated knock-ins for the remaining genes. Most knock-in strains for such genetically defined cuticle genes were morphologically normal and displayed bright fluorescence in the cuticle aECM. In our initial survey of localization patterns, we examined lateral body cuticle regions containing circumferential furrows and annuli as well as longitudinal alae (Fig 1A). Six ‘furrow DPY’ collagens localized close to morphologically defined circumferential furrows (Fig 3A) [24,26,63]. Within this set, individual patterns differed in detail, consistent with recent observations [64]. DPY-2::mNG showed the most consistent labeling, extending around the circumference to the lateral midline or lateral alae, without breaks (Fig 3A). DPY-3::mNG furrows often did not extend to the lateral midline whereas DPY-9::mNG extended to the midline but had occasional gaps (Fig 3A). DPY-10::mNG extended to the midline with few gaps but frequent bright dots at the ends (Fig 3A). Animals homozygous for DPY-2::mNG and DPY-9::mNG displayed brighter expression than single knock-in homozygotes, but with frequent gaps or dots (Fig 3B); neither mNG signal extended to the midline, although morphological furrows extended to the midline as visualized by DIC (Fig 3B, lower panel). In contrast, DPY-9::mNG DPY-10::mSc ROL-6::mNG triple knock-ins displayed furrow morphology similar to DPY-9::mNG single knock-ins (Fig 3C). These observations suggest certain knock-in alleles or combinations may mildly affect protein function or localization (see below). Although overlapping in the xy plane, DPY-10::mSc localized more cortically than DPY-9::mNG (Fig 3C, right panels), suggesting furrow collagens may define distinct subcompartments. Furrow collagen mutants display defective aECM permeability barrier function [65]. Interestingly, BIS-1/COL-121, identified on the basis of permeability barrier defects in loss-of-function mutants [61], also localized to embryonic and L1 stage furrows (Fig 4D). Overall, these results in conjunction with other observations [64] suggest ‘furrow’ collagens may define distinct subregions within furrows.

**Fig 3:**
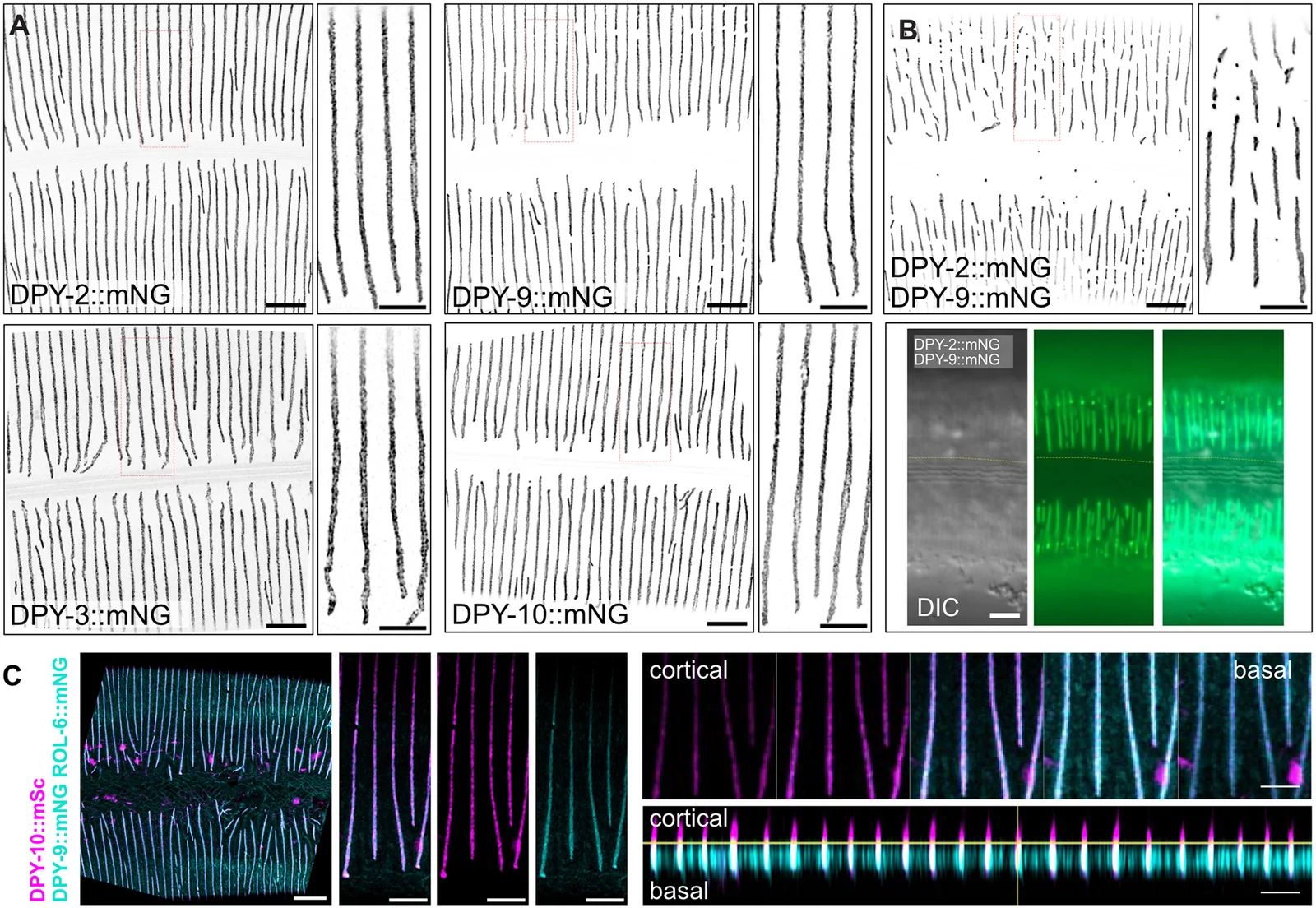
Localization of genetically defined collagens: furrows. Images are confocal z-stacks or single focal planes, oriented with anterior to the left and dorsal up; all images are of adults 24 h post L4 stage unless stated. Standard images of cuticle aECM in this and subsequent Figures show 75 x 75 µm panels centered on the lateral alae in the anterior or mid body. Insets show annular and furrow regions in close-up. Right, cuticle cross section, longitudinal aspect showing 1 annulus, two furrows, and three struts, indicating furrow and fibrous/basal collagen localization in adult cuticle. Our analysis suggests furrow collagens localize to the basal layer in adults [24]. **(A)** Localization of genetically defined furrow collagens DPY-2::mNG, DPY-3::mNG, DPY-9::mNG, and DPY-10::mNG. Airyscan confocal stacks. Large panels are 75 x 75 µm ROIs; enlarged insets are 30 x 10 µm. **(B)** Double label strain containing DPY-2::mNG and DPY-9::mNG showing fragmentation of the furrow mNG patterns. Lower panels showing DIC and widefield fluorescence. Scales, 10 µm (large panels) and 5 µm (small panels). **(C)** Triple labeling of DPY-9::mNG, DPY-10::mSc and ROL-6::mNG. Left panels, Airyscan projection and single-channel focal planes. Right panels, single focal planes (upper) and xz projection (lower) showing DPY-10::mSc localizing more cortically and DPY-10::mNG more basally. Scales, 10 µm (large panels), 5 µm (small panels), 2 µm (xz projection).

**Fig 4:**
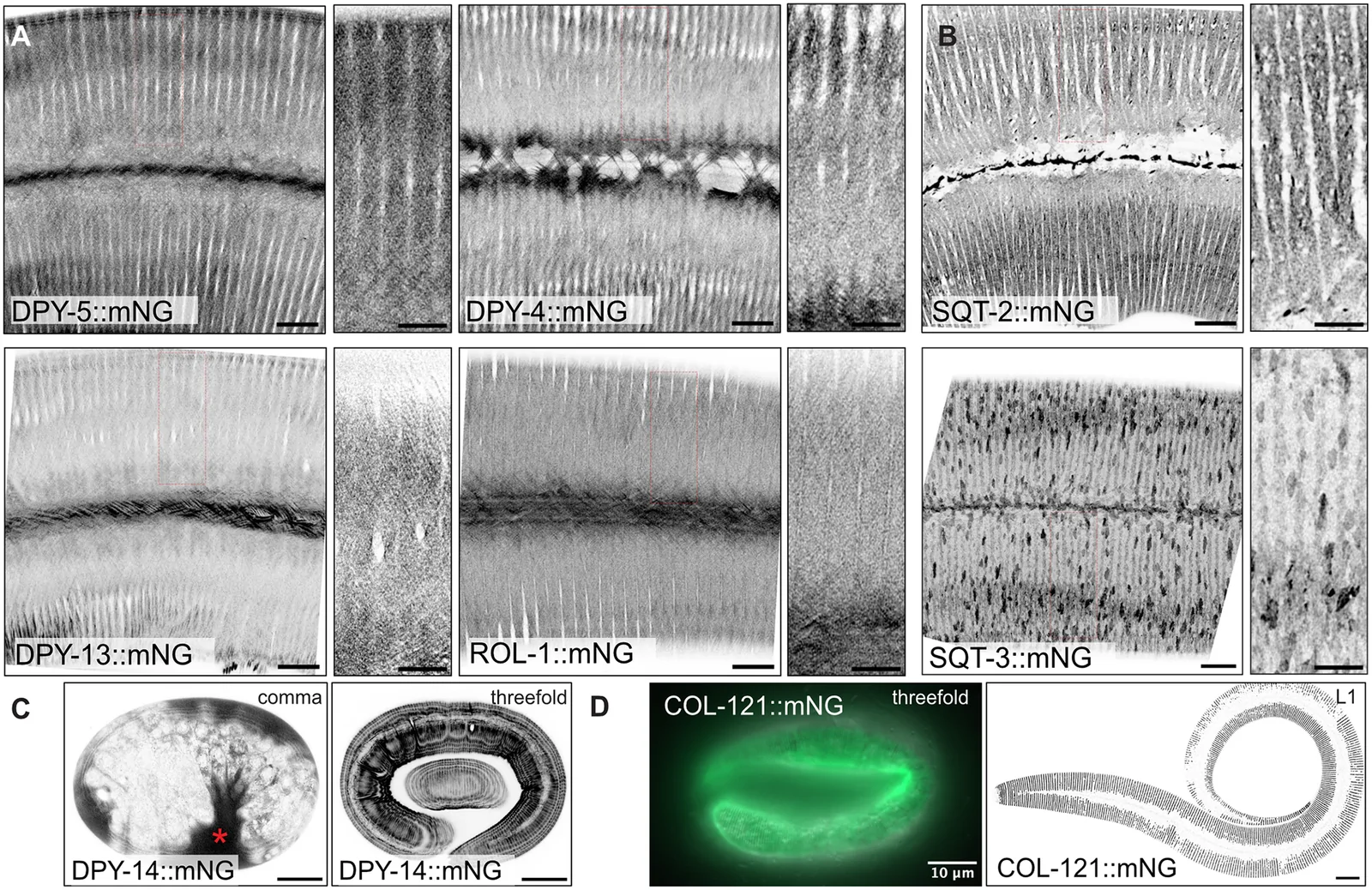
Localization of genetically defined collagens: annuli. **(A)** Representative images of fibrous annular collagens (DPY-4::mNG, DPY-5::mNG, DPY-13::mNG, ROL-1::mNG). **(B)** Images of nonfibrous annular collagens (SQT-2::mNG, SQT-3::mNG). **(C)** DPY-14::mNG in comma and 3-fold stage embryos. Note that DPY-14::mNG initially accumulates in the extraembryonic space (red asterisk) at comma stage prior to localizing to the aECM in 3-fold stage embryos, as observed for several other collagens expressed in embryos [64]. **(D)** COL-121::mNG localized to furrows in 3-fold stage embryos (widefield fluorescence and DIC) and in early L1 (Airyscan confocal). Scales, 10 µm (large panels), 5 µm (small panels).

Several collagen knock-ins, including DPY-4::mNG, DPY-5::mNG and DPY-13::mNG, were enriched in annuli, forming crossed fibrous arrays corresponding to the two fibrous layers of the cuticle [66] (Fig 4A); we classified these and similar patterns as ‘annular fibrous’. DPY-4::mNG displayed a more braided fibrous pattern within annuli (Fig 4A). These three collagens were not expressed in embryos but were upregulated in late L1 and visible as fibrous patterns in L2 onwards (S5 Fig), consistent with the lack of fibrous layers in L1 cuticle [67]. Other ‘annular fibrous’ collagens such as ROL-1::mNG were detected from late L4 stage onwards (Table 1, Fig 4A). In contrast, SQT-2::mNG and SQT-3::mNG were not overtly fibrous, instead forming smooth or punctate structures in annuli (Fig 4B); we term such patterns as ‘annular non-fibrous’. DPY-14::mNG was expressed beginning in comma stage embryos and localized to annuli in late embryonic and L1 cuticle (Fig 4C), consistent with the early larval Dpy phenotypes of *dpy-14* mutants and similar to the previously reported localization of DPY-17::mNG [62]. Three collagens originally defined by male ray abnormal morphology (Ram) phenotypes [60], as well as the novel secreted protein LON-8 [51], localized to various compartments in the male tail and other structures, discussed in more detail below (Fig 9). These observations suggest the Ram phenotype can result from aberrant function of multiple proteins in the male tail aECM. Many of the above knock-ins were readily detectable in shed cuticles, consistent with their accumulation in aECMs shed at molts. Overall, these findings suggest the genetically defined collagens are primarily localized to furrows or to the fibrous layers (Figs 1A, 3 and 4; Table 1).

In the course of this analysis, we found that *sc22,* annotated as a *rol-1* allele [68], did not contain a lesion in *rol-1* and was weakly linked to the ROL-1::mNG knock-in. Analysis of the *sc22* strain sequence revealed two other SNPs on chromosome II that would result in collagen missense alterations, including one causing R106H (RTAR > RTAH) in the *col-71* dibasic subtilisin cleavage site [69]. A *col-71(R106H)* mutation edited into a wild-type background also caused an adult Rol phenotype, suggesting this mutation is causative. The *rol-1* reference allele *e91*, which produces recessive left-hand rollers, also alters the dibasic cleavage site (RTAR > RTAC) [70]. Like ROL-1::mNG, COL-71::mNG displayed an annular fibrous localization (Figs 4A and 6A), consistent with it playing a similar role to ROL-1 in the adult cuticle.

An important question at the outset was whether collagens readily tolerate tagging. Most *C. elegans* cuticle collagens undergo N-terminal cleavage by subtilisin proteases [71] and otherwise largely consist of Gly-X-Y repeats that are critical for function (Fig 1B). Thus, we tagged most collagens at their C-termini; in a few cases, we inserted a tag within the protein. For example, SQT-3 is cleaved at its C-terminus [72]; therefore, we inserted a tag internally [62]. Several other collagens we tagged contain similar C-terminal cleavage sites (e.g., DPY-4, DPY-13, ROL-1; see S1 File for sequences), however C-terminal knock-ins in most cases appeared to preserve function. Of the cuticle collagens defined by mutant phenotype, we detected overt phenotypes in four knock-ins: DPY-4::mNG and DPY-8::mNG were mildly Dpy, and COL-34/RAM-4::mNG and COL-82/RAM-2::mNG males displayed Ram phenotypes, suggesting these insertions affect gene function. The SQT-1::mNG allele suppressed the Bli phenotype of *bli-2(0)*, consistent with it impairing *sqt-1* function [24]. As mentioned above, animals homozygous for DPY-2::mNG and DPY-9::mNG knock-ins displayed fragmented furrow collagen localization (Fig 3B) but were not overtly Dpy. While loss of furrow collagens results in a compromised aECM permeability barrier [65], we found that animals homozygous for DPY-3::mNG or DPY-10::mNG exhibited no such defects. These results indicate that the endogenous C-terminal tags do not significantly interfere with the physiological roles of these proteins. In contrast, animals homozygous for DPY-5::mSc and DPY-13::mNG resembled single homozygotes in expression and localization, suggesting synthetic effects may be specific to certain knock-in combinations. Although this analysis was not exhaustive, it suggests most cuticle collagens tolerate C-terminal knock-ins; in certain cases tagging may result in cryptic loss- or reduction-of-function that can be detected in sensitized backgrounds.

Most of our analysis relied on homozygous knock-in strains. To assess how tagged collagens compete with untagged proteins in the heterozygous state, we examined a set of 7 collagen knock-ins expressed at varying levels. Interestingly, the fluorescence intensities of cuticle mNG of most knock-in heterozygotes (5/7), when scaled according to copy number, were significantly above those of homozygotes; 2/7 (*col-81*, *sqt-2*) were not significantly different (when scaled) from homozygote intensity (S6A Fig). With the caveat that we have not assessed protein levels, these comparisons suggest tagged collagens can be incorporated as efficiently into the ECM as untagged alleles. Most KIs exhibited minimal recovery in FRAP experiments except for LON-8::mNG in the L4 vulva (S6B Fig), consistent with tagged proteins being stably incorporated into the matrix.

Many knock-ins were visible within underlying epidermal cells, localized to puncta that likely correspond to secretory organelles [24] (‘punctate’ in Table 1). Some mNG-tagged collagens accumulated in puncta within the epidermis and showed little cuticle fluorescence; in these cases, tagging may have interfered with secretion and the mNG-tagged proteins may be aberrantly retained in secretory compartments. Some mSc-tagged knock-ins showed intense fluorescence in filamentous structures that resemble epidermal lysosomes [73]. It is unclear if these reflect normal endocytosis or aberrant trafficking of tagged proteins.

### Other collagens and cortical aECM compartments

In addition to the above genetically defined collagens, we tagged an additional 35 cuticle collagens and a noncuticle collagen (*col-135*). As mutant phenotypes were not known for most of these, functionality of the tagged proteins could not be verified. All were detectable using dissecting or widefield microscopy. While some showed patterns reminiscent of genetically defined collagens, many showed distinctive localization especially to cortical layers. We summarize these patterns by compartment:

#### Furrow or furrow-flanking.

Four other collagens consistently localized to furrow-like patterns (Figs 5A and S7) resembling the six known ‘furrow’ collagens, although none were as specific as the furrow collagens. These new furrow-localizing or furrow-flanking collagens included COL-81::mNG and COL-103::mNG (thick furrows). Two proteins showed distinctive ‘furrow-flanking’ localization to double bands immediately adjacent to the morphological furrows defined by DIC: the cuticlin CUT-2::mNG and the adult-specific collagen COL-63::mNG (Fig 5B and 5C). The COL-63::mNG double-band pattern was reminiscent of the localization of BLI-1::mNG and BLI-2::mNG knock-ins to furrow-flanking bands prior to their condensation into punctate struts [24], however COL-63::mNG double bands persisted into adulthood, suggesting they define a distinct compartment of the adult furrow substructure. In *dpy-3(e182)* mutants COL-63::mNG and CUT-2::mNG had a punctate or vermiform appearance (Fig 5B and 5C), indicating their localization is dependent on normal furrow formation.

**Fig 5:**
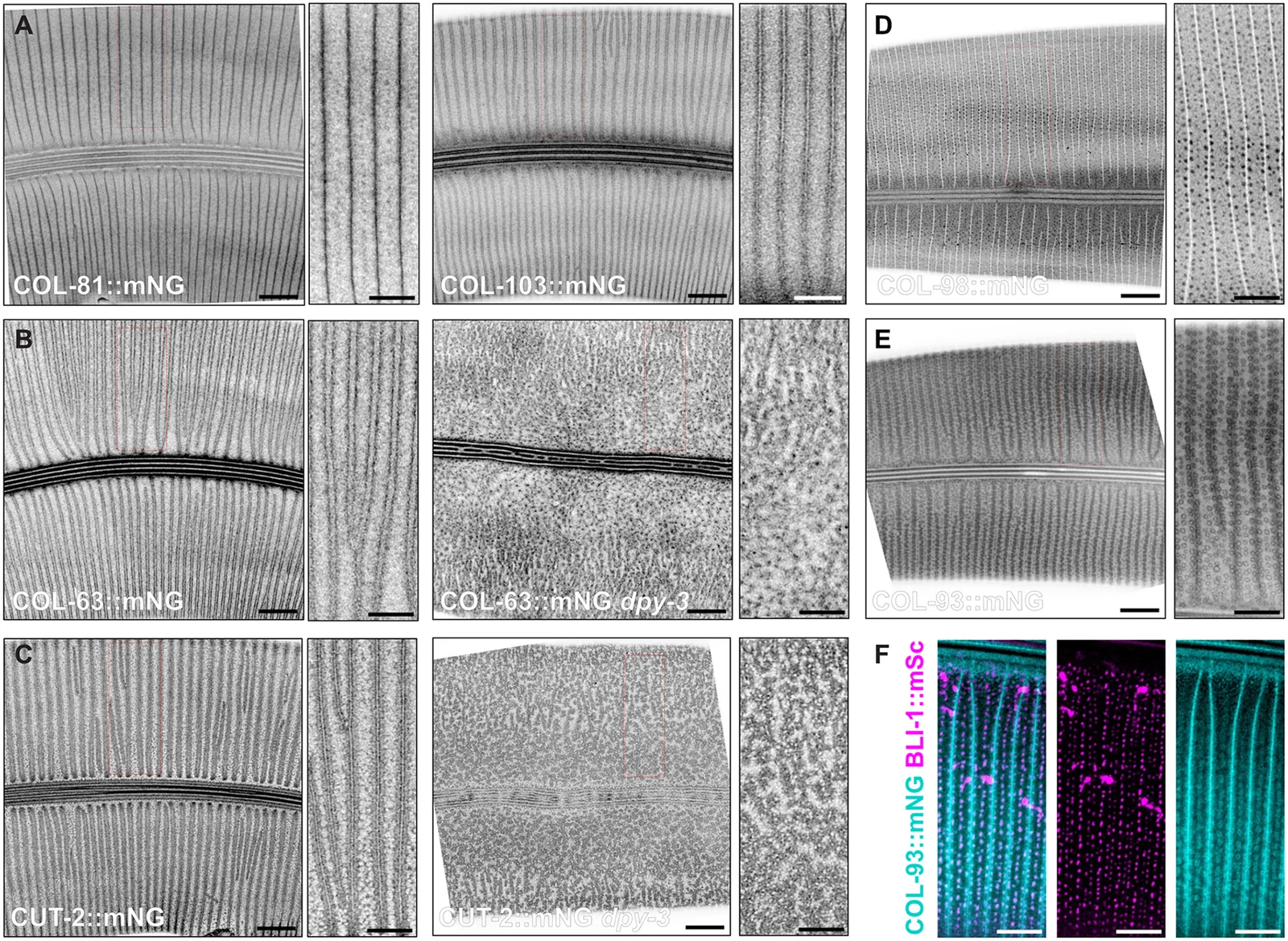
Novel Furrow, Furrow-flanking, and Strut-related patterns. **(A)** Airyscan confocal projections of COL-81::mNG and COL-103::mNG showing localization to furrow or furrow-flanking patterns, based on spacing and orientation relative to morphological furrows. **(B)** COL-63::mNG localization to furrow-flanking pattern. In *dpy-3* mutants, the COL-63::mNG furrow-flanking pattern was converted to a dispersed punctate pattern. *dpy-3* animals displayed slightly aberrant lateral alae, consistent with DIC observations [68]. **(C)** CUT-2::mNG localization was similarly aberrant in *dpy-3* mutants but was less punctate than COL-63::mNG. Airyscan confocal. Scales, 10 µm (large panels) and 5 µm (small panels). **(D, E)** Airyscan confocal projections of COL-98::mNG and COL-93::mNG (Airyscan). COL-103::mNG also localizes preferentially to furrow-flanking struts, more evident in basal sections (S7 Fig). **(F)** Co-localization of COL-93::mNG and BLI-1::mSc. COL-93::mNG circles surround BLI-1::mSc puncta in a z-projection (left panel). Scales, 10 or 5 µm. Panels in F are 4 µm wide.

#### Struts.

Struts are column-like connections between basal and cortical layers, formed in late L4 and containing three collagens (BLI-1, BLI-2, and BLI-6) [24] and two epicuticlins EPIC-1 and EPIC-2 [14]. We found several additional collagens that localized to adult-specific puncta corresponding to struts (Figs 5D – 5F and S8), although none were exclusive to struts. COL-98::mNG displayed faint strut-like puncta as well as diffuse localization in annuli (Figs 5D and S8). COL-93::mNG showed a unique localization to rings around strut bases (Figs 5E, 5F and S8). Compared to BLI-1 or EPIC-1-labeled strut ‘donuts’, COL-93::mNG rings were larger (0.44 ± 0.07 µm diameter, mean ± SD, *n* = 122) and slightly more basal than BLI-1::mNG (Fig 5F). COL-93::mNG localized basally in larval cuticles and did not display furrow/strut localization until after the L4-adult molt. COL-80::mNG localized to furrows and annuli in the L4 stage; additional strut-like puncta developed gradually over first 48 h of adulthood (S9 Fig). COL-103::mNG and COL-81::mNG showed faint strut localization in addition to furrow localization (Figs 5A and S7). Overall, our observations indicate struts are intricately patterned substructures with at least 9 distinct protein components, several of which may be recruited in the adult stage.

#### Fibrous patterns.

Several collagens displayed ‘annular fibrous’ patterns resembling those of the genetically defined collagens DPY-4/-5/-13 or ROL-1 (Fig 6A). The adult-specific collagen COL-19 was one of the first to be GFP-tagged in transgenics [74], but details of its localization had not previously been analyzed. COL-19::mNG localized to oriented fibrous layers [24] and to less consistently oriented fibers deep in the lateral cuticle. COL-20::mNG and COL-124::mNG displayed similar patterns (Fig 6A). COL-38::mNG and COL-138::mNG both displayed adult-specific annular fibrous organization (S10A Fig). Notably, transcription of both *col-38* and *col-138* is activated in the late L4 stage by the zinc finger transcription factor LIN-29, an important heterochronic pathway regulator [75,76]. Although the function of these adult-specific fibrous collagens is not known, they may contribute to adult cuticle resilience.

**Fig 6:**
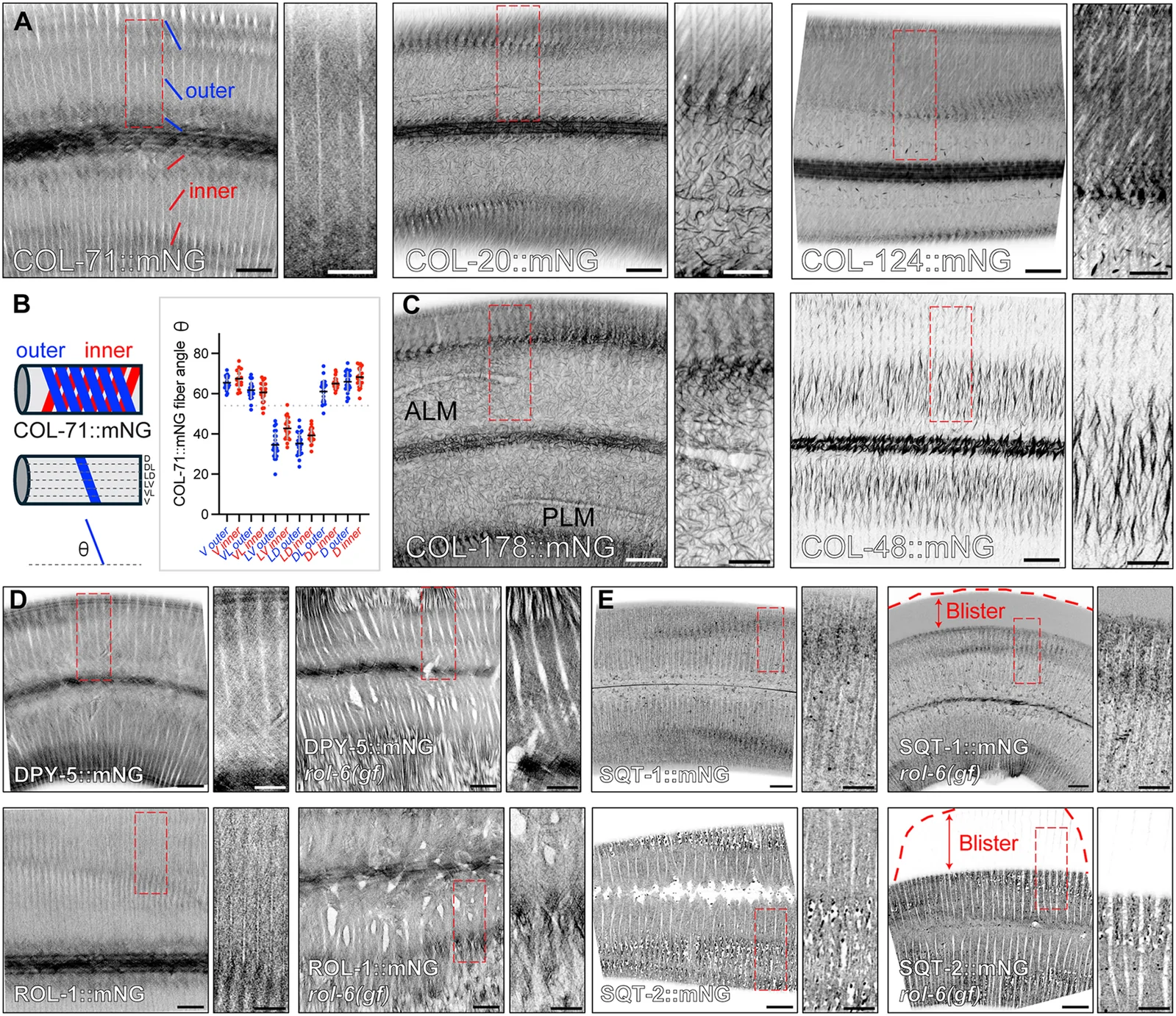
Markers for the oriented fibrous arrays in basal layers. **(A)** Airyscan confocal projections showing fibrous layer localization of COL-71::mNG (both layers; single confocal slice), COL-20::mNG (outer layer), and COL-124::mNG. Deeper COL-20::mNG fibers (lateral epidermis; lower part of inset) are not consistently oriented (see also S10B Fig). **(B)** Cartoon of fibrous array chirality. The outer fibrous layer forms as a right-handed helix (blue); the inner layer is left-handed (red), consistent with prior observations [66]. COL-71::mNG is found in both layers, whereas COL-20 is predominantly in the outer layer based on its right-handed helical pattern. Quantitation of angle θ of fibrous arrays to the long axis at different points in the dorsoventral axis in outer and inner layers, measured for COL-71::mNG in midbody at different distances from lateral midline defined as: V, ventralmost; VL, ventrolateral; LV, lateroventral, LD, laterodorsal, DL, dorsolateral, D, dorsalmost (see Materials and methods). Mean ± SD, *n* = 20−25 for each set. See S1 Data for the data underlying the graph. **(C)** Other examples of fibrous collagen patterns: COL-178 forms dense filigreed patterns in the basal cuticle, as well as faintly localizing to oriented fibrous layers. COL-48::mNG forms short circumferentially oriented fibers, especially under lateral alae. **(D)** Mislocalization of fibrous layer markers DPY-5::mNG and ROL-1::mNG in *rol-6(gf)* animals. **(E)** SQT-1::mNG or SQT-2::mNG markers cause Bli phenotypes in *rol-6(gf)* backgrounds. Scales, 10 µm or 5 µm (insets).

Most such ‘annular fibrous’ collagens localized to crossed fiber arrays. By examining closely spaced focal planes (see Materials and methods), we determined that in such crossed fiber arrays outer (more cortical) fibers formed right-handed helices whereas the inner array formed left-handed helices, consistent with earlier EM observations [66,77]. In the midbody, the angle of these fibers in either array to the long axis varied along the dorsoventral axis, with lateral fibers forming shallower angles (30–40°) compared to more dorsal or ventral regions (up to 80°) (Fig 6B), whereas in the anterior head the arrays were more consistently angled at ~65° to the long axis. Although most fibrous collagens were in both the outer and inner fibrous arrays, some appeared to selectively label a single array. For example, COL-20::mNG appeared predominantly in the outer right-handed array as well as in curving variably oriented fibers deeper in the cuticle (Figs 6A and S10B); the latter may represent the basal-most fibrillar layer. Some collagens displayed fibrous patterns distinct from the crossed fiber arrays (Fig 6C). These included COL-48::mNG, which localized to circumferentially oriented bundles of fibers, and COL-178::mNG, which localized to long thick curved fibers (Fig 6C).

Mutations in multiple collagens result in ‘Roller’ (Rol) phenotypes in which the cuticle and underlying tissues are helically twisted, potentially due to aberrant fibrous layers [68]. We therefore examined fibrous layer markers in Rol mutant backgrounds. Such markers were variably mislocalized in *rol-6(gf)* backgrounds (Fig 6D and 6E); however, crossed fibers remained visible (Fig 6D), suggesting Rol mutations may indirectly affect fibrous layers. Interestingly, introduction of SQT-1 or SQT-2::mNG in Rol background frequently resulted in Bli phenotypes (Fig 6E), suggesting these KIs have cryptic effects on function. Speculatively, such combinations could impair fibrous layer attachment to struts.

#### Annular and diffuse localization.

Several collagens showed annular localization but were not overtly fibrous (Fig 7A). COL-91::mNG displayed a slightly granular localization within annuli, reminiscent of EPIC-1 or EPIC-2 proteins [14]. COL-130::mNG was more concentrated at the furrow-flanking borders of annuli whereas ROL-8::mNG was more patchily distributed; COL-125::mNG and COL-149::mNG were concentrated along the central annular ridges (Fig 7A). Some knock-in strains displayed localization that was less easily classified due to their low fluorescence level (e.g., COL-39::mNG, COL-49::mNG) but may be diffusely localized within multiple compartments.

**Fig 7:**
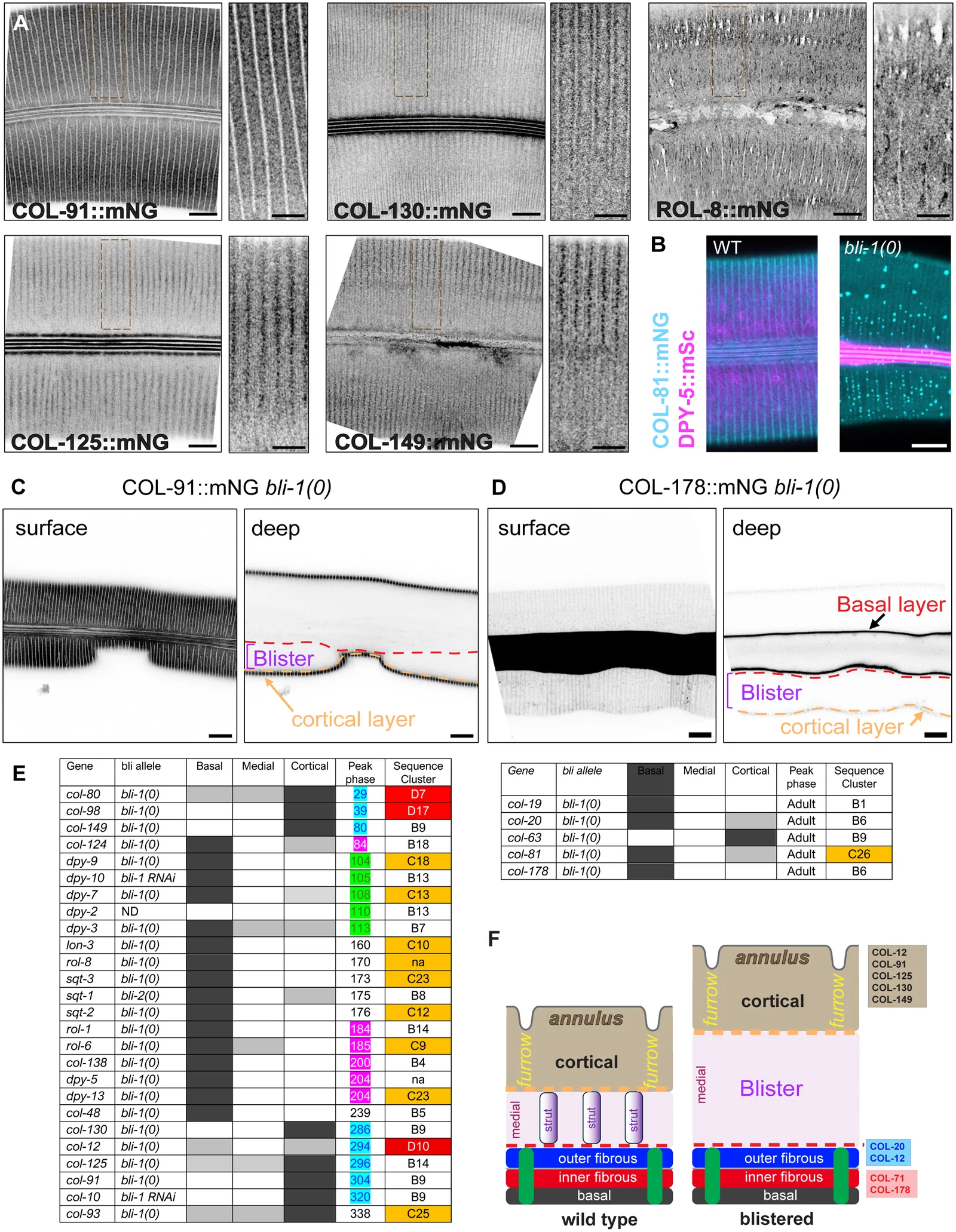
Non-fibrous annular and cortical compartments. **(A)** Airyscan confocal projections showing annular localization patterns: COL-91::mNG and ROL-8::mNG diffusely localized throughout annuli. COL-130 localized to the furrow-flanking regions of annuli. COL-125::mNG and COL-149::mNG were concentrated in the central region of annuli. **(B–D)** Cortical vs. basal localization in blistered mutants. (B) Double labeling of COL-81::mNG and DPY-5::mSc in wild-type and *bli-1(0)* mutants. In *bli-1* mutants COL-81::mNG remained on the cortical surface and became punctate; DPY-5::mSc is basal. Scale, 10 µm. (C) Surface and deep confocal sections of COL-91::mNG (C) and COL-178::mNG (D) in *bli-1(ju1395)* null mutants. Blistered regions (purple brackets) are demarcated based on their faint fluorescence visible when imaging gain is increased (see S11 Fig for additional examples). COL-91::mNG localized exclusively to the outside of blistered regions (cortical) and COL-178::mNG localized exclusively to the inside (basal). Cortical surfaces in deep sections marked with orange dashed line, basal surfaces with red dashed lines. Scales, 10 µm. **(E)** Table of cortical vs. basal localization organized by peak phase of transcript level. Shading indicates strong (dark gray), weak (light gray), or no fluorescence (white) in the respective layer in blistered regions of adult Bli animals. For collagens whose transcript levels oscillate, the peak phase angle is aligned to the molt at 0° [78]. Five collagens are adult-specific (Adult). Cyan lettering indicates cortical localization, green indicates furrow localization, and magenta indicates basal localization. Cortical collagen transcripts peak both before and after ecdysis; these may represent a single wave of expression for the subsequent cuticle, or may represent distinct early and late waves [79]. Localization of DPY-7::sfGFP and COL-19::mNG in *bli* mutants has been previously reported [24]. Collagen sequence clusters are based on domain organization and do not strictly correlate with sequence identity [25]; cluster D is in red and C in orange. **(F)** Cartoon summary of cortical and basal layer localization data from Figs 5 and 6. Right, schematic of separation of adult cortical and basal layers in blistered mutants.

#### Cortical versus basal compartments.

To assess whether a specific knock-in fusion protein localized to cortical or basal compartments, we used Bli mutants in which cortical and basal layers become more widely separated in adults. We previously used this approach to show that COL-19::mNG and DPY-7::mNG both localized basally in blistered animals [24]. We generated further double-label strains using cortical mNG-tagged knock-ins and the basal marker DPY-5::mSc, and found that markers such as COL-81 localized in more cortical focal planes to DPY-5::mSc in *bli-1(0)* (Fig 7B). Cortical collagens such as COL-91:mNG retained their annular patterning in *bli-1(0)* (Fig 7A and 7C); basal collagens such as COL-178::mNG remained in annular fibrous patterns in *bli-1(0)* (Fig 7D). In contrast, COL-80::mNG strut localization was abolished in Bli mutants and the remaining COL-80:mNG variably localized to annuli or furrows (S9 Fig). Thus, a caveat to this approach is that disruption of struts may affect cuticle organization beyond separation of cortical and basal layers. In total, we tested 21 collagens expressed in larval stages and 5 primarily expressed in adults (Fig 7E). Using this assay, many collagens could be classified as exclusively cortical (6) or exclusively basal (1); a minority localized to more than one layer in *bli* mutants.

Many aECM transcript levels oscillate during larval development, with different transcripts peaking at different phases relative to ecdysis during each larval stage (see Introduction) [29,78,80]. When such peak phases are calibrated such that 0° corresponds to the beginning of each larval stage (ecdysis) [78] it is notable that cortically localized proteins have peak phase angles either very early (0−80°) or very late (290−360°) in each stage (Fig 7E). In contrast ‘furrow’ collagen transcripts peak early in each larval stage (100−120°) whereas basal transcripts peak later (e.g., transcripts of the 6 *sqt* and *rol* collagens peak between 170−195°). This timing would suggest the cortical collagens are expressed either just before the molt, possibly for construction of the cuticle early in the next larval stage, or soon after the molt (see Discussion). Moreover, in a limited analysis of double-labeled strains, furrow collagens appeared either more cortical or more basal than fibrous collagens depending on the protein (S12 Fig). For example, DPY-9::mNG and DPY-10::mNG localized more basally than DPY-5::mSc, although DPY-10::mSc localized more cortically than ROL-6::mNG. These observations suggest that by the adult stage ‘furrow’ collagens are components of the basal aECM. Interestingly, most collagens identified based on morphogenetic mutant phenotypes localize to furrows or basal/fibrous layers, whereas cortical collagens have not been found in phenotype-based screens (Fig 7F).

To further explore the relationship between collagen expression phasing and cuticle structure, we examined the assembly of the first cuticle in embryos. Expression of the collagens followed their mRNA phasing (Fig 7E). The furrow collagen DPY-10 (basal layer; 105º) was expressed first, appearing in the extra embryonic space (EES) at the bean stage before localizing in the cuticle at the 2-fold stage (Fig 8). SQT-3 (basal layer, 174º) was faintly detected in the EES at the bean stage and localization to the cuticle was delayed until the 3-fold stage (Fig 8). COL-125 (weak basal and medial layer localization, strong cortical localization; 296º), was not detected in the EES and localized to the cuticle in the 3-fold stage (Fig 8). Finally, COL-10 (cortical; 320º) expression was not observed until hatching (Fig 8). Together, these data highlight the complexity of cuticle assembly. While protein expression largely tracked with mRNA expression data, localization was highly variable. Some collagens such as DPY-10 and SQT-3 are secreted into the EES and remain there for 1–2 hours, as previously reported for SQT-3 [62]. Others, such as COL-125 and COL-10, immediately appear in the cuticle. These data do not support a simple “outside-in” model of assembly as basal collagens were expressed and localized to the cuticle before the cortical collagens.

**Fig 8:**
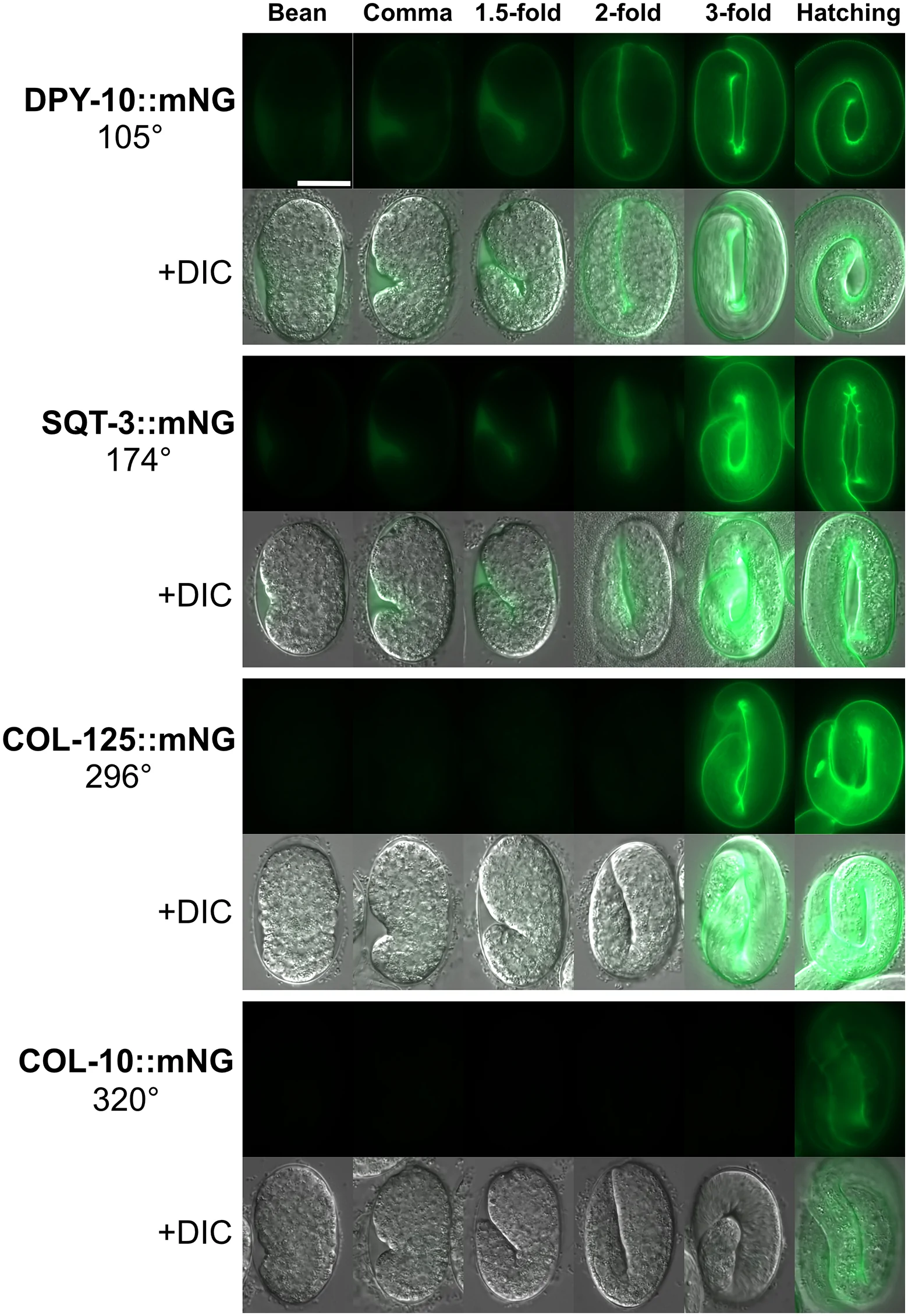
Embryonic cuticle assembly is complex and does not follow a simple “outside-in” assembly model. Expression and localization of the indicated collagen mNG translational fusions at the indicated embryonic development stages. The expression phase in degrees is provided for each gene. Two biological replicates were performed and images are representative of 20 embryos. Scale bars = 20 µm.

#### Lateral alae.

Several knock-ins localized to alae, ridges that extend longitudinally along the lateral cuticles of L1s, dauer, and adults [23,81]; selected examples are in S13 Fig. Adult alae localization could be broadly classified as localization to the three alae ridges (e.g., COL-125::mNG, COL-130::mNG), to the flanks of ridges (six longitudinal stripes; e.g., COL-103::mNG; COL-63::mNG), or to the valleys between the ridges (2 or 4 stripes; e.g., COL-81::mNG, CUT-2::mNG). Some collagens localized to deeper fibers beneath alae (e.g., COL-14::mNG). Several collagens also localized to the alae of L1 or of dauers (S13B and S13C Fig).

#### Specialized aECMs of interfacial cuticles and other tissues.

In addition to the body cuticle compartments defined above, many collagens are specifically localized to specialized interfacial aECMs such as those in the nose tip (including the mouth and amphid and other sensilla), vulva, rectum, excretory pore/duct, or pore-forming sensilla such as the phasmids and deirids (Fig 9A). For example, RAM-4/COL-34::mNG and COL-174::mNG were detected in many interfacial cuticles (Fig 9B and 9C). RAM-1/COL-64::mNG localized exclusively to male tail rays, whereas RAM-2/COL-82::mNG and RAM-4/COL-34::mNG also localized to the fan aECM and caused Ram phenotypes (Fig 9D). Numerous collagens localized to distinct regions of the vulval cuticle (Fig 9E). These observations reinforce findings that interfacial aECMs are highly specialized due to local secretion of aECM [19,20] or local determinants of assembly [82]. Although COL-51, COL-92, or SQT-2 have been hypothesized to function in an intestinal aECM, we did not detect intestinal localization in these or other collagen KI strains [83,84]. Other studies suggest cuticle collagens may be minor components of gonadal ECM, albeit only detectable using overexpression transgenes [85]. Indeed, all tagged cuticle collagens were found in epidermis or cuticle, apart from the divergent collagen COL-135, which was expressed in spermatheca and uterus (S14 Fig) and therefore may be a component of the little-studied uterine matrix [86].

**Fig 9:**
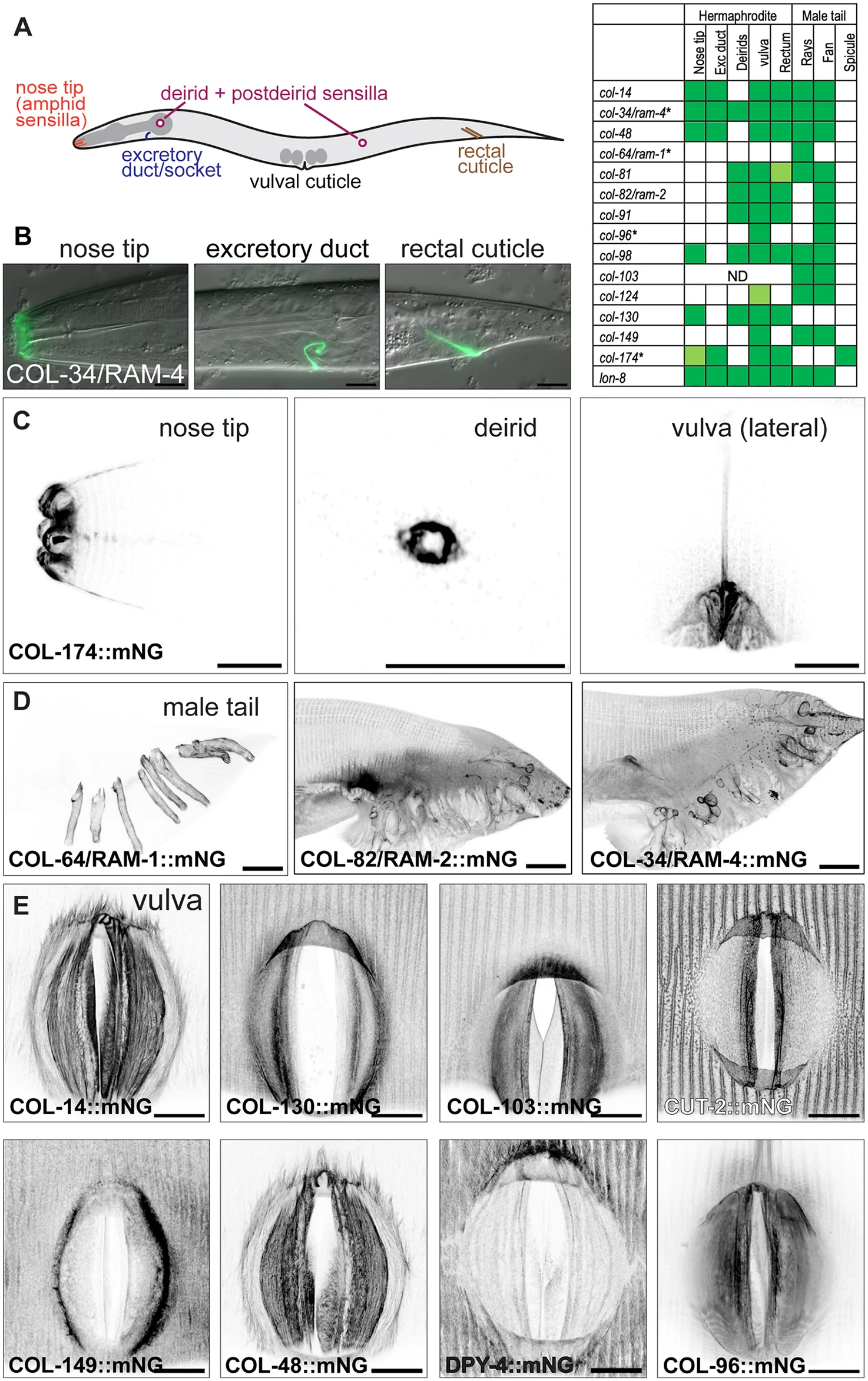
Localization of collagens and non-collagens to interfacial aECMs. **(A)** Cartoon of interfacial aECMs in adult hermaphrodites, showing nose tip (mouth, buccal cavity, amphid pores), excretory pore and duct, vulva, rectum, deirid and postdeirid. Right, heat map table of localizations of selected knock-ins that are primarily expressed in interfacial aECM. **(B)** COL-34/RAM-4::mNG localization to nose tip, excretory duct, and rectal cuticle; widefield fluorescence and DIC. **(C)** COL-174::mNG localizes specifically to interfacial cuticles; shown is localization of the nose tip, deirid, and vulva (lateral view) Scales, 10 µm for nose tip and vulva, 2 µm for deirid. **(D)** Localization of RAM collagens (COL-64/RAM-1, COL-82/RAM-2, COL-34/RAM-4) to male tail ray and fan aECMs. **(E)** Localization of COL-14, COL-130, COL-103, CUT-2, COL-149, COL-48, DPY-4, and COL-96 mNG knock-ins to vulval cuticle in early adult, ventral views; Airyscan confocal projections. Scales, 10 µm (B, D), 5 µm (E).

## Discussion

We report the first large-scale set of endogenously tagged aECM components in any organism. Our set of molecular markers reveals the exquisite tissue and stage specificity of aECMs, and the multiscale patterning of aECM substructures. Our work has implications for CRISPR methodology, for the molecular architecture of the *C. elegans* aECM, and for the spatiotemporal organization of complex ECMs.

### Molecular architecture of apical ECMs and regulation of aECM expression

Among the *C. elegans* aECMs, the cuticle alone likely contains hundreds of components. We consider whether, comparable to basement membranes, the cuticle aECM may be dissected into ‘core’ components expressed at all or most stages and cuticle types, to which are added stage and region-specific elements. Of the genetically defined cuticle components, some were expressed in all stages, and may form a ‘core’ cuticle aECM to which additional stage or structure-specific components are added. These include components of annuli such as SQT-3, and several furrow collagens, consistent with the presence of annuli and furrows in all body cuticle types. However, our gene set was slightly biased toward collagens expressed in a subset of larval stages; of ~20 collagens expressed at all stages by RNAseq we have only tagged SQT-3, COL-12, and RAM-4/COL-34, which is found in interfacial cuticles.

Clear examples of stage-specific components include the L1 collagens DPY-14 and DPY-17, the L2-4 stage collagen COL-41, and the numerous ‘adult specific’ collagens. It should be clarified that stage-specific collagens are first expressed in the stage preceding the stage in which they are incorporated into matrix: i.e., many ‘adult specific’ collagens are first transcribed in L4, secreted in late L4, and form stable structures in the adult cuticle [24]. Our knock-in analysis was consistent with the L4 upregulation of four other collagens known to be targets of the LIN-29 transcription factor [75]. Some aECM transcripts are first detected in L4 and are present at higher levels in adults, whereas others are first detected in adults. Interestingly, some genes such as *col-12* show an additional transcript peak early in adulthood [79], and our protein analysis was consistent with this observation. The mechanistic basis of the early adult peak is not known but could be related to larval transcript oscillations. Several proteins such as EPIC-1 [14] display distinct localization in adult versus L4 cuticle, suggesting the adult cuticle undergoes maturation well beyond the L4/adult molt.

We found several collagens that localize exclusively to the cortical compartment of the cuticle. In contrast, most genetically defined collagens localize to more basal or fibrous layers. The cortical compartment is an exciting area for investigation as it may contribute to surface properties involved in host-pathogen interactions or mate recognition [18]. Equally, the architecture of the fibrous layers is little-understood. Crossed fibrous arrays are integral to the strength of the hydrostatic skeleton in many nematodes and other animals [87], however, little is known about their biogenesis. Our markers reveal that the crossed fibrous arrays are variably angled with respect to the body axis, reminiscent of variable-angle composites that maximize load-bearing capacity [88]. Moreover, we identified collagens unequally distributed between the two fibrous layers. Aberrant patterning of such fibrous layers has long been hypothesized to underlie helically twisted ‘Rol’ mutant phenotypes [68]. Our resource provides tools to address how fiber angle is regulated and how the two fibrous arrays are laid down with consistent chirality [77].

The dauer cuticle is highly specialized, with unique structures such as a basal nanoscale mesh that may contribute to stress resistance. Transcriptomic studies show ~30 cuticle collagens are transcribed predominantly in dauer or predauer stages [89]. We did not prioritize such dauer-specific collagens as their low expression in nondauer stages made them more challenging for fluorescence-based screening. Two dauer-specific collagens tested, COL-43 and COL-51, indeed were only visible in dauer and/or predauer cuticle. Additionally, several proteins expressed in other stages showed distinctive localization in dauer larvae, e.g., COL-48 (dauer deirids) or LON-8 (dauer alae)(S13 Fig). An important future direction would be to focus on the dauer-specific aECM and our *unc-119(+)* pipeline may be best suited for these genes.

### Collagen localization determinants and timing

Our analysis indicates that collagen localization in the aECM shows a high degree of specificity, in that almost every collagen knock-in showed a slightly different pattern. Most *C. elegans* cuticle collagens are 300–400 aa; after cleavage, mature cuticle collagens mostly consist of Gly-X-Y domains and short N- and C-terminal regions. Cuticle collagens have been classified in several ways, such as the patterns of interruptions in the Gly-X-Y domain and the presence of a signal peptide or transmembrane domain [25]. It remains unclear which sequence features might direct localization. Interestingly, of four tagged collagens classified in cluster D [25], three were cortical (Fig 7E). Our toolkit provides a resource for the dissection of determinants of collagen localization.

Timing of aECM protein expression could provide additional information. Within each larval stage aECM transcription occurs in waves [79], resulting in oscillating transcript levels [28] and presumably phased secretion of aECM components. Such observations have led to models that the cuticle could be built in successive layers, with early-peaking proteins forming outer layers and later-peaking proteins forming inner layers. Such models are an oversimplification given the early expression of precuticle proteins [10] and the lag between collagen secretion and aECM assembly in the embryo [62]. Nevertheless, our findings confirm and extend previous evidence that transcription phases correlate with cuticle compartment, for example, precuticle proteins and furrow collagen transcripts peak prior to basally localized collagen [50]. Interestingly, transcripts for cortically localized collagens peak either very early (*col-80, col-98,* and *col-149*) or very late (*col-12, col-91,* and *col-93*) in the molting cycle. One possibility is that these are all deposited in the cuticle early in the molt cycle, consistent with the simple ‘outside-in’ model. Alternatively, the early-peaking cortical collagens may form an initial outer cortical layer, and the later-peaking cortical collagens may be somehow inserted after formation of basal layers. Our embryo imaging data (Fig 8) are consistent with this model as the cortical layer collagen, COL-125, is expressed late in embryogenesis, well after the basal layer collagens DPY-10 and SQT-3. COL-10 also follows this trend (Fig 8), though it is predominantly cortical with weak basal and medial expression (Fig 7), highlighting the complexity of collagen localization. EM images of the nascent adult cuticle in the late L4 stage suggest that a basal layer is present before the cortical layer reaches its full thickness [14]. Thus, cortical layers may be assembled in two or more phases. Electron microscopy studies suggest nascent furrow regions in the cortical and basal layers are compressed together, although they later become widely separated in the adult due to development of the intervening medial layer. An important future direction facilitated by this toolkit will be to more precisely define the order of assembly of aECM compartments and their relationship to the phased transcription of aECM genes.

For the current toolkit, we prioritized collagens, in view of the available genetic reagents and our finding that most cuticle collagens tolerate C-terminal tagging. Collagens make up about half the *C. elegans* cuticle by mass [66]. Less is known about noncollagenous proteins such as cuticlins or epicuticlins, components of the outer cortical layers [14,90]. Tagging of Hedgehog-related proteins has also revealed specific localization to substructures of the cuticle or precuticle [19,91,92]. Using collagen localization as a reference, tagging of additional noncollagen aECM components such as ZP domain cuticlins or lectins should be highly informative. Although *C. elegans,* like other nematodes [93] has an especially large set of collagen genes, mammals also have numerous collagens (~28 types, ~44 genes) [94] whose localization mostly remains little-explored.

### Optimization of CRISPR for large-scale knock-in projects

Our aECM toolkit establishes numerous lessons to establish large-scale tagging projects. Our fastest screening approach involved RNPs, PCR-derived linear repair templates, and screening under a fluorescent dissecting scope. We were most successful with genes expressed at high levels (>1,000 transcripts per million in a single-cell RNA-seq data set [95]) and in large tissues such as the epidermis. We detail failed edits in S1 Table. While we prioritized speed and hitting our target number of strains, revisiting these failed edits will be valuable for further refining our editing pipeline. Interestingly, neither *dpy-10* co-conversion nor a dominant *rol-6(su1006)* co-injection marker helped enrich for aECM knock-ins, perhaps because such cuticle mutants display genetic interactions with aECM target genes. KIs with spatially or temporally restricted expression were much easier to identify with our selection-based approach.

Approximately 10% of the genetically defined cuticle collagen strains displayed overt or cryptic phenotypes, suggesting C-terminal tagging of most collagens is mostly tolerated. Some collagens, such as SQT-3 undergo C-terminal processing by the protease DPY-31 [72]. Although we avoided tagging SQT-3 at the C-terminus, other collagens with predicted C-terminal cleavage sites (see Materials and methods) appeared to tolerate tagging. Some collagens proved challenging to tag due to lack of unique crRNA sites (see Materials and methods). Our C-terminal QUA-1 knock-in was likely processed and an internal tagging attempt broke QUA-1. While our RNP and *unc-119(+)-*based selection approaches are high-throughput, SEC-based selection [96] with longer homology arms could be a useful alternate approach to tagging proteins that have highly similar homologs, which poses a challenge when using 35–65 bp homology arms.

Several groups have recently reported parallel efforts to optimize *C. elegans* protein-tagging knock-ins, either using CRISPR/Cas9 or recombinases such as FLP or phiC31. CRISPR/Cas9 has been used to generate KIs in 30 distinct genes with 3 different FP tags inserted per injection [97]. This approach has the potential to accelerate the workflow with a tradeoff of needing to use green, red, and blue FP KIs in each injection. Recent recombinase-based systems create an initial landing pad that can be efficiently re-edited [98,99]. While similar to our CRISPR-based modular knock-in approach, recombinases are not adversely affected by the size of the insertion, so can insert larger cargo, and additionally the recombinase methods allow tag swapping by genetic crossing. Ideally, a combination of an initial fluorescent knock-in carrying recombinase sites could allow functional initial strains combined with efficient modular re-editing.

### Uses of the resource and prospects for future tagging resources

Our resource provides the first large-scale overview of aECM protein localization. Our resource can address key questions in aECM biology such as how the aECM is re-established in wound healing, how it is maintained during normal adult aging, how proteases and protease inhibitors coordinate its remodeling, and how specific aECMs are assembled in a cell type-specific manner [82]. Importantly, pathogenic variants [100] can now be modeled using proteins expressed at physiological levels. The multiscale patterning of the ECM remains a major challenge in mammalian biology. Protein-tagging knock-ins have been reported for a small number of mammalian collagens, primarily those of the basement membrane [101]. Our studies suggest tagging of other collagens and matrix components could reveal unanticipated aspects of ECM architecture and function. The human genome contains ~45 collagen-encoding genes in ~28 families; pathogenic variants have been found in at least 17 collagen families [102]. Imaging collagen patterning in normal and disease states may inform understanding of such collagen disorders.

The *C. elegans* matrisome includes ~700 members of conserved matrix families [25], including receptors. Other potential matrix components are not highly conserved in primary sequence but are conserved in terms of intrinsic disorder propensity or low sequence complexity. For example, the *C. elegans* pharyngeal cuticle contains numerous intrinsically disordered proteins [103], and epicuticlins consist solely of low complexity repeats [14,104]. Taking 700 as a lower bound of protein species in the *C. elegans* ECM, at most 25% have been tagged in this work and other projects [7]. The matrisome itself is a small fraction of the ‘secretome’ defined as all proteins normally secreted into extracellular compartments, ~3500 proteins in humans [105]. The distinction between matrisome and secretome proteins is not rigid, and proteins may form a continuum of stability or diffusibility within extracellular space. Localization of most secretome proteins has not been investigated using protein tagging, but has been proposed as a priority for genome-scale tagging [106]. Such approaches could be highly informative for understanding the architecture of the extracellular environment in development, adult homeostasis, wound healing, and aging.

### Limitations of the study

Our toolkit is based on tagging endogenous loci with fluorescent protein tags, and thus conclusions are based on the localization of the tag itself rather than the endogenous protein. We detected an effect of tagging on gene function in a small minority of cases; however, it is possible that many more tags have subtle effects on function not detectable in the single mutants. Fluorescent tags could alter stability or localization of the tagged protein. In most cases, we used a single tag site and have not validated multiple sites. We have validated numerous knock-ins at the site of insertions but have not searched exhaustively for aberrant editing. Off-target effects of CRISPR/Cas9 editing in *C. elegans* are very rare, but complex alterations at the editing site are sometimes seen [107]. In any event, conclusions based on a single knock-in strain should be treated with caution.

## Materials and methods

### *C. elegans* genetics

*C. elegans* were maintained at 20 °C on NGM plates seeded with *E. coli* OP50, using standard procedures unless stated. Mutations were confirmed by PCR or sequencing. Strains used in this study are listed in S4 Table.

### Molecular biology

We designed flexible 39 bp 5′ and 3′ homology arms encoding N-SSSSSSGGSGGSA-C and N-AASSGSSSGGSGG-C linkers, respectively. These arms were added to the mNeonGreen::3xFLAG from pJW2172 [108] by PCR and the products were Gibson-cloned into pDONR221 to generate pJW2332. We also designed an mNeonGreen::AID*::3xFLAG equivalent for tagging proteins with nuclear or cytoplasmic domains using a pJW2171 template to generate pJW2333. These cassettes include 4 unique crRNA sites to allow re-editing. pJW2332 was linearized by PCR, introducing silent mutations in the PAM to disable the internal crRNA sites. We then cloned in SNAPTag and HaloTag cassettes to make pJW2335 and pJW2336, respectively. We also designed a set of mScarlet cassettes to replace the modular mNeonGreen knock-ins. First, we replaced the 3xMyc cassette from pJW2072 (30 amino acid linker::mScarlet(GLO)::3xMyc::10 amino acid linker) [108] with 3xHA and 3xOLLAS tags through Gibson cloning to make pJW2469 and pJW2470, respectively. We then linearized pJW2335 and cloned in the mScarlet(GLO)::3xHA to make pJW2471. We attempted the same with an mScarlet(GLO)::3xOLLAS cassette but were only able to obtain a plasmid with 2xOLLAS (pJW2558). To make a SapTrap compatible modular mNG cassette, we first Gibson-cloned the modular mNG::3xFLAG cassette with the 39 bp homology arms into pDONR221 with TCC and GGT connectors for SapTrap cloning to make pJW2474. We then linearized this plasmid by PCR and used Gibson cloning to insert a syntron+ *C. briggsae unc-119(+)* cassette from pMLS271 [44] such that the cassette is in an antisense orientation to the mNG cassette, yielding pJW2476.

dsDNA repair templates with 35 bp homology arms to the genome were generated for RNP-based genome editing by PCR amplification using in house made Phusion polymerase. 8–12 50 µl PCRs were pooled, then purified using Ampure XP beads (Beckman Coulter) as described [32]. ssDNA repair templates with 35 nt homology arms were generated by PCR amplification similar to the dsDNA repair templates except that one primer was phosphorylated on the 5′ end. This phosphorylation was either performed during primer synthesis (Invitrogen) or in-house using T4 polynucleotide kinase (NEB). The phosphorylated strand was degraded using lambda exonuclease (NEB) and purified as previously described [37]. For some edits, ssDNA or 5′ biotinylated dsDNA repair templates drastically improved editing efficiency. Our cassette swaps particularly benefitted from using ssDNA or biotinylated DNA templates (Fig 2). Notably, we observed efficient editing with 35 nt homology arms with our ssDNA templates, whereas a previous report used 59–134 nt homology arms [37]. The shorter homology arms should substantially cut down reagent costs.

*unc-119*-selection based repair templates were generated by SapTrap cloning (S2 and S3 Figs) [44]. Oligos to generate a 20 bp sgRNA targeting sequence and 60 bp 5′ and 3′ homology arms were designed by SapTrap Builder [109]. We set the program to produce a C-terminal fusion and removed the “5′-CGC-3′” connector at the 5′ end of the hUS-B oligo with “5′-GGA-3′” to make this homology arm compatible with our modular toolkit. We performed similar SapTrap reactions as described [44,110] with minor modifications. To anneal the oligonucleotides, we added 1 μl of each 100 µM oligo stock (100 pmol/μl) to 8 μl of water and pipetted up and down to mix. We then added 0.5 μl of that dilution to 5 μl 10x annealing buffer (500 mM KOAc, 50 mM Tris pH 7.4) + 44.5 µl dH_2_O. We then ran an annealing program on a thermocycler consisting of 95 °C for 5 min followed by dropping 1 ºC per minute down to 4 °C. Annealed oligos were stored at 4ºC before use. We made 10x SapTrap buffer by adding 2 µl of 2.5 M potassium acetate to 48 µl of 10x T4 ligase buffer. We thawed a fresh tube of 10x T4 ligase buffer and made ~50 µl aliquots. For both the 10x SapTrap buffer and 10x T4 ligase buffer we tally each freeze-thaw on the tube as part of our quality control pipeline. We made a 10x SapTrap enzyme mix by mixing 10 µl of SapI (NEB) (pipetting up and down repeatedly before transferring), 5 µl of T4 PNK (NEB) and T4 DNA ligase (NEB). If multiple stocks were present in the lab, they were marked and used oldest to newest. We then set up SapTrap reactions with ~20 fmol of pDD379 [111] (backbone with F + E sgRNA; ~50 ng), ~20 fmol of pJW2476 (modular mNG::3xFLAG+*unc-119(+)*; ~75 ng) and 200 fmol of each annealed oligo pair (5′ homology arm, 3′ homology arm, sgRNA). While establishing an efficient editing pipeline we set up a control omitting pJW2476 and the annealed oligos. We ran the reactions on a thermocycler as follows: 37 °C for 10 min, 16 °C for 5 min followed by 30 cycles of 37 °C for 5 min, 16 °C for 5 min. There was a final 37 °C 10 min digestion followed by 65 °C for 20 min. To increase cloning efficiency, we added 1 µl of SapI (pipetting up and down before transferring) and incubated at 37 ºC for 2 hours followed by a 65 °C 20 min heat kill step. The entire reaction was transformed into DH10beta competent cells and plated on LB + Amp. We frequently screened reactions with colony PCR using M13-Rev (5′-CAGGAAACAGCTATGAC-3′) and 5′-AGCTCCTCGTATCCGTCGTTTG-3′. Colonies were picked into 50 µl of sterile water in a 12-well PCR strip or 96-well plate. 1 µl of this mixture was used as a template in a 10 µl PCR using homemade Phusion PCR and 5x Phusion HiFi buffer with green loading dye. We typically use a pJW2586 *col-75* repair template as a positive control in colony PCR reactions.

Plasmids and oligonucleotides used in this study are detailed in S5 and S6 Tables, respectively.

### CRISPR knock-ins

JDW strains were generated by the Ward lab using the following methods. crRNA, trcRNA (both from IDT), and Alt-R S.p. Cas9 Nuclease V3 (IDT 1081058) or Cas9-NLS purified protein (*S. pyogenes* WT and R691A, AsCas12a Ultra; UC Berkeley QB3 MacroLab) were mixed and incubated at 37 ºC for 15 min. Note that a range of protein and RNA concentrations were used in optimization (S1 Fig). Pre-optimization reactions used 700 ng of IDT Cas9 and a 2:5:2.5:1 ratio of crRNA:trcRNA:Cas9. Post-optimization reactions used 250 ng of IDT Cas9 and a 1:1:1 ratio of crRNA:trcRNA:Cas9. Repair template PCRs were melted as described [32] and used at 50 ng/µl. ssDNA repair templates were used at 110−250 ng/µl. We tested *dpy-10* co-conversion and pRF4 co-injection markers using previously described approaches [31,38]. We also developed an alternate co-conversion approach using insertion of a T2A::GFP cassette at the 3′ end of *myo-2* to create an endogenous promoter reporter [112]. Optimized editing used 10 ng/µl of a pSEM232 (*mlc-1p::2xNLS::tagRFP::tbb-2* 3’UTR) co-injection marker [113]. Animals were screened for mNG expression on a Leica M165 FC GFP/RFP stereomicroscope with 16.5:1 zoom ratio, 7.3-120x magnification range and GFP or GFP long pass filters, and an Excelitas X-Cite FIRE LED light source. For mSc color swaps, we similarly screened by RFP fluorescence using an mCherry filter. We generated 9 color-swapped strains as a proof of principle using a biotinylated linear dsDNA repair template and the internal crRNA sites. We chose this repair template as it boosted efficiency and we tended to get more injections out of a single prep compared to ssDNA. PHX strains were generated by SunyBiotech. We also performed 3 color swaps of PHX mNG knock-in strains.

*unc-119(+)-*based selection was performed by injecting repair template plasmids containing sgRNA (F + E) cassettes [111] at 65 ng/µl, 25 ng/µl pCFJ2474 [114] (*smu-1p::Cas9(PATCs)::gpd-2 tagRFP-T(myr, PATCs)::smu-1 3’UTR*), 10 ng/µl pSEM238 [115] (HisCl counter-selection marker), 25 ng/µl pSEM232 (*mlc-1p::2xNLS::tagRFP::tbb-2* 3’UTR) [113] into CFJ302, a temperature-sensitive *unc-119(kst33)* strain. We made use of a recently generated temperature-sensitive *unc-119* allele which further facilitates adoption of this pipeline as labs can inject into animals with a WT phenotype. *unc-119(kst33)* mutants were maintained at the permissive temperature of 15 °C, at which they are phenotypically wild-type, prior to injection. After injection, animals were shifted to the nonpermissive temperature of 25 °C at which a fully penetrant Unc phenotype manifests, unless animals are rescued with *unc-119(+).* Plates were flooded with histamine when they starved and wild-type moving animals lacking the *mlc-1p::tagRFP* marker were picked as candidate knock-ins. The selective marker was excised through injection of 10 ng/µl pMDJ20 (*smu-1p::Cre Recombinase (PATCs)::smu-1 3′ UTR*]) [114], 10 ng/µl pCFJ782 [116] (HygroR marker), 25 ng/µl pSEM232. Unc animals were picked, outcrossed to remove *unc-119(ts)*, and the knock-in recovered by either fluorescence or PCR. The *spia-1:: mNG::3xFLAG* and *bli-5::mNG::3xFLAG* knock-ins were generated using an SEC-based approach [50]. Modular mNG::3xFLAG knock-ins for *bli-1* and *noah-1* recapitulated independently generated knock-ins and have been described [117]. Reagents used for genome editing are detailed in S7 Table.

We observed an interesting variation in repair template efficiency that warrants further investigation. With our *glh-1* GFP knock-ins, we did not observe a difference in efficiency with unmodified dsDNA, 5′ modified dsDNA, or ssDNA repair templates (S1C and S1D Fig). In contrast, for *glh-1* mNG::3xFLAG to mSc::2xOLLAS color swaps the biotinylated dsDNA repair template was 10x more efficient and the ssDNA repair template was 34x more efficient than an unmodified dsDNA template, respectively (Fig 2B). As *glh-1* is an efficient edit, modification might not further improve efficiency. Notably, unmodified dsDNA repair templates allowed efficient tag swapping with other combinations of crRNAs (Fig 2C). For large-scale tagging, we used unmodified dsDNA repair templates as they were cheaper than biotinylated repair templates and faster to produce than ssDNA repair templates. However, for smaller-scale editing or edits that prove challenging such alternative templates may be a better approach.

### Sequence validation

All knock-ins were validated by PCR using primers that bind outside of the homology arms followed by Sanger sequencing. For knock-ins with *unc-119(+)* selection, we initially performed PCRs to amplify the 5′ and 3′ knock-in junctions and Sanger sequenced the PCR products. Following selectable marker excision, we PCR amplified the insertion and performed Sanger sequencing.

### Editing efficiency and troubleshooting

Our approach was to avoid lengthy troubleshooting of genes that were not tagged in an initial attempt (‘failed’) and therefore estimates of efficiency across the gene set are to be taken with caution. In total, we had 23 failed RNP-based edits and 8 failed *unc-119-*based edits, with one gene in common between the two approaches (*lrp-2*; S1 Table). Our initial *unc-119-based* knock-ins (*col-75, wrt-2*) were successful, so future efforts will focus on optimizing plasmid amount, purity, and homology arm length. Among RNP-based edits, three putative knock-ins could not be confirmed by genotyping (*col-90, col-155, cpz-2*) and will require additional analysis. For certain collagens, the presence of near-identical paralogs likely precludes efficient editing (e.g., *col-154* and *col-155*, or *col-10*, and *col-144*).

### Collagen domain organization, sequence classification, and cleavage sites

Cuticle collagen genes are defined as encoding proteins with a central block of 37−43 Gly-X-Y triplet repeats (with one or more short interruptions) and additional motifs such as a dibasic subtilisin cleavage site (Homology box A), three Cys-rich clusters, and a signal peptide or transmembrane domain (see S1 File for annotated sequences of *C. elegans* collagens). Thirty-nine collagens have potential C-terminal cleavage sites resembling that of SQT-3 (DGG motif) including BLI-6, DPY-4, DPY-13, ROL-1, and LON-3. ROL-8 has been classified as non-cuticular [25] however, it has several hallmarks of cuticle collagens, including an N-terminal dibasic cleavage site. COL-135 has an atypically large region of ~200 triplets, most of which are Gly-X-Lys [25]. COL-55 has a short Gly-X-Y region and is not represented in RNAseq data; it contains a dibasic cleavage site and a Cys-rich motif. COL-70 lacks Gly-X-Y repeats but instead contains a repeated motif GGPG(N/T)P. We excluded *col-182* as it is a pseudogene in the domesticated N2 strain [118]. We excluded known noncuticular collagens such as LET-2, EMB-9, CLE-1, MEC-5, and transmembrane proteins such as COL-99, UNC-122, and COF-2.

### Whole–Genome sequencing, sanger sequencing, and editing of *col-71*

We performed whole–genome sequencing (WGS) of the BE22 strain, containing the roller allele *sc22*. Genomic DNA was prepared as described [119] and resequencing performed at BGI Americas (San Jose, CA). We inspected BE22 sequence-specific variants for genic changes on chromosome II relative to N2 wild-type. Sanger sequencing of alleles of genetically defined collagens used standard procedures. To edit the *sc22* missense change in *col-71* we used the melting method [32]. We injected N2 animals with 2 µM *col-71* crRNA and 4 µM of repair template along with 50 ng/µl P*inx-6*-RFP co-injection marker and Cas9 (UC Berkeley MacroLab). F_1_ progeny were picked based on the co-injection marker and the *ju2038* SNP mutation verified by sequencing. Positive edits were retrieved as heterozygotes; we picked Rol progeny and verified the mutation again by sequencing.

### Imaging

We assessed overall strain brightness and localization using Leica MZ FLIII or M165 fluorescence dissection microscopes. For widefield and confocal imaging, we immobilized animals using 10 mM levamisole (Sigma) and imaged them on a Zeiss Axio Imager M2, a Zeiss LSM800 confocal, or a Zeiss LSM900 confocal with Airyscan capability, as described [14]. We varied laser power depending on the brightness of the specific knock-in strain. For the brightest strains, we reduced intensity by cutting the emission window. Confocal z stacks were generally made from 5–7 planes 0.3 µm apart to image the entire adult cuticle thickness. For imaging of the fibrous layers, we used z sections 0.17 µm apart. Airyscan speeds varied from 7 (fast) to 5 (slow); samples were first imaged using the fast setting, then more slowly for higher resolution as needed. Unless stated, animals were staged in mid L4 based on vulval morphology, then imaged 24 h later; images in Figures are representative of at least 10 animals imaged per strain. FRAP experiments were performed as described [14].

To measure the angles of fibers in the fibrous layer arrays with respect to the body axis, we examined maximum intensity projections of the midbody in day 1 adults. Angles were measured manually using line scans in Fiji at multiple points spaced 10 µm apart along the alae ridges (laterodorsal (LD) and lateroventral (LV)), and at multiple points 10 and 20 µm further dorsal or ventral (dorsolateral (DL) and dorsal (D); ventrolateral (VL) and ventral(V)).

Localization in L4 or adult males was generally analyzed in mNG/+ heterozygous cross progeny from crosses of N2 males with mNG/mNG hermaphrodites. For quantitation of knock-in expression intensity in heterozygous animals, we first crossed N2 wild-type males with mNG/mNG knock-in homozygotes, then crossed mNG/+ heterozygous F_1_ males with N2 hermaphrodites. Fluorescence intensity was measured in at least 3 ROIs in the midbody lateral cuticle of F_2_ animals 24 h post mid L4 stage. Background was subtracted and data normalized such that the homozygous strain mean = 1.

To image embryonic expression and localization, 100–150 embryos were picked from plates and mounted together on a 2% agarose pad in a droplet of 5–10 µl of M9 + 0.05% gelatin. Embryos were imaged on a Zeiss Axio Imager M2. We varied light exposure time depending on the brightness of the specific knock-in strain. A minimum of 20 embryos were observed for each developmental stage and knock-in strain.

### Quantitation and statistical analysis

Statistical analyses were performed using GraphPad Prism 10. Data were tested for normality, and parametric or nonparametric tests were used as appropriate. Statistical significance was determined using Fisher’s exact test, unpaired *t* test or Mann–Whitney test. For multiple comparisons, we used one-way ANOVA or Kruskal-Wallis tests followed by a post test. Data are represented as mean ± SEM unless noted. Sample sizes are shown inside or above columns in Figures or legends.

## Supporting information

S1 File Cuticle collagen sequences with annotations. (DOCX)

S1 Table Unsuccessful CRISPR edits. (XLSX)

S2 Table Heatmap of expression patterns. (XLSX)

S3 Table Kunitz inhibitor domain containing genes. (XLSX)

S4 Table Strains, new alleles, and genotypes. (XLSX)

S5 Table Plasmids. (XLSX)

S6 Table Oligonucleotides. (XLSX)

S7 Table CRISPR reagents. (XLSX)

S1 Fig Optimization of knock-in editing parameters. (A–D) Editing optimization using GFP::GLH-1 knock-ins as a test case. (A) Cas9 from the indicated sources at the indicated amounts with a 2.5:1 RNA:protein ratio. IDT Cas9 at 250 ng/µl was most efficient, similar to previous reports [31]. (B) The indicated RNA:protein molar ratios (crRNA and trcRNA at equal concentration, RNA amount varied to 250 ng/µl Cas9). (C and D) The indicated repair templates with 250 ng/µl Cas9, 1:1 RNA:protein ratio). (E) Number of GFP::GLH-1 knock-ins found in animals lacking (-) or containing (+) a *mlc-1p::NLS::tagRFP* co-injection marker. (F) Percent of *mlc-1p::NLS::tagRFP* co-injection marker positive animals laid on day and day 2 post-injection with *myo-2::T2A::GFP* knock-ins. (G) Number of *mlc-1p::NLS::tagRFP* co-injection marker positive animals laid on day and day 2 post-injection with *myo-2::T2A::GFP* knock-ins. (H) Number of GFP::GLH-1 knock-ins found in animals lacking (−) or containing (+) a *myo-2::T2A::GFP* co-CRISPR knock-in. Statistical significance was determined using a two-tailed unpaired Student *t* test. *P* < 0.05 was considered statistically significant. * Indicates *P* < 0.05, ** indicates *P* < 0.005. Two independent experiments were performed for all optimization tests. Each dot in the graphs A–D and F–H represents the parameter scored on the Y-axis in the F1 of a singled, injected P0 animal and the line indicates the mean. See S2 Data for the data underlying the graphs.
(PDF)

S2 Fig An *unc-119(+)-*selection based modular mNeonGreen::3xFLAG knock-in pipeline. (A) A schematic of the knock-in cassette with the *C. briggsae unc-119(+)* rescue cassette being transcribed from the opposite strand as the mNeonGreen::3xFLAG cassette. The selection cassette is flanked with loxP sites and is within an intron. Knock-ins rescue the uncoordinated phenotype of *unc-119* mutants and the cassette can be excised through Cre expression. (B) Strains generated with the *unc-119(+)* knock-in pipeline. Our RNP-based pipeline was most efficient with genes expressed in larger tissues (hyp7 syncytium, seam cells, pharyngeal cells) and at high levels (>1,000 transcripts per million in a single cell L2 stage RNA-seq dataset [95] as these criteria allowed facile screening under a fluorescence dissecting microscope. *unc-119(+)* selection is more suitable for high-throughput pipelines than alternatives such as self-excising cassettes, as the 60 bp homology arms can be made using oligo annealing, bypassing need for PCR. We developed a robust assembly pipeline and successfully tagged two collagens (*col-75* and *col-97*) and one warthog gene (*wrt-2)* as proof-of-principle. We chose these test cases as: i) *col-75* is predicted to be restricted to socket cells and excretory duct or pore cells at moderate levels; ii) an RNP-based attempt at *col-97* tagging had failed; and iii) *wrt-2* was expressed at moderate levels.
(PDF)

S3 Fig A SapTrap assembly pipeline for *unc-119(+)-*selection based modular mNeonGreen::3xFLAG repair templates. (A) Oligos encoding the target sgRNA, the 5′ and 3′ homology arms (HA) are annealed to produce the indicated 3 bp overhangs and combined with the pJW2476 vector containing the modular mNeonGreen::3xFLAG *unc-119(+)* cassette and an appropriate backbone (such as pDD379) in a SapTrap cloning reaction. (B) lists the settings used in SapTrap Builder. We set cloning for SapTrap, output as oligos + annotated, and 60 nt oligos for the upstream (hUS) and downstream (hDS) homology arms. (C) is a representative output for tagging *col-75.* The red box indicates the change to the output needed for a custom 3 nt overhang needed to use the pJW2476 cassette. For the hUS-B oligo the 5′-CGC-3′ at the 5′ end needs to be replaced with “5′-GGA-3′
(PDF)

S4 Fig aECM markers in non-epidermal tissues. Representative examples of mNG knock-in localization in nonepidermal tissues. (A) Pharynx (KUND-7 (K10D3.4)::mNG). Widefield imaging, embryo to L4 stages. Nontransgenic control N2 is shown above (B) Gut (CTSA-1.1::mNG). Widefield and Airyscan confocal images. Expression in the gut/ intestine is marked with yellow dashed lines; expression in the coelomocytes is marked with red arrows; expression in the muscles is marked by blue arrows. (C) Coelomocytes (CTSA-1.1::mNG). Confocal images of anterior, midbody and posterior coelomocytes. (D) Somatic gonad (SKPO-1::mNG). Confocal images of anterior and posterior gonad arms. Scales, 10 µm.
(PDF)

S5 Fig Stage specificity of collagen knock-in expression. (A) DPY-4::mNG, (B) DPY-5::mNG, and (C) DPY-13::mNG are not detectable in late embryos; fluorescence was first detectable in late L1 and remains visible through L4 stage. Widefield DIC and fluorescence. Autofluorescent gut granules are visible in the DPY-5::mNG L1 (yellow arrows). Scales, 10 µm.
(PDF)

S6 Fig Knock-in dosage and mobility of fusion proteins. (A) mNG fluorescence intensities in the midbody cuticle from homozygous and heterozygous strains, dot plots of intensities normalized to controls (see Materials and methods). Intensities of heterozygous strains were doubled to correct for copy number and compared to the homozygous strain intensity by Mann–Whitney test; *** *P* < 0.001. See S3 Data for the data underlying the graphs. (B) Fluorescence Recovery After Photobleaching (FRAP) of selected KIs in the body and vulval cuticles. Most mNG fusion proteins did not show significant recovery, apart from LON-8::mNG in the vulval cuticle. See S4 Data for the data underlying the graphs.
(PDF)

S7 Fig Furrow and strut localization. Single Airyscan focal planes showing dual localization to furrow and struts. COL-81::mNG localized to the midline of each furrow in cortical planes (S6) and to strut puncta in basal planes (S9). COL-103::mNG localized to furrow-flanking stripes in cortical planes (S6-8) and to strut-like puncta in basal planes (S9). Scales, 5 µm.
(PDF)

S8 Fig Strut localization. Single Airyscan focal planes of strut-related localizations. COL-98::mNG showed diffuse localization in more cortical planes (S9-10) and punctate strut localization in medial planes (S11). COL-93::mNG showed ring-like localization in multiple focal planes. Scales, 5 µm.
(PDF)

S9 Fig COL-80::mNG localization. (A) COL-80::mNG localization in adults (L4 + 24 h). COL-80::mNG localized to furrows and annuli in the L4 stage; additional strut-like puncta developed in adults. (B) In *bli-1* mutants COL-80::mNG failed to localize to struts and remained in furrows and annular ridges on the cortical surface of blisters. Scales, 10 µm.
(PDF)

S10 Fig Crossed fibrous array collagens and localization within the fibrous layer. (A) Airyscan single focal plane images of COL-38::mNG and COL-138::mNG showing crossed fiber arrays in young adults. Scale, 10 µm. (B) Airyscan z-stacks (11 x 0.17 µm) and single focal planes of COL-20::mNG showing localization to outer (right-handed) oriented fibrous layer and basal fibrillar layer. Stacks from anterior body, midbody, and posterior body. Scales, 5 µm. Cartoons of COL-20::mNG localization in different focal planes.
(PDF)

S11 Fig Collagen localization in blistered mutant adults. Confocal images of collagen KIs in Bli mutant adults. Lateral views of head or midbody cuticle; anterior to the left and dorsal up. Blister cortical surfaces outlined with pink dashed lines, basal surfaces outlined by red dashed lines. (A) LON-3::mNG, DPY-13::mNG and ROL-8::mNG were basal to blisters. (B) COL-63::mNG, COL-125::mNG, and COL-130::mNG were cortical to blisters. (C) COL-20::mNG was primarily basal with faint localization to cortical surface. Patterning of the markers was otherwise unaffected in the blistered background. Scales, 10 µm.
(PDF)

S12 Fig Double label imaging of collagens. (A) DPY-5::mSc DPY-9::mNG double label knock-in strain. (B) DPY-5::mSc DPY-10::mNG double label. (C) DPY-10::mSc ROL-6::mNG double label. (D) ROL-6::mNG; DPY-5::mSc double label. Images are from L4 or day 1 adult as indicated. Left, single focal planes (0.2 µm slices) from cortical to basal. Scales 10 µm. Right, Magnified xz slices generated using Zeiss Zen 3D projection tool; *z* axis is exaggerated to illustrate differences in z plane. Scale in *x* axis, 2 µm.
(PDF)

S13 Fig Localization patterns in adult lateral alae and in dauer stage larvae. (A) Selected examples of adult alae localization patterns, showing localization to alae ridges (yellow arrows), ridge flanks (red lines), and valleys. Confocal Airyscan projections; scale, 5 µm. (B) L1 alae localization of DPY-14::mNG (yellow arrow) compared to exclusion from alae of DPY-9::mNG. (C) Dauer alae localization of COL-43::mNG, COL-103::mNG, COL-48::mNG, and LON-8::mNG. Scales, 10 µm.
(PDF)

S14 Fig Localization of the atypical collagen COL-135:mNG. Images of anterior spermatheca (left), uterus/eggs (middle), and posterior spermatheca (left) in L4 and young adult stages. (A) COL-135::mNG was expressed in spermathecal cells beginning in late L4 stage (L4.7 shown). (B) In young adults COL-135::mNG localized to puncta in spermathecal cells and/or the spermathecal lumen, and to uterine matrix surrounding newly fertilized eggs. COL-135::mNG was often deposited on eggs in extended folds or furrows on the egg surface (yellow arrows) and appeared to be retained on the eggshell after egg laying. Images are maximum intensity projections of z stacks of 23–49 x 0.5 µm slices. (C) Fluorescence and brightfield images of spermathecal and uterine expression in young adults. Scales, 10 µm.
(PDF)

S1 Data Numerical data for the graph in Fig 6B. (XLSX)

S2 Data Numerical data for the graphs in S1 Fig. (XLSX)

S3 Data Numerical data for the graph in S6A Fig. (XLSX)

S4 Data Numerical data for the graph in S6B Fig. (XLSX)

## Abbreviations

aECMs: apical extracellular matrices
BMs: basement membranes
CGC: Caenorhabditis Genetics Center
Cys: cysteine-rich domains
ECM: Extracellular matrix
EES: extra embryonic space
FRAP: Fluorescence Recovery After Photobleaching
HA: homology arms
hDS: and downstream
mNG: mNeonGreen
mSc: mScarlet
RAM: ray abnormal morphology
RNP: ribonucleoprotein complex
S6: planes
WGS: whole–genome sequencing
ZP: zona pellucida.
