# Supplementary material for: A systematic fluorescently tagged protein toolkit for dissecting apical extracellular matrix patterning in C. elegans
Source: PLoS Biol. 2026 Oct 14;24(10):e3004059. doi: 10.1371/journal.pbio.3004059 (PMC13705611; doi:10.1371/journal.pbio.3004059)
Supplement: Supplementary file 1 [file pbio.3004059.s009.pdf]

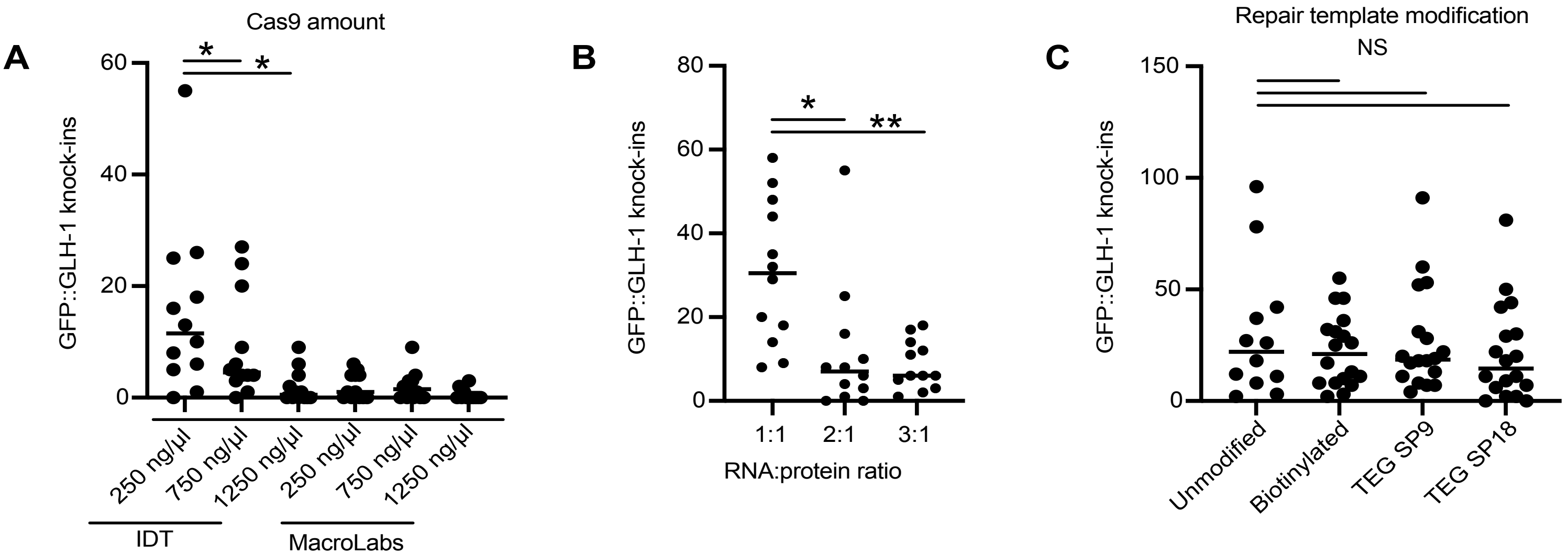

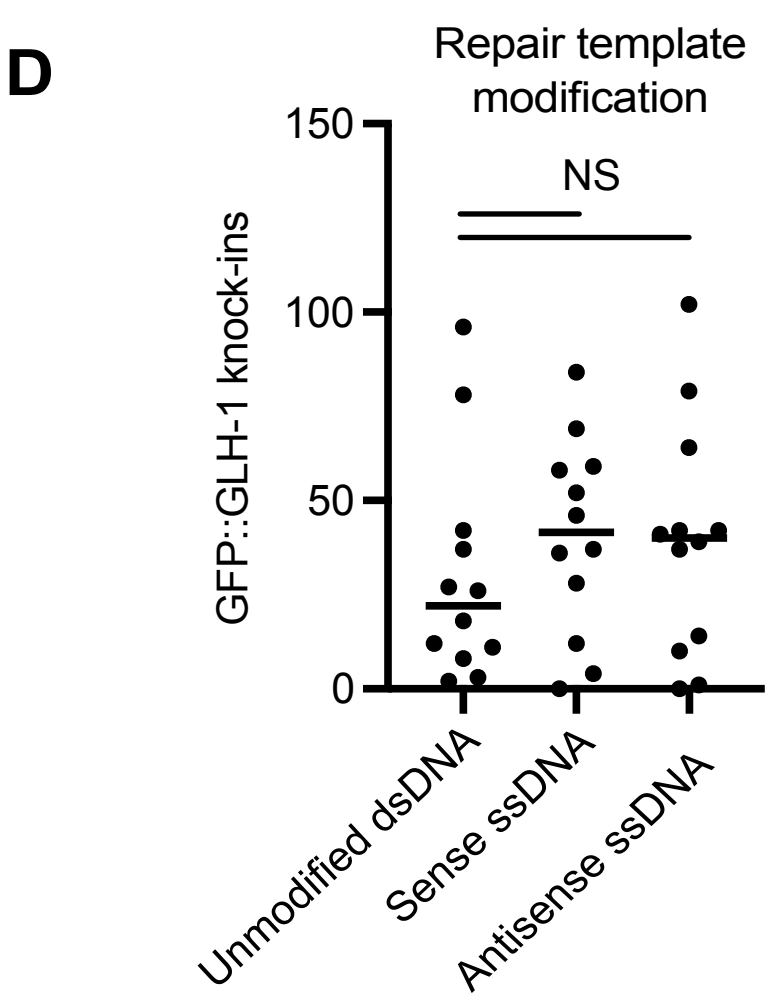

E

mlc-1p::NLS::tagRFP	% GLH-1::GFP positive animals	N
-	2.4	367
+	26	400

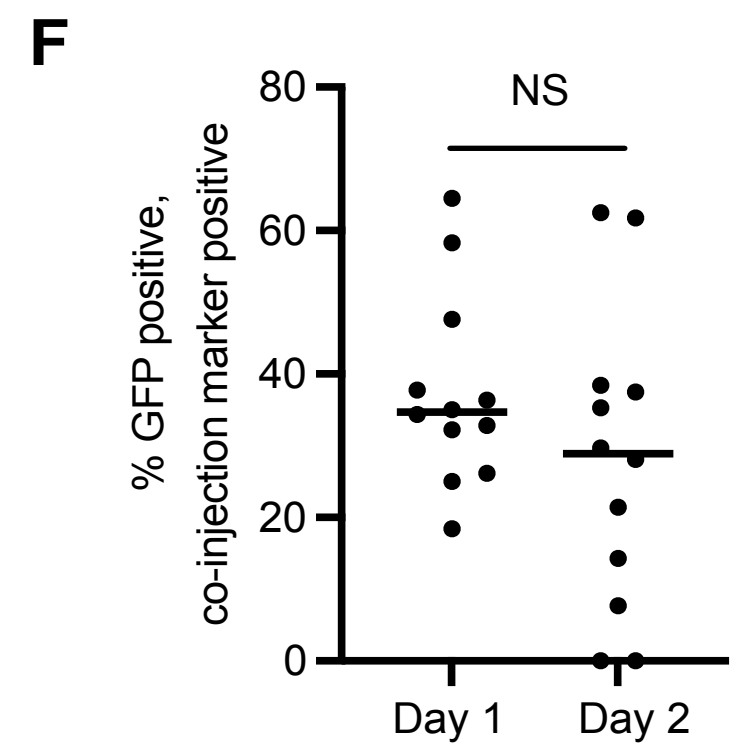

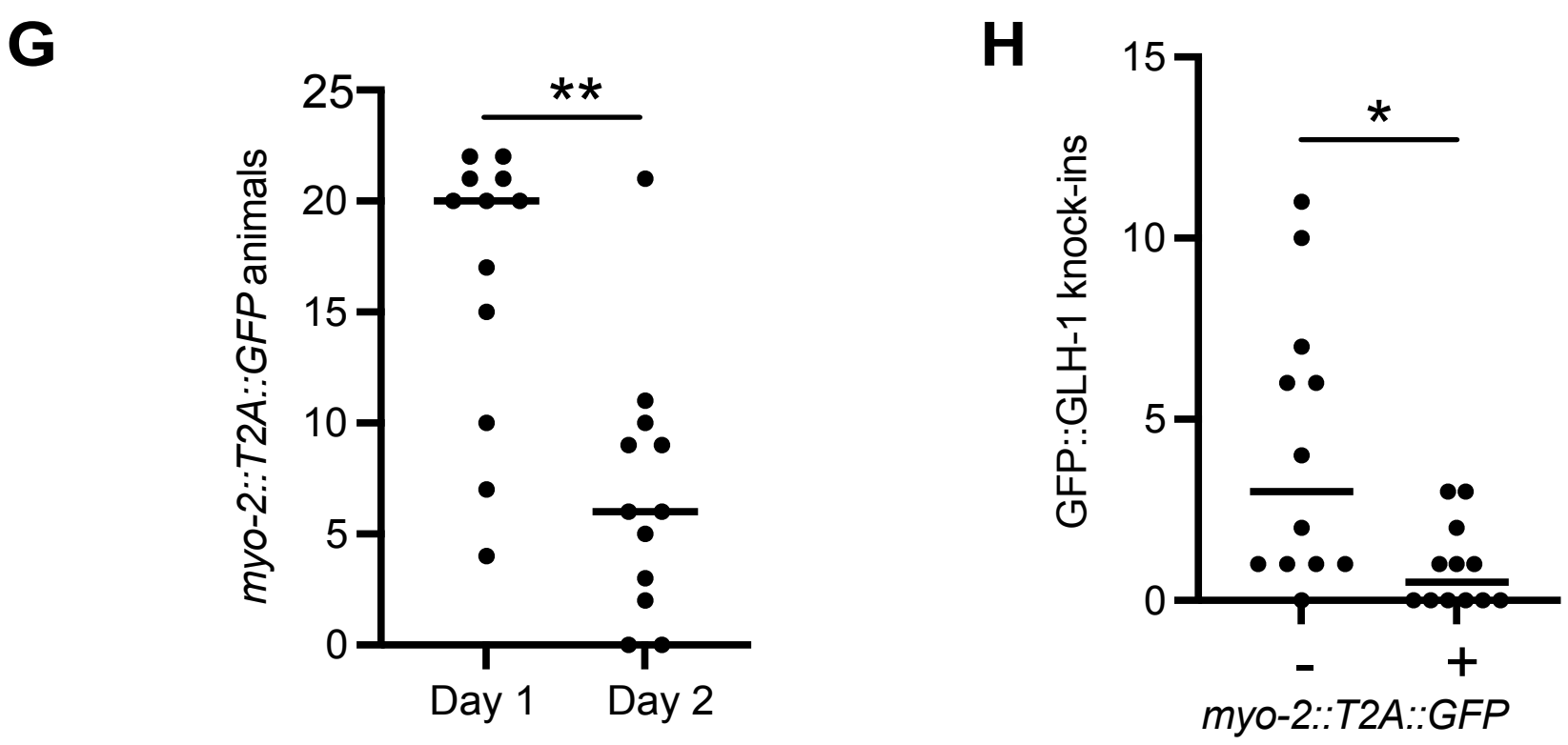

Figure S1
