# Supplementary material for: A systematic fluorescently tagged protein toolkit for dissecting apical extracellular matrix patterning in C. elegans
Source: PLoS Biol. 2026 Oct 14;24(10):e3004059. doi: 10.1371/journal.pbio.3004059 (PMC13705611; doi:10.1371/journal.pbio.3004059)
Supplement: Supplementary file 2 [file pbio.3004059.s010.pdf]

A

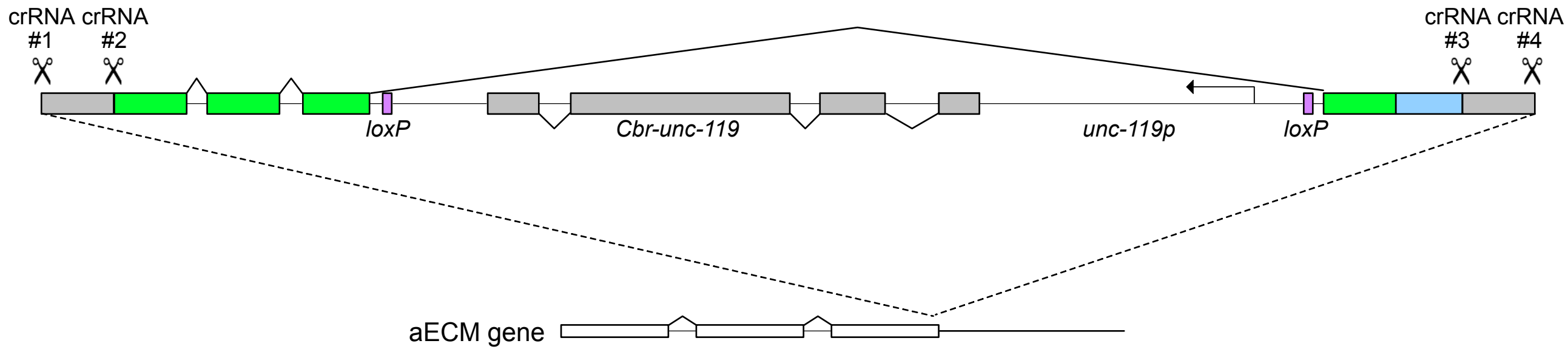

Inject repair template+sgRNA vector into *unc-119* temperature-sensitive strain or null. Either include Cas9 plasmid in injection mix or have a stable Cas9 transgene in the injected *unc-119* mutant. Isolate insertions and homozygose knock-in.

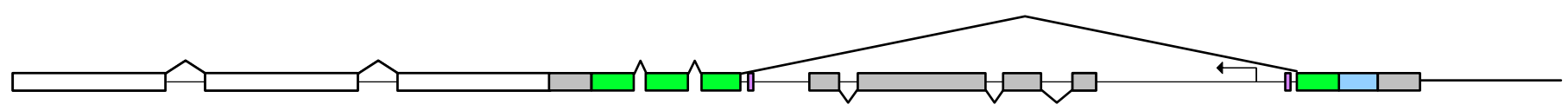

Cre expresion. Some strains carrying a stable Cas9 transgene also have a *hsp-16.41p::Cre* transgene in the insertion and the cassette can be excised through heat-shock. Otherwise injection of a Cre plasmid is required. The *unc-119* mutation (and Cas9 transgene if used) then need to be removed by outcrossing.

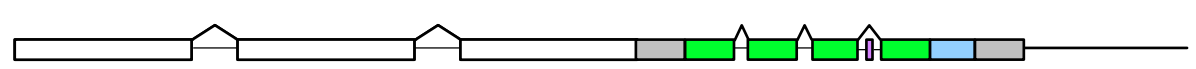

B

**Strain**	**Genotype**
JDW850	*col-75(wrd349[col-75::mNG::3xFLAG]) II*
JDW834	*col-97(wrd346[col-97 internal C-term modular mNG::3xFLAG (unc-119+)]) ; unc-119(kst33) III*
JDW877	*wrt-2(wrd365[internal after signal sequence mod mNG::3xFLAG]) X*

Figure S2
