# Supplementary material for: A systematic fluorescently tagged protein toolkit for dissecting apical extracellular matrix patterning in C. elegans
Source: PLoS Biol. 2026 Oct 14;24(10):e3004059. doi: 10.1371/journal.pbio.3004059 (PMC13705611; doi:10.1371/journal.pbio.3004059)
Supplement: Supplementary file 3 [file pbio.3004059.s011.pdf]

A

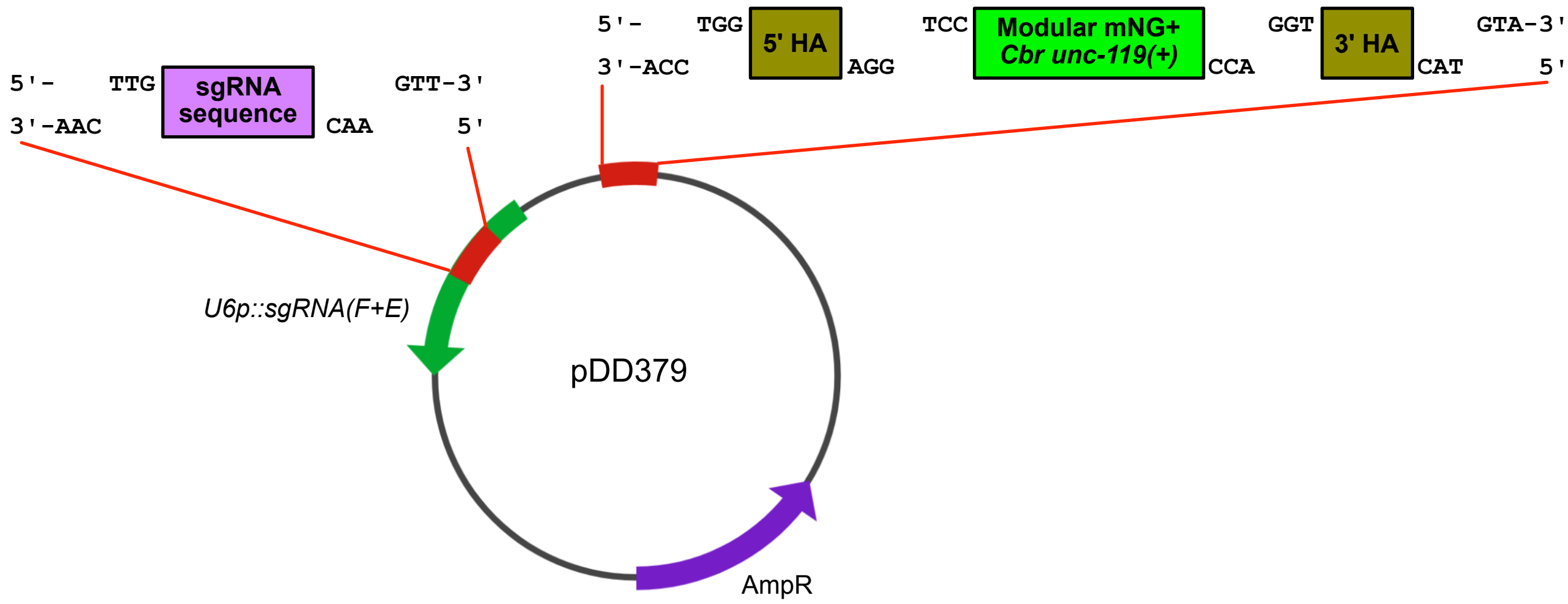

B

**Type**	SapTrap
**Output**	Oligos +Annotated
**hUS target length**	60
**hDS target length**	60

C

**col-75hUS_T**	TGGCCCTGTCCATCTCGGAAAATGTTCTTATCAATTTAAGAGAAAAAACCAAGAAACAATTG
**col-75hUS_B**	CGCCAATTGTTTCTTGGTTTTTTCTCTTAAATTGATAAGAACATTTTCCGAGATGGACAGGG
**col-75hDS_T**	GGTTAAaacaaacagatcatacatgtatttttaaattgaaattttccatatttacaattgtt
**col-75hDS_B**	TACaacaattgtaaatatggaaatttcaatttaaaaaatacatgtatgatctgtttgttTTA
**col-75sgRNA_T**	TTGtttgttTTACAATTGTTTCT
**col-75sgRNA_B**	AACAGAAACAATTGTAAaacaaa

Need to change CGC at 5' end of hUS_B to GGA if using pJW2476 modular mNeonGreen+*Cbr unc-119(+)*

Figure S3
