# Supplementary material for: A systematic fluorescently tagged protein toolkit for dissecting apical extracellular matrix patterning in C. elegans
Source: PLoS Biol. 2026 Oct 14;24(10):e3004059. doi: 10.1371/journal.pbio.3004059 (PMC13705611; doi:10.1371/journal.pbio.3004059)
Supplement: Supplementary file 4 [file pbio.3004059.s012.pdf]

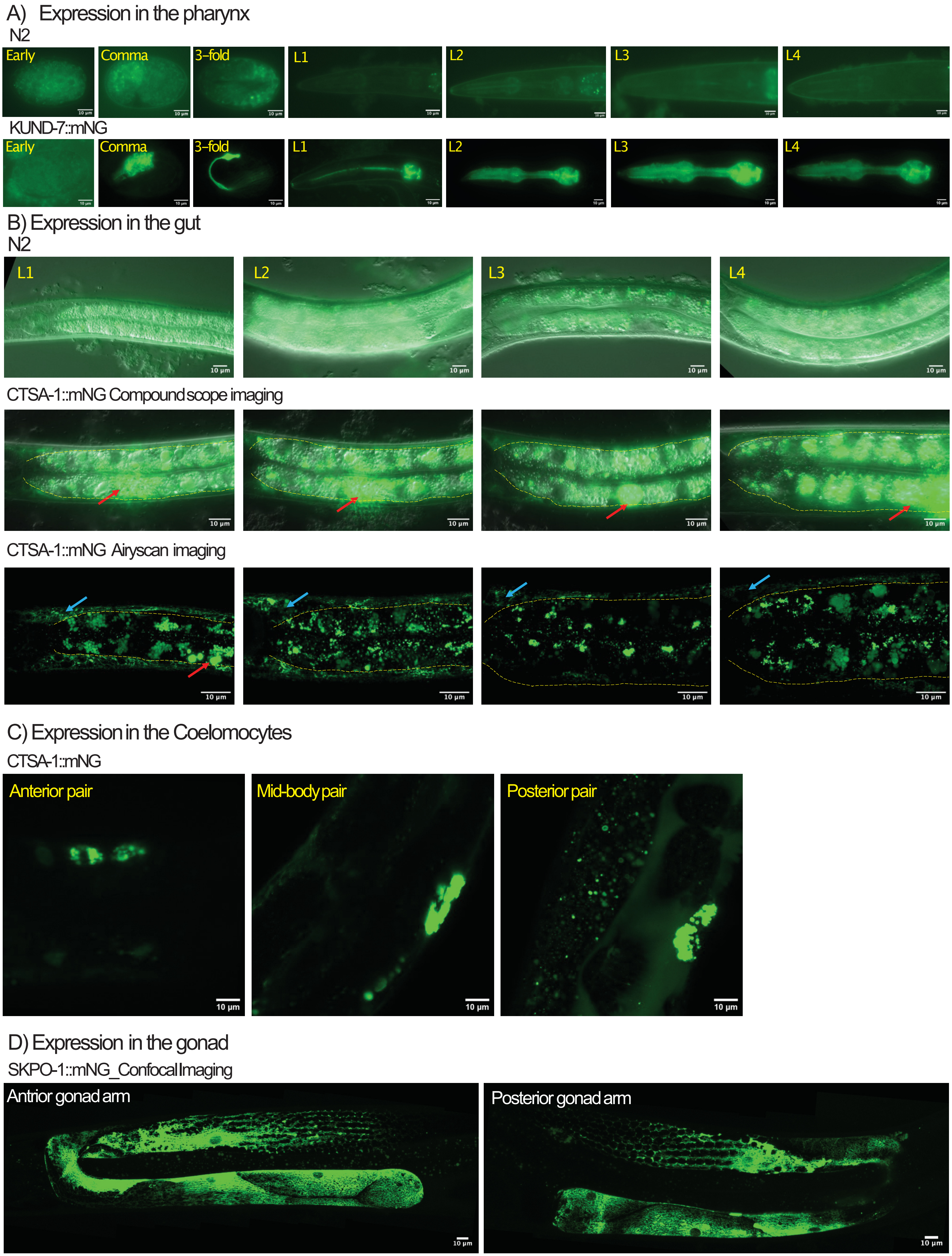
A) Expression in the pharynx
N2
Early
Comma
3-fold
L1
L2
L3
L4
KUND-7::mNG
Early
Comma
3-fold
L1
L2
L3
L4
B) Expression in the gut
N2
L1
L2
L3
L4
10 μm
CTSA-1::mNG Compound scope imaging
CTSA-1::mNG Airyscan imaging
C) Expression in the Coelomocytes
CTSA-1::mNG
Anterior pair
Mid-body pair
Posterior pair
D) Expression in the gonad
SKPO-1::mNG_Confocal Imaging
Antrior gonad arm
Posterior gonad arm

Figure S4
