# Supplementary material for: A systematic fluorescently tagged protein toolkit for dissecting apical extracellular matrix patterning in C. elegans
Source: PLoS Biol. 2026 Oct 14;24(10):e3004059. doi: 10.1371/journal.pbio.3004059 (PMC13705611; doi:10.1371/journal.pbio.3004059)
Supplement: Supplementary file 5 [file pbio.3004059.s013.pdf]

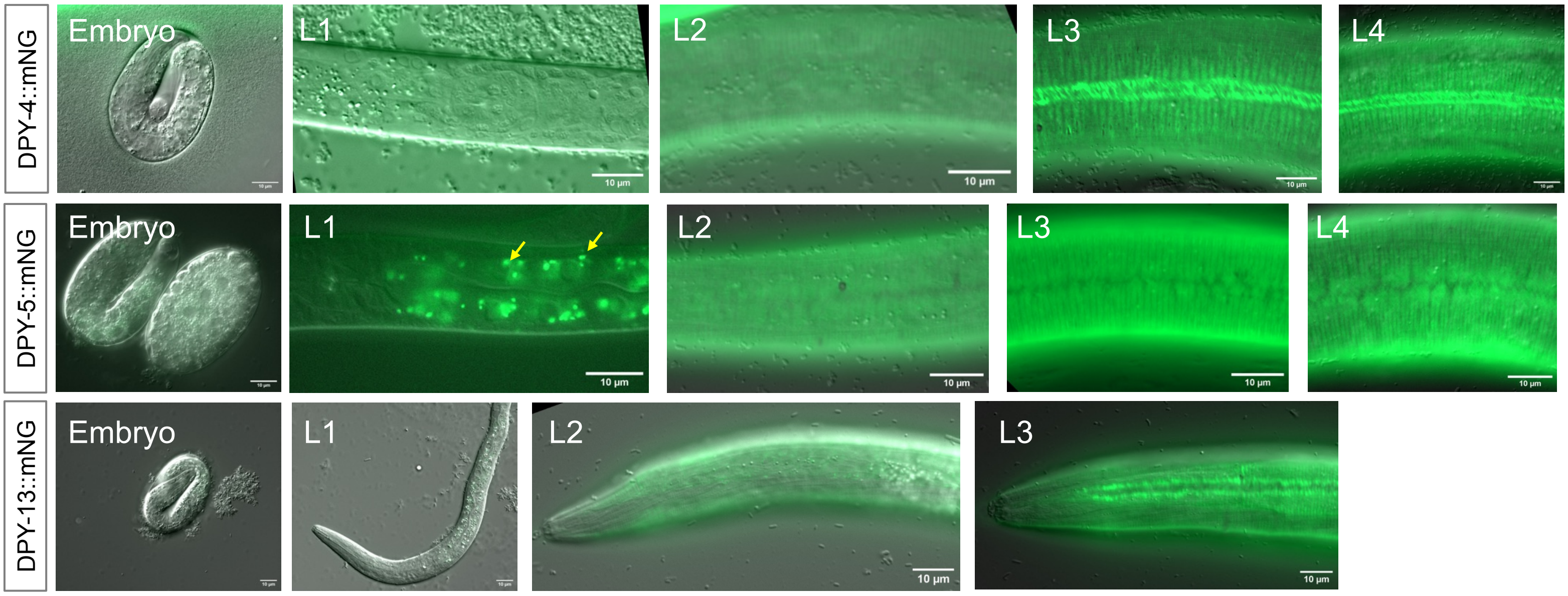
DPY-4::mNG
Embryo
L1
L2
L3
L4
DPY-5::mNG
Embryo
L1
L2
L3
L4
DPY-13::mNG
Embryo
L1
L2
L3
10 µm

Figure S5
