# Supplementary material for: A systematic fluorescently tagged protein toolkit for dissecting apical extracellular matrix patterning in C. elegans
Source: PLoS Biol. 2026 Oct 14;24(10):e3004059. doi: 10.1371/journal.pbio.3004059 (PMC13705611; doi:10.1371/journal.pbio.3004059)
Supplement: Supplementary file 6 [file pbio.3004059.s014.pdf]

A

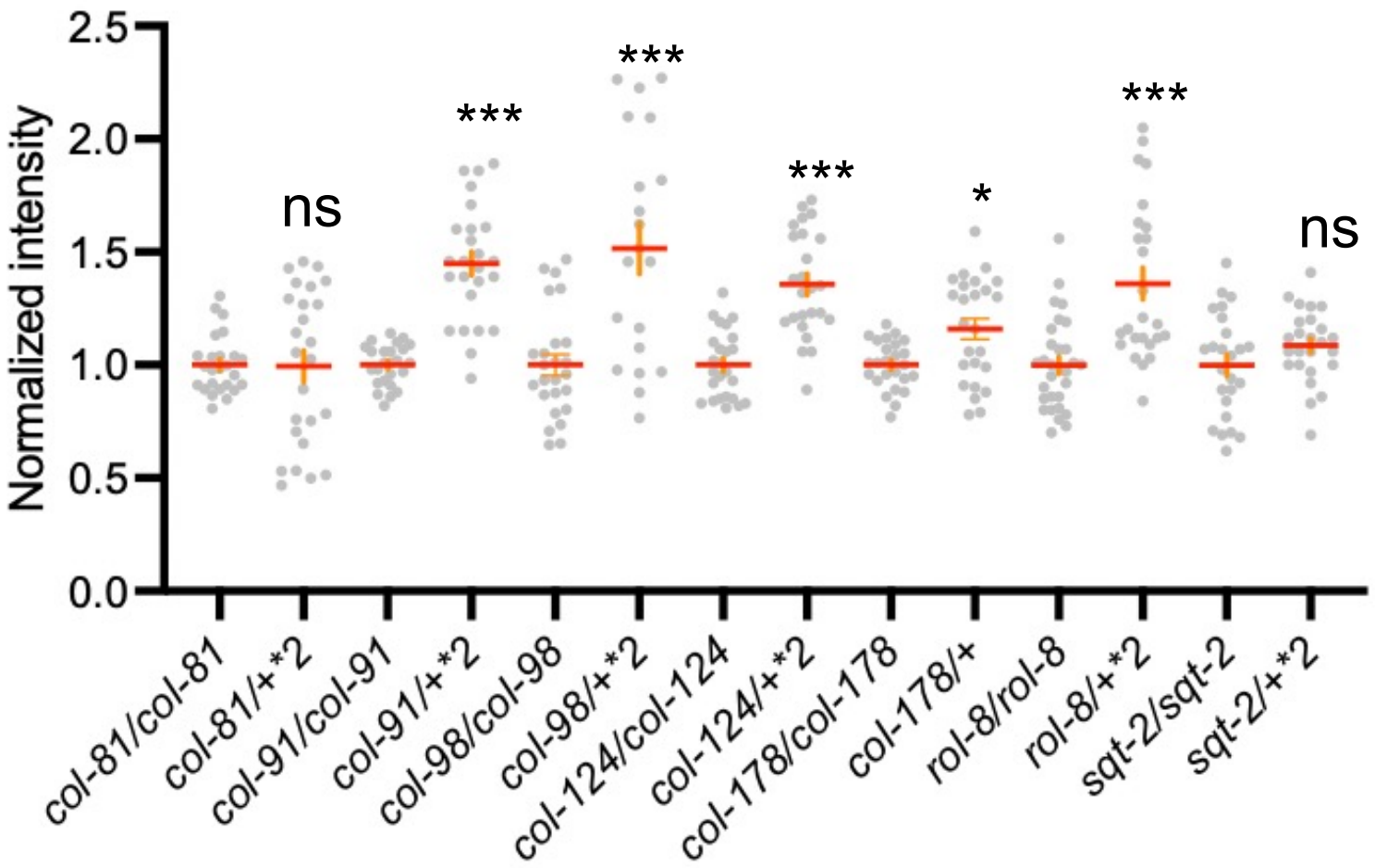
Normalized intensity
2.5
2.0
1.5
1.0
0.5
0.0
ns
***
***
***
*
***
ns
col-81/col-81
col-81/+*2
col-91/col-91
col-91/+*2
col-98/col-98
col-98/+*2
col-124/col-124
col-124/+*2
col-178/col-178
col-178/+
rol-8/rol-8
rol-8/+*2
sqt-2/sqt-2
sqt-2/+*2

B

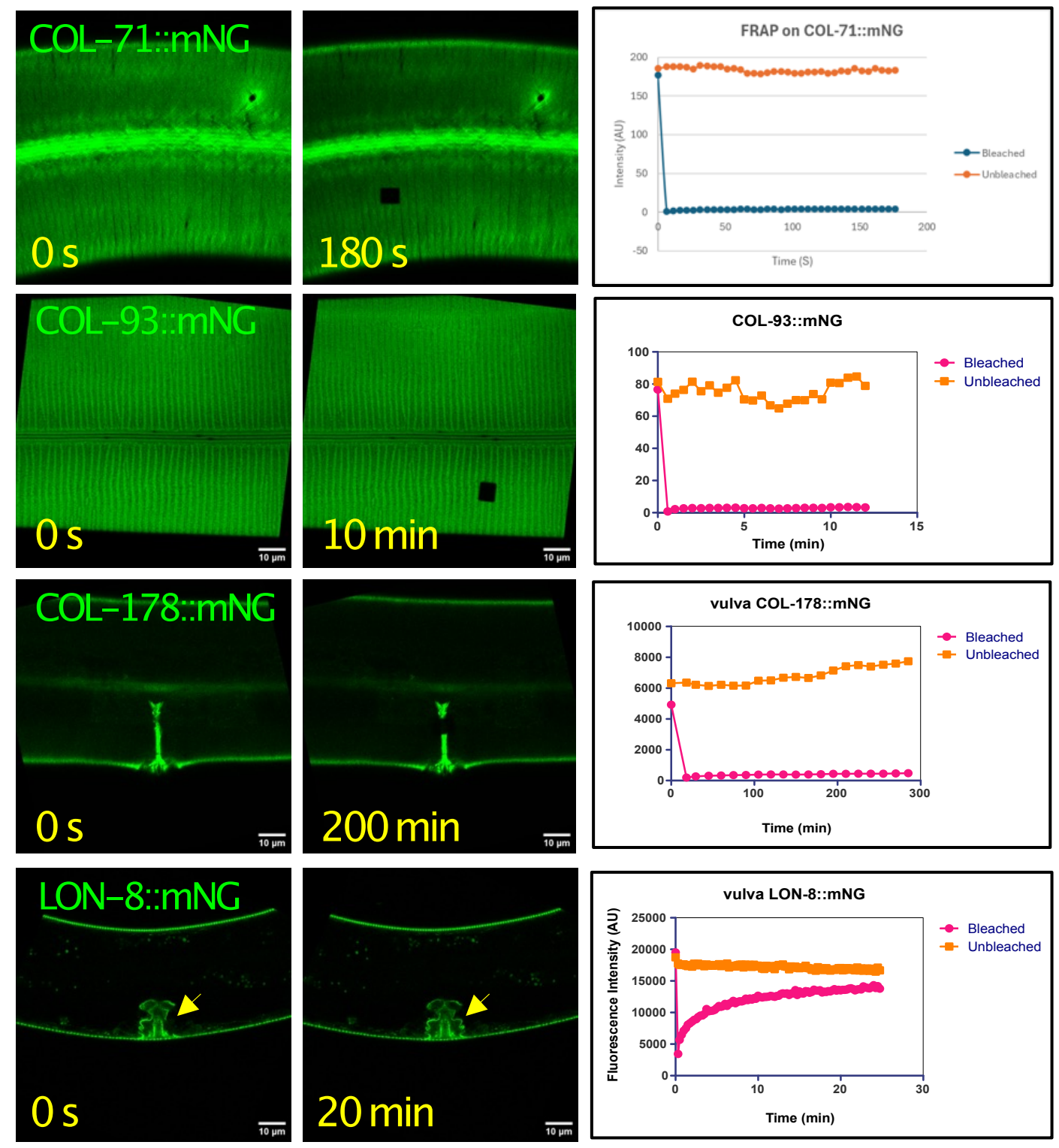
COL-71::mNG
0 s
180 s
FRAP on COL-71::mNG
Bleached
Unbleached
COL-93::mNG
0 s
10 min
COL-93::mNG
Time (min)
COL-178::mNG
0 s
200 min
vulva COL-178::mNG
Time (min)
LON-8::mNG
0 s
20 min
vulva LON-8::mNG
Fluorescence Intensity (AU)
Time (min)

Figure S6
