# Supplementary material for: A systematic fluorescently tagged protein toolkit for dissecting apical extracellular matrix patterning in C. elegans
Source: PLoS Biol. 2026 Oct 14;24(10):e3004059. doi: 10.1371/journal.pbio.3004059 (PMC13705611; doi:10.1371/journal.pbio.3004059)
Supplement: Supplementary file 7 [file pbio.3004059.s015.pdf]

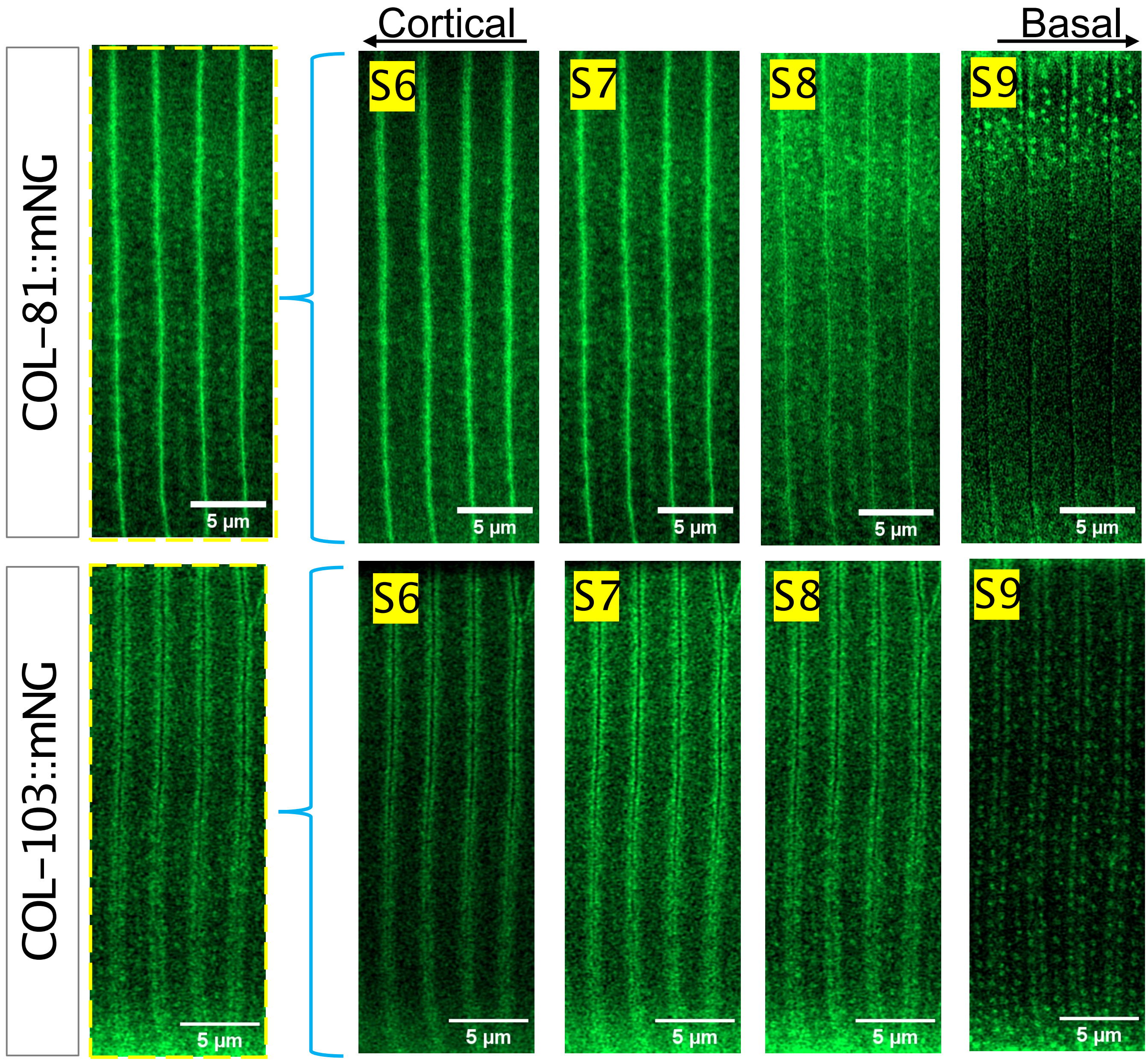
Cortical
Basal
COL-81::mNG
S6
S7
S8
S9
5 µm
COL-103::mNG
S6
S7
S8
S9
5 µm

Figure S7
