# Supplementary material for: A systematic fluorescently tagged protein toolkit for dissecting apical extracellular matrix patterning in C. elegans
Source: PLoS Biol. 2026 Oct 14;24(10):e3004059. doi: 10.1371/journal.pbio.3004059 (PMC13705611; doi:10.1371/journal.pbio.3004059)
Supplement: Supplementary file 8 [file pbio.3004059.s016.pdf]

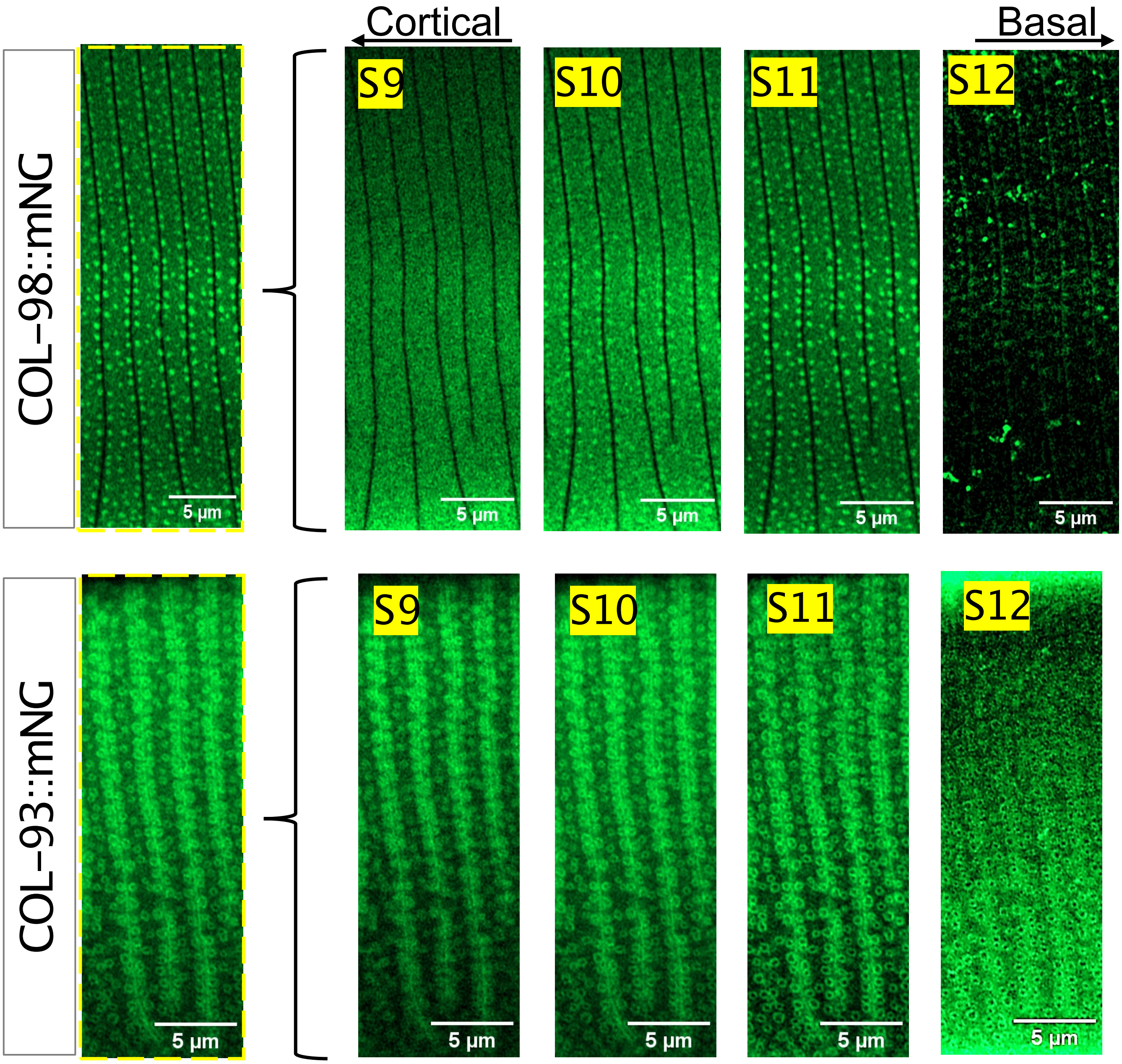

Cortical
Basal
COL-98::mNG
S9
S10
S11
S12
5 µm
COL-93::mNG
S9
S10
S11
S12
5 µm

Figure S8
