# Supplementary material for: A systematic fluorescently tagged protein toolkit for dissecting apical extracellular matrix patterning in C. elegans
Source: PLoS Biol. 2026 Oct 14;24(10):e3004059. doi: 10.1371/journal.pbio.3004059 (PMC13705611; doi:10.1371/journal.pbio.3004059)
Supplement: Supplementary file 9 [file pbio.3004059.s017.pdf]

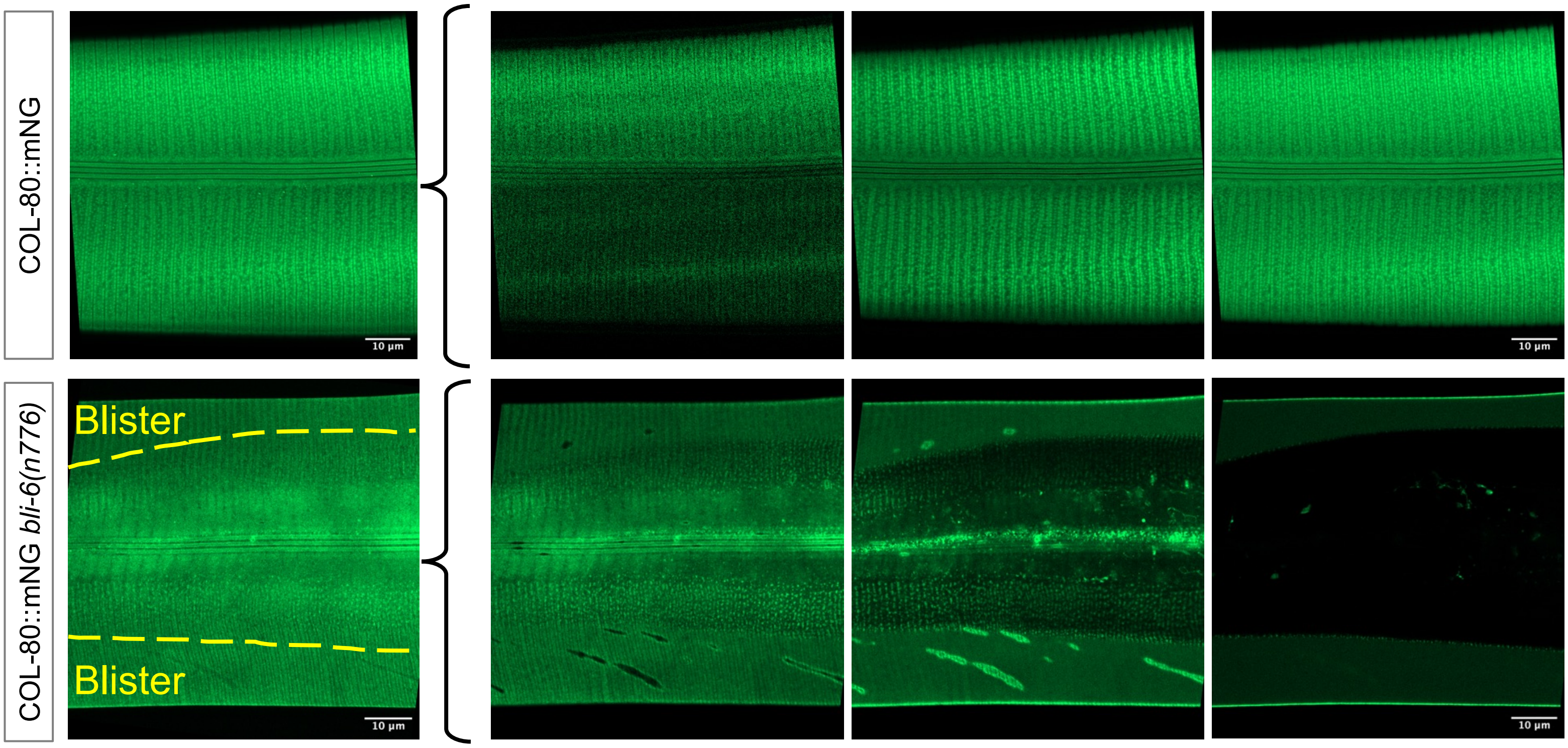

COL-80::mNG
COL-80::mNG bli-6(n776)
Blister
Blister
10 µm
10 µm
10 µm
10 µm

Figure S9
