# Supplementary material for: A systematic fluorescently tagged protein toolkit for dissecting apical extracellular matrix patterning in C. elegans
Source: PLoS Biol. 2026 Oct 14;24(10):e3004059. doi: 10.1371/journal.pbio.3004059 (PMC13705611; doi:10.1371/journal.pbio.3004059)
Supplement: Supplementary file 10 [file pbio.3004059.s018.pdf]

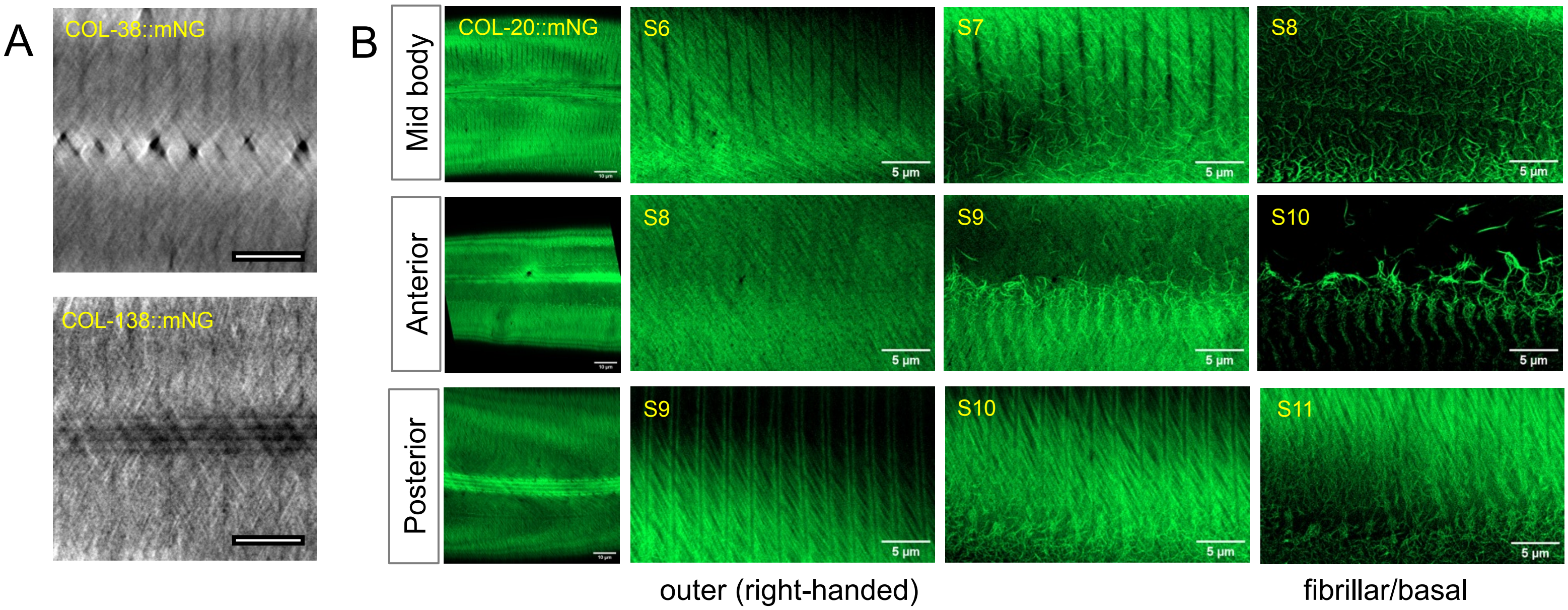

A
COL-38::mNG
COL-138::mNG
B
Mid body
Anterior
Posterior
COL-20::mNG
S6
S7
S8
S8
S9
S10
S9
S10
S11
5 µm
outer (right-handed)
fibrillar/basal

Figure S10
