# Supplementary material for: A systematic fluorescently tagged protein toolkit for dissecting apical extracellular matrix patterning in C. elegans
Source: PLoS Biol. 2026 Oct 14;24(10):e3004059. doi: 10.1371/journal.pbio.3004059 (PMC13705611; doi:10.1371/journal.pbio.3004059)
Supplement: Supplementary file 11 [file pbio.3004059.s019.pdf]

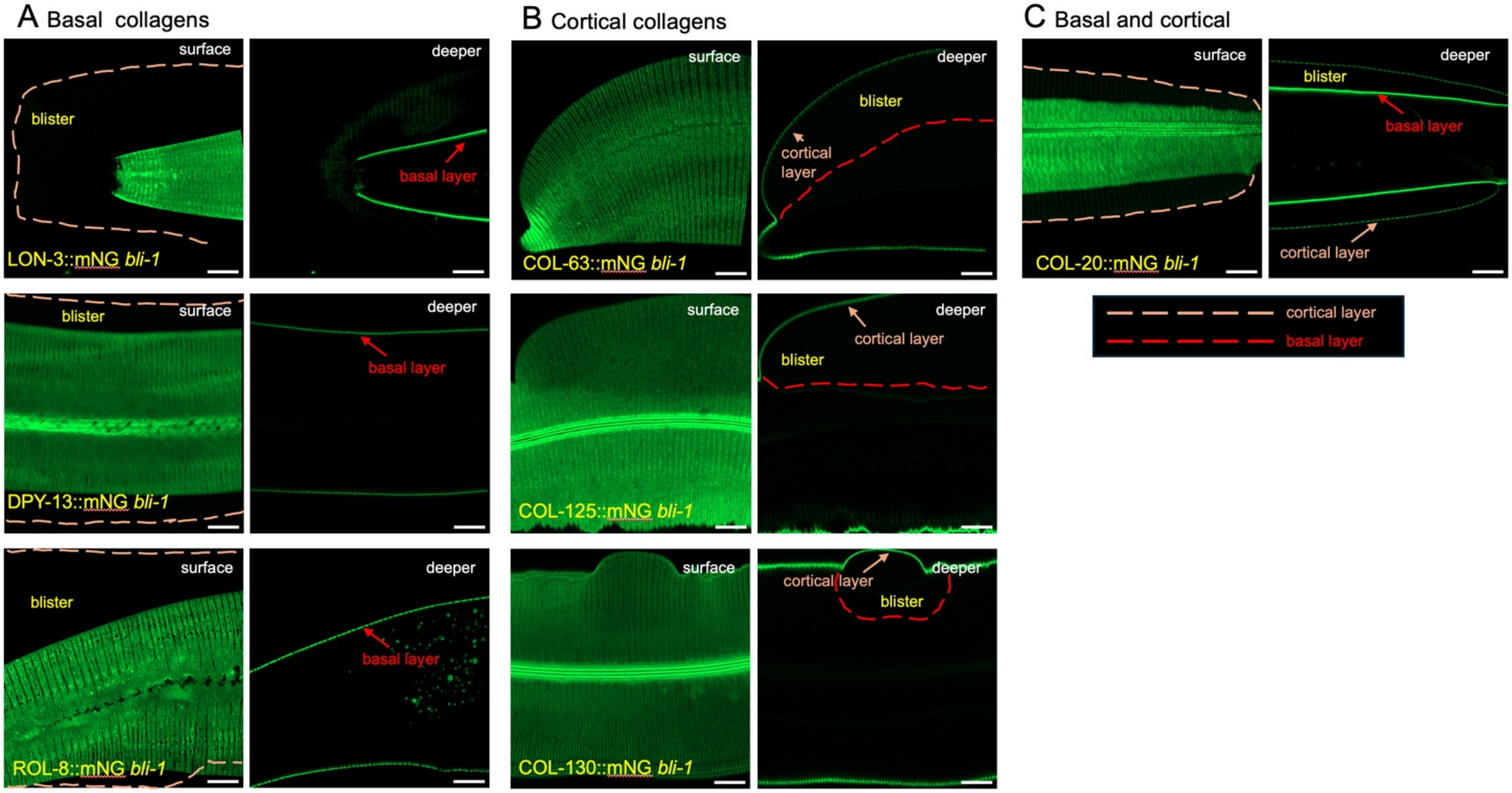
A Basal collagens
B Cortical collagens
C Basal and cortical
surface
deeper
blister
basal layer
LON-3::mNG bli-1
DPY-13::mNG bli-1
ROL-8::mNG bli-1
cortical layer
COL-63::mNG bli-1
COL-125::mNG bli-1
COL-130::mNG bli-1
COL-20::mNG bli-1
cortical layer
basal layer

Figure S11
