# Supplementary material for: A systematic fluorescently tagged protein toolkit for dissecting apical extracellular matrix patterning in C. elegans
Source: PLoS Biol. 2026 Oct 14;24(10):e3004059. doi: 10.1371/journal.pbio.3004059 (PMC13705611; doi:10.1371/journal.pbio.3004059)
Supplement: Supplementary file 12 [file pbio.3004059.s020.pdf]

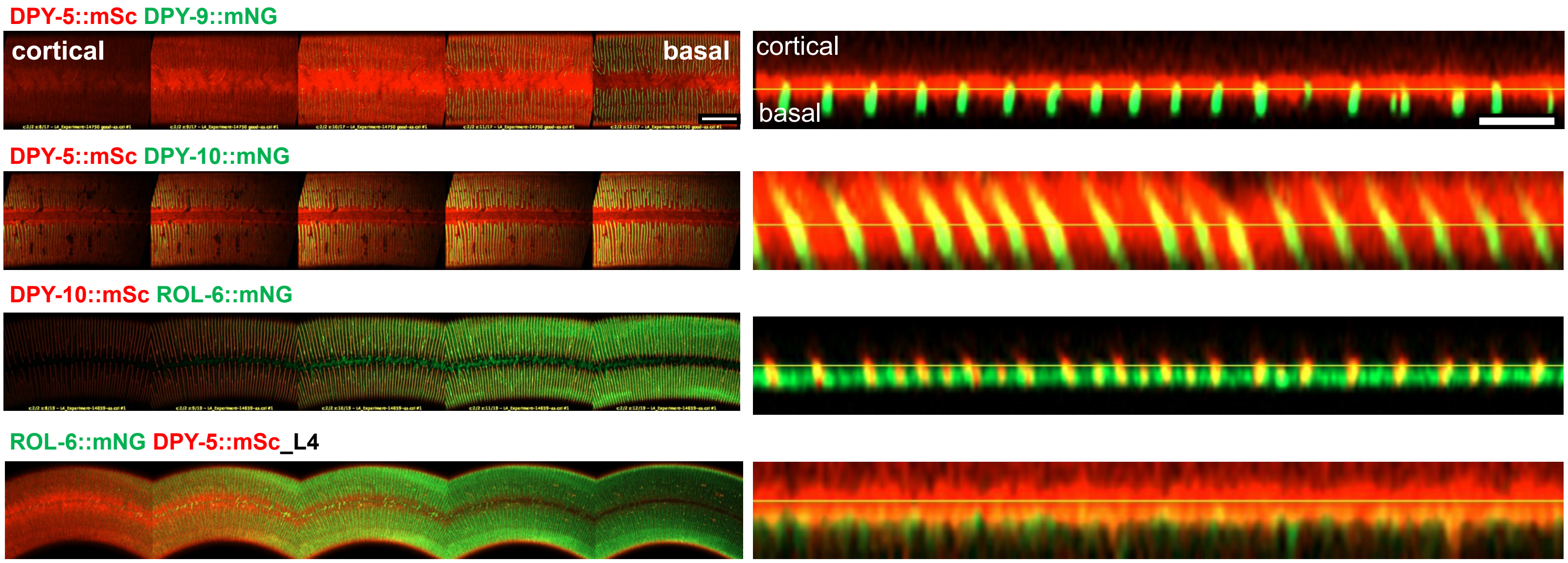
DPY-5::mSc DPY-9::mNG
cortical
basal
cortical
basal
DPY-5::mSc DPY-10::mNG
DPY-10::mSc ROL-6::mNG
ROL-6::mNG DPY-5::mSc_L4

Figure S12
