# Supplementary material for: A systematic fluorescently tagged protein toolkit for dissecting apical extracellular matrix patterning in C. elegans
Source: PLoS Biol. 2026 Oct 14;24(10):e3004059. doi: 10.1371/journal.pbio.3004059 (PMC13705611; doi:10.1371/journal.pbio.3004059)
Supplement: Supplementary file 13 [file pbio.3004059.s021.pdf]

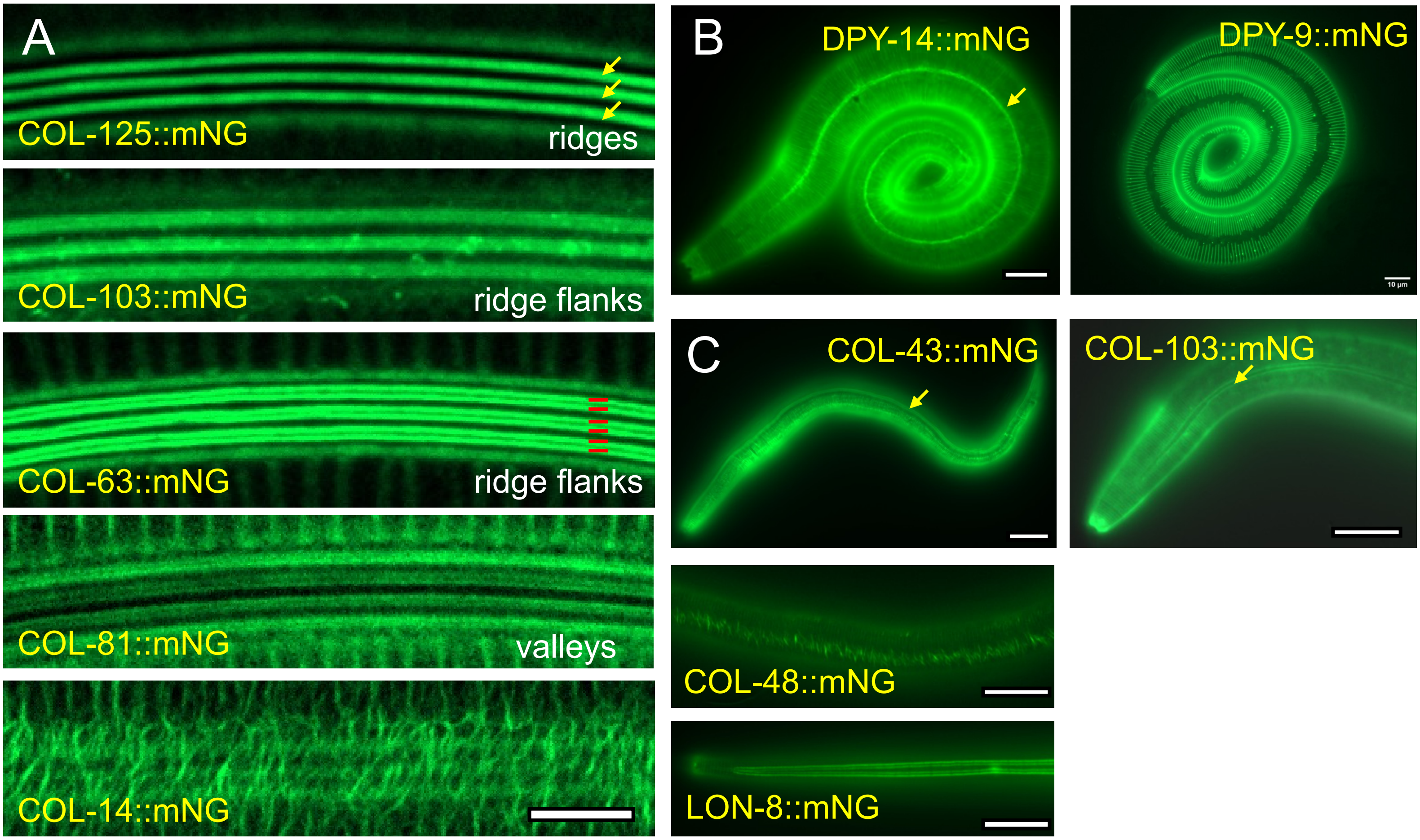
A
COL-125::mNG
ridges
COL-103::mNG
ridge flanks
COL-63::mNG
ridge flanks
COL-81::mNG
valleys
COL-14::mNG
B
DPY-14::mNG
DPY-9::mNG
C
COL-43::mNG
COL-103::mNG
COL-48::mNG
LON-8::mNG

Figure S13
