# Supplementary material for: A systematic fluorescently tagged protein toolkit for dissecting apical extracellular matrix patterning in C. elegans
Source: PLoS Biol. 2026 Oct 14;24(10):e3004059. doi: 10.1371/journal.pbio.3004059 (PMC13705611; doi:10.1371/journal.pbio.3004059)
Supplement: Supplementary file 14 [file pbio.3004059.s022.pdf]

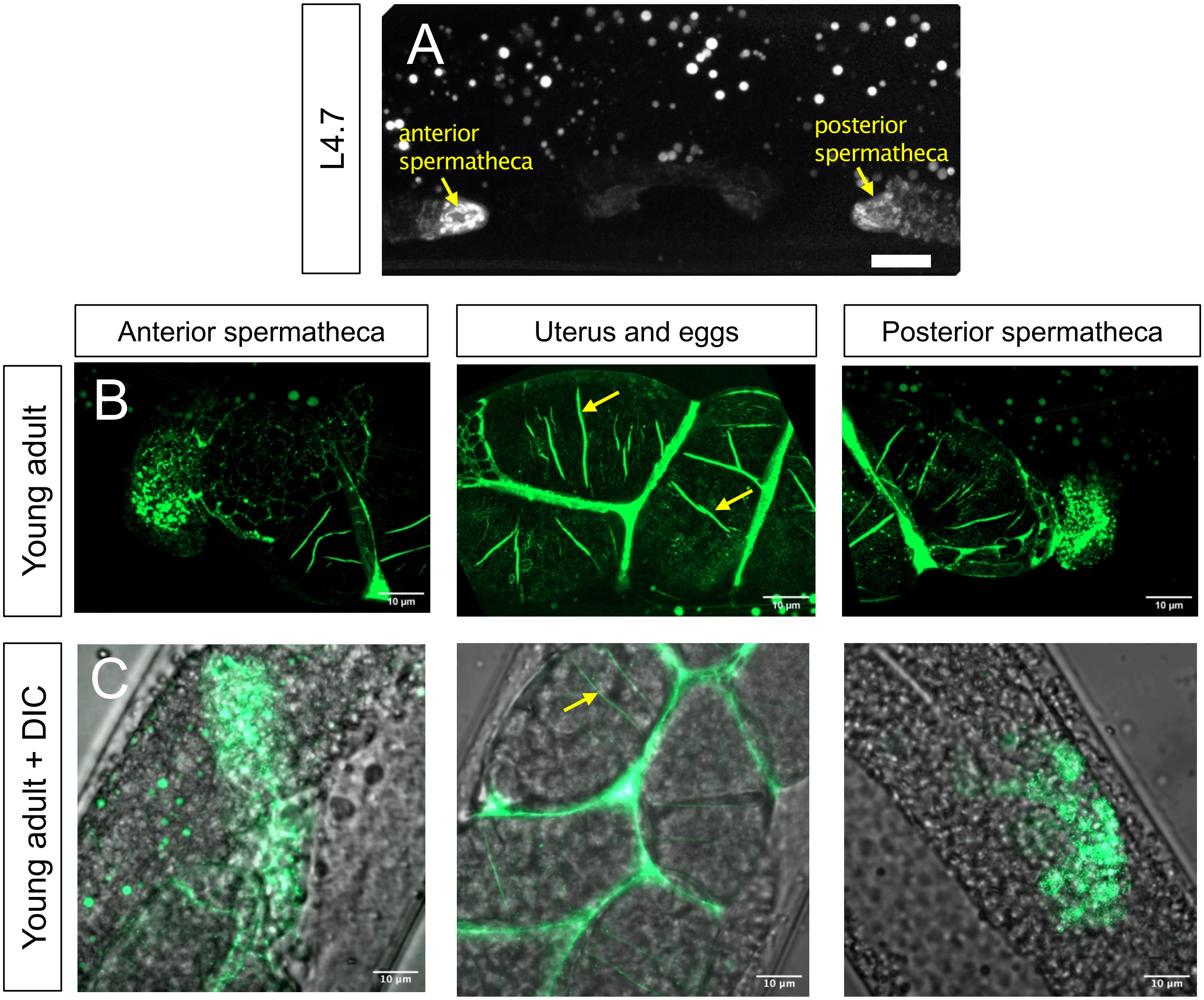
L4.7
A
anterior spermatheca
posterior spermatheca
Anterior spermatheca
Uterus and eggs
Posterior spermatheca
Young adult
B
10 µm
10 µm
10 µm
Young adult + DIC
C
10 µm
10 µm
10 µm

Figure S14
