# Supplementary material for: A systematic fluorescently tagged protein toolkit for dissecting apical extracellular matrix patterning in C. elegans
Source: PLoS Biol. 2026 Oct 14;24(10):e3004059. doi: 10.1371/journal.pbio.3004059 (PMC13705611; doi:10.1371/journal.pbio.3004059)
Supplement: Supplementary file 15 [file pbio.3004059.s001.docx]

**File S1. *C. elegans* cuticle collagens with sequence motifs annotated**

**Conserved W** in homology box B (frequent but not invariant)

Dibasic cleavage site aka homology box A (RXXR, or similar)

Cys clusters domain I, II, III

**Signal sequence** or Transmembrane region predictions from DeepTMHMM v1.0.24

**C-terminal propeptides (predicted cleavage at ^DGG)**

>COL-2

MDIDARIKAYKFVAYSAVTFSVVAVVSVFITLPMVYNYVNNVKKQIHTDVNFCKVSARDI**W**SEVHLIKDAPGNNTRVARQAYSTGGAGGGGGGGGGGCDGCCNPGPPGPGGSPGKPGKPGKPGAPGAPGNPGKGASAPCEPVTQPPCQPCPGGPPGPAGPAGPPGPPGPDGNPGSPAGPSGPGPAGPPGPAGPAGNDGAPGAPGGPGEPGASEQGGPGEPGPAGPPGPAGPAGNDGAPGTGGPGPAGPKGPPGAAGAPGADGNPGGPGTAGKPGGEGEKGICPKYCAI**DGGVFFEDGTRRR**

>COL-3

MDLEQRIKAYRFVAYSAVAFSVVAVISVCVTLPMVYNYVHHVKRTMHSEINFCKGSAKDI**W**SEVNHLKAIPSGNRTARQAGYDAGVTGGSASAGGCDACCLPGAAGPAGTPGKPGRPGKPGAPGLPGNPGRPPQQPCEPITPPPCKPCPAGPAGAPGPAGPAGDAGANGNPGAPGNDGQPGAPGNKGPSGPNGNPGAPGAPGQPGQDAPSEPITPGAPGPAGPAGPQGPPGAPGQPGHDGQPGAPGPKGPNGNPGAPGADGNPGAPGQAGTSGGAGEKGICPKYCAI**DGGVFFEDGTRR**

>COL-7

MSSATFLSVMAGLSGIVVFGALISVFHIYSDINSFVEDSHRELGEFKGFANDA**W**NSMINQDDSVRMARSVFGRRRQKKQSQCNCGQQASNCPAGPPGPPGASGDKGHDGQPGQAGKPGQPGVAGPSHHQKQECIKCPQGLPGPAGVPGQPGPKGPNGNPGAPAQGGGQGPPGPPGPAGSAGSPGQAGAPGNPGSPGKSGQRGRGLPGPSGAPGPQGPPGAPGQPGSGNAPGPAGPPGPAGPNGQPGHPGQDGQPGAPGNDGTPGSDAAYCPCPTRSSVLRHRNVNRHRAAASKKRVVAKKRVAKKRVVAARRHVQA

>COL-8

MLVCVFVALYTMMGLLTDIKQLQSDFDDEMFEFRAITKDT**W**QRIVTKHTYPGGVDEETIESHPPTFETLFGTRKARQAYPEQCNCGPKSEGCPAGPPGPPGEGGQSGEPGHDGDDGKPGAPGVIVAITHDIPGGCIKCPPGRPGPRGPSGLVGPAGPAGDQGRHGPPGPTGGQGGPGEQGDAGRPGAAGRPGPPGPRGEPGTEYRPGQAGRAGPPGPRGPPGPEGNPGGAGEDGNQGPVGHPGVPGRPGIPGKSGTCGEHGGPGEPGPDAGYCPCPGRSYKA

>COL-9

MESCVDLKLRIFKFVAYSVVGSSVIATAFLCVTLPLIYEHAQNGMMTISREGAYCKKSINEI**W**FDMTEDVDIVHNRTIREAYTVTRGEGNTGYAVGRGSNIKPRGYNTGYRSQKKKNNGWRSPPRSGIVVASEGGTCMGCCNPGPPGPSGHPGKPGRPGRPGGHGMPGMPGLGASPCQPRTQSQCQQCPQGPPGPPGLNGQPGLPGGMGPPGQSMSNVLPGPLGPPGPPGQPGNHGLAGQPGSPGQSVLGGIVGFAAPGPMGPPGLMGPPGLDAQNGANGFGPPGLPGPPGPPGRPGSDGNPGVPGQSGGSRRHGEKGVCPKYCAM**DGGVFFEDGTVQR**

>COL-10

MEKFLVTLSTGAASIAVLAVLFTVPSLYNTINEVHDEVLDGVSVFRVETDSA**W**TEMMDIQITVTPPTKPRVNPFNSIFRQKRQTFSGLPAWCQCEPTKPTCPPGPPGPPGQPGAPGTPGAPGPKGDDNTATFAPLTCAPVSQDCVKCPEGPAGPAGPEGPAGPAGPDGQPGAPGNAGNPGSDGQPGAPGDNGQDGAPGQDGQPGAPGQDGQRGSGAPGGPGAPGNAGPAGPAGQDGAPGQDGQPGPAGPAGQDGAPGNAGSDGQPGAPGGPGLPGNDAAYCACPPRSAVFVSRH

>COL-12

MTEDPKQIAQETESLRKVAFFGIAVSTIATLTAIIAVPMLYNYMQHVQSSLQSEVEFCQHRSNGL**W**DEYKRFQGVSGVEGRIKRDAYHRSLGVSGASRKARRQSYGNDAAVGGFGGSSGGSCCSCGSGAAGPAGSPGQDGAPGNDGAPGAPGNPGQDASEDQTAGPDSFCFDCPAGPPGPSGAPGQKGPSGAPGAPGQSGGAALPGPPGPAGPPGPAGQPGSNGNAGAPGAPGQVVDVPGTPGPAGPPGSPGPAGAPGQPGQAGSSQPGGPGPQGDAGAPGAPGAPGQAGAPGQDGESGSEGACDHCPPPRTAPGY

>COL-13

MSEDLKQIAQETESLRKVAFFGIAVSTIATLTAIIAVPMLYNYMQHVQSSLQSEVEFCQHRSNGL**W**DEYKRFQGVSGVEGRIKRDAYHRSLGVSGASRKARRQSYGNDAAVGGFGGSSGGSCCSCGSGAAGPAGSPGQDGAPGNDGAPGAPGNPGQDASEDQTAGPDSFCFDCPAGPPGPSGAPGQKGPSGAPGAPGQSGGAALPGPPGPAGPPGPAGQPGSNGNAGAPGAPGQVVDVPGTPGPAGPPGSPGPAGAPGQPGQAGSSQPGGPGPQGDAGAPGAPGAPGQAGAPGQDGESGSEGACDHCPPPRTAPGY

>COL-14A

MAKRARLRLILLAKLVSLK**M**SDEKEKRSLRPVAFVAVVFSTVAITSCLITFPLILHYIQTLESQVQLDLEFCQARARDMWKEMLDIETGGKKDSAKLANIVLNHRRLEKRDTLQDFWARRLHDQELRDQPVGYDNPSVGVESFNSEGGGCCTCHRGPPGPAGDGGRDGADGVDGTPGEIGPPGPPAPPGPDPHSLFPPQCPCEAPPGDGGPPGQPGPDGPPGAPGNAGEDGKPGDQGPRGPPGIPGAPGQPGRPGPPGEPGTYKTEVGPAGRAGAPGRPGPPGQPGPAGPPGENGKGGGQGPSGLPGPPGQPGQNGAPGEVGQPGDNGAPGSCDHCPPARLAPGY

>COL-17

MEEKQKLVEVETVKKFAFFGIAVSTVATLTAIVAVPMLCMYMQNVQSGLQDEINFCRTRSDSLRGEYTKLDQFRKTESREKRQTYQCCSCGIGPAGSVGNPGQDGAPGNDGRPGAPGAPGPDAPNDHVQPTPADFCFECPPGPAGNAGNPGPKGPPGQPGAPGEQGPNGRPGTPGAPGPQGPPGTPGQDGTPGQPGAPGQVRTVPAPPGNPGQPGEPGAQGPPGPDGHPGSAGPQGPPGPQGDAGQDGAPGNPGAPGETGEPGKDGSKGSCDHCPPPRTAPGY

>COL-19

**MGKLIVVGSCGVLVCVLASLYTI**GNLLNEIEELQVEFQDGMVEFRAITQDT**W**ARMVSKHINPTGHTNAPPTFETLFGARTARQAGFEQCNCGPKSEGCPAGPPGPPGEGGQKGNPGHDGDDGKPGAPGVIVAITHDIPGGCIKCPPGRPGPKGPSGLPGSAGPAGGNGRRGPPGPVGGPGEQGPQGDAGRPGAAGRPGPAGPRGEPGTEYKPGQPGRPGPQGPRGETGPAGNPGAPGNDGEAGKNGNAGRPGPPGHPGKNGVPGQKGEDAAPGPDAGYCPCPSRAAYKA

>COL-20

MADEKSIKYAEADSVKKFAFFGVAVSTIATLTAIIAVPMLCLHMQNVQSGLQEELVFCKSKNADLKSEFFKLDKVRSNAVSDRQKRQSEYQCCSCGIGAAGAPGSPGEDGAPGDDGKPGNDGEPGRDSTDKDEKPTAADFCFDCPVGPAGPAGGPGPKGPPGPPGGHGEPGPAGRGGPRGPPGPGGPQGEAGEDGGAGNPGTPGQVRSLPSPPGEPGQPGEPGGPGEPGADGRPGHPGRAGPPGPPGDNGPNGEPGKDGEDGAPGAAGNAGAKGSCDHCPPPRTAPGY

>COL-33

MEVQEIKNRMKAYRFVAYSAVAFSVVAVISVCVTLPMVYNYVHHVKRSMQNEIVYCRGSAKDI**W**SEVRTLKTALEPIQNRTARQAYADAAVHGGGGGGGNCEACCLPGPAGPAGAPGNPGRPGKPGAPGLPGNPGKPPVQPCEPITPPPCKPCPDGPAGPPGPPGAPGDAGTNGAPGAPGGDAPPGEAGPKGPPGPPGSPGAPGEPGRPGDDAPSEPLIPGEPGPQGPPGPPGQAGPDGQPGAPGGPGTPGSKGPPGNPGAPGADGKPGAPGQAGPAGAKGEKGICPKYCAI**DGGVFFEDGTRR**

>COL-35

MSHSTSYYFILSSTIGCSLIVVASLLTLYSISLDIQQFEMNINSEMVHFKSRANDV**W**NKMVRENLPQNQATSPSFGRYILAKRAAGYATGGGTERECNCAAQSTGCPAGPPGAPGEDGAPGEPGTAGTDGENGAAGVADSFLGVEGNCIKCPAGPPGPPGPDGNAGPEGPSGAPGADGDAAGPGEAGPAGPAGPPGPDGQPGAPGPNGQPGAAGTTSTNEPGSPGPAGPPGPAGPAGEDAAAPESPPGPPGPPGPPGKDGEAGAPGAPGGPGTDGAPGVDAAYCPCPPRTAGLAQEAPAGAATGADASSGAAGGAPDAGAGAGDGATGTYSGSAVVRRRARF

>COL-36C

MKIDKEDDQQQQMRRVAFFAVAVSTAAVISSIVTLPMIYSYVQSFQSHLIMETEFCKTRARDM**W**VEMQVLHKSGVTRSRRDAGYKEGSGSGGSGSGGYGGPTGAGADIGPTCCPCQQGPAGPPGPAGDTGPNGNDGHHGAPGVPGKEGSILSSALPPSEPCIICPPGPQGAVGQQGPKGPPGPKGKSQERAADGKNGEPGMIGPPGPPGGVGEPGPPGPAGQPGRVIQVNGAAGPAGPRGVKGPPGPKGLPGIAGLTEIGGQGPPGDAGGPGPVGGQGPPGPQGPQGPPGDEGSCDHCPEPRTPPGY

>COL-37

MGHVQVLLFVVTSASALAALTAIFSAIHVMTDISNFQDEVISDLSNFKQYADDA**W**GVMVQTGNSPGFRFDQITGRVRRQYATAAVSGGGGGCQCAAQASGCPAGPPGPPGPDGSAGPAGPAGNPGADGDAGSPGEAGGAGPPGPPGPDGNPGAPGNDGEAGSPGTRSISSPGPAGPAGPPGPPGPDGAPGGGSGPGPAGPAGPPGPDGNPGSAGADGQPGGPGQDGAPGSDAAYCPCPPRSGAIAGGGGGGDAGVSGGSDSAVGGGGDTNASTDSQTNRWTKHFQRLRKHSSKTLPFSFSIIYLMGNKHFHLAFTVFIFKCKTRGKNLLG

>COL-38

MSKYLVPVCASISLVAVFGALVAMHSIVVDIDTMREEIVTGVHDMKVMSDDA**W**NRMIGFTKPSLDSESRSAAFASVFRNKRSAYPSQCNCDANSQGCPPGPPGPPGLPGGRGDQGPSGDKGRDGASGVSLAVTHHLPGGCIQCPQGPPGETGPDGDIGEPGFPGASGSAGQCGEDGAPGEAGITGEQGPQGEPGTEGSEGPTGQDGTIGGPGLPGQPGTPGWPGSQGEPGKNGDSGVDGEQGPQGPQGPDGQPGRDADNGQPGLPGKDGSIGPDANYCPCPARAKKH

>COL-39

MTGPTCLAVVAGISGVFVFGALFSVAQIYNDISSFADNAHRELGEFKGFANDA**W**NSMVNHDDATRVARSVFVRRHKKHSQCNCGPQASNCPAGPPGPPGAPGDRGLDGQPGGAGNPGQPGVAGPKSHEQQECIKCPAGSPGPAGAPGAPGPQGPNGQPGHPGQGGSQGPAGPRGPAGDAGAPGQVGAPGNPGQAGRGGQRSHGLPGPSGAPGPQGPSGAPGQPGQSGGQGQQGPAGPAGPDGQPGQPGQDGQAGAPGNDGAPGADAAYCPCPSRSGSSSAVETGAAEQGYRHRAVAARHRNVIRRRVAKKRVVKKKRVVARQA

>COL-40

MEEKQKIAEAESLKKLAFFGISVSTIATLTAIIAVPMLYNYMQHVQSSLQNEVEFCKHRTDGL**W**DEFHRFETVKGVDSRIKRDTRSRRGGYAEGGAAAGGGGGGGGSCCSCGIGAAGPAGAPGKDGAPGEDGKAGNPGTAGSDAEAAAAPTASDFCFDCPPGPAGPAGGPGPAGPPGPAGADGNTPSGGGEGPAGPPGPPGPAGNPGTDGAPGNPGAPGQVTETPGTPGPAGAAGPPGPPGPAGNPGSAGASEPGPAGPAGDAGPDGAPGNAGAPGAPGEAGAPGSGGGCDHCPPPRTAPGY

>COL-41

MSTLGYIGAGVCLLGVFSALCSIGHIVQDINNLRSEVEGRVDEFKVLADDT**W**DRLLILQSPTGESANPVPSLLRNKRFVYPGMCNCDSNSQGCPAGAPGPPGNPGKRGDEGHPGDEGRRGASGISLATTHDIPGGCIKCPEGPAGPPGPDGDSGPEGFPGLQGQSGPSGEDGAPGQEGAPGDQGEQGPKGYDGTDGPDGQPGTTYFPGQAGQPGEPGWLGEPGLPGQHGEPGKDGEEGPQGAPGTPGNAGHDAFPGTPGQAGKPGAPGKDANYCPCPQRQDDRTPPSSGTSAPQPPPRGSTAAPGTRAPPATRAPPATRAPPATTRAPPATTRPAPASQPPVREPETPDSGYPSPAPQEPAHPSPSYPSPSYPSPSYPSPSYPSPSYPSPSYPAEPAYSVPPPAKPEQPSGGYDAPSPPQTGSYQRRW

>COL-42

MEDKQEYLRKESEIGTLRSVALGAVTITAFSLGTSILLLPLLFSHAQTVQSTLEHELQFCVLRSHDLFDELRKVEAVTGQQSRIKRQYVTGPMGGGGGYGGGGYGHQQQQQNYGHQGYAQRPQQPQPVYRPSAPVYQPQPQVQSSICIFGPPGPPGFPGSDGQPGRDGEAGAPGQPGRDGAGGYGGPQQNDCQTCAPARQGAPGPPGPAGQPGQPGEPGHSDTSSTAGPPGPPGPGGPPGQDGHPGDAGAPGAPGQVVAGPGSVGPPGPQGPPGLAGPDGQPGHGGGGQPGPQGPPGDDGHPGEDGGAGGPGQPGQDGGSGGSGACDHCPPPRVAPGY

>COL-43

MDSSDGRTKAYKFVAYAAVSFSIVAVLSVVLTLPMVYNYVSHVRQQMNHELSFCKGSAKDIFAEVNFMKTHSGPAAPGNRTARQSGYGQPEVNPSANLQCEGCCLPGPPGPAGVPGKPGKPGRPGAPGQPGVPGKPPTAPCEPTTPPPCKPCPQGPPGPPGPPGAPGDPGEAGTPGRPGTDATPGLPGPRGPPGPPGEAGQPGPQGDAGTPAQSEPLTPGAPGEAGDAGPAGPPGPPGAPGNDGPPGPPGPKGSPGPDGPAGVDGQAGPPGPPGQAGTPGEKGICPKYCAL**DGGVFFEDGTRR**

>COL-44

MECPTDLRLKAYRLIAYSAVGFSITAAVSLCLVLPLIFSYVQNTKTQLKKDSLYCKTSVQDM**W**AEINNEVHEITVNRTARQAGYTSGGGADAGAGASSGGQCEGCCNPGPPGVAGNPGKPGKPGKPGAPGNPGAPGKGAAVPCEAKTPPPCKPCPAGPPGPPGPDGPAGPAGPDGEAGSPAAPSPPGPPGPSGPAGPAGNDGAAGTPGPDGPAGESTYPEPAAPGPAGPPGPAGPPGPDGASPTAAPGAAGPPGPPGPAGAPGPDGNPGTAGPPGKAGGEGEKGICPKYCAI**DGGIFFEDGTRR**

>COL-45A

MKSLIEEDDLEVGRFRRNAFSAVALSTSCILAIVVLTPIAYQAIQRIHSNLLNDAHFCQARNRDL**W**TETMKMARSRGRDEELVARTKRAVKGTWLFGQYIPDRSSRNRRQQYAEAAGGGGGAPHAECRRGPPGPPGDDGVDGTDGKDGAPGTDGKDGQVPQGPADGGYQEGPSDAVEACTRECPPGPPGPTGAPGDKGSRGYPGESGEPGTPGSAGPKGNAGPAGPPGPPGYPGRPGETGDHGKTIAGEAPPGPPGRQGEMGPQGPPGPPGPRGKDGAGGEKGAPGDQGNPGPYGKPGQPGAPGPDGSAGEKGGCDHCPPPRTPPGY

>COL-46

MNENARETAYATLIIVSLFISLSTMSSFIVSVGPLASILRPAIQNTVNDAEYCQESALEMKSMLSDVANDFQNITRQKRDEIDDFLHETGQTARSRVCECRHPQGPPGIPGRDGLPGTKGSEGAPGLPARLPCEPPVNYKSLCADPCPRGVQGTTGITGVKGDTGKPGLPGKNGLRAENGKMGPKGLQGPQGIPGLDGEMGDPGADATPKPFIPGPAGPIGEEGEFGPPGPSGMPGIDGPIGAQGPRGKKGKPGFPGNDGAAGAEGLIGEHGEEGNRGVCPTYCAT**DGGVFFVQPPDWMLQ**

>COL-47A

MKNISREVGSVVLEEKRGKRDYTEASKCTCSSTISQKCPPGSKGAPGADGLDGIPGSRGLNGMDGEAGHSNMDVFMSPTGCIRCPAGPPGSPGDHGSPGEPGYPGPSGPPGEPGYATIGLIGPPGLAGQDGLDGAPGEPGVGGMDAVKIVSVDGKIGRRGEAGNKGSKGSRGGVGKAGGPGVAGEMGEKGARGEDGKPGESGKPGAPGIPGKDGTYCRCPDRNRYDIYPYKSKI

>COL-48

MDEKSKGSLRSVAFVAVTFSTVAVTAVLIAFPLVFHYVQTLQASVQGEVEYCKSRSRDM**W**REMVEVAPEGADDPLDVLLRATRQADAQCCTCQQGPPGPDGEPGTPGKDGEPGQGPGEPGPPGPDAELHDRILPVPPQCPCTAAPGLGGPPGPPGQDGIPGNPGRNGEDGAPGPQGPSGPPGPPGQPGQPGQRGPPGEPGALLPGGDAPPGPSGPPGRPGAPGQPGKAGSPGQDGSNGDAGVAGEPGQRGPPGPPGQAGTPGSPGDAGSPGSCEHCPPARLAPGY

>COL-49

MWKFVIGSVSTAAFFVSVCTIYFSVSMLDELDSFRLSIRDELED**W**KEVSDDT**W**QRLNDMTSRNVPKKTNILKEFVRGKRNVGNDQCNCAEPTKNCPAGPPGEKGSLGNPGQPGPDGVDGDNGVDGDVVIHDMPNPKECIKCPAGPPGPPGPPGPLGPRGDKGPSGPRGALGDQGETGPVGEIGDQGPPGSAGRAGPRGQAGQPGTIAIVGLAGRPGPQGPLGEPGAQGEPGVDGKDGALGAPGRKAENGRPGKRGKDGVAGVPGTRGKEGEDAGYCTCPPRTA

>COL-50

MSSGTYYALSAGTAVAAILVAGSIVSMVSIIGDLQSFETDFVDDMSAFKHKANDA**W**SQMMVARRVESPTERLAFEGLFGRVKRQYAGGDAAAAATPAKEGYAEGAGGGGGCQCAAQASGCPAGPPGPPGEAGADGEPGEAGQDGAAGEAGSADTYAGAAGNCITCPAGPPGPPGPDGNAGPAGPAGAAGPDGEGAGYAEPGPAGPAGPPGPDGQPGAPGPDGQPGAGGTTSTNQPGPPGPAGPPGPAGPAGEDAYAQPSPAGTPGPPGPPGKDGEAGPDGPAGAPGTDGAPGPDAAYCPCPPRTLGAGAYPEGGDAAAAAPAGGYDGGAGAAPEAAPAAAAAPQPAPAPAAAAAPAGGYQGGAAAGAAAPPPAPAAAAAPEPAPAPAAAPPPAPAAGGSPTGGYRRKKVRRVLRIVRS

>COL-51

MSSSASFYALTGVTGVAAVIVAGVVFSYLSILGDINQFESDFSSDMSVFKTKANDA**W**SQMMASRRIESPSEKVAFEGLFRAKRQYATAAGGGGGYAAGGGGGGGGGGGGGGCHCAAQASGCPAGPPGPPGEAGTDGEPGQAGQDGQPGQAGQADSGSSGQACITCPAGPPGPPGPDGNAGPAGAPGVPGPDGDAGSPPPPGPPGPPGPPGNDGQPGAPGQDGQPGAPGTNTVNSPGGPGPAGPPGPPGPPGQDGSGGAAQPGPPGPPGPPGNDGQPGGPGQPGGPGQDGGPGTDAAYCPCPPRTPAGGGGGGDFPAGGGGGGYSTGGGGGRADSGGAAGGAGGAGGYSGGGGGGGGGAAAGGGYNAGGGGGGAPQAAPAPQAAPAPAAPAGGGYNAGGGGGAGGGGGYAGGAGAGSYRRRFQ

>COL-52

MVPADTYQSVLASEHHLPDAVAAVDGSSLGSINGVDVRKKEKLVKSRKKLEKKKNYLEICLADCQRKSKNCPEGPPGIPGKPGKDGIPGEPGVNGTNGFSGISVLVANGCIQCHIGKPGPRGKRGKRGKKGKDGKNGISVKAVIGLPGPTGEVGWQGYPGFPGPVGPVGPPGNNKNRYIGLPGPRGRAGRKGYQGAQGNAGFIYNTGIIGPSGRSGKNGLNGRTGATGHPGHTGRPGRDGNYCECPPMSRRNPFYNLLSA

>COL-53

MSYIWRQLGSPTVAITLSVSTVIYVTVICPIVFIHLFGDHLNVLEDDECPIQMSDLRQRVSFVREQIHENTRKKRELASCCIGPPGMKGFPGKNSRDGYDGEPGRDGQPGRPADGDSRMVCIRECPTGRPGREGGDGPKGSKGQRGRTGQQGDSAPPSIRGEPGERGDKGFPGVAGPAGEPGEPGHIYVSDGPPGKDGETGPRGPPGEKGRTGRDGEDGLPGERGERGYQGLKGEIGKRGPVGTFGPIGPKGEPWPCESCPPPRVSPGYYVNKRNKKH

>COL-54

MSTTSTVLLFITSVCGVAIISSLLVAGSLFFEAQGFLETSLDEIESFKHYSDVA**W**KDMISSNTFDRKKRDDEDKSCGCPCANGPNNCPPGEAGPPGAPGTPGADGEAGNPGKDGAAGSSLVFEDEKLPCIKCPAGEPGPQGPDGAPGAPGPDGQPGAPGSNGNPGSAGEQGSEGEPGDAGATGEIGVAGAPGKNGNRGSGAPGAPGPEGPIGEPGKDGQQGPHGDVGGDGTEGIQGAPGKDGENGSDGVAGSDGAPGAPGPDAAYCSCPARTKLVFSSESP

>COL-55

MNYLKHVGGDICMRRTTSERVKLFQNIGSGVAVIACLFAVGYSFNDINSFYDEVMDTMTEFKFNEQQA**W**NSMVKPIDVFGRIKRRANTRQAQCNCESQSQGCPAGPPGPPGQPGAQGEQGHVEVSGQPGAPRPQGPAGGPGQPVQSGGSGASGAPGQPGQAGAQGTPGAPGNSGSPGGDAAHCPCPSRSVVVRKHRKVFRKRVDKVSA

>COL-56

MKSMPWIHFLFTIIVILFILSIGIFFYFYSEIRDIQRIADDAIKDFEIYEKMS**W**NDLQVVLISLKSREKRKIRKKSRRQSEKDYLVAFPSSPSIKPPECPSGPPGRPGKPGLDGEPGFDGLPGRDYNYMELMTEHCILCPAGPPGDPGMEGSDGIPGPPGLIGPPGPNGESGVIGSVGAAGREGTPGKRGIGGEEGEPGLIVMKMMNTRGPKGSVGPRGLTGQPGMPGRTVNAPPGSPGIRGRPGMPGASGRSGIIGKRGIHGEPGLDASYCPCPRRSYQMQKMVDPAQEKKFSYDYYEFEVAKSRRNRKSIWNRRRIKYI

>COL-58

MTASSKWDNVAFGLSAACLSAAIVLLVSIATWQSEISRITEEAIVETQHFQETSEKI**W**DDLVFVATGETDRHKRHAYLTRAPVVPVTGGFQTGRTANRGCNCRGPPGPPGFNGEDGYDGEPGVPGSSGGDDISVLRHDPGRCAQCPAGPMGPPGPRGPPGVPGITGAPGLDGIPGRHGTPGYPGEPGLPGPVGDPGFDGKPGNPGNNGVRVRRIPGPPGPRGAEGDSGVQGGPGSPGLAGAPGDSGAPGPQGPPGQKGMDGTPGGFGQPGPQGVDGEYCPCPKQIKKSV

>COL-60

MTGTTTILYGCTVFSSVAILAALLFTTFMYNDLYDFHSEVEEELYGFKDIYNDA**W**FLMTKTASTKFSSTESSSEMDRIFKRNSDSCNCGAKASNCPRGPPGPPGEPGENGTDGQAGQDGQDGNTPSEEYGNSQDGSTSTCPAGPPGEQGPDGAPGEAGRDGQPGSDGQSGNEGPTGPRGSPGNKGRDGNPGRDGSPGRPGRDGTRGVGQPGSPGQPGSRGPEGPRGQPGNMGRPGEYGDAGPAGQPGNDGEQGPDGVVGIPGEPGLPGSDASYCQCPDRTPTVEKTSAGGYKVRRRLVKHAN

>COL-61

MWEVKAVAAVSTTCSLTAIVSCLVVLYGLFQEMDDIELQVNESIRVFQFETDPA**W**HDMMDVQSSVTPPSRAPRNPFESHLKKKRHTYGGLPSWCQCEPTKPVCPPGPPGPPGNSGSPGNPGSRGVPGEDNHQIYEPIQCPLPSRDCIICPVGLPGYPGRDGLPGEPGPNGKPGNPGYRGEDGKPGRPGEPGSSGLPGRPGSDGKPGLPGRDGKKGIGLPGRMGRPGFAGKEGQPGINGKPGRMGLPGPAGPEGKPGYPGMLGRDGIPGEPGLAGGPGPDAGYCKCPNRGNTYRNPYPYRARSKYHKQ

>COL-62

MSSAMFLSVMAGLSGIVVFGALISVFHIYSDINSFVEDSHRELGEFKGFANDA**W**NSMINQDDSVRMARSVFGRRRQKKQSQCNCGQQASNCPAGPPGPPGASGDKGHDGQPGQAGKPGQPGVAGPSHHQKQECIKCPQGLPGPAGAPGQPGPKGPNGNPGAPAQGGGQGPPGPPGPAGSAGSPGQAGAPGNPGSPGKSGQRGRGLPGPSGAPGSQGPPGAPGQPGSGNAPGPAGPPGPAGPNGQPGHPGQDGQPGAPGNDGTPGSDAAYCPCPTRSSVLRHRNVNRHRAAASKKRVVAKKRVAKKRVVAARRHVQA

>COL-63

MTTSGQLVAGTAVALCGVAIVPAIFAALFVLHDINSFQSGVYEDLAEFKTLAEDA**W**TTMVPREDAVQPFETFLKRFRRGYGDAGVAGSASGSSGCNCGPQPKDCPAGPPGPPGNPGTPGDDGPAGRAGNPGSDSTEGDRMADFNKDVKCPAGPPGPPGPNGFPGHPGPDGDFGVDGTNGKDGEPGPDGPEGDEGTPGLPGPPGEDGPVGQNGTRGQGQPGPVGAPGAPGGPGRDGEPGENGQDGQQGPEGPAGADGQPGHPGPDGPSGDVGEVGAPGADAAYCPCPPRSAEMAATGSSDSQPASYEAPAPAATKGYDSPAPAAPKGYDAPAPTAPHPPPPAPVAPPKLHDYESPAPVADAHDAAPAAQPYKRRKVARAHMV

>COL-64/RAM-1(?)

MGYGTNVASGVTVICAAFLIPMLTCTISLFYEVAQMHMSAMDGMDEFKLYANGA**W**EDMLPATRLPREASPTPDSNKDPCDCDTGPAINCPVGPHGPRGRPGRKGADGPHGGQGRKGPDGMDVFKEMIGPVECIPCVAGEPGPPGENGSVGEKGEDGQPGSPGKPGEDGESGTDGEPGFIGFPGFPGAPGENGEQGNNATRGVGIPGPAGPPGPPGEVGMPGVTGETGEDGHDGPEGPEGPAGNIGDIAEEGPDGIPGEQGVPGFDSGYCPCPRRSSSRVSPSGGSSPISDTFIASPTLLPPSTTLSSTPPTISPSAPTLLPTRSPKSREETPTRREEQQVPPNPSSSEVPETGDSANSQNFGGYLALKRL

>COL-65

MSRFLAGTLSAISGIVILGSLVIAGVLFNDINTLYFDVMDDMHEFKLLADDA**W**NKMITVEHNAPNTLDSLFKRGKRQSGSSCNCGPQPSNCPAGPAGPPGEPGQPGDDGAPGKAGDNGHDGIPSPIDAYAQHDCIKCPAGAPGPAGPDGEAGAPGPDGQPGQDGPAGIDGAPGEAGPQGDAGAAGQDGEAGAPGPAGKNGQRGQGAPGPQGPEGPAGAAGQDGAPGEDGAPGAQGTEGIAGAPGKDGETGPDGAAGEAGGEGAPGPDAAYCPCPPRTVGYGEVDQPQAEQSGYRRRAARRL

>COL-68

MASESSHDDPKYEAQEADAMRKLAFFGIAISTVATLTAIMAVPMLYNYMQHVQSALQSEVDFCRHRTNGL**W**DEYNKFESVAGVRRRIKRATYQKSNGVTGRLAHRRRAVARGASSYDGGFNEEVPVGGGGGGGACCSCGVGPPGPSGAPGTDGNPGNDGSPGNPGTPGQDAVGDESPTAADFCFDCPTGPPGPPGKQGPKGPNGEPGAPGTPGGNALPGPPGPPGPQGPPGADGLPGNPGAPGVPGQVIEVPGTPGPAGPPGPPGPIGPPGQPGAPGSSQPGPQGPPGDPGIDGAPGNPGSPGEPGPPGQPGAGGGCDHCPPPRTAPGY

>COL-69

MNLNQSCSLKKFQLSTVIRVSFIASSLLISLCLFLIMMTSREISEFREQTLDDLKE**W**KYFSDAAWNEMTSLTIRKARSPSTTGNSGNAGKRFWAKRSYDSYAPQQVNSYARVNSYATAPVLQHPTQQQCNCAPQANNCPPGPQGPTGYPGAPGEPGSRGLDGRPGANGVTLIAYDQEIPGCIQCPQGPQGPPGAVGPCGPSGIPGLPGNPGYTSPPGLPGPPGPCGEAGREGPNGTPGLKGEPGNNSIRQISLPGRKGPPGLPGQCGPPGPPGYTPPPGPPGTPGVQGEAGQDGEPGVPGVTGYPGVRGPPGQDAEYCPCPPKNAGVNRNSYVQPAPVSTSYATASQSRYVSEYNKEDEYRKRVLARVLKKQRRLLLATRAKNVRRKPVSHASP

>COL-71

MRNAGGDKPFSTPVLLREMGLKTHVDDDPKTKAYRLIGYAAVTFSTVSVICFCVTMPVVFTYVQSVKRQMSHEMATCNVNARQIFDDVAALRAGFPFAQAGNRTARQAGYDVHPSKPTPVGYSGGDAAAAEVKGNSPAAAYEDKPFVAVGVEEGPHTATTGSDSEGTCHDCCLPGPPGPPGPPGRPGPNGKAGANGLNGNPGRPPEAPCEPVTPPPCPPCPAGPKGAPGQAGYPGADGQPGSQGDNGEKGSDGAAGEKGRPGPLGKIGEPGATGETGENAENSEPTPGPQGPPGAIGPVGSRGTPGHPGEDGEAGAPGAPGENGTDGENGEDGVPGVPGHDGKAGRAGERGICPKYCAK**DGGIFFEDGTRR**

>COL-72B

MGWLTDFVGYSSILTSILVITCTMAYIPYMLHRMEAITISLKVHNDEFLVLETEARSQLGELRSDYPRVRRATNRKRQAGECQCGGQNVCPGGTKGQPGRNGEDGTPGQPGQPGEPGITGMTPPTDYQNEKSCRQCPPGPRGQPGYGGPPGPPGLPGQPGQNGNGGRPGNNGPMGQPGEPGREGQPGKAGGPGSPGRDGIRGSKGSTGDKGPNGPPGQNGPPGWAGRDGNIGPQGTPGGRGEPGGPGTDGFPGFAGNPGPDGAPGVDSGYCKCPSRGAYARTASSKRS

>COL-73

MSQDDKQKLVEVETVKKFAFFGIAVSTVATLTAIVAVPMLCMYMQNVQSGLQDEINFCRTRSDSLRGEYTKLDQFRKAESREKRQTYQCCSCGIGPAGSVGNPGQDGAPGNDGRPGAPGAPGPDAPNEHVQPTPADFCFECPPGPAGNAGNPGPKGPPGQPGAPGEQGPNGRPGAPGAPGPQGPPGTAGNDGTPGQPGAPGQVRTVPAPPGNPGQPGEPGAQGPPGEDGRPGNSGPQGPPGPQGEPGQDGAPGNPGAPGEAGEPGKDGAKGGCDHCPIPRTAPGY

>COL-74

MTSTATAYAAITVGLVSIGFILFAVQGISSNIEDIRNEMNIEMDA**W**KFETDSLYRDMQKFGRVKRQYGGYGASGANPTPHGGFPGGASPMNPGSFPGVVGVPPQFNPTQGGEPNPGGSCNCQADNSCPAGPAGPKGAQGSDGIDGIPGVPGIDGQDADDAQAQTQQYAGCFTCPQGPQGPQGPQGKPGVRGMRGARGQGAMPGRDGTPGQPGSLGPVGPVGPQGEPGSEGEPGADVEHQVGLPGPKGISGPTGQPGDQGPQGDTGAQGIPGAPGERGPRGDKGDNGENGSAGAPGEEGEPGTDAEYCPCPQRQESAPVNGNQGYKNRRH

>COL-75

MRASPLLSLSIFITFVSIFSLLTYLPSFFAQISRIERQLEEELDEFLNIEHEFRKEFDIYPKNYERRQKKHVYSYCECEYINTCPPGRIGITGPDGQPGKDGLPGMPGSPGIPGELPNILYQQVFGCRICPYGPKGKSGYKGIDGPPGIPGWPGEPGVRGMNGERGSTGLDGAPGEPGSPGLPGFAGLPGSIGITGIKGVMGEVGEPGAPGIPGEEGLSGPTGQPGSPGSIGAMGYEGAYGDRGEPGPPGPIGRRGGPGLDSHYCPCPSRKMFLSILREKTKKQL

>COL-76

MLVPHSPQSILVVSTVISTFTFLATITLLPLFYMKVQRINSQMLTDLRNCHQDSTDI**W**KQLSQENVRGKRSYGRDAPSIPMGKCCSCQQGKPGPTGPKGPDGGPGQPGLIGLNGRNGRNGKYVASEAHNEPACQKCPVAPPGPPGHPGRKGPRGTAGNAGTAGKNGIPGRMGPPGPAGVRGPPGELGMRGPQGDPGKVLNGAPQGQPGRPGKVGSRGKAGHTGRDGRPGLEGTPGTRGVQGERGSRGARGPPGPPGPAGSDGRKGSCAHCQSDDKLSQGGANRRPEETDPEGEFSNQQPASYEEQPPVITTTTTETAPVYTNPPAHQPVHRSFPSAEPRQSHKNSPSVPVPQEQYNSANSEVIPKTINYPDTINHQSYKHDGDVSVAGTTDDSEDVNVIPAGQYSSRQYASKTNKIGKAVHFGYSNDQLSSFVPMGATSEEDENYGKPRAFQTNKGYNVQIMGSGPTYNAAPVGNAWKQPENHKVRQYTPQYLPPPAYA

>COL-77

MYQQDEKKLMQEAEGLRKIAFFGICISTVATLTAIVAIPSLYNYMQTVQSTLQTEVDFCVHRTNGLFEQYERIKGVKKLVKRQAGYGAPAEYSTDAAVSAGGSEAGGQCCSCGSGPAGPPGTPGEDGRDGNDGQPGPDGQPGSDAPAEAIPTADDFCFDCPAGPPGPAGNAGPKGAPGNAGAPGNDGQAGAPGAPGNDGPQGPPGQDGAAGQPGPDGQPGVVEEVAVPAGPPGPPGPQGAPGTDGQPGSAGQPGQDGPQGPAGDAGTDGAPGQAGAAGEQGEAGQPGEGGGCDHCPPPRTAPGY

>COL-78

MKTFVSHVSHHTDEVAKSVKSAKKFAFFAIISAVVTSSLIIFIIPAVFFRFEDDYHSGYSYNTPINIYQPVSAPTAAPDVSKCCTCSQGPSGEKGDKGSDGRDGNDGYPGSPGKSGQDALERMITDGYIDFCFECPPGAPGSPGEVGHKGPEGVLGPSGKKGKTGQSLPGPVGPPGPPGKDGSIGAPGPDGASGKILTMEGPPGLPGVPGSVGLPGIPGKPGPSGPDGKIGATGPGGDPGKPGGNGIPGEPGPIGNQGPRGSDGSCEHCPPARTPPGY

>COL-79

MDSRFVTYFASAISASVLVTTIFVCWNVTHDLNTLQAMSDRNMQDFKAISDRT**W**DKMMFKQQSSPPSPSLIFGRNKRSGDKCNCSEEPSNCPGGPPGPPGEKGNDGVDGVDGIPGFPGENGGAALDQPADGTCIKCPPGPRGPPGPQGEEGPSGDVGEDGEPGVPGNDGADGTPGKSGAPGGKGPQGPPGTPGRPGQPGRKAIGEAGPKGPPGAPGTDGRRGENGTDGDDGVDGQPGDEGDAGKDGTPGEPGPQGEQGTEGQPGTDGAYCPCPARSISKVAIQ

>COL-80

MSTTFLSVMAGLSGIVVFGALISVFHIYTDINSFVDEAHRELGAFRGVANDA**W**NSMVNHDDSARVARSVFVRRQKKQSQCNCGPQASNCPAGPPGPPGASGDRGLDGQPGPAGKPGQPGVAGPAHHQQQECIKCPQGAPGPAGAPGNPGPQGPNGNPGAPAHGGGQGPPGPPGPAGDAGSPGQAGAPGNPGRPGQSGQRSRGLPGPSGRPGPQGPPGAPGQPGSGSTPGPAGPPGPPGPNGQPGHPGQDGQPGAPGNDGAPGSDAAYCPCPARSAAVFRHRNVAVNRHRAVAKKRVVAKKRVVARKRVVAARRHVQA

>COL-81

MEDKRILVEKEVESFRSIAFVGISVTIAAALIAVIAIPAFYSYVQHVQSKLDSEVDFCKHRSLSLHNQYEVVEEFTGVPSKFVVKREAHRGMSKRRVARRKAIRRRQAECCSCGVGAAGPQGPPGRPGNDGNDGQSGNPGSPGQDGSDSYEHQQNREFCFECADAPAGPPGAPGQAGAPGNDGRPGQSGGAAIPGPPGPPGPSGQPGQDGHPGQDGQPGQPGESREVPGAPGPAGQPGSQGEPGPSGPDGRPGQPGQAGQPGGPGDAGNDGQPGQPGGPGEDGEDGDSGSSGSCDHCPPPRTAPGY

>COL-83

MTWQQQKHRPEADDVEIRTLRVVTLISVGITVAAILLGLFGSVILFAKTLTFQNELEYELRYCVSRSHDL**W**DILSQLETSKGVAISQRRKRQFFMNYCNDVTNCGGPSDCGQETGCAIHPVHSCGGGGGCSQPVVVQQPVIQQPIVHSPQQQFCIFGPPGPPGIPGLDGNDGDDGSEGYPGVPGANVPDTVLPEIDDFCFDCPPARSGPPGPPGPKGPRGTPGPTGPKNFYITSGPPGPPGPPGPPGNPGGIGPRGPPGDPGIMTQDSRGVEGPSGPSGPPGPMGFPGPKGIDGFSQVGSMGPPGNRGAPGRVGLNGPRGPPGDIGPPGSAGGCSACPIPRVSPGY

>COL-84

MQVHTATFVASSLSGIALLCSLLALGQIFNNVQDYWTELDTEITSFRQETDAM**W**KDLVQIGTNAATNRRGRRDSGYSEGGYAASGNTGGSYHPGAAPAHDFGAQAPAGPAPTGGSCQCQSAAENKCHAGPPGPKGSPGSPGPNGVPGVDGKPGHDSLDVTPEPQDTGLCYYCPGGPPGAPGPNGRPGLRGMKGADGGLGRTGHDGNPGHPGEMGAPGPQGKQGPDGRQGEKGADGRKPIGRPGPKGQRGPQGEQGPVGDNGRNGPVGGAGPAGPQGGSGGPGEKGLDGGPGSEGKVGRPGKDAEYCPCPARESHGGTGGRGGSGGAGDHYQKA

>COL-85

MADEKLKLAEIESLKKVAFFGISVATIATLVAIVATPMMYNYLQHVQSSLQNEVEFCRHRTDGL**W**DEFGRFEVVRGKESRVKRRARAFGSGSYAAGGAGGGGGGGYATGGGGGGGGGGCCSCGIGAAGPAGPPGADGNPGTDGAAGNPGQAGADASQDAPSASDFCFDCPPGPAGPAGNAGPPGAPGGPGAPGNTPSGGAAGPPGPPGPPGGPGNDGQPGGPGNPGAPGAVTEVPGQQGPPGPPGPAGPPGPAGNAGQAGYAQPGPAGPPGDAGPDGAPGNPGQPGAPGDAGAPGSGGGCDHCPPPRTAPGY

>COL-86A

MDNKVKYLLNSIIFASSISVITAIFGCAYILSDISEFQSEILDNLNEFQYYSSDS**W**NVMLRGKTSGLRVKRQYQTAPVFGGARVEAGTCQCAQQSTGCPPGPPGPPGSEGHPGDSGVPGNPGQPGTAGISESHSSSGCITCPAGPMGSPGPDGPPGVPGATGNPGSLGESSLGAGSPGPPGGVGPPGPNGTPGAPGDEGIPGVPGANVICPPGPAGSMGSVGLPGPPGNDLGNSNGPPGPPGQMGQPGKPGSMGGLGQSGPTGSDGIPGIDGQYCHCPQRTHNLGMNRYHDEPIPVSKPKRNKDKMRKMKKFMRKLQKKFSVV

>COL-87

MPSSHYFKPLSYVSTVLLLTSMTCLIYFATDLQKFEEQCKSQISIFQIYEQAA**W**KDIITLERAHGRVRRQSLFDAPKLRRTRKSKLRKLKQPIRPALFHKPLVPVADELAELEQYRSSGNSGNSENSELTEADSDGETEQETEEANNPEASSSERDEADEDPEAPPEAPNHNMSRREMQYGITGGLAQILEVDGYGEGEEDEEDDDGSGSGGPEEDVEDPNECADTDRKCYGNPCPRGPPGSPGVPGLPGEDAADGTDGGNGTEWELLAADLVIILGCVKCPIGPRGVEGDPGIPGNKGGKGNNGNPGLDGYEGFPGPSGPEGEAGYDGEPGAGGWPGQRGENAKSYRNFPGPPGPPGPVGPPGDSGERGNIHEGGPPGIVGGSGAPGVHGIPGPPGAPGLPGDPGTRDDTYVGQCKCPERRQPPKKPYPPAPTMPPDAYQPTTEAYYYPPTETSTEYVTAEPTTSIYRYFVKAEAPSYKTTSSSSAEESEYMEITMTSSEYPMDTPTTVGYPVTEYPTTVVGYPVTDQMDREGQLLAEVLEKSMEPD

>COL-88

MSSNTILIGTQVILSTAILGSLLYAGVLYSEINELRDDVLQDMHSFRGLANDA**W**RSMVQANAPVNGGALPDFGSIFTRAKRSGSCNCGPQPSNCPAGPPGPPGAPGDAGLDGENGQAGPNGQDGAGQGSSQQPSECITCPAGAPGPQGPDGPAGAPGADGQAGSPGAPGQDGQPGGPGAQGDAGAPGNDGTPGQDGAPGQNGQRGNGAAGAPGPQGPAGNAGQAGQPGADGQPGAQGSAGIAGAPGQPGKAGEDGEAGENGTDGEPGTDAEYCQCPPRTGAIEVEPATQEEGYKRRKYRFSN

>COL-89

MEKFIVGVAATASTLACLVMVITIPQLYNTILEVHDEVLDGVSVFRIETDAA**W**TDMMDIQITVTPPSKPRVNPFNSIFRQKRQDFSGLPAWCQCEPAKPVCPPGPPGPPGEPGQPGSAGPPGPPGEDNTATFAPITCPPKDPSCVKCPEGPAGPPGPEGPAGPAGSDGQPGAPGDAGKDGPPGPPGPDGDSGPNGEAGPNGEPGAPGKDGEKGKGEPGPAGPPGPPGPGGPPGDAGGAGSDGAPGPQGPPGQDGTPGNAGPDGQPGSPGGPGLPGNDAAYCPCPPRSAVFVNRFAH

>COL-90

MSTIGYIGASICLLGVLSSLFSISHIVRDINSLRDEVEGRVDEFKVLADDT**W**QRLLVLQSPTGETENVMPSIFRSKRFVYPGMCNCDSNSQGCPAGPPGPPGLPGKRGDEGVVGDLGRSGASGISLAAVHHIPGGCINCPAGPAGPPGQQGPVGPQGFPGVVGTCGPSGDDGQPGPAGPLGDKGAQGPKGFDGADGPDGMPGTAYFPGAVGQPGEPGWLGQPGLPGKHGEPGQDGEEGPKGAPGTPGSNGRDAYPGQPGKAGEPGAVGKDANYCPCPARRDSKTESVHEPPAASQNGGYRKAKRH

>COL-91

MPSASTISVISAISGVAVLGALLTATIIFNDINRFYYETMETFDEFKINEKNA**W**DTMVMSTRSPKEMLLGRARRQAALPDCQALAKNCPPGPPGPPGAPGAAGEPGVDGDAGAAGIDGVAIQFASAAGGACIQCPAGEAGPAGAPGAPGPAGPDGQPGADGQGGAPGPAGPEGPAGDAGAPGAPGAPGNDGQPGQNGQRSTGTPGAAGAPGPQGPVGSDGQPGSAGAPGAPGPAGAPGVDGQPGANGQPGPDGEQGHDGQPGPDAAYCPCPARSSVRQ

>COL-92

MDEKQRLQAYRFVAYSAVTFSVVAVFSLCITLPLVYNYVDGIKTQINHEIKFCKHSARDIFAEVNHIRGSPKNASRFARQAGYGTDEAVETGNTGSQGGSCSGCCLPGAAGPAGTPGKPGRPGRPGAAGLPGNPGRPPAQPCEPITPPPCKPCPQGPAGAPGAPGPQGDAGAPGQAGQGSGAGAPGPAGPKGAPGAPGNPGQAGAPGQPGSDAQSESSPGAPGQAGPQGPPGPAGSPGAPGGPGQAGAPGPKGPSGAPGQPGADGNPGAPGQPGQSGGAGEKGICPKYCAI**DGGVFFEDGTRRK**

>COL-93

MEEQQRLQAYRFVAYSAVTFSVVAVFSLCITLPLVYNYVDGIKTQINHEIKFCKHSARDIFAEVNHIRGSPKNASRFARQAGYGTDEAVETGNTGSQGGSCSGCCLPGAAGPAGTPGKPGRPGRPGAAGLPGNPGRPPAQPCEPITPPPCKPCPQGPAGAPGAPGPQGDAGAPGQAGQGSGAGAPGPAGPKGAPGAPGNPGQAGAPGQPGSDAQSESSPGAPGQAGPQGPPGPAGSPGAPGGPGQAGAPGPKGPSGAPGQPGADGNPGAPGQPGQSGGAGEKGICPKYCAI**DGGVFFEDGTRRK**

>COL-94

MDEKQRLQAYRFVAYSAVTFSTVAVFSLCITLPLVYNYVDGIKTQINHEIKFCKHSARDIFAEVNHIRGSPKNASRFARQAGYGTDEAVETGNTGSQGGSCSGCCLPGAAGPAGTPGKPGRPGRPGAAGLPGNPGRPPAQPCEPITPPPCKPCPQGPAGAPGAPGPQGDAGAPGQAGQGSGAGAPGPAGPKGAPGAPGNPGQAGAPGQPGSDAQSESSPGAPGQAGPQGPPGPAGSPGAPGGPGQAGAPGPKGPSGAPGQPGADGNPGAPGQPGQSGGAGEKGICPKYCAI**DGGVFFEDGTRRK**

>COL-95

MEDITRRNAYRFVAYAAVTFSVVAVFSLCVTIPMVYNYVNTVKTQIDHQISFCKHSARDIYSEVNHIRENPKVNNTRFARQAGYDFDTGAVDSFASSGGSGGGGSCSGCCLPGPAGPSGAPGKPGRPGRPGAGGLPGNPGRPPAQPCHPITPPPCRPCPQGPPGAPGSPGPQGNAGAPGQPGHGGGSGAPGPAGPKGQRGAPGHPGQAGSPGRPGVDAQSQSNPGAPGQAGPQGPPGPSGAPGAPGGPGYPGPPGQKGPSGAPGQPGSDGNPGAPGQPGQSGGSGNRGICPKYCAI**DGGVFFEDGTRRK**

>COL-96

MDEITRRNAYRFVAYSAVTFSVVAVFSLCITLPMVYNYVHGIKSQINHQISFCKHSARDIFSEVNHIRASPNNATLREKRQAGDCSGCCLPGAAGPAGTPGKPGRPGRPGAAGLPGNPGRPPAQPCDPITPPPCRPCPQGPPGAPGSPGPQGDAGAPGQPGFGSGFGAPGPAGPKGQRGAPGHPGQAGAPGQPGVDAQSQSTPGAPGQAGPQGPPGSSGAPGAPGGPGFPGAPGSKGPSGAPGQPGANGNPGAPGQPGQSGGSGERGICPKYCAI**DGGVFFDDGSFRRR**

>COL-97

MEEDSREKAFRFVAYSAVTFSLIALVSVFVTLPLVNNYIHSVHLRVHDEMQFCKLSARDVMLEMHNFRQTPNKNLPFFLRRSSNETTRLKRDAAACQGCCLPGLPGPDGPPGKNGAPGRPGAPGAPGFPGRPPAVCEEITEPPCTPCPPGEPGPPGPPGDAGNPGQSGHPGRNGNDGGVGPQGPPGPPGNNGEGGRDGPRGEQGRPAISTPALPGDPGAPGEPGPSGLPGDQGQAGRPGSDGAPGPQGPPGPPGQQGHPGQAGPAGQPGQPGPQGERGICPKYCAL**DGGVFFEDGTRR**

>COL-98

MDIDVRLKAYRLLAFSAVAFCVLSVVSVCITLPMVYNYVSGMRVRLVQEVSFCKHSANEVFTEVNHLRASATNSTRSARHAGYGGYAQPQGGGGGGGGQCSTCCRPGPPGAGGTPGKPGRPGAPGAWGMPGNPGKGGSGPCHPVVPTPCKPCPGGRPGPPGPPGRPGSDGQPGRPATGGGAAPRPGPPGPKGPRGAPGNSGRAGAPGQPGNDAHGYGGGVGAPGPAGPRGAPGPAGHPGSSGGGRPGPAGPKGAPGQPGRPGPDGHPGQPGRPGQSGGSGNRGVCPKYCAI**DGGVFFEDGTVRRK**

>COL-101A

MVLDFVVQSPTVIMTSDDPKQVSYETESLRKVAFFGIAVSTIATLIAIVAVPMLYNYIQHVQSSLQSEVEFCAHRSNGL**W**DEYKRFEGVAGVEGRIKRNVYERKYHAKQRRQSYGDSGASGGFSSGGSGGGSCCSCGVGSAGPAGSPGQDGAPGNDGAPGAPGNPGQDAQDNQPAGPDSFCFDCPAGPPGPSGAPGQKGPSGAPGQPGRSGGASLPGPPGPAGPPGPSGQPGSNGNAGAPGAPGHVVDVPGTPGPAGPPGPAGPAGAPGQPGQAGHAQPGQPGPQGDAGAPGAPGQPGSAGQPGNDGGDGGQGACDHCPPPRTAPGY

>COL-102

MTQAVLGVVNTVTAVSVITAILLSVAILVDINNFSDEVSQDLTQFRGYYDDA**W**RTMMVNEGMKGLTARISRQAGYATGGSQQCNCGSQAAGCPAGPPGAAGEDGTPGEPGTDGEDGGNGMPGVAQMAMDANGGACIKCPAGEPGPPGPEGPAGPAGPAGNAGADGEAGAEGPAGEAGPPGPPGPDGKPGAAGSDGEAGAPGTKTTNAPGEPGPAGPPGPPGPNGEAGAGSGPGPEGPAGPPGPNGKDGEAGAPGKDGDAGADGAPGTDAAYCPCPPRSAALGAGGGAEPAGAAPEAAAAAPEAAPEAAPAAEGAGGGAEPAGAAAPDAAAAAPEAAPAEAAPAAEGAGGGAEPAGAAPDAAAAAPEAAPAEAAPAAEGAGGGAEPAGAAAPDAAAAAPEAAAAPEAAPAAEGAGGGAEPAGAAPEAAAAAPEGAGGGEAPPTNGKGGEASPAYPTGQSSY

>COL-103

MSASIKFATGAAVLSGVTILACLFFAAQVFNDVNSLYDEVMVDMDAFKVKSNIA**W**EAINDVIAPNREKRGYAQYGGGGGYGGGHGGAAVGGGYGGAVGGGGGGGYGGGHGGGHGGAVGGGYGGGGGGGGGCQCSPSSNTCPPGPRGPPGQAGLDGLPGAPGQPGSNGGAGSNGASEGSAGGCKTCPAGPPGPPGPAGQAGRPGNDGQPGAPSFGGGVGAPGAPGPAGDAGSPGQPGAPGQPGRPGKNAQGGSSRPGPPGPAGPPGPPGNNGAPGGGYGVGPPGPPGPSGRPGAPGQPGPDGQPGAPGNDGTPGTDAAYCPCPGRGGGGGGYGAGAHHGGPQGFSRRKVARKVLAKKRVAVQRKAAAAARHA

>COL-104

MSARVAGYAAIAFSLSSLFLVSTFVPALWNKINDITGELHYDMNEFRDLHNEV**W**ANMRGETFGESVVPFARKKRQTTRPGECVCNSNSACPPGEAGPQGIPGHDGTPGLAGDAGAPGLPGNYPSINMDAHQQCRMCPPGAPGFPGPAGPPGAPGDKGNDGAPGLRGPDGHPGHPGNPGYGGEPGAPGNNGEPGDKGRDGSHGTKGAPGPQGPSGPVGQPGFPGQPGNDGNSGVPGIQGSSGGPGEDGAPGAPGFDGQQGPSGTPGEDANYCKCPPRSKLSR

>COL-105

MFFQVKAYRFIAYSAISFSFVAILSVCLSLPMIYYYINNAKTSLQGEIEFCRNSARDIYGEVQQFKQIPRNRTTRQAYGSLLDTDECDGCCIQGAPGPTGEPGRPGRPGKPGVPGMPGNPGKPPQQPCELVTPPPCSPCPAGPPGKPGPQGAPGDIGQPGNPGERGMDGEPGPPGPTGPPGEPGLPGAQGITGQPGVNAEPVAYRAGAPGPPGEPGPPGAHGDPGDLGEEGPPGLPGPKGPPGASGIPGNDGPPGARGQPGLSGDSGEKGICPKYCAI**DGGVFFEDGSRR**

>COL-106

MGAFGTLACASATVLTIGSLATMCLIVSDINGLYNDVMEESRTTREVADDA**W**EELLRKNGAPEASRAAVTAMLFGRNKRNAQCNCGAKSKGCPAGPPGPRGPNGAPGLDGPDGLDGPDGHPGYAAAIKENFGGACISCPAGPAGPPGPDGCAGEPGTDGAPGEDGQNGAPGSPGGPGETGPQGHQGKPGRAGPRGPPGAKGQTYLPGSAGHPGNSGRRGPPGPPGANGKNGKQGADGIPGAGGAPGQHGVPGVDGSDGAPGEDAAPGGDAAYCPCPTRSVAVEAAPSQAEQIYKTH

>COL-107B

MSTKAIVIGASFFSGVTIIVSLVAAGMLLQDINDLYYDVMDDMTEFRNTANEA**W**KDMIVPSGAVDASLIFGRNKRSGGTCGCGAQPNNCPAGPAGPPGAPGAPGEDGTPGQAGKNGGNGIGIVNSADAGGCIKCPAGEPGPAGPDGPAGAPGADGQPGAPGNAGQDGAPGAAGEQGDAGAAGQDGEAGAPGGPGKNGQRGSGAPGPAGPEGPAGPAGQDGAPGQDGAPGNAGSEGPAGAPGKDGEAGAPGQDGEAGGEGIPGQDAAYCPCPPRTAAVEATPASQGGYRRRFSHV

>COL-108

MDLEQRIKAYRFVAYSAVAFSVVAVISVSVTLPMVYNYVHHVKRSMQTEINFCKGSAKDI**W**TEVNHLKSIPASRNRTARQAGYDAGVNGGGSEASGAPGNCEACCLPGAAGPAGTPGKPGRPGKAGAPGLPGNPGRPPQQPCEPITPPPCKPCPQGPAGPPGPPGPAGDAGSDGSPGAPGSDGQPGAAGDKGPAGPNGNPGAPGSPGKPGEDGKSEPITPGAPGPAGPPGPQGPPGAPGQPGHDGQPGTPGPKGPNGNPGAPGADGNPGTPGVAGVAGSGGEKGVCPKYCAI**DGGVFFEDGSRR**

>COL-109

MEDTREKAYKAVTYSAVTFSFLAILSVCISMPIIYNFVDSIHQQTKRDMTFCKSTARDIMSEISHKKPIAAAIAGNLTTIRNKRQAVGCAGCCKPGHPGRPGLPGRNGKPGVPGAPGRPGTPGRPPIVCEEQDVPPCNPCPPGPPGPQGPTGNSGQPGRPGNPGRPGSPGTPGPVGPNGASGDSGAPGNDGEKGEPGRPAQSTPSTPGEPGNPGDAGATGAPGDDGAPGRDGQPGQSGPPGPPGPPGNVGEAGPPGKPGQPGLPGPQGERGICPKYCAL**DGGVFFEDGTRR**

>COL-110

MSSEYSHLRIQRFIAFSVAIVSLVLTVGAMVTLPLAYHYVNQLRNSLERQFVECNDVVRTIYEGVDELSVLIATSNHTRSIRGAKPRRRFDEDETTGTCDGCCIPGAPGPSGTPGRHGRHGAPGAPGRPGNPGEPPRGPCDPTLASPCDCPAGEPGEPGPRGSPGNNGLPGLKGRRGFDGLDGRPGRPGKTGIDGVPGTKGEDGADGEPGEVDTSDVGPPGDDGDEGQSGEAGQPGNDGRDGKPGQVGPPGPPGKDGVDGENGNDGDLGEAGIQGRPGEEGICPKYCAI**DGGVFFDSGSRRRRT**

>COL-111A

MTVDNAAIATWTSVLVSVACISFCAFFAASVCEDINQFLDDSLLSFKEGQGRTDDA**W**NEVVFLNDKKFKSELLVSNSATVRQKRSDLPAHCNCLSQNQCPPGPPGPPGPAGMDGKPGEPGASGPHGMSGSELTKMMRNDDTCIKCPAGPPGPRGAPGSVGEPGPQGRDGMGGRPGNMGRPGPPGPAGDPGQAGSKGAPGTHGRQGMPGVRYQLGEPGPAGYPGPRGAPGPVGPPGVAEEGPDGLPGPAGPPGKPGMPGPMGTPGGPGEPGIPGSDAAYCPCPKREALVESSVISQEVPETGYGDGGDKSKREKVVETHQEEQTYDSFKFEQV

>COL-113A

MKSLSTQRAGFTACVFSGGAILVCLVYVPSLVTKIQNINDQLKVDSEEFRAMADYT**W**RELITMRRGKRQAYGDSPKKTYPLHTAYVKEDAFVEGASTCSCNQNNGCPAGQAGAAGKPGLDGEPGKSGEPGAPGLAGIAPPVTIDPTKGCRVCPNGPRGPTGPPGETGPSGPEGPPGNPGRIGEHGREGYPGQQGIPGEPGKPGKLGEAGPNGRDGTRGQKGPIGPKGDSGPPGQKGPSGYPGRDGQRGNDGESGPVGPSGQLGMPGEPGQIGMVGAPGAPGQDASYCKCPERNAGVNKFEPPPIEQPSYEPQPSTSQPATYAPAPATPGPDAAVEAPRSPYRKWKWLQ

>COL-114

MEDESCQRIKAYQWVANIAMCFAIVSVLTVCVSMPAIYMYMFHVKQLINTEVVSCEDHAREI**W**REVEIMKNLPFTNRTSRFPRQAAYKDAAPAGGGGGGEKYSEAPPVIEAAGGGSCGGCCPGGPGPNGKPGKPGRPGKPGAPGLPGVPGKTTIQAPCEAASPPPCRVCPVGMPGEQGVTGAPGDKGPAGQPGRNGPDGPNGEPGPKGPPGQPGADGQPGMPGEPGKDAPQEGSPVPGDPGPPGDSGIPGPPGPPGMAGPDGIPGMGGAKGQNGPDGPPGASGESGPNGPPGSGGGAGEKGICPKYCAI**DGGVFFEDGTRR**

>COL-115

MKGNEKDLELLARRMRQLAVFSSSVAVFVAVLAVFALPILITSTLQAVTSVDDSLGRCTADAFKMYKAIDEIEIQLIQSFNKTTSQRSKRGGGYVSSSSYGGQYSGQQYDSTGNAATNGQYRFFPEVIEAIRARRPTLYSLQQDYNSGGYAGQGGCGASQQGYASRPVFVGPQTGSILRDGQSGFNGGGGCIPRYGPPGAPGASGQSGRDGQDGQPGTDGQQGRDGIADIDREPCQVCAPAPQGYPGPPGPKGRTGEQGPPGLDGETIDGEDGSPGLPGPPGPPGPPGLHGSPGSYGESKEEEIAGPPGPPGVRGIQGGVGSRGAEGNPGPKGPPGLQGDIGSHGIAGRPGLQGRPGNPGAPGSPASCDHCAPPVLEPGYQVDVKKTIKRASP

>COL-116

MIRLATFIIVISAVSIFGALCVAASILVGINEFHTIVSEDLRDFKGYFGNA**W**KTMHVSEGNPLRILARRDASKMARERKQAGYEFDGGASVEAHIQQCNCAPATSHCPPGPPGIFGPPGEDGTPGEPGLDGDPGTTATRQMAMDYTGGSECVKCPAGPPGAPGPNGPPGYEGQMGVPGLPGDGPTPGSPGPPGPPGPPGYPGRQGCQGKDGPSGDPGLRFIKTPGPVGAAGPPGPPGPPGSPGENTEGAPGLDGPLGPPGKPGTPGTDGAPGVFGDDGVPGQDAQYCQCPPRTPIGSADADSEDNGGYFFFKS

>COL-117

MDLEQRIKAYRFVAYSAVAFSVVAVISVCVTLPMVYNYVHHVKRTMHSEINFCKGSAKDI**W**SEVNHLKAIPSGNRTARQAGYDAGVTGGSASAGGCDACCLPGAAGPAGTPGKPGRPGKPGAPGLPGNPGRPPQQPCEPITPPPCKPCPAGPAGAPGPAGPAGDAGANGNPGAPGNDGQPGAPGNKGPSGPNGNPGAPGAPGQPGQDAPSEPITPGAPGPAGPAGPQGPPGAPGQPGHDGQPGAPGPKGPNGNPGAPGADGNPGAPGQAGTSGGAGEKGICPKYCAI**DGGVFFEDGTRR**

>COL-118, isoform a

MMKLVVSGFAALSGFAILALTIFVPQIYWEMSDLQDQVVGVVESFKVETDSL**W**IDLMDVQIRHTAPSRPLENPLLRRDKRFTKLPDWCQCDSAPQCPPGPPGPQGAPGAPGTPGENGPRGMDNPHSLGSTSCDQQSIGCVQCPRGPPGPPGDVGSVGQRGPDGASGAPGAASQIHGKPGLPGPPGDAGAPGAPGQDGAPGHPGADGQRQRGQPGPPGPPGRPGKVGINGEPGQKGIDGENGSEGIPGVPGPRGAPGQPGTIGVPGGPGIPGRDGQYCPCPPRSFILAANSRRLRSNV

>COL-119

MDIDSKIKAYRFVGYAALTFSTVAVLSVCITLPMVYNYIHHARKTMHHDIVECRAEAKKL**W**GDVNRVPDMVMAHNRTARQVGNGQCEGCCLPGAQGPPGTPGRAGRPGKPGAPGLNGNPGRPPKEPCEPITPPPCKPCPEGPPGPAGPPGPQGNKGPLGPPGPPGPEGPNGTAGNKGPAGPPGPGGKPGPAGPPGENGRNGEPQPGSPGEPGRPGQPGSRGPAGQPGKDGAQGGPGEKGANGEPGQPGRDGQPGRPGQPGRDGHPGEKGVCPKYCAL**DGGVFFEDGSRR**

>COL-120

MNLKKNLVNSEEDELRKVAFVATVVSAASGIMCIILIPGLYTYLQYIQSSVQTDVGFCVDGAKNLENMYESTKAVGSGPVKRQAGYGASSPSRASGSHPAPSPYDAASTSSSSSSDSCCSCGIGLAGPAGFPGRPGRDGIDGPAGKPGRDGQDLDGESSSDGSQIELDCPAGPPGPPGNPGPQGNSGRPGMDGMPGRNGRCGRPGEQGERGPNGEDGRPGRRGDDGMPGTVNEIPGQAGPPGLRGAPGATGSQGPRGNDGRPGNKGPAGPPGDQGFDGAPGGPGADGEPGAQGPLGAKGECSHCPPPRTAPGY

>COL-121

MAVDVARLGARLICLVSALTVGFTLITFIHISSKIQKTENEIIGNQSAFKAKSQVI**W**AELMETGRSLRIPRSSYGTDEKKGVLHQCSQCTRLHCPQGPIGEEGSPGNDGQPGTPGKPGSPGLEGVDVDLEPQEELPCSICPAGPQGPRGAQGERGLPGNAGRKGKSGKSGHPGQPGGIGASGAKGPTGTRGTPGMKGPPGDTIIAGEGIKGPPGLQGSQGPKGPPGNSGRSSNIRGPSGKPGQQGGIGGTGKFGKYGEQGGIGAPGEPGLPATYCPSDCGVNTILSQFGSRAQHPESINGEDNGAPAANTEEYNSEERIESPAPPAPQPTTNQQQETVAAAASEQESFEESSYRSFFGH

>COL-122

MEDNKIKAYRLIGYAAVSLSAVGILSVCITLPLVYSYVSNLRSQMHSELRHCRDTVNTVYATAVAMPALPGRGNRTARQAGYDVAVQDAPAQEHNSCDGCCLPGAAGSVGAAGRPGTPGRPGAPGAPGHPGKPPQSPCEPLTPPPCPACPPGPPGAPGAPGKPGSEGPPGPPGHDGPNGGPGQPGQPGPHGPNGHPGKPGSQGPPGPPGHSDEPKPGQPGQPGRAGPRGPRGVAGIKGKDGAPGSPGQPGPRGGPGEPGQDGAPGQPGLPGSDGTPGEKGICPKYCAI**DGGVFFEDGSRR**

>COL-123

MGIFSEFLYRMQVSSVTFVASSLSAIALISCLLVLSSVYRNVQDY**W**TELDDQMVEVRKDLDDM**W**REIMHIQSNSLGSVFRARRPARGAYDEGDYKEAPPPTSYPSSGGYDEPSAPAHDTAPQAPAGPAPTSPDSCQCQAENKCKAGPQGAKGAPGLPGPNGVPGLDGKPGLDSLDVEPQAQGTGTCFHCPQGPPGSPGPMGHPGARGMKGSDGQPGQPGKDGDPGKPGEMGPNGFQGKVGPDGFPGEKGGDGRRLIGRPGPKGQKGETGHPGAQGTPGHNGPTGPPGDAGPDGGPGPQGEQGADGGPGSEGKVGRPGRDAEYCHCPARGSHGGSSGGGGGGGGEYKATGGDSYRKKKL

>COL-124

MDIDSRIKAYRFVGYAALTFSTIAVLSVCITLPMMYNYIHHTRKVMHSDIVECKSEAQRLFSQVNRIPDLMMAHNRTARQAGEGNGQCEGCCLPGAQGPPGTPGSAGRPGKPGAPGLNGNPGRPPKEPCEPLTPPPCKPCPEGPPGPAGPPGPDGNKGPLGPPGPPGPEGPNGEPGNKGPAGPPGPGGKPGPAGPPGENGNNGEPQPGAPGEPGQPGQPGQRGPAGEPGKDGSPGGQGEKGASGPGQPGRDGQPGHPGQPGKDGRPGEKGVCPKYCAL**DGGVFFEDGHRR**

>COL-125

MDLEQRIKAYRFVAYSAVAFSVVAVISVCVTLPMVYNYVHHVKRTMHSEINFCKGSAKDI**W**SEVNQLKSIPSGNRTARQAGYDAGVTGGAASAGGCDACCLPGAAGPAGTPGKPGRPGKPGAPGLPGNPGRPPQQPCEPITPPPCKPCPAGPAGAPGPAGPAGDAGANGNPGAPGQDGQPGAPGNKGPSGPNGNPGAPGAPGQPGQDAPSEPITPGAPGPAGPAGPQGQPGAPGQPGHDGQPGAPGPKGPNGNPGAPGADGNPGAPGQAGTAGGAGEKGICPKYCAI**DGGVFFEDGTRR**

>COL-126

MEKILVTISTGAASIAVLAVLFTVPSLYNTINEVHDQVLDGVSVFRVETDSA**W**TEMMDIQVTVTPPTKPRVNPFNSVFRQKRQTFSGLPAWCQCEPTKPTCPPGPPGPPGQPGAPGVPGAPGPRGGDSTVTYAPINCPQVSFDCIKCPAGPAGPAGSSGPAGPAGPSGQPGTPGQRGNDGFPGAPGAPGDNGQPGFPGQDGAPGQPGADGQRGAGTPGAPGAPGNAGPAGPSGQDGFPGQDGAPGSAGPAGQDGFPGNNGSDGQPGAPGGPGLPGNDAAYCACPPRSAVFLSRH

>COL-127

MEKILVTISTGAASIAVLAVLFTVPSLYNTINEVHDQVLDGVSVFRVETDSA**W**TEMMDIQVTVTPPTKPRVNPFNSVFRQKRQTFSGLPAWCQCEPTKPTCPPGPPGPPGQPGAPGVPGAPGPRGGDSTVTYAPINCPQVSFDCIKCPAGPAGPAGSSGPAGPAGPSGQPGTPGQRGNDGFPGAPGAPGDNGQPGFPGQDGAPGQPGADGQRGAGTPGAPGAPGNAGPAGPSGQDGFPGQDGAPGSAGPAGQDGFPGNNGSDGQPGAPGGPGLPGNDAAYCACPPRSAVFLSRH

>COL-128

MWLYTLAALSISFSCLLIIVQVIYFPYLISQAESAHGIVMSKIESFKNLESEITRELSDFTFLHVARKREIISCEPGYPGLPGSNGRDGQAGNDGPNGIRGLDARDIMEEIQANQECITCPQGERGPTGAPGDRGETGDKGDKGVAGIPGQDGLDGEQGEEGPVGPSGPPGRRGRKGADGKSAMGGFGSPGPKGVPGAKGGSGLQGPRGKRNYIYGPPGPMGQPGPSGLDGVSGNPGERGPKGPPGDRGADIKFCPCPLELELLKGKHSKSVRPSPPASPSSTSQPTTSATTTSEPLSPSTTIVTDAPELVTTETSTEDLAVMEDITATTTMETITFASQDLGKEEIREPENVFGRAPGAETGPQGGNEIEVLNYDDEEIRTIELPKEYEHEETRGPIITEEEDDYPEETTTTRRFRYVTKRPIRFE

>COL-129A

MSTARYLVSIAAAASTFAVLASIVSLVYLVNDVNNYYDDVTRNIENFKDDANTV**W**HQMMTVVPQGGKQRQVYESLFGRTKRQAAQCNCGANSAASNCPAGPPGPPGAPGEDGDHGLVGEDGTPGADGNLGVPLSANGCIVCPAGPPGPPGQPGQNGQRGSDGQPGQDAMGGGQGAPGPQGPAGDNGQPGRPGNDGRPGAPGAPGTRSVGRPGTAGSPGPQGPPGQNGNDGQPGNDGAPGQPGQPGPDGHPGQPGNAGPPGTPGAAGQPGGDAAYCPCPSRSSVVRAQAAVARNRHVAARHRSVSRRKAVAARRSKARRVVAAKHRAVAKRHRAVQKA

>COL-130

MSAKYVVTIASCFSGLAIVACLFTVGAIFKDINDLYDNVMDDMDEFKMFANNA**W**KDMIPVTRPSLDNQSNLRAIFGREKRQAGQCNCGAQPNNCPPGPPGPPGAPGAPGDDGHAGEAGKTGINGISLISHEGESGCIKCPAGEAGPAGPDGAPGAPGPDGQPGQDGPAGQPGAPGPAGPEGDAGAPGDAGAPGAPGAPGQDGQRGTGLPGAPGAPGPQGPSGNPGQDGAAGAPGQAGPAGPAGPDGQPGQAGQDGEAGPEGNAGQPGADAAYCPCPARTGAVENKPETSGYRRRVSKVV

>COL-131

MSSEILVIGTSLFVFLSVLVFVLQTKNDLDQVWAEFDQEVQEVTVLREAS**W**NTLRNLATAKKIPIERSKRQSYYEKVADSYALPPSLGVEAHAPSISPECNCNLENNNCPAGPSGPKGLPGQDGAPGEPGPAGKMGQNTGDVQTMHVDPGCQYCPAGEMGPPGAPGKIGPRGQRGQSGGAGTPGNNGAPGHNGELGPCGQAGPPGPAGAIGRRGMDTVREKGVRGPIGAPGPVGPVGMEGDRGNRGGLGVDGPLGSIGLQGPPGRTGSTGDVGESGPIGPNGQDALYCPCPKRVADGAGGGVYQPYKQ

>COL-132

MSNNFICAAVSVSSILVVGYITAGLLIFADFRTFYSTALDDVAEFNKFATEA**W**DDMIEINRSVVRQKRSGESKCGCMNKVNNCPPGPAGPPGEPGAPGDDGWNGDAGQPGPDFPLFDQSLIYQRPPCIRCPVGEPGPPGPDGEPGEQGPPGPPGVDNFEVGHDGPPGVEGEPGDAGDQGEDGEPGVDGRPGASGIREIGMPGAPGAPGVAGAEGLPGKDGEPGIPGPEGEEGPPGAAGLDGERGDEGEPGPEGPPGIPGPDAAYCSCPLRSARLVF

>COL-133

MDEKQRLQAYRFVAYSAVTFSVVAVFSLCITLPLVYNYVDGIKTQINHEIKFCKHSARDIFAEVNHIRSSPKNASRFARQAGYGGDEGVDQGSEGAQGGSCSGCCLPGAAGPAGTPGKPGRPGRPGAAGLPGNPGRPPAQPCEPITPPPCKPCPQGPAGAPGAPGPQGDAGAPGAPGQGSGAGAPGPAGPKGASGAPGNPGQAGAPGQPGADAQSESIPGAPGQAGPQGPPGPAGSPGAPGGPGQPGAPGQKGPSGAPGQPGADGNPGAPGQPGQAGGAGEKGICPKYCAI**DGGVFFEDGTRRK**

>COL-137

MEDSKKVAAETESFRKIAFAGIAISTIATLTAILAIPMLYNYMQHVSSSLQTEVDFCQHRTDGL**W**DEYKNFQNLQGVTGRLRRSTNKRHTGQMNRSKKMRRQNEEEEVEIVYVDEDGNEVDDQTPTAAPVAQAQVPASSFAATFSHASRAPASSGSCCSCGVGAVGPPGAPGQDGAPGNDGSSGAPGHPGQDAVDDSQAGPDSFCFDCPAGPPGPSGAPGQKGPSGAPGAPGQSGGSALPGPPGPAGSPGRSGQPGSNGNVGAPGAPGQVVDVPGAPGPAGPPGPPGPSGAPGHPGQAGSSLPGAPGPQGDAGAPGQPGAPGQQGEPGEDGESGSEGACDHCPPPRTAPGY

>COL-138

MDEDRQSVKKFAFFGIAASTVATLTVVMAVPMLCLYLQNIQSGLQDDLTFCKSRTDSLRGEYTRLAAYRDSAVATLRQKRSTNEKCCSCGTGTAGPVGAPGDDGAPGSDGKAGKPGVNGKDADAQQIPTADDFCFECEPSPVGPPGPPGPKGPDGESGAPGEPGPAGRPGNKGAPGPAGPVGADGEPGELGAPGAPGVQRTVASPVGEPGEPGEQGPKGEAGQDGRPGQPGRQGPQGEPGQNGKDGEPGKDGVDGEPGKPGEDGPKGSCDHCPPPRTAPGY

>COL-139

MELQTRLKAYRFIAYSAVTLSVAAVFGICFTLPLLHNYVEGMKSHVDKELAQCRHTTADIFSEMAHIKKAKNGTRFARQAGYGGDAEYEDDAAVEVAPSRVSGGSCQGCCLPGPPGTPGPAGRPGKPGRPGACGNHGNPGKPTGLPCDPVTVPPCKPCPPGPAGEPGHDGAPGAPGKPGAPGVGGGSGAPGAPGPKGAPGGPGQPGRDGQPGQAGQPGSSSSEPGQPGPNGQPGPRGPPGQAGSPGGNGQPGGPGQPGQRGSDGQPGNDGQPGAPGQPGQSGGSGEKGICPKYCAI**DGGVFFEDGTRRRR**

>COL-140A

MGKLILVGSSIALTCCLVTVFTMGSLLQEISDMKMEVEDGMMEFREIHQDT**W**NRVVRKHINPTGETDAPPNFQTLFGIRRSRQSGFPEQCNCGPRSEGCPPGPPGTPGEQGPQGESGPDGDDGKPGLAGEVVAIVHDLPGGCIQCPPGRPGPRGPPGEVGPAGPSGDNGRKGPPGPPGGPGGPGEGGDGGRPGNPGRPGPAGPRGEPGTEYKPGQPGRPGPPGPRGEAGPAGQPGSPGNDGESGKPGNAGRPGPPGAPGKNGTPGVRGEDAAPGPDAGYCPCPARAYKA

>COL-141

MHTSSNTMSTSTFVSVASAISGLAVLGCLFTVAMIFNDINSFYDEKIEELNEFKGLEQNA**W**RSMIPSTRPVNAESLLLGRNKRQAQCNCGAQSQGCPAGPPGPPGYPGAQGEPGLPGIAGQPGSGARVNPQTGRDSFCITCPAGPPGPAGTPGAPGAPGNAGAPGQDSFGGGPGPAGPTGPTGDAGAPGQPGHPGSPGNPGRGGQRSRGLPGPSGRPGPQGPAGGPGQPGQSGGSGTPGQPGAPGAPGHPGQAGTPGTPGAPGNSGAPGGDAAYCPCPARSAAMAARKRVFKVARSRAAVAKRRVVKRVRVQKA

>COL-142

MSASTLVTVASAASGIAIVVCVFTVGMIFNDINSFYDEKIGELKEFKGYEQIA**W**QAMIPTTRPSSGSSFLLGRNKRQAQCNCGAQSRGCPAGPPGPPGQPGAPGEQGHPGLAGQPGSGARINPATGRPGFCITCPAGAPGPAGPPGAPGPKGNNGQPGAPAQSGGRGPPGPRGPAGDAGSPGQPGHPGSPGNPGRGGQRSRGLPGPSGRPGPPGPAGGPGQPGHSGGAGSPGPQGPPGPSGQPGHSGNDGVPGAPGNPGSPGGDAAYCPCPARSAAIAARSRVAARSRVAARSRVVARSRVVARNRVAAKRRAAVRRHRVQKAVA

>COL-143

MSASTLVTVASAASGIAIVVCVFTVGMIFNDINSFYDEKIGELKEFKGYEQIA**W**QAMIPTTRPSSGSSFLLGRNKRQAECNCGEQSRGCPAGPPGPPGQPGARGEAGLPGIAGQPGSGARINPATGRPGFCITCPAGAPGPAGPPGAPGPKGNNGQPGAPAQSGGRGPPGPRGPAGDAGSPGQPGHPGSPGNPGRGGQRSRGTPGASGRPGPQGPAGAPGQPGRSGGAGTPGPQGPPGPSGQPGHSGNDGVPGTPGNPGAPGGDAAYCPCPARSSVMAVNRRVAVNRNVARNRVVVRHRIAARRRVAVQRRARVHAQAV

>COL-144

MEKLLVTASATAATFAVFAVLFTVPSLYNTINEVHDQVLDGVSVFRVETDSA**W**TEMMDIQITVTPPTKPRVNPFNSIFRQKRQTFSGLPAWCQCEPTKPTCPPGPPGPPGQPGAPGTPGAPGPKGDDNTATFAPLTCAPVSQDCVKCPEGPAGPAGPEGPAGPAGPDGQPGAPGNAGNPGSDGQPGAPGDNGQDGAPGQDGQPGAPGQDGQRGSGAPGGPGAPGNAGPAGPAGQDGAPGQDGQPGPAGPAGQDGAPGNAGSDGQPGAPGGPGLPGNDAAYCACPPRSAVFVSRH

>COL-145

MEKILVTFSTGAASIAVLAVLFTVPSLYNTINEVHDQVLDGVSVFRVETDSA**W**TEMMDIQITVTPPTKPRVNPFNSVFRQKRQTFSGLPAWCQCEPTKPTCPPGPPGPPGQPGAPGTPGAPGPKGDDNTATFAPLTCAPVSQDCVKCPQGPAGPAGPSGPAGPAGPDGQPGFPGQRGNDGFPGAPGAPGDNGQPGTPGQDGFPGQPGADGQRGSGAPGAPGAPGNAGPAGPAGQDGFPGQDGAPGPAGPAGQDGFPGNAGSDGQPGAPGGPGLPGNDAAYCACPPRSAVFLSRH

>COL-146

MVKTLVTIASIGSGVAVVACLFTVGYIFNDINSFHDEVIDTMTEFKFNEQQA**W**NSMMKPTDVFGRIKRGINKRGAQCNCGAQSNGCPAGPPGPPGQPGAQGEAGHAGEAGKPGANGVTIGLTGGNGPCITCPAGAPGPAGAPGAPGPQGPSGAPGQDAVGEGPGPAGPQGPAGDAGAPGQAGAPGHPGAPGQGGQRSRGTPGPAGAPGPQGPAGGPGQPGQSGGAGAPGPAGAPGAPGGPGQPGQDGQPGAPGNDGAPGGDAAYCPCPARSQVVKHRKVMAHRRHAKIVA

>COL-147

MVQTLVTIASIGSGVAVVACLFTVGYIFNDINSFHDEVIDTMTEFKFNEQQA**W**NSMMKPTDVFGRIKRGINKRGAQCNCGAQSNGCPAGPPGPPGQPGAQGEAGHAGEAGKPGANGVTIGLTGGNGPCITCPAGAPGPAGAPGAPGPQGPSGAPGQDAVGGGPGPAGPQGPAGDAGAPGQAGAPGHPGAPGQGGQRSRGTPGPSGAPGPQGPAGGPGQPGQSGGAGAPGPAGAPGAPGGPGNAGTPGTPGAPGNAGAPGGDAAYCPCPARASAVRKHRKVFRRRHAKVSA

>COL-148

MFEEKLIVGISTAISALAVFGWFFVIPSLYNTISAIHDEVSSGVSLFRDETDLA**W**TELMDVQISVSPPSYPRDHPFSSIIRQKRQSFASLPEWCQCEPTKPICPPGPPGQPGQQGQKGPPGVPGQRGQDCTIVYAPIICPPVSQECVKCPPGPVGAIGPMGPIGSMGRAGQPGYSGPRGNDGFPGLPGPPGENGQPGQSGKDGQPGRNGADGKRGYGAPGAPGAQGKPGPSGPPGIDGYPGQNGWKGQQGVPGQDDFPGTFGEPGAAGKQGYPGLPGSDASYCPCPNRSANFHTK

>COL-149

MFEEKLLVGIASLASTIAILTCVVVIPGLYSTINEMHDEVLDGVKMFRADTDAA**W**VEMLDVQVMVSPPSQPKENPFNSVFRQKRRSTFSGLPAWCQCEPTKPKCPRGPPGPPGHPGQRGIPGIPGRNGQDNYNTIRAPACPPRNQDCIKCPAGPPGPSGTCGQVGRPGPDGRPGQPGRRGNDGRPGQPGPQGNAGQPGRDGNPGQPGHPGKDGRRGHGSPGAPGRAGQPGRQGAPGNPGRPGERGPSGPCGPAGRSGQPGNRGSDGHPGAPGNPGLQGSDAAYCACPTRSVMFLKRH

>COL-150

MFEEKLLVGIASLASTVAVIVCLVVIPSLYSTINEVHEEVLDGVKIFRAETDAA**W**VEMLDVQVMVSPPSKPKENPFNSVFRQKRQTFSGLPSWCQCEPAKPRCPRGPPGPPGRPGQRGTSGSPGPRGQDNHQVHAPIQCPRQQPGCVKCPRGPAGPTGSIGQAGRPGPDGRPGQPGRKGNNGRPGQPAPQGNAGHPGRDGEPGQPGNPGKDGRKGRGHPGTAGRPGQPGKQGSAGNPGRKGERGQDGQPGPGGRPGQPGNRGSDGHPGTPGGRGLPGPDAAYCACPPRSSVFVKARH

>COL-151

MKHTNFLFATILLIVFAVLTILASIFSVFNEIENFEITSLEDLKTFEKYSDDA**W**NLITESIREKRAVYRIKLPPRHGYGQPRYVTIPPTNSYAVPEVGGSMPLICPEGPPGRPGHPGHPGEDGSAGQPGSPGGVGVLLSMKLPYNGCIQCPMGPQGQPGRPGLMGHPGDVGVTGMDGIPGGRGLPGYPGMPGDNGSGGANGHDGATGQPGRSGTRSYGSPGPAGSPGRVGRGGPPGPDGGYPMSGEPGSSGPQGKAGQPGGPGVDGHPGRPGGSGRPGPDSGYCKCPQRGKTDEFSNYNTKASTPTEKPEASSDWRRVRKVKVSA

>COL-152

MASIHFFTGIYSISTLLIFSSIICSAVILFEINDISNEVFNEMRAFNYYSTDA**W**EMMSNQINNSPPSQPTYFSRLARSTEQKCNCSQANNCPVGPPGPPGAAGTNGEDGSPGVAGQNGNEGKSINAQIDTYATPSCTQCTPGPPGAPGPDGVSGPVGINGRPGENGADGNPGAPGEVGAPGDQGGAGNQGAAGKPGLPGKNGQRGSGHPGPTGPVGETGNAGNNGHPGVDGENGKPGTRGEAGLQGKEGQTGPIGPAGQKGENGMPGLDAGYCPCPERTYGVEPMPPARQYEGI

>COL-153

MYLTTSDQILIGSLAASCITFLISLSVIALILRDVTSLFGELEVEMSSLKYDTNSA**W**SDMLHFRSEITQIDRIRRDIMQQKADYSGYQAQVIVSDFANDGSGEFPDETLELQRTEPMMPSFGPICSCFTLQSCPSGPAGPLGDQGIDGIDGIDGVDGYDGFDAYPSPPAKHSKAPFTCPRGPPGDIGSDGPRGKRGLRGPRGRPGRPGINGNPGFPGDMGAPGQPGVDGIVGERGVNGIDGVTYIPRQGVKGAMGQPGNEGQPGKKGRPGLPGTEGIRGYEGMSGLRGIEGVGGPPGPPGPQGTIGGDAAYCQCPNRGDNKVEELVGRGSGESPLFR

>COL-154

MEFEQRIKAYRFVAYSAVAFSVVAVLSVCITLPMVHNYVHHVKRTMNQEVQFCRGSAKDI**W**TEVNELKSIQHANRTARQAGYDAGVNGGSASAGGCDACCLPGAAGPAGTPGKPGRPGKPGAPGLPGNPGRPPQQPCDPITPPPCQPCPQGPPGPPGPPGPSGDAGGNGNPGSPGQDGQPGAPGNKGPSGPNGNPGAPGAPGQPGQDAPSEPITPGAPGPQGTPGPQGPPGQPGQPGHDGQPGAPGPKGPNGNPGQPGADGNPGAPGQSGTPGGVGEKGICPKYCAI**DGGVFFEDGTRR**

>COL-155

MEFEQRIKAYRFVAYSAVAFSVVAVLSVCITLPMVHNYVHHVKRTMNQEVQFCRGSAKDI**W**TEVNELKSIQHANRTARQSGYDAGVNGGSASTGGCDACCLPGAAGPAGTPGKPGRPGKPGAPGLPGNPGHPPQQPCDPITPPPCQPCPQGPPGPPGPPGPSGDAGGNGNPGSPGQDGQPGAPGNKGPSGPNGNPGAPGAPGQPGQDAPSEPITPGAPGPQGTPGPQGPPGQPGQPGHDGQPGAPGPKGPNGNPGQPGADGNPGAPGQSGTPGGVGEKGICPKYCAI**DGGVFFEDGTRR**

>COL-156

MSNTFIAGLTTVSGVAILGCLLFLGSIYQDINVLYDNVMGDMTDFQTYANDA**W**DSMITMNQPVRHLNADTIFGRNKRQASCNCGTQPSNCPAGPPGPPGAPGDQGLDGAPGQPGNVGQNAVVASIAASTSECIPCPAGAPGPQGPDGAPGAPGPSGQPGQDGASGQPGAPGVAGPQGDAGTPGQPGQQGQDGRPGQNGRRGRGTPGAPGAPGAAGAPGNNGQPGQDGAPGQAGAPGASGPDGQPGQPGEDGQPGENGLDGVPGTDAAYCPCPARSGPVEVAPARPDKKIRIRFSN

>COL-157

MEFEQRIKAYRFVAYSAVAFSVVAVLSVCITLPMVHSYVHHVKRTMNQEVQFCRGSAKDI**W**SEVNQLKSIQHANRTARQAGYDAGVNGGSASAGGCDACCLPGAAGPAGTPGKPGRPGKPGAPGLPGNPGRPPQQPCDPITPPPCQPCPQGPPGPPGPPGPSGDAGSNGNPGSPGQDGQPGAPGNKGPSGQNGNPGAPGAPGQPGQDAPSEPITPGAPGPQGAPGPQGPPGQPGQPGHDGQPGAPGPKGPNGNPGQPGADGNPGAPGQSGTPGGVGEKGICPKYCAI**DGGVFFEDGTRR**

>COL-158

MSVTKATAGALCLSSATLILSLYAIFSIYSDVQSIWSQLDQEMDQFKVTTDDL**W**TQMLGLGAATVSNRQRRQGKEQYGGYEAQGVNPGPTCSCSSGNGNGDDALGTGGCPAGPSGPQGSAGPDGIPGIDGQDGFPGENAEDSQNAPFNGCITCAPGKPGSPGERGKPGLRGMRGPRGTGGSPGTDGYPGRPGEMGPPGPPGDDGKPGSNGEKGQDVEQPTPRKGPRGPPGDSGPPGPEGDAGNDGPVGAAGAPGPDGINGFQGPGGPPGEEGKPGEDGKVGDDAAYCPCPDRNAPKENYASAPSHNPSNAPGASYSAGTGGYSGGTGHAQNSYGKK

>COL-159

MSSEDPKQIAYETESLRKVAFFGIAVSTIATLTAIVAVPMLYNYMQHVQSSLQSEVEFCAHRSNGL**W**DEYQRFEGVSGVAGRIKRESYHRKARASKVRRQSYGADAAVGGFGGSAGGSCCSCGTGAAGPAGSPGQDGAPGNDGAPGAPGNPGQDASEDQTAGPDSFCFDCPAGPPGPSGAPGQKGPSGAPGVPGQTGGASLPGPPGPAGPPGPSGQPGSNGNAGAPGAPGQVVDVPGTPGPAGPPGPPGPAGAPGQPGQSGSGQPGGPGPQGDAGAPGAPGQPGQAGAPGQDGDSGSEGACDHCPPPRTAPGY

>COL-160

MSSEDPKQIAYETESLRKVAFFGIAVSTIATLTAIVAVPMLYNYMQHVQSSLQSEVEFCAHRSNGL**W**DEYQRFEGVSGVAGRIKRESYHRRASGPHRASKVRRQSYGADAAVGGFGGSAGGSCCSCGTGAAGPAGSPGQDGAPGNDGAPGAPGNPGQDASEDQTAGPDSFCFDCPAGPPGPSGAPGQKGPSGAPGAPGQSGGASLPGPPGPAGPPGPSGQPGSNGNAGAPGAPGQVVDVPGTPGPAGPPGPPGPAGAPGQPGQSGSAQPGGPGPQGDAGAPGAPGQPGQAGAPGQDGDSGSEGACDHCPPPRTAPGY

>COL-161

MSAKTLVYGASVLSGFAILGCVFTVGYIFNDINEFYEQTMETMDEFKLNERGA**W**HGMVERTRAPSEILFGRAKRQAGQCNCGAQSSGCPAGPPGPPGAPGAPGDDGHAGEAGKPGTAGVAVGIVSEGGPCIKCPAGEPGPAGEAGAPGPAGPDGQPGQDGQGGQPGPAGPAGPAGDAGAPGAPGQDGQPGAPGQDGQRSTGTPGAAGAPGPAGPAGQDGQPGSDGGPGEAGAPGAPGPDGQPGQPGQDGEAGAPGQDGQPGSDAAYCPCPARSVAVQRSVSRRRASKVVVA

>COL-162

MSAKTLVYGASVLSGFAILGCVFTVGYIFNDINEFYDQTMETMDEFKLNERGA**W**HGMVERTRAPSEILFGRAKRQAGQCNCGAQSSGCPAGPPGPPGAPGAPGDDGHAGEAGKPGTAGVAVGIVSEGGPCIKCPAGEPGPAGEAGAPGPAGPDGQPGQDGQGGQPGPAGPAGPAGDAGAPGAPGQDGQPGAPGQDGHRSTGTPGAAGAPGPAGPAGQDGQPGSAGGPGEAGAPGAPGPDGQPGQPGQDGEAGAPGQDGQPGSDAAYCPCPARSVAVQRSVSRRRASKVVVA

>COL-163

MSRLAVENYVVVGITLAVITASCQLLFLPFLFSEIDAIRHEFDVEIREAMRSFESSYEVLAGFRGEPGEVRKRREAAKNMIHMDGDTVGPADHPAHAAAAKTHGTRLSGIAGQSGGAPPSGPSLFSPELRKLAREVPAPTDDYDDEESEYEDAEAMSDTSGGAQVILGTIPTSTTTTTASPSPTPQQHPTLMNSGERLGPLPICPLDENRCPMGPPGPPGLRGTPGQSGTDGNDGLPGFDAMDEITPNFPTFCVTCPAGPDGTPGQPGGTGKPGLGGAPGILGPMGKNGQPGAPGGYGSPGKQGSMGSRGVAGNVGQDGVVLLNTKGPKGPSGKHGTEGQPGEDGTNNNTPGPIGPPGLPGSPGAPGAAGIRGKRGEFGVQGQKGVDSKECTCESLMKESIVSAENLAYLAVEAQSQAMGHQIDLKASEIRRTQQKASGLASGLPTLNELRNKYQKGAVTFAQEDSTIQKFAVAPSDVSISETEELLPASNFESPAAEKSDESTFAAESEDSETIDEPMVPPKAQFPATFAIAQAVTSENSDTSVSDPEPLDSGTGHSRPALFAPPRAQLPSQSVPKAQAHGAKSHSRGSGESKKKSAPLNGNTEIVKKTRGSGSTKRRRVVKVKRVPYRHPAFRVFSTTNEKFAMSMDEGERGFRAEKEIARESEKKVVNLREIRRSPKKLRRKLKPGKTEKKLRRVRVKPSKSLADQQRQNSNILENIAESLGLIENHPAKMSRVAIP

>COL-164

MSYKTMESSSSTVSFAVILSGSAFLIMVTATPVLFYTMGDMTAQLETAKTGFEETSNVL**W**KELVTNESHRRVARQTYKEGGAAGEGSPAATAPEASPVKIDDSCPAGPKGESGSAGEPGTDGTDGTPGKDGADGSNEQTASQAGDLCTACPAGPPGVAGYKGKRGPRGEKGDKGAPGNAGRDGAPGDDGPEGDHGHPGPMGPAGEKGTSGADGKGFAKGAPGPKGPTGMDGLAGDEGLPGERGEDGSAGAQGETGPQGPQGEKGPDGFPGLPGSAGASGEDAAYCPCPSRKGDIAGGGGGGYAAAPGTEFEGSPVESDPAKLSADIPAPSPISDVSAEGAGAPVSDASGEGPADSATEGEGAPAGGAPKGDTTYPTKNRARFTARQRAVSRIRRTF

>COL-165

MEGKETDQHRHMRVVVMAACTVATISVFSAILVLPVLFTSVQSLQQEIDFETNFCKMRSRDMLVNLYQTGGQNRVKRGWLFGQWIPDSGAGGGAGKEDYGAPATEQPYNAPAPSPSHGGYDQPSNNGYGPVVNAEPAPQCCTCQQGNAGPPGPPGDDGHDGKDGNAGSDAHNGKDGGVAPSDGLQSEPCMICPPGPQGLMGQAGKKGPTGPRGSPGLAGIDGRRGEPGMVGPTGLDGEQGPQGPPGKRGEDGRVIDINGPQGAPGAPGAQGRKGESGPKGRRGNTIPGPQGPNGDAGRKGRAGHKGEAGGPGGLGSKGPNGDCFHCPTPRTPPGY

>COL-166B

MSDEKQLEIEASSLRRVALVGVAVSFTATLVCVIAAPMLYNYMQHMQSVMQSEVDFCRSRSGNI**W**REVTRTQVLAKVSGGALRSRRQAGYESAGVEGSSVGHQGGCCGCGVSAAGPPGAPGSDGEDGADGQPGAPGNDGPDGPAATPAPAHEFCFDCPAGPPGPAGPAGPKGPNGNSGSDGQPGAPGNNGNAGGPGAPGQAGQDGHPGNAGAPGAPGKVNEVPGPAGAPGAPGPDGPAGPAGSPGAPGNPGSQGPQGPAGDNGGAGSPGQPGANGDNGADGETGAPGGCDHCPPPRTAPGY

>COL-167

MSDIKQLEQEASSLRRTALVGVAVSFTATLVCVIAAPMLYNYMQHMQSVMQSEVDFCRSRSGNI**W**REVTRTQVLAKVSGGALRSRRQAGYESAGVEGSSVGHQGGCCGCGVSAAGPPGAPGQDGEDGADGQPGAPGNDGPDGPAATPAPAHEFCFDCPAGPPGPAGPAGPKGPNGNSGSDGQPGAPGNNGNAGGPGAPGQAGQDGHPGNAGAPGAPGKVNEVPGPAGAPGAPGPDGPAGPAGSPGAPGNPGSQGPQGPAGDNGGAGSPGQPGANGDNGADGETGAPGGCDHCPPPRTAPGY

>COL-168

MSDIKQLENEASSLRRVALVGVAVSFTATLVCVIAAPMLYNYMQHMQSVMQSEVDFCRSRSGNI**W**REVTRTQVLAKVSGGALRSRRQAGYESAGVEGSSVGHQGGCCGCGVSAAGPPGAPGQDGEDGADGQPGAPGNDGPDGPAATPAPAHEFCFDCPAGPPGPAGPAGPKGPNGNSGSDGQPGAPGNNGNAGGPGAPGQAGQDGHPGNAGAPGAPGKVNEVPGPAGAPGAPGPDGPAGPAGSPGAPGNPGSQGPQGPAGDNGGAGSPGQPGANGDNGADGEIGAPGGCDHCPPPRTAPGY

>COL-169

MSDIKQLEHEATSLRRVALVGVTVSFAATLVCVIAAPILYNYMQHMQSVMQSEVDFCRSRSGNI**W**REVTRTQVLAKVSGGAIRSRRQAGYESAAVEGSVGHQGGCCGCGVSAAGPPGAPGQDGEDGADGQPGAPGNDGPDGPAATPAPAHEFCFDCPAGPPGPAGPAGPKGPNGNSGSDGQPGAPGNNGNAGGPGAPGQAGQDGHPGNAGAPGAPGKVNEVPGPAGAPGAPGPDGPAGPAGSPGAPGNPGSQGPQGPAGDNGGAGSPGQPGANGDNGADGETGAPGGCDHCPPPRTAPGY

>COL-170

MSDIKQLENEASSLRRVALVGVAVSFTATLVCVIAAPMLYNYMQHMQSVMQSEVDFCRSRSGNI**W**REVTRTQVLAKVSGGAVRSRRQAGYESAGVEGNSFSQGGCCGCGVSAAGPPGAPGQDGEDGADGQPGAPGNDGPDGPAATPAPAHEFCFDCPAGPPGPAGPAGPKGPNGNSGSDGQPGAPGNNGNAGGPGAPGQAGQDGHPGNAGAPGAPGKVNEVPGPAGAPGAPGPDGPAGPAGSPGAPGNPGSQGPQGPAGDNGGAGSPGQPGANGDNGADGETGAPGGCDHCPPPRTAPGY

>COL-171

MSRSIFIQMSDSKQLENEASSLRRVAFVGVVVSFTATLVCIIAAPMLYNYMQHMQSVMQSEVDFCRSRSGNIWKEVTRTQVLSKVSGGAIRSRRQTEYENLGVEGSSSQGGCCGCGTSAAGPPGSPGPDGQEGSNGRPGAPGTNGPDGRPATQASASDFCFDCPPGPPGPAGSIGPKGPNGNPGFDGQPGAPGNNGFAGGPGAPGLGGKDGQSGNAGVPGAPGKITNIQRPAGLPGVPGPIGPVGSAGTPGSPGNPGSQGPQGSAGDNGGDGFPGQPGANGDNGPDGETGVSGGCDHCPPPRTAPGY

>COL-172A

MDSSEKQLCAETERIKKTAFLSVAISTFVVFCSVVMLPLIYNYVQTLQTHMLNEMDFCRTKSRDL**W**VEVVSVDGAFGRGKRTIRSGDRAKREWSFGQFFERSQRATGSGSGSSGGYGTYPPAPTVPPTYGDYKVAAVEEFQPASCCTCQQGPVGPAGPPGDDGLDGIDGKSGVDGPPGRDGILLPPLGQAPEPCIICPPGQTGPPGFPGQKGPNGPRGSPGLQGQDGKKGEQGMPGPQGPTGRPGRPGPKGPKGEDGRVIMVAGPAGPAGPPGLPGTPGKRGARGMDGLPGPQGAPGQQGDSGLSGPDGKEGARGERGPQGPQGLKGSCEHCPLPRLPPGY

>COL-173

MELDPKEEAALRAEAERLRRMAVIGVALSIGAAIVCVMTVPFVYNYVQRVETVLQNEADFCRAKRDSVLKELTRTQKRAGLRLKREDTYSGYSASYGTYDSVPVMHYKSPEVTEHKEERIEKKDDFQQCCGCGFGSPGEPGSPGQDGHDGVDGRPGSDGNPGADMHLDDTYNMADQFCFECLPAPTGPPGPPGLKGMPGIPGNQGEDAGPGRQGMPGPTGPPGPRGPPGETGDEGEGGPDGIPMEKKSPPGPRGPPGPAGPDGPQGPEGRSGKQGPPGPTGQEGEEGVIGEPGKPGEPGKSGAAGARGPAGPCNHCPNPRTAPGY

>COL-174

MTSSGSDDLKSLQEQANSLRKFAFYGVATSTIATLFSVISVPILYSVMQHMQSSMQSEVDFCRLRSGNI**W**RELSKTQILTQVAGGAIVRSRRQAGYSSGPLESAVESAPANVGGGGGGCCGCGVSPQGPPGVPGRDGDNGADGEAGAPGKDGPDGPAATPAPNYDWCFDCPPGPPGPAGSAGVKGPSGNPGAPGTDGPAGNKGGQGPPGNAGAPGANGNPGNAGTPGIPGKTIEVPAPAGPAGAPGPAGPPGTAGTPGSQGQPGKDGSPGDAGDNGTAGSPGHPGGEGPVGPDGARGPSGGCEHCPPPRTAPGY

>COL-175

MTFALTVSCATSAVVITASLCSVLVIMNDINKLSEDISIGMENFKDVSDVA**W**GNIMVLHGGVRQQSASVHRDVKKLMGINLRNKRNSDSCQCESRAAACPAGSPGEKGEPGLAGLPGPDGEDGKDGAPGVALLVTHDIPGGCIKCPAGPRGPRGDAGPPGPPGNGGRPGQRGTPGEAGTPGPVGEPGETGGPGPQGRPGQRGEPGQPGTVHTPGNPGRPGPQGPRGQQGEPGKDGSKGNPGDEGRQGHPGKNGRTGAHGKDGAPGKCGKEGPIGPDAGYCQCPSRSN

>COL-176

MFEEKLLVSVASAFSTLAIVACLVVVPSLYATIDEIHAEVLDGVNVFRVETDSA**W**TEMMDIQILVTPPTKPRVNPFNSVFRQKRQTFSGLPAWCQCEPTKPTCPPGPPGPPGPAGQPGTPGAPGPKGQDSTTTFAPINCPTAPSDCIKCAPGPAGPAGSAGPAGPKGPDGRPGQPGSAGHPGAPGQPGSKGNNGAPGAAGGPGQPGRPGKDGQRGKGSAGAPGAPGKAGPAGPAGGPGNNGSAGTPGPAGPAGNPGTPGNKGSDGHPGTPGAPGGPGHDAAYCACPPRSAVFVARRH

>COL-177

MKKKEVEKDDLIENASRVASCISIFLIIASTTMYIFIGLKINHISHRLYNHAKGFEILKLDAETELLHVEDEHIRTSRQAWRKQQQRWRSGNGSGNRRKTQFEEPKICDCTVIDCPKGPRGPPGDDGNSPTDGHPGDNGKDGTDGIYQVDEPDCPPCPQGPPGEDGLRGEAGEPGRPGTPGDPGRPGTNEPGGIGARGPRGQSGRTGSKGDPGEAGQDFVQLNGLPGPKGLPGSVGLPGARGDPGNNGKPAPPGPEGFPGPVGDLGDAGEYGLRGVPGRRGSPGKDGGYCQCPPREGSSFSSSTSTLNRQSVKVSTSRNRLTSNNNDAVDDYEAEVERHVTPRQQQQQQQPFQRRRWQKTNQQNQRDMYVNLRKNTHSDLIDVGESSMRRSPMAPLIAYKDSREEGHTNRVVMRKTSYGSKANTVDFELR

>COL-178

MEDKAKLVQASELKKFAFFGVAVSTVATLIAIIAVPLLCLHMQTVHSGLSDELLFCKSKNVDMRSEIEKLSVIRDNGRQKRQTPQTCCSCGIGETGPAGVPGQEGAPGNDGKAGQPGAPGADADEQGFHYKAPEFCFDCPAGPPGAVGGPGPKGPPGPPGGPGELGGPGRGGNRGPPGPRGPPGEAGPDGEGGRPGQAGQTRSAPSPPGQPGQPGEPGSPGEPGPDGRAGHPGRNGPPGPPGDNGGQGEPGKDGEDGENGAAGAAGPKGSCDHCPPPRTAPGY

>COL-179

MEDKAKFAYAADLKKFAFFGVAVSTVATLIAIIAIPLFCVHMQSVTSGLSEELLFCKSKNVYIKGEIEQLSVTREAGRQKRQTPQTCCSCGIGETGPAGVPGQEGAPGNDGKAGQPGAPGADADEQGFHYKAPEFCFDCPAGPPGAVGGPGPKGPPGPPGGPGELGGPGRGGNRGPPGPRGPPGEAGPDGEGGRPGQAGQTRSAPSPPGQPGQPGEPGSPGEPGPDGRAGHPGRNGPPGPPGDNGGQGEPGKDGEDGENGAAGAAGPKGSCDHCPPPRTAPGY

>COL-180

MSDIKQLEHEASSLRRVALVGVAVSFTATLVCIIAAPMLYNYMQHMQSVMQSEVDFCRSRSGNI**W**REVTRTQVLAKVSGGSVRSRRQAGYESAGVEGSFSSQGGCCGCGVSAAGPPGAPGQDGEDGSDGQPGAPGNDGPDGPAATPAPAHEFCFDCPAGPPGPAGPAGPKGPNGNPGSDGQPGAPGNNGNAGGPGAPGQAGQDGHPGNAGAPGAPGRVNEVPGPAGAPGAPGPDGPAGPAGSPGAPGNPGSQGSQGPVGDNGAAGAPGQPGGNGNNGADGETGAPGGCDHCPPPRTAPGY

>COL-181A

MRAKRRRLSIHKTILCNVSVTINSYPQSELYIKAGDVTSNVSVPSFLLFSKMTMDKKFETEKVKRFAFFGIAVSTVSTLTAIVAVPMLCLYLQSVSSSLQDEVNFCRVRATSLEGELAKLNVVRAPRKARAAEGTCCSCGVGSAGPPGPPGFDGSAGKDGSNGRPGNPGEDADGDDYKPDASQFCFDCPEAPAGPPGRPGPDGENGGAGEPGRDGLAGRPGARGSPGPRGPSGNPGADGPAGQLGPQGGSNVAPSPAGEPGAPGAQGPKGPPGAAGRPGRDGQPGAPGDQGENGPDGAPGKSGSDGQPGAPGKEGSRGGCDHCPPPRTAPGY

>COL-182 (ancestral)

MSDSARIAAYGAITICTISITATLFYLPVFFSRIENIRTSLAFSMEEFNTLQAEV**W**GDMVHLRGEIPGLKRHQVKRQAGNEQCNCDQQNLCPTGPKGRPGSDGLDGLPGTPGEQGEQGLAGIAPEAEKTTGNNYIQGYPGQPGPQGLPGNQGDQGEIGREGSPGAQGAPGDMGMMGEAGKEGEKGAPGREGVRGEKGPSGPPGLPGLVGPKGFSGNSGPPGSPGEPGPEGEQGPAGKDGAPGYDGGFGNYGEAGQPGEDAGYCKCPTRKQRKH

>COL-183

MESHVDLKLRTYRFIAYSAVAFSCIAAVSLCFTLPLLYNYVQNTKIQVQKESMYCKGSVHDM**W**SDIRDMEVTHNRTARQAYASAGGAGGGGGYGAGGGGGGGGGEAAGGGGGCTGCCNPGPPGPGGRPGKPGTPGKPGAPGNPGASGKGAAAPCEPSTPPPCQPCPAGPPGPPGPDGTPGEPGGPGPAGSPAGPSGPGPAGPPGPAGPPGNDGQPGQPGGPGQDGASSAGGEAGPGPAGPPGPAGPAGPDGQSGSGSAGGPGPKGPPGPAGQPGSDGNPGTAGPPGNPGGEGEKGICPKYCAI**DGGIFFEDGTRRR**

>COL-184A

MNLFQMSYAVAVTSAASIVATGMLLISVGNIISDLNNLQLEITDGMNDFKMKSDET**W**SRILSLHVNPTGETAAPPNFKTLFGRHKRDNSQCHCGLQSQGCPDGPPGARGPQGAPGLPGAPGVDGKPGVDGKSLTYVHAAEADCIPCPGGEAGPPGPDGEPGVRGPNGPPGPNGHPGQPGRPGPAGPAGPEGKDGPVGPHGPAGPAGPRGTRHSAGVPGKPGDVGPCGPEGPKGAPGTPGKNGAPGDRGFGGQPGVPGEPGEDGTPGEPGADGAPGDDAGYCACPGRTMFEA

>COL-185

MESPTDLRLKAYRLIAYSAVGFSVTAAVALCLTLPLIFTYVENTKAQLHRESLYCKNSVQDM**W**SEINSDVSVNRTARQAGYSSGGPDAGAGAGAADAAAGGGCTGCCNPGPPGPGGSPGKPGKPGKPGAPGAPGAAGKGASAPCEAKTPPPCQPCPAGPPGPPGPDGPAGPAGPDGEAGSPAAPSPPGPPGPPGPAGPAGNDGAAGTPGPDGPAGESTYPEPAGPGPAGPPGPAGPPGPDGASPTAAPGAAGPPGPPGPAGAPGPDGNPGTAGPPGKAGGEGEKGICPKYCAI**DGGIFFEDGTRR**

>COL-186

MSTVATFVCIITVPLAYNKMQQMQSNMIDQIDFCKTRSNLF**W**REVTKTQYMADSRGIRVARRAEKRYSTNYDNQRTAYDRFYHAQQQYESRDSNYDKSASYGLWNTESPIPARNYESSSSSAPVYGNNYGSQGGGGGCCGCGQSPPGPPGRPGSDGSPGSDGEAGIPGRDGPDAPRATPAPNFDWCFDCPPGEPGRQGKQGRKGPPGRPGGSGLPGDKGLPGLPGSMGPVGPEGRPGIPGLPGGKGIPGKLIEVAGLEGPPGQSGLPGMPGLQGKPGNDGYPGRDGSPGDQGDDGIPGGPGKPGQKGYPGPDGSAGFPGGCDHCPPPRTAPGY

>COL-187

MPSSSPSFVSYISLPLISLIAALCGVFYYITVYQELSALSLNVKKSVAEFSVATNFA**W**EQIATLNELHLVSKRGISKKSVFVDRIFRSPFNQIATLPPWCQCEPIRPKCPPGPPGPPGCRGEPGPSGLPGRRGINNYETLPLKKCIWRERLACIMCPRGPPGRRGRMGDDGEPGAPGRRGLSGTFLTGIKKLRGPPGEPGEPGRPGTPGTPGNDGKDGRNGYRLRPDKITRGPPGPMGARGEPGHRGLPGDNGMPGPMGPPGYRGDNGRRGAPGLPGRTGRHGKPGGDSSYCPCPPRSLIVESFKKI

>BLI-1

MVKSYTLAGLAAIACLLLAIGAVISTVYILNDISDFYSEAQEELVEFKDIANNI**W**EEMVFELTPEEMREAEDNDREKRSYEPEGPYQSETTTPSTTTSTAATTTEAAEDESGYDFVNDNGPPSSRPRKPEPPTMPRTIQGFRAPPPAATSTYRPPHGSNYDNYGREPASSRRPYPPQQPPSTSAPHSSPNNRTSLYNPQPPPKTGYPTNPRVPYNPPQPNYTRQPTYPEDNRAPYKPTRSPNTPPPRQPSGGYDSDGQTPPSSPRIYNTRRPNNHGPGYPEDQVPTAPPVPGQQRVPPTQTRNPPNPTNTRQPSRPVPPTSDGHIEATTPYNPSAQYPTGKRGSHPGFGPQRPRPGTRPRGNPCDQCSAQPNHCPSGPPGPRGRPGPPGFPGQDGPRGLRGLNGGYSGVQPSSYDPVIGCVQCPIGPPGERGPDGTPGVPGEDGIDGEQGVNGQDGQPGAPGAPGYHGMNGSPGTPGKPGLPGRNGQSCKSIPGPPGQPGVMGVPGRDGDPGTDGEHGQDGSPGIQGPPGRDGTSGPDGQPGVSAPGAPGTDGGYCPCPKRSSKFDFNDAYNDDEKRGLEEHRPRGYDSERAEEPRPRQTVRTNTYDENSGAEHQRRPNYEPSAEVAPPRQDRYEDEERVREPPPKRPPPPHRQTPHELYPEEQPYVRRPPPPQNRGNYEVERSREYVPEAPRPRQGYEHSSGYGGDDRKEQPKYMESRPVDEPRYETDAPSRSRPLKKVEIIRHPERGYDRRQPSYEDSKPRQEEPRRYETEAEPRYEEVPRKETHPPGFGRPKSEENPRETGRTYSEEAMPPVQQTEKYNGDKRKKNQPEYEDISKPEEDKERAMEKHHKKPNKFQEKQWEEHRKSQELRNSREHGGQVPVETSVPMQQVKPESSAEEKPVPPVHDNFQSSEKRAFSNENPLPSEQIPYRRRNRFYRNHYYEKFLV

>BLI-2

MDEKELNHEASMLRKVAFLGICISTVSTLTCIIAIPLLYNYMQHVQTNLHSEIDFCRHRTVGLFIQYERMQSASGIKGRRIIVKKQAGYDFAESNTNAESGFSSSKSSLAPGGQCCSCKTGPSGPPGPPGEDGRDGRDGKPGLNGEDGTDAKDSAPRRDAAAPCYDCPVGPPGPPGNIGSKGQPGRNGKDGLPGVPGLPGQPGEPGDDGEPGEDGDPGQPGDNGEPGKCDEVNVAQGPPGSPGPPGLPGPDGLPGTPGNPGQDGEQGPAGEPGRDGKDGQPGRPGQPGPPGEPGTGGGCEHCPTPRTAPGY

>BLI-6

MEVETRVKAYRFVAYAAVTFSVVAVLSVCITLPMVYNYVSHVKKGIHHEIRYCKGSAKDILNEVSSIRSNRTARQASYGDSGAAGSAGAAGGSCSGCCLPGPAGPVGTPGKPGRPGKPGVAGLPGNPGRPPQAPCDPITPPPCKPCPQGPPGVQGPPGPAGDAGLDGQPGGPGQDGQPGQPGPKGPPGVNGQPGAPGQDGNPGQDAPSEPLVPGEPGAPGEAGPQGPPGQPGTPGQNGQPGQPGPKGPSGQPGQPGSDGNPGPAGQAGQAGSVGEKGICPKYCAI**DGGIFFEDGTRR**

>DPY-2

MKSQTSGYVDPQTEEYDESREKSYQVLIAVSGVLSITSLLILVLFVPSMYNYVDNISRFSRRDFEFCQASANDLETEMMSVREGLLRGRNVTKRAAGYGHYNPSMLAADSPQFQECPACCIPGERGPSGDSGLPALPGAPGPDGAPGRPGTTPNASCIPERVFEPPPCLPCPQGPRGVPGHPGFPGDPGEYGIGGRPGSDGMPGKPGDPGLAGPIGPPGESGPIGDKGRTPEAHVIPGPPGDSGLPGPWGPPGSAGMPGEDGYAGTPGEKGWPGPPGAPGPGGMPGPNGPTGEQGPSGTPGTCVCQDTEVVMNDEKGRVPAPRDNVAPGATGGSYEPQGGNDAAAPSAISQSQNDGYGNGESVSNHKNNGGYYHLRKFTQ

>DPY-3

MEQKCAPRRSLRLLAIASATLAIVSMLATVIIVPLVYNHVQHLQSVMNSEVDFCKTRSRDL**W**REMVTVQSATGGIPARTARRTRRDNYGAQPIAANPPSSAAGSCCTCQVGPPGPPGPPGRDGRPGAPGRPGNPGPPGRDGALLPGPPPKPPCQKCPPGPPGPAGPPGPKGLPGPQGDAGTSGQDGVPGLPGPPGPSGPQGAPGVPGEKGPTGEPGKVINGAPPGPPGPPGPPGPQGPPGPPGKDGQPGKAGPPGLPGDPGEKGSDGLPGPHGGTGPRGPPGQPGSCDHCPPPRTGPGYARR

>DPY-4

MDIDSKIKAYRFVGYAAVCFSAMAVLSVCITLPMVYNYVQHVRTQMHQELYACKGSAKDV**W**NSVYSIRELPLSANRTARSTYNEQCAGCCLPGPQGPQGTPGKPGKPGKPGAPGQPGTPGRPPQQPCEPTTPPPCQPCPQGPPGPPGQPGIPGDNGPPGEQGPKGPDAAPGEPGPKGPIGPPGPPGQAGAPGEPGSPAKSEPAVPGPPGPPGQAGQQGPPGPPGSNGIDGAPGAPGAKGEPGTPGEPGKDGEPGKPGTPGQDGTPGEKGICPKYCAL**DGGIFFEDGTRR**

>DPY-5

MVKAVVGFGAACGISAIVACLWAALVITNDINDMYDDVMGELGGFRDISDDT**W**GTLLDVRHGAGESAEQYVRGIFGRHKRSNSQCSCGLPSQGCPAGAPGNPGAPGEPGGTGPDGKNGPTGLPGLNIPIPNDFPKECIKCPAGPPGQDGLPGQEGFQGLPGDAGKRGTPGKDGEPGRVGDIGDQGTPGQDGQPGLAGPPGRDGLTGKGQPGVAGRPGMPGPRGEPGNNGNPGEEGQTGAQGPTGQPGKDGFNGNDGTPGQAGPQGAVGADAEYCPCPERKRRRV

>DPY-7

MEKPSSGANHVAKATVSLSIASVLILGAVLTMLSIQLDEAHERLQNRMGSFKFVARNI**W**HDIVLVKSNGRIKRQYGGYGSDSAQSDNQQCTSCVQLRCPPGPIGPPGVSGEPGMDGANGRPGKPGLDGLDVPLDPEPAFPCVICPAGPPGTRGPQGEVGRPGQTGESGHPGLPGRPGKPGRVGDAGPQGEPGEQGEPGIKGPPGDDSIGGTGIKGPPGPPGPRGPKGPPGSNGLPSQNSGPPGPIGEMGPPGPPGPRGEPGPPGPFGPPGDSGEPGGHCPSSCGVQEIVAPSVSELDTNDEPEKPARGGYSGGGYGKK

>DPY-8

MRDFYDVAVASIVLSTFTLVVAVFSVPMLFHKSDNVLLEISSFSDGIKAKSDLV**W**NELMEIKQGDAVPGIQYFSRKARSNWMGGAFCKGCFLLSCPQGPPGPPGAPGPDGQPGDAGRQGRSGDDGFDIQPESEPELPCVICPAGPPGPRGAQGERGMTGNPGMAGMQGTIGMEGVDGGPGLPGPPGPKGERGPLGPYGPPGDTVVAGVGIKGPLGPPGPQGMKGPPGPSGKGSNQPGQPGQPGDIGGIGKTGKMGGYGMEGPAGPPGDPGMPASYCPSDCGVQPILTEMFPTPFTPTAGYSNMQRPAAPSRPLAHPEYADGSAPAHLPHSGASDSAAASEVDAPAPHQHRKSHRLRPSHQQQYGDEQNNAVADAGRSVNAPEYGDGSAPSHMPSHRAHHHPNSASQNYVDPERGSDTAAADAYANRMHDFQDFERKWRKKLLQRRLRMMKRQ

>DPY-9A

MEPSNIWRTSTIVGSVVSTFAVLTVIIGLPLMHNHVQKVTTLMLTEVELCKTESQDI**W**KQMKFSRMAPNRTKRQSPYGNYGASGSGSCCACTQGSPGPRGQPGDDGEPGRDGFPGREGDNGIAGKYLPAPPPGTNACQKCPTGAPGPPGLPGPKGPRGPAGIEGKPGRLGEDNRPGPPGPPGVRGEPGSPGEKGPTGDRGKVLNGAPPGPTGPPGKVGPRGLSGGKGHDGKPGLGGQPGVRGAVGARGDAGNPGLPGPQGPKGEPGNPGTCHHCQNRAPAPSEAPGKAPPPPQDYTRPAPESYPSAPNGQYLWIH

>DPY-10B

MKNNAKEDYRTFSLTTNYSRQMIYRCVTGLQIGFSLFSFIIVCVALPIMYNHVQNTITYVDREMAYCEVRFLYFYCIKFFSTLQRSNDEAALELQYGKMRMTGNRTARGAYGSGASHGFRPTAYGDEITGAPLETECPGCCIPGPPGPRGSSGTPGKPGLPGNAGKPGMPGTTPNQTCPLNQVREPPPCRPCPKGPPGIKGWPGFPGDVGPPGPPGLKGIDGEDGAPGETGPTGPPGYRGGPGAPGDKGPTPEGDLKEGPPGDEGPPGPIGAPGMPGLPGRNGLTGGQGERGWPGVSGESGEPGYPGPEGPMGGQGPPGEPGVCVCQNVDSILLINPGPQPRIRADDYNSDDGYGGSRGGGDRAGYQGYGRK

>DPY-13

MDIDTKIKAYRFVAYSAVVFSVIAVLSVCITLPIVYNYVHTVRRQLHNEALTCKGSMKDV**W**ADVHHLRVDAASN**R**TARAVRYGRDDAAAGNGPNFDSGCEGCCQPGPQGAPGAPGKPGRPGKPGAPGFPGNPGKAPQKPCEEITPPPCKPCPQGPPGAPGLPGDQGDKGEAGQPGQPGSDAAPGEQGPKGPNGAPGKPGAPGAPGDEGLPAVCEPVQKGEPGSTGEPGPVGPPGPSGQPGNDGTPGQPGPKGPPGPDGKPGADGNPGQPGPVGPPGTPGERGICPKYCAI**DGGIFFEDGTRR**

>DPY-14

MDGKDQHCHARRIAIAAIIVSTCAVVSSLVALPMIYNMVISIQSEISQETGFCMMRSRDMLVNMYSTAGKSGYRAKRAWQFGSWVQDSGVGGGAGGYGSGPADSGYGAQGTNNGYGPVVNAEPEPQCCTCQQGKAGPPGPPGDDGKDGNDGSAGNDAKNGKDGGVGPSDGLQSEPCMICPPGAQGLGGAPGAKGPQGPRGSPGLSGVDGRRGEPGMSGPAGTQGEPGPQGPPGKKGDDGRVINVNGPPGAGGAPGPQGRKGERGPKGVPGSVHPGVQGPTGDQGRKGRAGRKGETGGQGPIGSKGPNGDCFHCPTPRTPP

GY

>DPY-17

MSVFAGYAACTLGAVSMLLCVSLVPQVYQQVSMLRDELTTEMEA**W**RLESDQIYMDMQKFGRVRRQAGGYGGYGGYGSGPSGPSGPSGPHGGFPGGPQGHFPGNTGSSNTPTLPGVIGVPPSVTGHPGGSPINPDGSPSAGPGDKCNCNTENSCPAGPAGPKGTPGHDGPDGIPGVPGVDGEDADDAKAQTQQYDGCFTCPAGPQGPPGSQGKPGARGMRGARGQAAMPGRDGSPGMPGSLGPIGPPGAAGEEGPTGEPGADVEHQIGLPGAKGTPGAPGESGDQGEQGDRGATGIAGPPGERGPQGEKGDDGPNGAAGSPGEEGEPGQDAQYCPCPQRNTNAAVSGNQGYRN

>LON-3

MSVTTATSGALIFSGASLLVSLFAAASIYSQVSSI**W**TELDNEIDSFKLLTNDI**W**GDMINLGAGSASNRIRRQAYGGYGATGTNAPEPQFPQGDKGPLPVPGLPFGPNVSGGSDRCQCTVENTCPTGPDGEEGEQGPDGQDGVDGVPGFDGQDCPDVEQQPSQGCFTCPQGLPGPQGSQGAPGIRGMRGARGQPGYPGRDGQPGMPGEMGPTGAPGDDGAPGASGMKGDDAEKPVGRQGQRGQPGEQGPDGEEGPAGKDAFEGPPGVEGEVGVPGYQGSAGPDGEEGPRGPSGLPGKDAEYCKCPTRD**DGGNSHRAWRRKHKRVY**

>RAM-2/RAM-3/COL-82

MSSADNEKKLWVEAESLRKVAFFGICISTVATLTAIVAIPLLYNYMQHVQSTLQTEVDFCKHRTNGLFDQYERMQQLKGVRGKLIVKRQAGYDAPADAAVQATTQSSGGSCCSCKAGASGPPGPPGTDGSDGKDGLSGPDGQPGADADPEATPSEADFCFDCPAGPPGPAGNPGPKGPSGNAGADGQPGAPGNAGAAGAPGAPGPAGPDGQPGSTGQDGAPGVVEEVAVPAGPAGAPGPAGAPGADGAAGAPGQPGQDGPQGPAGDAGSDGAAGQPGAAGEQGPAGEPGAGGGCDHCPPPRTAPGY

>RAM-4/COL-34

MDLETRIKAYRFVAYSAVAFSVVAVISVCVTLPMVYNYVHHVKRTMHNEITFCKGSAKDI**W**NEVHALKSLPNSNRTARQAYNDAAVTGGGAQSGSCESCCLPGPPGPAGTPGKPGRPGKPGAPGLPGNPGRPPQQPCEPITPPPCKPCPQGPPGPPGPPGPPGDSGEPGSPGLPGQDAAPGEPGPKGPPGPPGAPGAPGTPGEPGVPAQSEPLIPGEPGPPGEAGPQGPPGSPGQPGADGSPGQPGPKGPNGPDGQPGADGNPGAPGPAGPPGSPGERGICPKYCAI**DGGVFFEDGTRR**

>ROL-1

MEVDSNTKAYRIIGYVTVTVSTLAIIALCVTLPIVHGYIKEVQHSMKIEMNQCQNNAKTL**W**TDVYMLRASFTRGFNRTARQAGYEYSNDETTPFAPPKFGYPVVGEAKEEANPYDAPAPPPPKTEAPTTTTKKTTTTTTTTTTTTSTTTTEAPTTTKTTTTRRPSTTTTGSVPSYPDASEAETTHGYDDVAVVTDAPTVSDAPASEPSQPTSTLEYLKSTVPPEEPHGTSGPSGTTAAPTAEVTHEDHEESTATCDNCCLPGPAGPPGAPGRPGPPGKAGANGLPGNSGKPTKRPCNPVTKPPCVPCPQGPLGPAGPAGPQGNLGGPGVFGEKGPQGPVGAPGQKGRRGEKGKDGEPGAPGAPGENVKEVPAIPGPQGTPGPRGRQGPRGAPGIPGKDGLGGDQGAPGEGADGEPGMDGVPGNPGRDGTHGHGGEKGVCPKYCSA**DGGVFYEDGTRR**

>ROL-6

MTLTTATSGAIVFSGATLLVSLFAAASLYSQVSNI**W**NELDAEIANFRSLTEDM**W**VDMVKLGAGTASNRVRRQQYGGYGATGVQPPAPTPNPYGGYGASQPAPPEKFPDGIPNGGNQPKFPGGGFPDGPFPNGGGPRGGNQCQCTVENSCPPGPAGPEGEEGPDGHDGQDGVPGFDGKDAEDVQNTPPTGCFTCPQGPLGPQGPNGAPGLRGMRGARGQPGRPGRDGNPGMPGDCGPPGAPGSDGKPGSPGGKGDDGERPLGRPGPRGPPGEAGPEGPQGPTGRDAYPGQSGPQGEPGLQGYGGAAGEDGPEGPPGAPGLPGKDAEYCKCPGREGDAGRSARRHRKFQL

>ROL-8A

MDVKETEEQRQMRRIAFVAVVVSTAAVIASVVTLPMLYNYVQSFQSHLMVETDYCKARSRDM**W**LEMTALQAGKGMGHRMKRAWLFGQWIPETSAGGGGSGNGGGNYGSGASTGGGANTAGYGGYGAAVNAEPAAVCCTCNQGAAGPPGPEGPPGNDGKDGRNGNDGKNGRDAEVLPAPASEPCIICPTGAPGPMGAMGPKGPPGPKGSPGEPPQDGKSGDDGMAGQPGPIGRPGRDGMKGAPGAAGRLIPVPGPQGAPGKPGPIGPPGPKGNPGPDGQSYQGPPGPPGDSGTPGHEGRAGPNGPAGPPGDNGEKGDCGHCPPPRTPPGY

>SQT-1

MSVKLACYVTASVTVATLMVCFMTMSTIYSEVDGFREKLDTEMNVFRQSTNGL**W**KDIVVIGRSSKRVRRQYEETNATPTPHADGSPSAPPGQPPAVPPVFNQPKTPNGANGNGPTCNCNADNKCPAGPSGPKGVPGVPGLDGVPGLDGVPGVGADDIAPQRESVGCFTCPQGPVGPPGALGRPGPRGLPGPRGQNGNPGRDGQPGHPGEQGSSGQIGKIGEPGPPGEKGRDAEHPIGRPGPKGPRGDQGPTGPAGQNGLHGPPGEPGTVGPEGPSGKQGRQGPDGTQGETGPDGRPGKDAEYCQCPDKSPPSEAVNANRGYRNI

>SQT-2

MMETSEHKELRRVAFFAIVVSTVAVIAAIVILPMLYSYVAGFQSHLIIEADFCKTRSRDM**W**AQIHDIDGPHLFHRQKRQYSSPNPPAAGGYGAPVTNSEPAPTCCSCQQGPAGPPGPPGDDGNGGQDGVRGNDGTDGKEGSLLESAIVNEPCIICPPGPPGPQGMAGAKGPQGPKGGNGDNGPDGKAGANGMQGPPGMMGPPGRQGVSGPKGAPGRINQINGPAGPAGHKGVRGPPGPRGEAGLDGGNSEGPQGPQGDAGRPGPVGEQGPQGPEGPQGPPGEPGGCEHCPIPRTPPGY

>SQT-3A/COL-1

METDGRLKAYKFVAYAAVGFSIAAVASVLLTLPMVYSYVSHVRQQMHHEINFCKGSAKDIFAEVNYMKANAGPVPPRNRTTRQAYGGPEVNPAPNLQCEGCCLPGPPGPAGAPGKPGKPGRPGAPGTPGTPGKPPVAPCEPTTPPPCKPCPQGPPGPPGPPGAPGDPGEAGTPGRPGTDAAPGSPGPRGPPGPAGEAGAPGPAGEPGTPAISEPLTPGAPGEPGDSGPPGPPGPPGAPGNDGPPGPPGPKGAPGPDGPPGVDGQSGPPGPPGPAGTPGEKGICPKYCAL**DGGVFFEDGTRR**
