# Supplementary material for: A systematic fluorescently tagged protein toolkit for dissecting apical extracellular matrix patterning in C. elegans
Source: PLoS Biol. 2026 Oct 14;24(10):e3004059. doi: 10.1371/journal.pbio.3004059 (PMC13705611; doi:10.1371/journal.pbio.3004059)
Supplement: Supplementary file 16 [file pbio.3004059.s029.docx]

RESPONSE TO REVIEWS:

Reviewer #1 (Jake Leyhr, identifies himself): In this manuscript, Ragle et al present the large-scale tagging of 102 C.elegans cuticular collagens and other aECM components, and describe their expression and localization in different substructures of the cuticle. After optimizing CRISPR methods and generating novel modular knock-in constructs, they selected cuticular collagens and other aECM components, prioritizing tagging those with known phenotypic information and high expression levels. Following this impressive tagging effort, they use Airyscan imaging to describe in beautiful detail the highly distinct localization of each protein. Most of the tagged strains described in the manuscript are already available from the CGC, and when the remaining ones become available, they represent a powerful advance for the ECM community.

Main comments:
*	The authors should correct the order of the numbered references to follow the order of the manuscript sections. For example, the very first reference in the introduction is currently numbered (5), while reference (1) first appears in the results section. I suggest the authors double-check all the references in case other references are out of order or misplaced. For example, the sentence "Estimates of the total number of protein species of the ECM, i.e. the matrisome,(11, 12) range from the hundreds to over 1000, many of which are subject to complex processing and post-translational modification." cites references 11 and 12, but reference 10 (Teuscher et al. 2019) is also highly relevant here, but doesn't appear for the first time until 3 paragraphs later.

**We have fixed the reference order.**

*	The authors describe a novel modular tagging system, introducing specific sgRNA sites on either side of the knock-in sequence so it can be efficiently swapped out for other tags by subsequent CRISPR injections. Two recent preprints have developed different modular tagging techniques (Han et al 2026; doi: 10.64898/2026.01.16.700006, Hefel et al 2026; doi: 10.64898/2026.01.15.699741) - the authors should discuss the pros and cons of their modular method in comparison with these.

**We have added a paragraph to the discussion on p.28, beginning ‘Several groups have recently reported....’**

*	The manuscript would benefit from additional schematic diagrams that show the anatomy of the cuticle layers. Figures 2F and 5F are helpful, but especially for understanding the distinction between WT and bli(0) cortical/basal morphologies, a 3D diagram showing the relative positioning of the cortical and basal layers in WT and bli(0) worms would be helpful to understand Figure 5B,C. Otherwise, the basal layers in bli(0) worms shown in Figure 5B could be mistaken for alae.

**We have created a new Figure 1 with a schematic of the cuticle and a representative collagen protein architecture. We have also added new labeling to the panels in Figure 7, a diagram to Fig 7F, and a new Figure S11 to better illustrate the morphology of the blistered phenotype.**

*	"In contrast, COL-80::mNG strut localization was abolished in bli-1(0) mutants and the remaining COL-80:mNG variably localized to annuli or furrows." - This should be shown in a panel of Figure 5 or a supplementary figure.
**We have added a new supplementary Figure S9 focusing on COL-80, as its localization is relatively complex.**

*	102 genes were successfully tagged for this project - can the authors include any targeted genes that were unsuccessful in generating viable knock-in strains, if there were any? It would be useful to guide future tagging efforts.

**We have added a section on ‘Editing efficiency and troubleshooting’ to Methods (p.34), and an additional Supplemental Table S1 describing failed edits. We stress that we did not extensively troubleshoot problematic cases, so an initial unsuccessful attempt could have multiple causes.**

*	The authors may wish to consider publishing some form of their imaging dataset alongside this manuscript. Table 1 is valuable, but even better would be a web resource that hosts many of the high-quality images the authors obtained to prepare it.

**We appreciate the comment. To minimize redundancy we are planning to contribute our data to the recently announced ‘WormtagDB’ online resource.**

Minor comments:
*	In the sentence "Such resources have also established the importance of studying ECM components using endogenous knock-in tags, consistent fluorescent protein (XFP) tags and a modular editing pipeline for stable and reproducible analysis of protein localization." XFP is defined but never used again in the manuscript - consider removing it.

**We have removed it**

*	In Figure S1A, the y-axis label is partially obscured.

**This correction has been made.**

*	In Figure S1B, is 2:3:1 supposed to read 2:1?

**This correction has been made.**

*	The authors should elaborate on the sentence "In contrast a pan-muscle mlc-1p::RFP co-injection marker enriched for edits (Figure S1F,G)" as it's not immediately clear to me how the data in S1F,G support it.

**We have added an addition sub figure (S1E) showing how the marker enriched for edits and clarified the description of these data. We apologize for the lack of clarity.**

*	"We successfully swapped mNG::FLAG to 2xOLLAS in 10 strains." should read "mSc::2xOLLAS"

**This correction has been made.**

*	In Figure 1, presumably the gray/blue/black boxes are the linker, FLAG, and OLLAS sequences, but this should be made clearer either in the figure itself or in the caption.

**This correction has been made.**

*	"we first examined a set of 27 cuticle genes initially defined by mutant phenotypes (17)." Table 1 should be referenced here, but it has 26 genes in bold, not 27. Reference 17 is cited for these 27 cuticle genes, but the genes bolded in Table 1 include some genes not mentioned in reference 17. Additional references should be cited to support the mutant phenotypes of col-121, lon-8, and ram-1.

**We have added the relevant references, which had been cited elsewhere.**

*	In Figure 4B, the quantification labels  V, VL, LD, DL, D are too close together and difficult to read. The abbreviations should also be explained in the caption.

**We have explained the abbreviations in the legend and a new paragraph in the Imaging Methods on p 36.**

*	The first panel in Figure 4D should have the text "DPY-5::mNG"

**Corrected**

*	The caption for Figure 5A mentions col-91 and col-125, but not the other panels - col-130, col-149, and rol-8. Is this deliberate?

**We have corrected the caption.**

*	"Notably, we got efficient editing with 35 nt homology arms with our ssDNA templates, whereas a previous report used 59-134 nt homology arms." - Cite this previous report.

**This correction has been made.**

*	"We then ran on an annealing program on a thermocycler" should read "We then ran an annealing program on a thermocycler"

**This correction has been made.**

*	In Table 1, the "puncta" note for acn-1 is in a cell that appears merged with bli-5 below it.

**Corrected**

*	The first sentence of the Molecular biology methods section is confusing. The authors describe 36bp "homology arms", but then say these encode flexible linkers that are both 13aa long. 36bp encodes 12aa not 13aa. Is the 36bp referring to the homology arms for Gibson assembly, and a different 39bp sequence encodes the linkers?

**We thank the reviewer for catching this error. We’ve corrected the linker length to 39 bp, which now matches the 13aa.**

*	In Supplemental Table 1, the strain PHX3691 is described as sqt-1::mNG(syb3691) II. However, in the CGC, the strain PHX3691 is listed as being sqt-3(syb3691[sqt-3::mNG(int)]) V. Which is correct - sqt-1 or sqt-3?

**Apologies, syb3691 is sqt-3.We have made the correction in the table.**

Reviewer #2: Via the tagging of a large number of apical ECM proteins, Ragle et al. report here in the very interesting paper the generation of a very useful and rich resource that will be very important to the ECM field. The strains and patterns reported here represent an invaluable starting point for future studies. In more general terms, the manuscript very nicely illustrates how protein tagging has the potential to reveal new biology and to pave the path for future discovery science. 

While I'm generally very enthusiastic about this manuscript, I do have one major request: To fully exploit and unleash the potential of this manuscript, the authors need to show more images of their protein localization patterns. As it stands the authors are quite focused on proteins that localize to the skin (perhaps not surprisingly, because that's what they are generally interested in), but it would be very useful - and, in fact, perhaps foundational for the research efforts. of others -  if the authors were to better report and document the non-skin patterns. I'm specifically referring to the "interstitial" and "other" category in Table 1. The authors presently only give the reader a very limited taste of such patterns in Figure 6, but they really should be showing more. I'm not requesting what, in fact, other papers with large surveys of expression patterns have done - to show every single one of their expression patterns in the Supp, but the author should really consider showing one representative for each pattern.

(1) I really would want to see the "other" patterns in the pharynx, gut, uterus and coloemocytes (9 reporters). Please show all patterns, except those where two proteins show the exact same.

**We have added a new supplementary Figure 4 showing representative non-cuticle localization patterns.**

(2) The interstitial patterns: those are generally very poorly described ("etc." as in the Table legend doesn't cut it). I totally understand that the authors may not be able to make "sense" of some of the patterns - but that's even more of a reason to show these patterns. As I said, many of these patterns have the potential to serve as very valuable tools for others in the community and may reveal novel biology. For example, folks studying the excretory system, would love to see the patterns for the excretory pore/duct. As it stands, any read of the manuscript would need to order >20 strains to see for themselves which of the interstitial pattern is in the duct/pore, which a strong, specific, etc. It's also not presently clear whether any of the patterns is specific just for one specific locale (i.e. are their patterns that are exclusive to, e.g., the deirids; or the pores).  Again, the authors to show all the patterns, except if two proteins show the exact same localization pattern.

**We have added a more detailed Table into Figure 9A summarizing interfacial localization for ~15 markers.**

For all of the images above - and also for the images in Fig.6 as they currently are - it would be good to see a low mag overview image of an entire worm so that the reader can see the specificity of the expression pattern. In my understanding PLoS Biology is very flexible with Figure number, so the authors should not shy away from going double digit with Figure numbers.
**We appreciate the comment. However given our limited resources we have chosen to focus this manuscript on high resolution imaging of microscale and nanoscale aECM patterning, which we feel is the major area of innovation opened up in our approach. Overall our current manuscript includes high resolution imaging of >65 distinct patterns or experiments and we hope this will be sufficient.**

A few additional comments:

- Is Table 1 incomplete? I haven't checked systematically, but I don't see col-34 listed, for example.

**col-34 is listed as ram-4.**

- please consider perhaps ordering the data in Table 1 in a different way. Right now it's alphabetical - it may be more useful to sort by pattern?

**We have added an Excel version of the Table to the Supporting Information, which readers can sort in a variety of ways.**

- Since much of the manuscript is about collagens, more "visual" background about their structural features may be useful. The authors describe some background on collagens in the Methods, but I would recommend to morph this into something more visual, into a Supp Fig.

**We have added a panel with cartoon of collagen structure, describing processing to Fig 1.**

- One very obvious question is which cells generate these proteins. For many patterns it's quite obvious (hypodermal cells), for other it's not quite as obvious (i.e. the interstitial patterns). Have the authors looked at scRNA databases to make some educated guesses/speculation about the source of the protein? 
**The cuticle collagens are primarily expressed in the epidermis or interfacial glia, based on scRNA datasets such as CENGEN. (We mostly check expression in cengen; other scRNA resources give similar results). We have added a citation on page 7: “Single cell transcriptomics (**[**https://www.cengen.org**](https://www.cengen.org)**) {Taylor, 2021 #116} indicates cuticle collagens are primarily expressed in the epidermis (seam cells or epidermal syncytia) or in interfacial glial cells.”**

**In the CENGEN scRNA data some collagens are detected at low levels in other tissues (neurons, muscle etc), however we were unable to detect protein corresponding to these tissues. We were also unable to detect cuticle collagen localization in intestine or somatic gonad. Such differences may reflect very low protein levels or low level contamination of the RNAseq source cells. Non-collagen proteins are expressed more widely; their protein localization is generally consistent with transcriptomics.**

Reviewer #3 (David Fay, signs review): The manuscript by Ragle et al., describes a major undertaking to endogenously tag and categorize the expression and localization of scores of apical extracellular matrix proteins in C. elegans, most of which correspond to the larval and adult worm cuticle. In addition to the immediate findings, this work represents a valuable resource to the community for future studies, specifically for those trying to understand the orchestration that underlies the deposition and remodeling of complex extracellular matrices. The tagging methods used will allow for the further modification of the generated strains, such as color swapping, and the studies provide some useful methodological data and strategies for the use of CRISPR in C. elegans. The strains and dataset also provide a useful comparator to several recent transcriptional studies implicating several thousand genes in the molting cycle, including many secreted components or regulators of the aECM. Collectively, this work represents a substantive advancement in itself and will facilitate the advancement of the ECM field. I am therefore enthusiastic about its publication.

My comments relate almost entirely to presentation and are organized by Figure and section. Although well written, I was confused in several sections, and I think that there could be improvements in the overall organization that will better enable readers to find relevant information. This is admittedly not the easiest dataset to organize, and I think the authors tried their best and had some good ideas, but the large number and similarity of gene names underscores the need to optimizing this aspect of the paper. My general suggestions are to increase the number of figures (PLoS Biology doesn't have a specific limit), to further subdivide the proteins and include sub-tables of relevant proteins in most figures, and to ensure that all descriptive statements in the text are appropriately supported by either figure panels or easily accessible sub-tables within each section. 

1) Fig 1 and associated. 
a) I wasn't very clear on some aspects of the CRISPR strategy descriptions and believe this section would benefit from some additional clarifications (Fig 1 and S1). There is some very good information and tools in this section so it's important that readers can grasp this easily and is consistent with the overall intent of the paper as a community resource.

**We have revised the text on pages 5-7 to clarify our editing optimization and hope that these data are now more accessible.**

b) For the legend for Fig 1 it would be helpful and consistent to move the (B) and (C) designations to the beginning of the relevant statements.

**This correction has been made.**

c) The transition from testing edit efficiency by targeting on GLH-1 versus the collagens (page 7 top) was abrupt or hard to follow.

**We have added: “We chose *glh-1* as a test case due to its use in optimization of editing efficiency and ease of scoring [31, 32]. ”**

d) There may also be some typos in Fig S1 or in the legend or it may just need more information; I was most confused when trying to parse results in S1E, F, and G. State what the dots represent etc.

**We have added text to the figure legend in Fig S1 to explain these data. “Each dot in the graphs A- D and F-H represent the parameter scored on the Y-axis in the F1 of a singled, injected P0 animal.”**

e) Some experiments (color swaps) were carried out using short homology arm PCR templates whereas others used plasmids with longer arms. I think this needs to be spelled out more clearly.

**We have added text to explain our experimental rationale.**

2) Fig 2 and associated. 
a) At the bottom of page 9 some comment about how the markers may have some effect on protein function as suggested by the double-marked strain would be appropriate here, although I realize that it is mentioned later.
b) The panels in Fig 2 weren't always precisely aligned and the lower images assigned to lower 2B could be made clearer.

**We have realigned all panels and reorganized Figure 2.**

c) In the legend, should DPY-13 be included in reference to panel C?

**We have removed it**

d) I was confused about "but it is unclear whether they also localize to the cortical layer" since the diagram and other data indicates furrow collagens localizing to the cortical layer.

**We apologize for the discrepancy. We have made revised cartoons to show furrow collagens as mostly basal. Based on EM morphology of furrows in nascent versus mature cuticles (e.g. Pooranachithra et al 2024 Fig 1D) we hypothesize that furrow collagens localize to the base of morphological furrows when they initially form in larval cuticles. At this stage the furrow extends almost to the base of the nascent cuticle, thus cortical and basal layers are compressed at the furrow. We suspect that as cuticle growth proceeds more cortical collagens are added in between the furrow base and the basally localized furrow collagens. In the adult, the cortical and basal layers are further pushed apart by expansion of the medial layer.**

e) I was unclear about the reason for the statement here that: "furrow' collagen localization does not precisely correlate with morphological furrows and instead may define distinct subregions within furrows.

**This was an attempt to account for the observation that morphological furrows extend to the lateral midline, whereas the furrow collagen labeling often does not extend to the midline.**

f) "Two other collagen knock-ins, DPY-5::mNG and DPY-13::mNG, were enriched in annuli…" Is it more accurate to say that "Two other collagen knock-ins… were enriched in the fibrous layers underlying the annuli…? Is it still part of the annuli if it is below the strut? Fig 2F seems to indicate annuli and furrows being present in the cortical layer specifically.

**The morphologically defined structures (furrows and annuli) are cortical, however collagens such as the furrow collagens and DPY-5/13 appear to be basal in the adult. We refer to their patterns (loosely) as furrow and annular as they correlate with the morphological structures in the xy plane although at different levels in the z axis. As mentioned above we suspect this reflects the function of the furrow and basal collagens at the earliest stage of a new cuticle.**

g) Fig S4 indicate gut granules in DPY-5 L1?

**We have labeled the gut granules.**

h) Throughout this section, such as on page 11, a number of localization/expression patterns are mentioned without a reference to a figure or table. Generally, if something is mentioned in this context one expects a corresponding figure or table reference, usually with an image, to accompany the statement. Conversely, from a statement on page 11, I was expecting DPY-17 to be shown in Fig 2E.

**We have revised the manuscript to ensure consistency between the genes mentioned in text and in Figures or Tables. For DPY-17, this had been previously reported and we have now added the citation to Birnbaum 2023.**

i) Fig S5A. I would suggest not doubling the intensities of the hets prior to plotting as I think most readers won't expect this and may misinterpret the data (as I did) prior to reading the legend.
**We elected to keep the Figure as-is, since this depicts the numbers used in the statistical analysis. We revised the associated text to make it clearer that intensities were scaled according to knock-in copy number: “**Interestingly, the fluorescence intensities of cuticle mNG of most knock-in heterozygotes (5/7), when scaled according to copy number, were significantly above those of homozygotes; 2/7 (*col-81*, *sqt-2*) were not significantly different (when scaled) from homozygote intensity”

3) Fig 3 and associated.
a) As for comment 2h, I found there to be some mildly confusing elements to this figure and text. From the text I thought that COL-149 would be shown in either Fig 3 or S6, but that was not the case. In the Fig 3 legend, it refers to Fig S3, but this reference was seemingly incorrect. The authors will want to go through all the figures and text to ensure consistency and correctness, but also to consider organizing the figures somewhat differently to make accessing the information as straightforward as possible.

**We have added more of the primary data so that all data are available for all markers mentioned in text.**

b) In the same vein, I kept going back to Table 1, which is a good summary, but the table spanned two pages, so I wasn't able to see the top row key on the second page. It would be helpful to have more smaller tables throughout (e.g., 5E) that group genes according to localization. This type of study could warrant 8-10 regular figures, versus the current six, which might help to distribute the data into bite-sized chunks and would allow the inclusion of more images that are referred to in the text.

**We have increased the number of figures to break up the data more. We have also included Table 1 as a supplemental file, so that readers can mine and sort as they seem fit. We considered breaking it up, but there is value in having all of the data together.**

c) Here (Fig 3F) and elsewhere, consider avoiding red/green images and text.
**We have converted all red-green double label images in the main Figures to cyan-magenta.**

4) Fig 4 and associated.
a) On page 16 COL-71 is referenced to Fig S8, but this figure shows COL-20 instead.

**We have corrected this to ‘COL-20’**

b) In 4A indicate inside panels which is inner and outer. Anything to reduce reliance on the legend, which didn't explicitly state which was which either, is helpful.

**We revised this Figure with better labeling of the fiber arrays.**

c) Define V, D, VL, DL, LD, etc. in 4B. Also decompress the text.

**We have defined these in the Figure 5 legend and in the Methods.**

Fig 5 and associated.
a) I like the idea of the table in 5E but was puzzled that quite a few of the proteins (COL-130, ROL-8, etc.) in the Fig 5 panels were not listed in 5E. For organization, it would be helpful to group these accordingly.

**We omitted COL-130 and ROL-8 from the Table (now 6E) as they are adult-specific (non-oscillating). We have revised this Table to more clearly separate the ‘oscillating’ collagens on the left (defined as in Meeuse 2023) versus adult-specific collagens (right part of Table).**

b) From the text statement on page 18 I was expecting some accompanying figure images regarding COL-80 expression in bli-1(0) mutants. As mentioned above, there were a number of instances where it seemed there could or should have been images following statements, but this was not always the case.

**We agree and have added a new supporting Figure (S9) to present the COL-80 pattern in more detail.**

c) Correlating localization with reported transcriptional timing is an interesting component of the work. Consider showing this in graphical form using the x axis for timing (earliest expression) and color coding the proteins by dots based on where they are expressed. This would highlight interesting anomalies as well.

**We attempted this however for reasons of space we have kept the tabular form, which we feel is closer to the data.**

Fig 6, associated, and Discussion.
a) On page 20 "RAM-1::mNG" seems to be missing from the sentence that beings with the word "localized".

**Corrected**

b) On page 24, I wasn't familiar with the meaning of "cluster D" classification to understand the point. Also, when looking up this reference elsewhere I noticed that it was missing on page 3.

**We have added a reference to Teuscher et al (Ref. 25) to the introduction.**

c) Any thoughts regarding the relative activities of fluorescent proteins in the cuticle? For example, there used to be some idea that superfolder GFP worked better than regular GFP, but it seems like the mNG works well too.

**In our initial tests of BLI-1 KIs we found mNG much brighter than regular GFP (Adams, Pooranachithra et al 2023), although for other aECM proteins the difference was less dramatic. Moreover the BM toolkit (Keeley et al 2020) has mostly used mNG. We do not have extensive experience with superfolder GFP, however a DPY-7::sfGFP single copy insertion (Xiaochen Wang lab) resembled other furrow collagen KIs in brightness. Preliminary results suggest mStayGold is often but not always either brighter or more photostable than mNG. Thus, our choice of mNG is primarily for consistency and ease of comparison; it may not be optimal in all cases.**

As for all my reviews this is signed by David Fay.

Reviewer #4: C. elegans has a collagen-based cuticle that is organized into multiple layers and three-dimensional substructures. By tagging many components of this apical matrix, Ragle et al provide a view of its amazing structural complexity and an explanation of why there are so many different cuticle collagens (180!) - each is expressed in different stages, tissues, and spatial patterns. Major cuticle substructures such as furrows, alae, and struts are shown to contain numerous different collagens that mark distinct nano-structures within (or adjacent) to them. Various orifices where tubes open to the outside environment also contain unique collagens. Components of the deep fibrous layers and of more cortical cuticle layers are also identified. This is a landmark study that contains many fascinating observations and provides tools that will be transformative for the C. elegans research community. The data also hint at levels of complexity that could be present in other apical ECMs and in various other collagen-based matrices.

Major comments

1. I appreciate that the authors are trying to stick to a high level summary style and not get bogged down in details, but there are many statements in the text that need to be more clearly backed up with data in the figures/legends/methods. e.g.
-Table 1 summary has some omissions/errors - information there based on imaging of how many animals per stage/tissue?

**We have added this information.**

-Claimed differences in localization of different furrow Dpy collagens need firmer evidence

**We have added two-color imaging of two of the furrow collagens (Fig 2D)**

-Use of blistered mutants to infer layer organization is hard to understand from the provided figure and has some caveats that should be acknowledged

**We have explicitly stated a caveat on page 20. “Thus, a caveat to this approach is that disruption of struts may affect cuticle organization beyond separation of cortical and basal layers.” In addition we now provide more double label data showing localization to distinct layers (Figure S12)**

-Various statements about specific collagens are made without citing a figure panel or table where the data are found (see detailed comments below)
**We have improved the citation of the data throughout such that all references to a gene in text have associated Figure panels.**

2. The data suggest some possible inconsistencies with prevailing models, but do not clearly resolve these issues. While it's not necessary (and probably not feasible) to resolve all of these issues here, it is important that the text and summary cartoons not imply evidence that doesn't exist. Instead, highlight possible inconsistencies and questions that will require follow-up. e.g. 

i. Do the 5 furrow collagens extend through both basal and cortical layers? Or are some exclusively basal and others cortical? (Text and figures imply different results, which is confusing, but the data seem inconclusive. Two color imaging might address this. Avoid drawing cartoons in a way that is inconsistent with your data)

**We agree and have remade these cartoons. In brief, furrow collagens appear mostly basal relative to the expanded medial layer. We appreciate that cartoons of the adult cuticle with clearly separated cortical and basal layers may be misleading as the cortical and basal layers are highly compressed in nascent larval furrows (likely where these collagens are acting to constrain furrow assembly). Two color labeling experiments (Figure S12) are also more consistent with furrow collagens being in the same z plane as basal collagens.**

ii. Do Roller mutations disrupt fibril orientations in the basal fibrous layers, as classically proposed? (not in any obvious way but needs further analysis, text handles this issue well)

**Indeed, our analysis to date suggests roller mutations may not simply alter fibril orientation as originally proposed (Cox et al 1980). Moreover many ‘Rol’ mutations are gain of function in collagens that we find do not normally localize to fibrous layers. We agree that extensive further analysis will be needed to resolve more clearly how Rol mutant collagens alter aECM architecture and believe this would be beyond the scope of the current work.**

iii. Are cortical collagens the first or last to be secreted and added to the cuticle? Or a mix of these? This relates to a central dogma about layered aECM - the idea that layers are built sequentially from the outermost to the innermost. I personally do not believe this dogma is correct. (Text leans towards dogma, expression data lean the other way, actual experiments not done but should be quite easy with the available fusions)
**We agree that the order of assembly of the aECM layers remains to be resolved and we have revised the manuscript to present both the prevailing hypothesis (‘dogma’) and more complex alternatives. We note that there is evidence for other C. elegans aECMs being assembled from outer to inner, e.g. the pharyngeal grinder aECM (Sparacio et al eLife 2020). See also response to point 17 below. We have added Figure 8 where we monitored the expression and localization of basal and cortical collagens, which supports the reviewer’s view that the “outside-in” model is not correct. We observed basal expressed collagens localize first and later, collagens that were either cortical or basal+cortical then localized to the cuticle.**

3. The authors could consider reorganizing some figures in a way that would allow summary cartoons to be more complete and accurate. e.g. it would be nice to have a complete summary cartoon indicating which collagens are in which basal fibrous layer (instead of just an incomplete cartoon in Fig. 2F before more data are shown in Fig. 4), another for furrows and furrow flanking patterns, another for struts. The existing summary cartoons in Figs. 5 and 6 are very effective but perhaps not complete.

**We have added summary cartoons to Figure 1 and 7.**

4. To be broadly accessible to non-experts and those outside of the C. elegans community, it would be helpful to provide a little more up-front background/explanation and to compare the worm collagens to mammalian collagens
-an early cartoon of cuticle layers and substructures (to be cited in Introduction) would help put subsequent text and figures in context

**We have added this to Figure 1A.**

-an early cartoon of a generic cuticle collagen would help introduce relevant domains/cleavage sites and justify that C-terminal tagging is a reasonable approach (whereas it wouldn't be in the most highly studied mammalian type I collagen)

**We added a cartoon of C. elegans collagen organization to Fig 1.**

-the worm must build at least most of its new cuticle before shedding the old one at the molt, so most cuticle assembly occurs during the prior stage (this is mentioned in the Discussion, but needed earlier in order to understand the whole discussion about cortical collagens).

**We have added the following to the introduction, p.3. “Each of the four larval stages displays a specific cuticle type that is initially formed in the preceding stage (e.g. L1 cuticle is assembled in the embryo, L2 cuticle is assembled in L1, and so on).”**

-the worm has 180 cuticle collagens, how many collagens do humans have? there are parallels in the tissue specificity and the fibril type distinctions
**We have added some text to the discussion on p28, beginning: ‘The multiscale patterning...’**

Specific comments, in order of Figures and text appearance

5. Table 1 RELATED TO POINT 1. Be clearer about the strength of the evidence supporting this table. Because I see some errors/omissions, I'm not confident that white boxes are truly evidence of absence (rather than just absence of data).
sqt-3::mNG is expressed in the embryo (Birnbaum et al 2023) and found in the cuticle of all stages - why does Table show it as adult-specific? dpy-6 is expressed in various interfacial tubes, including buccal cavity (Mazzoli et al biorxiv).

**We have corrected the boxes for sqt-3 and dpy-6.**

Some distinction between cuticle and pre-cuticle would be helpful here
**We have indicated known pre-cuticle genes e.g. noah-1,2.**

6. Fig. 1A would benefit from clearer labels and/or legend - blue box = FLAG, grey boxes = linkers, black box = 3' UTR?

**We have added clearer labels to the Figure as suggested by the reviewer.**

Mention the rationale for using mNeonGreen? I didn't see any citation for this fluorophore.

**We have added a legend and provided more rationale for using mNG.**

7. Fig. 1B,C,D could be moved to Fig. S1 with the rest of related methods information

**As other reviewers noted the value of the CRISPR optimization and reviewers encouraged us to expand the number of figures we decided to keep this CRISPR optimization data in to ensure visibility to the community.**

8. Fig. 2A (furrow collagens). RELATED TO POINT 1. I'm not seeing a clear difference between DPY-2 and DPY-3 here, despite claims in the text - both have some shorter lines and some longer ones. DPY-9 does look potentially different in extending less close to the seam, but this could be a focal plan difference. Any claimed difference here needs some more detail and numbers associated with it.

**We have cited the recent publication by Mazzoli et al 2026; the shorter lines of DPY-3 compared to DPY-2 are also visible in Mazzoli Fig 1D.**

9. Fig. 2E (DPY-14) It's worth pointing out that, like SQT-3 and DPY-17, this basal layer collagen is being secreted very early into the space between the embryo and the eggshell. Mazzoli et al (biorxiv) see a similar result with the furrow collagens, so this appears to be a widespread trend - L1 cuticle collagens are dumped into a slurry long before specific patterns emerge.

**We have added text to Fig 2E and cited Mazzoli 2026.**

10. Fig. 2F RELATED TO POINTS 1 AND 2. It is confusing that the legend (and Fig. 5E) says there is evidence that furrow collagens are basal (citation needed here), yet the cartoon here very explicitly indicates them as cortical. The striped pattern observed with the basal fibrous layer collagens is consistent with furrows or struts extending down into those layers (but that is not how it is drawn in the cartoon). It would be useful to do some 2-color imaging of furrow collagens with cortical/medial/basal collagens to resolve the relationships and redraw the cartoon accordingly. Or if you aren't going to address it, then maybe don't put the furrow collagen images in a figure that is emphasizing conclusions about layers! 
**We added a citation for basal localization of furrow collagens in Bli mutants (Adams, Pooranachithra 2023 Nat Comms).**

**We have also performed two color imaging as suggested, data now shown in Figure 2D and Figure S12. The results suggest a complex spatial relationship between the compartments: for example furrow marker DPY-9::mNG appears more basal than the fibrous layer marker DPY-5::mSc, but conversely the furrow marker DPY-10::mSc appears more cortical than the fibrous layer ROL-6::mNG (from L2 through adult). Overall our data are more consistent with furrow markers being basal in adults, however this is after the formation of the medial layer. As mentioned in response to R4 comment 2.i, the furrow regions in larval cuticle may be compressed relative to non-furrow regions, with less of a distinction between cortical and basal.**

11. Fig. 2F RELATED TO POINTS 1,2,3. It is confusing that the text and legend say that SQT-2 is non-fibrous, but the cartoon indicates it being in a fibrous layer - why? Explain how you determined that a collagen is in the "outer fibrous" or "inner fibrous" layer. Features of these layers are not explained until p. 16 and Fig. 4. I kept having to go back and forth between these sections.

**We have tried to clarify the cartoon. SQT-2 appears to localize basally but does not form visible fibers so we classify it as non-fibrous. Our data do not resolve whether these non-fibrous annular markers are in the same layers as the fibrous layers or in another basal layer. For the distinction between outer and inner fibrous layers, this was based on handedness (outer = right-handed helix, inner = left-handed).**

12. Fig. 3B and 3C are showing an intriguing alae defect in dpy-3 mutants - worth mentioning, especially given that this furrow collagen is not itself observed in the alae.

**We have added a note to the Figure 4 legend that dpy-3 mutants have alae defects (which we confirmed in DIC) and have cited Cox et al 1980 who also reported alae defects in ‘furrow’ collagen mutants.**

13. Fig. 3 legend citation to Figure S3 is an error, I think you mean S6

**Corrected.**

14. Fig. 4B Define the abbreviations at graph bottom. Legend statement about chirality should cite Fig. S8 and ref. 57.

**We have defined the abbreviations in the Figure legend (now Fig 6) and have added further explanation in Methods. We have added the citations.**

15. Fig. 5B-D RELATED TO POINT 1. It's very hard to understand how the collagen patterns relate to blisters since we can't see the blisters in any of these images. e.g. how can you tell that COL-91 is cortical from this image?? Please provide bright field images if available.

**We have tried to better illustrate the blistering morphology in the data. Blister morphology is visible when confocal gain is turned up to visualize the faint autofluorescence, or in a complete z series (see COL-80 Figure S9), however this saturates the collagen signal so we have not shown these images for reasons of space.**

16. Fig. 5 As the authors point out, struts may do more than simply hold the cortical and basal layers together - they may actually influence layer organization or integrity. In that case, one could be misled about normal layer localization by inferring it in these mutant backgrounds. Another independent confirmation of the layer assignments would be to do two color imaging of putative cortical vs. basal components. In the absence of that, at least acknowledge caveats.

**We have acknowledged the caveat that blister mutants may have complex effects on aECM organization.**

17. Fig. 5E RELATED TO POINT 2. Please indicate the Meeuse molt time on this table (or in legend) - it will run right through the middle of the cortical collagen phases. Leaving this out is misleading as people will assume that molt is at the bottom of the table. The data don't fit the prevailing model of cortical collagens being the earliest secreted, but that is one of things that is exciting here and needs follow-up.

**We agree and have revised this Table using ‘molt aligned’ transcript peak phases (Gaidatzis et al 2025) relative to ecdysis at 0°. Indeed, some cortical collagen transcripts peak very late, but others peak very early, as we stated on page 19 of the original ms. We have expanded the discussion to present two possible models: (1) all or most cortical collagens expressed early in the molt cycle (the “dogma”, i.e. prevailing hypothesis), and (2) cortical collagens are expressed in more than one wave, with early ones (e.g. col-80) perhaps forming an initial cortical layer, and late ones (e.g. col-12) forming internal cortical layers after formation of a basal layer. Indeed, in the available EM imaging of nascent cuticles (e.g. Pooranachithra 2024) the cortical layer appears to forms gradually i.e. a thin cortical layer is present on top of the basal layer, but presumably expands later. We do not have a mechanistic explanation of how late-expressed collagens might be targeted cortically; it is possible that the basal layers are porous and allow collagen movement. We agree that the precise order of formation of the various compartments is an exciting area for future study.**

Several of the listed cortical collagens appear to be expressed only in adult according to Meeuse et al 2020, which would mean they must be added on top of the existing cuticle. This should be easy to confirm or refute by imaging your tagged collagen strains at the molt, and would be a very important result!

**We have tried to clarify that the distinction of oscillating vs non-oscillating (stage-specific) was based on Meeuse et al. 2023 which differs slightly from the original Meeuse 2020 analysis. Several ‘adult specific’ collagens appear to be first transcribed in late L4 (presumably incorporated into nascent adult cuticle) but their transcripts may peak in the adult.**

**In some cases (e.g. COL-71, COL-80, COL-81) a collagen was classified as oscillating based on transcriptomics but we find the protein does not accumulate except in late L4 or adult. For example COL-81::mNG transcripts are detectable in L4 but protein is not seen until adult (and is basal). Possibly these are regulated post transcriptionally. We have now separated these in the Table from those clearly expressed at protein level in multiple larval stages. COL-80 is of particular interest as its transcription begins in L4 but is highest in young adults; we have included images of COL-80 in new Fig S9.**

18. Fig. 5F RELATED TO POINT 3. Why are several collagens listed as cortical in 5E but then not shown here in 5F? (col-12, col-63, col-80, col-81, col-98) - what is the basis for inclusion/exclusion?

**We have clarified that Figure 5 images (now Figure 7) focus on the ‘annular cortical’ subset of markers. (The COL-63, COL-81, COL-98 patterns are shown in detail in Fig 5). Patterns very similar to other markers were excluded for reasons of space/readability.**

19. Fig. S9 This should be a main figure! 
**We appreciate the reviewer’s enthusiasm. However as we do not have super resolution imaging for all the data points shown here we have elected to keep it as Supplemental (now Fig S13).**

20. Fig. S10 This is a cool result but it is very hard to understand what we are looking at here. The fluorescent images need accompanying bright field images or at least some cartoons to help orient us.
**We have added brightfield images of the COL-135::mNG imaging.**

21. Figure scale bars are sometimes missing, sometimes labelled with sizes, sometimes not, sometimes explained in legend, sometimes not. (panels without scale information include 2D, 3F, 5B) 
**We have made the scale bars consistent**

22. p. 4 In addition to ref 26 (which is about glial sockets), other interfacial tube matrix citations include ref. 61 or Cohen et al 2020 (vulva), Kelley et al 2015 (buccal cavity), and Gill et al 2016 (excretory duct/pore).

**We agree these are additional examples that further make the point. We have added the reference to vulva. With regard to the Kelley 2015 paper on FBN-1 it is not clear if FBN-1 is specifically localized to interfacial matrix as it is widely distributed over the epidermis.**

23. p. 5 Here we report a protein-tagging resource of knock-in strains for 102 aECM components…
For select genes, we generated an additional mScarlet(mSc) red FP-tagged versions to facilitate co-localization studies - citeTable S1

**We have cited in the results.**

24. p. 8 We next prioritized collagens based on expression as described above. The above text is a dense description of methods that doesn't say anything about collagen expression as far as I could see. The text vaguely implies, but should say more explicitly, that you screened for edits based on visible fluorescence.

**We have reworded: “We focused on secreted protein families likely to comprise stable components of the aECM [10, 43] and which were robustly expressed in public RNAseq datasets” (p.7)**

25. p. 9 To test whether aECM components tolerate mNG tagging we first examined a set of 27cuticle genes initially defined by mutant phenotypes (17). Knock-ins for four of these had been previously reported (e.g. bli-1, bli-2, bli-6, dpy-13) (7) - actually should be 6 since knock-ins of SQT-3 and DPY-17 were reported in Birnbaum et al 2023, though not analyzed for adult patterns there.
**Corrected**

26. p. 9 Knock-in strains for such genetically defined cuticle genes were morphologically normal and displayed bright fluorescence in the cuticle. This is not entirely true according to later paragraph p. 12 (at least 4 strains had mutant phenotypes, more if you count genetic interactions).

**Reworded as ‘most knock-in strains...’**

27. RELATED TO POINT 1. p. 9 Within this set, individual patterns differed in detail. DPY-2::mNG showed the most consistent labeling, extending around the circumference to the lateral midline or lateral alae, without breaks (Figure 2A). DPY-3::mNG furrows often did not extend to the lateral midline. Clarify what is the evidence behind these statements, because this distinction is not apparent from the images shown. In both strains I see some furrows that don't extend to the mid-line.

**We have clarified in the Fig legend. Our conclusion is that none of the larvally expressed ‘furrow’ collagen KIs perfectly correspond to furrows as defined by DIC morphology. In contrast, some adult furrow-localizing collagens e.g. col-81 consistently extend all the way to alae. Possibly the embryonic/larval furrow collagens are more restricted and/or affect furrow extension to midline. Slight differences between furrow collagen localizations are also visible in published data (Mazzoli 2026). We have noted this in passing, as it is unclear if it reflects a true distinction between the endogenous collagen distributions vs difference in tolerance for mNG tagging.**

28. RELATED TO POINT 1. p. 9, Interestingly, BIS-1/COL-121, identified on the basis of permeability barrier defects (56),also localized to furrows specifically in embryonic and L1 stages - Table or Figure citation for these data?

**We have added COL-121 images to Figure 4.**

29. RELATED TO POINT 4. p. 11-12 mentions dibasic cleavage sites completely lacking any context; the fact that worm collagens are cleaved at a dibasic site isn't introduced until the next paragraph.

**We have introduced the cleavage sites earlier.**

30. RELATED TO POINT 4. p. 12 An important question at the outset was whether collagens readily tolerate tagging. Yes, so why not discuss it at the outset? Many readers will find it surprising that you are tagging collagens at their C-terminus, since C-terminal cleavage is such an important part of human Type I collagen regulation.

**We have added a sentence at end of first paragraph of results: “In most cases we initially inserted a tag at the C-terminus unless there was evidence for C-terminal processing (e.g. SQT-3) [41]; as discussed further below, C-terminal tagging appeared to be broadly tolerated.”**

31. p. 12 C. elegans cuticle collagens undergo N-terminal cleavage by subtilisin proteases... Citations needed for this statement. I don't see a citation for Thacker et al 1995 anywhere in the manuscript.

**We have added a citation to Thacker.**

32. RELATED TO POINT 1. p. 12. Several other collagens we tagged contain similar C-terminal cleavage sites, however C-terminal knock-ins displayed cuticle localization. Which other collagens specifically are you referring to? Citation of a methods section, figure or table for these data?

**At least 30 cuticle collagens have C-terminal DGG cleavage sites; we have named some and have added a supporting File with all collagen sequences annotated.**

33. p. 12 Of the [27] cuticle collagens defined by mutant phenotype we detected phenotypes for only three [FOUR or FIVE?] knock-in strains. The DPY-4::mNG and DPY-8::mNG KI strains displayed mild Dpy phenotypes whereas RAM-4/COL-43::mNG and RAM-2/COL-82::mNG KI males displayed Ram phenotypes, suggesting these insertions affect gene function....

**Corrected to four (counting overt phenotypes).**

34. RELATED TO POINT 1. p. 18. In contrast, COL-80::mNG strut localization was abolished in bli-1(0) mutants and the remaining COL-80:mNG variably localized to annuli or furrows. Figure or Table citation for these data?
**We have added the new COL-80::mNG Figure S9.**

35. p. 18 Thus, in some cases, disruption of struts may affect cuticle organization beyond separation of cortical and basal layers. If struts influence layer organization/integrity, then one could be misled about normal layer localization by inferring it in these mutant backgrounds.

**We have emphasized this caveat. Prior analyses of bli-1 suggests it is very specific to struts and that other layers are largely normal prior to their separation in early adulthood, however there could be secondary effects.**

36. RELATED TO POINTS 2, 4. pp 19-20: This timing would suggest the cortical collagens are expressed either just before the molt, presumably for construction of the cuticle early in the next larval stage, or right after the molt. As it is unclear how cortical collagens would be assembled outside basal layers, we favor the interpretation that the cortical collagen genes are expressed very early in the cyclic synthesis of each aECM. Then how do you explain col-63 and col-81, which don't fit this model? Meeuse puts ecdysis around 135˚, which would suggest some cortical collagens are expressed very early but others are expressed very late. This section feels like you are trying to fit the data to the dogma instead of acknowledging inconsistencies.

**We agree there may be exceptions to the general trend, however col-63 is not an exception as it is adult specific. Although col-81 transcript was reported by Meeuse as oscillating we find that the protein is adult specific. We have expanded the discussion to include two possible models for the expression of cortical collagens. (See also response to point 17).**

37. p. 20: [? collagen name missing?] localized exclusively to male tail rays,
**Corrected to RAM-1**

38. p. 20: in addition to not seeing the proposed intestinal expression of cuticle collagens, do you care to comment on recent claims for distal tip cell expression of such collagens?
**We have added a comment on page 22-23. Indeed, Stolzenbach et al 2026 have tested several of the collagen knock-in markers reported in this work, however they did not detect KI expression in the ECM of dissected gonads. The only marker visible in such gonads was the overexpressed COL-12 transgene (kaIs12). Thus, collagens of the apical ECM of the epidermis may be at most minor components of other ECMs.**

39. RELATED TO POINT 2. p. 24 Nevertheless, our analysis suggests that cortically localized proteins tend to have early transcript peaks whereas basal proteins have later peaks. I disagree strongly with this assessment. Several of the listed cortical collagens appear to be expressed only in adult according to Meeuse et al 2020, which would mean they must be added on top of the existing cuticle. This should be easy to confirm or refute by imaging your tagged collagen strains.

**See response to point 17.**

40. p. 24 Tagging of Hedgehog-related proteins has also revealed specific localization to substructures of the cuticle or precuticle (79). Ref 26 also relevant here, along with Chiyoda et al 
**We have added the citations.**

41. p. 30 citation to Figure 1, specify panel(s) and or move to Fig. S1. Description of SAP Trap methods should cite Fig. S3.
**This correction has been made.**

42. p. 32 Description of unc-119 selection method should cite Fig. S2.

**This correction has been made.**

43. p. 33 citations to Figure 2 appear to be in error - data are in Fig. 1 and Fig. S1

**This correction has been made.**

44. RELATED TO POINT 1. p. 33 Approximately 35 collagens have potential C-terminal cleavage sites resembling that of SQT-3 (DGG motif) including BLI-6, DPY-4, DPY-13, ROL-1, and LON-3. Provide a citation or data (e.g. a sequence alignment) to back up this statement.
**This statement was based on our own manual annotation of collagen sequences in Wormbase. We have added a supplementary .doc file (File S1) with the annotated sequences.**
