# Supplementary material for: A systematic fluorescently tagged protein toolkit for dissecting apical extracellular matrix patterning in C. elegans
Source: PLoS Biol. 2026 Oct 14;24(10):e3004059. doi: 10.1371/journal.pbio.3004059 (PMC13705611; doi:10.1371/journal.pbio.3004059)
Supplement: Supplementary file 17 [file pbio.3004059.s030.docx]

Dear Dr Ward,

Thank you for your patience while we considered your revised manuscript "Multiscale patterning of a model apical extracellular matrix revealed by systematic endogenous protein tagging" for publication as a Methods and Resources Article at PLOS Biology. This revised version of your manuscript has been evaluated by the PLOS Biology editors and the Academic Editor.

Based on our Academic Editor's assessment of your revision, we are likely to accept this manuscript for publication, provided you address the following data and other policy-related requests that I have provided below (A-E). Please also ensure that you double check the figure legends, experimental methods and the supplemental material to ensure everything is correct at this stage.

(A) We routinely suggest changes to titles to ensure maximum accessibility for a broad, non-specialist readership. In this case, we suggest a minor edit to the title, as follows, to further bring out the Resource aspect of the study. Please ensure you change both the manuscript file and the online submission system, as they need to match for final acceptance:

“A systematic fluorescently tagged protein toolkit for dissecting apical extracellular matrix patterning in C. elegans”

>>That title looks good to us. We will change it.

(B) You may be aware of the PLOS Data Policy, which requires that all data be made available without restriction: <https://www.google.com/url?q=http://journals.plos.org/plosbiology/s/data-availability&source=gmail-imap&ust=1790603136000000&usg=AOvVaw1ChbwagsEzCjD-sD2o9Tat>. For more information, please also see this editorial: <https://www.google.com/url?q=http://dx.doi.org/10.1371/journal.pbio.1001797&source=gmail-imap&ust=1790603136000000&usg=AOvVaw0DURNydP-mpaiWdLCw_IZB>

Note that we do not require all raw data. Rather, we ask that all individual quantitative observations that underlie the data summarized in the figures and results of your paper be made available in one of the following forms:

-Supplementary files (e.g., excel). Please ensure that all data files are uploaded as 'Supporting Information' and are invariably referred to (in the manuscript, figure legends, and the Description field when uploading your files) using the following format verbatim: S1 Data, S2 Data, etc. Multiple panels of a single or even several figures can be included as multiple sheets in one excel file that is saved using exactly the following convention: S1_Data.xlsx (using an underscore).

-Deposition in a publicly available repository. Please also provide the accession code or a reviewer link so that we may view your data before publication. 

Regardless of the method selected, please ensure that you provide the individual numerical values that underlie the summary data displayed in the following figure panels as they are essential for readers to assess your analysis and to reproduce it:

Figure 6B, S1A-H, S6A-B
>>We have added four supplemental spreadsheets with these data.
NOTE: the numerical data provided should include all replicates AND the way in which the plotted mean and errors were derived (it should not present only the mean/average values).


(C) Please also ensure that each of the relevant figure legends in your manuscript include information on *WHERE THE UNDERLYING DATA CAN BE FOUND*, and ensure your supplemental data file/s has a legend.
>>We have added information about where to find the data to the legends and there are legends for the supplemental data files.

(D) Per journal policy, if you have generated any custom code during the course of this investigation, please make it available without restrictions. Please ensure that the code is sufficiently well documented and reusable, and that your Data Statement in the Editorial Manager submission system accurately describes where your code can be found. More information on our Code Policy, what and how to share can be found here: <https://www.google.com/url?q=https://journals.plos.org/plosbiology/s/code-availability&source=gmail-imap&ust=1790603136000000&usg=AOvVaw3cDSXJO8GO3ZeIcnubedPK>

Please note that we cannot accept sole deposition of code in GitHub, as this could be changed after publication. However, you can archive this version of your publicly available GitHub code to Zenodo. Once you do this, it will generate a DOI number, which you will need to provide in the Data Accessibility Statement (you are welcome to also provide the GitHub access information). See the process for doing this here: <https://www.google.com/url?q=https://docs.github.com/en/repositories/archiving-a-github-repository/referencing-and-citing-content&source=gmail-imap&ust=1790603136000000&usg=AOvVaw264nzOdTqB8NTfu1039PG_>

>>We did not generate any new code.

(E) Please ensure that you are using best practice for statistical reporting and data presentation. These are our guidelines <https://www.google.com/url?q=https://journals.plos.org/plosbiology/s/best-practices-in-research-reporting%23loc-statistical-reporting&source=gmail-imap&ust=1790603136000000&usg=AOvVaw3hG5Xb9wsqf7x1XXNE-GLg> and a useful resource  on data presentation <https://www.google.com/url?q=https://journals.plos.org/plosbiology/article?id%3D10.1371/journal.pbio.1002128&source=gmail-imap&ust=1790603136000000&usg=AOvVaw39ETb6i9G8NPiV3uGRoQl_>

- If you are reporting experiments where n ≤ 5, please plot each individual data point.

>>We have reviewed these documents and done our best to adhere to the guidelines.

------------------------------------------------------------------------

As you address these items, please take this last chance to review your reference list to ensure that it is complete and correct. If you have cited papers that have been retracted, please include the rationale for doing so in the manuscript text, or remove these references and replace them with relevant current references. Any changes to the reference list should be mentioned in the cover letter that accompanies your revised manuscript.

In addition to these revisions, you may need to complete some formatting changes, which you will receive in a follow up email. A member of our team will be in touch with a set of requests shortly. If you do not receive a separate email within a few days, please assume that checks have been completed, and no additional changes are required.

We expect to receive your revised manuscript within two weeks. 

To submit your revision, please go to <https://www.google.com/url?q=https://www.editorialmanager.com/pbiology/&source=gmail-imap&ust=1790603136000000&usg=AOvVaw0nUvPkFFkRfm0XGsmUBWfq> and log in as an Author. Click the link labelled 'Submissions Needing Revision' to find your submission record. Your revised submission must include the following:
-  a cover letter that should detail your responses to any editorial requests, if applicable, and whether changes have been made to the reference list
-  a Response to Reviewers file that provides a detailed response to the reviewers' comments (if applicable, if not applicable please do not delete your existing 'Response to Reviewers' file.)
-  a track-changes file indicating any changes that you have made to the manuscript. 

NOTE: If Supporting Information files are included with your article, note that these are not copyedited and will be published as they are submitted. Please ensure that these files are legible and of high quality (at least 300 dpi) in an easily accessible file format. For this reason, please be aware that any references listed in an SI file will not be indexed. For more information, see our Supporting Information guidelines:
<https://www.google.com/url?q=https://journals.plos.org/plosbiology/s/supporting-information&source=gmail-imap&ust=1790603136000000&usg=AOvVaw1HpbzXFnuWzTMcJ0fkZx6Z>  

*Published Peer Review History*
Please note that you may have the opportunity to make the peer review history publicly available. The record will include editor decision letters (with reviews) and your responses to reviewer comments. If eligible, we will contact you to opt in or out. Please see here for more details:
<https://www.google.com/url?q=https://plos.org/published-peer-review-history/&source=gmail-imap&ust=1790603136000000&usg=AOvVaw2wT3XmQKRLtRJttqbesHuL>

*Press*
Should you, your institution's press office or the journal office choose to press release your paper, please ensure you have opted out of Early Article Posting on the submission form. We ask that you notify us as soon as possible if you or your institution is planning to press release the article.

*Protocols deposition*
To enhance the reproducibility of your results, we recommend that if applicable you deposit your laboratory protocols in protocols.io, where a protocol can be assigned its own identifier (DOI) such that it can be cited independently in the future. Additionally, PLOS ONE offers an option for publishing peer-reviewed Lab Protocol articles, which describe protocols hosted on protocols.io. Read more information on sharing protocols at <https://www.google.com/url?q=https://plos.org/protocols?utm_medium%3Deditorial-email%26utm_source%3Dauthorletters%26utm_campaign%3Dprotocols&source=gmail-imap&ust=1790603136000000&usg=AOvVaw2BeC9lqp312JuejM9RbiZ2>


Please do not hesitate to contact me should you have any questions.

Best wishes,

Richard

Richard Hodge, PhD
Senior Editor, PLOS Biology
[rhodge@plos.org](mailto:rhodge@plos.org)

Journal Requirements:

1) Please ensure that the funders and grant numbers match between the Financial Disclosure field and the Funding Information tab in your submission form. Note that the funders must be provided in the same order in both places as well.
 Currently, "P40 OD010440" is missing from the Funding Information 

2) Please refer to the following article for PLOS Biology's best-practice recommendations for data presentation: <https://www.google.com/url?q=https://journals.plos.org/plosbiology/article?id%3D10.1371/journal.pbio.1002128&source=gmail-imap&ust=1790655288000000&usg=AOvVaw3iUWEHWyD7_NlEg2-oM0xI>. 
Guidelines for statistical reporting, if relevant, may be found here: <https://www.google.com/url?q=https://journals.plos.org/plosbiology/s/best-practices-in-research-reporting%23loc-statistical-reporting&source=gmail-imap&ust=1790655288000000&usg=AOvVaw3Qk6xh21OzNjE-c-53M8MG>
