# Supplementary material for: A systematic fluorescently tagged protein toolkit for dissecting apical extracellular matrix patterning in C. elegans
Source: PLoS Biol. 2026 Oct 14;24(10):e3004059. doi: 10.1371/journal.pbio.3004059 (PMC13705611; doi:10.1371/journal.pbio.3004059)
Supplement: Peer review history

# Editor report: Decision Letter 0

**DOI:** 10.1371/journal.pbio.3004059.r001

**Authors:** Richard Hodge

16 Mar 2026

Dear Dr Ward,

Thank you for submitting your manuscript entitled "Multiscale patterning of a model apical extracellular matrix revealed by systematic endogenous protein tagging" for consideration as a Methods and Resources Article by PLOS Biology.

Your manuscript has now been evaluated by the PLOS Biology editorial staff, as well as by an academic editor with relevant expertise, and I am writing to let you know that we would like to send your submission out for external peer review.

However, before we can send your manuscript to reviewers, we need you to complete your submission by providing the metadata that is required for full assessment. To this end, please login to Editorial Manager where you will find the paper in the 'Submissions Needing Revisions' folder on your homepage. Please click 'Revise Submission' from the Action Links and complete all additional questions in the submission questionnaire.

Once your full submission is complete, your paper will undergo a series of checks in preparation for peer review. After your manuscript has passed the checks it will be sent out for review. To provide the metadata for your submission, please Login to Editorial Manager (https://www.editorialmanager.com/pbiology) within two working days, i.e. by Mar 18 2026 11:59PM.

If your manuscript has been previously peer-reviewed at another journal, PLOS Biology is willing to work with those reviews in order to avoid re-starting the process. Submission of the previous reviews is entirely optional and our ability to use them effectively will depend on the willingness of the previous journal to confirm the content of the reports and share the reviewer identities. Please note that we reserve the right to invite additional reviewers if we consider that additional/independent reviewers are needed, although we aim to avoid this as far as possible. In our experience, working with previous reviews does save time.

If you would like us to consider previous reviewer reports, please edit your cover letter to let us know and include the name of the journal where the work was previously considered and the manuscript ID it was given. In addition, please upload a response to the reviews as a 'Prior Peer Review' file type, which should include the reports in full and a point-by-point reply detailing how you have or plan to address the reviewers' concerns.

During the process of completing your manuscript submission, you will be invited to opt-in to posting your pre-review manuscript as a bioRxiv preprint. Visit http://journals.plos.org/plosbiology/s/preprints for full details. If you consent to posting your current manuscript as a preprint, please upload a single Preprint PDF.

Feel free to email us at plosbiology@plos.org if you have any queries relating to your submission.

Kind regards,

Richard

Richard Hodge, PhD

Senior Editor, PLOS Biology

rhodge@plos.org

PLOS

Empowering researchers to transform science

Carlyle House, Carlyle Road, Cambridge, CB4 3DN, United Kingdom

California (U.S.) corporation #C2354500, based in San Francisco

# Peer review: Decision Letter 1

**DOI:** 10.1371/journal.pbio.3004059.r002

**Authors:** Richard Hodge

7 Apr 2026

Dear Dr Ward,

Thank you for your patience while your manuscript "Multiscale patterning of a model apical extracellular matrix revealed by systematic endogenous protein tagging" was peer-reviewed at PLOS Biology. It has now been evaluated by the PLOS Biology editors, an Academic Editor with relevant expertise, and by four independent reviewers.

In light of the reviews, which you will find at the end of this email, we would like to invite you to revise the work to thoroughly address the reviewers' reports.

As you can see, the reviewers are all enthusiastic and positive about the study, noting that the resource will be useful for the community. However, they all raise overlapping concerns that are mostly textual and presentational in nature. This includes improving the organization of the manuscript and figures, adding additional explanations for the tagging strategy and ensuring statements in the text are sufficiently supported by the data. In addition, Reviewer #2 asks that additional documentation of proteins that do not localize to the skin (‘other’ and ‘interstitial’) are provided. After discussions with the academic editor, we agree that this is an important suggestion given the resource format of the study and consider this essential for the revision.

Given the extent of revision needed, we cannot make a decision about publication until we have seen the revised manuscript and your response to the reviewers' comments. Your revised manuscript is likely to be sent for further evaluation by all or a subset of the reviewers.

In addition to these revisions, you will need to complete some formatting changes, which you will receive in a follow up email. A member of our team will be in touch with a set of requests shortly.

We expect to receive your revised manuscript within 3 months. Please email us (plosbiology@plos.org) if you have any questions or concerns, or would like to request an extension.

At this stage, your manuscript remains formally under active consideration at our journal; please notify us by email if you do not intend to submit a revision so that we may withdraw it.

**IMPORTANT - SUBMITTING YOUR REVISION**

Your revisions should address the specific points made by each reviewer. Please submit the following files along with your revised manuscript:

1. A 'Response to Reviewers' file - this should detail your responses to the editorial requests, present a point-by-point response to all of the reviewers' comments, and indicate the changes made to the manuscript.

*NOTE: In your point-by-point response to the reviewers, please provide the full context of each review. Do not selectively quote paragraphs or sentences to reply to. The entire set of reviewer comments should be present in full and each specific point should be responded to individually, point by point.

You should also cite any additional relevant literature that has been published since the original submission and mention any additional citations in your response.

2. In addition to a clean copy of the manuscript, please also upload a 'track-changes' version of your manuscript that specifies the edits made. This should be uploaded as a "Revised Article with Changes Highlighted" file type.

*Re-submission Checklist*

When you are ready to resubmit your revised manuscript, please refer to this re-submission checklist: https://plos.io/Biology_Checklist

To submit a revised version of your manuscript, please go to https://www.editorialmanager.com/pbiology/ and log in as an Author. Click the link labelled 'Submissions Needing Revision' where you will find your submission record.

Please make sure to read the following important policies and guidelines while preparing your revision:

*Published Peer Review*

Please note while forming your response, if your article is accepted, you may have the opportunity to make the peer review history publicly available. The record will include editor decision letters (with reviews) and your responses to reviewer comments. If eligible, we will contact you to opt in or out. Please see here for more details:

https://blogs.plos.org/plos/2019/05/plos-journals-now-open-for-published-peer-review/

*PLOS Data Policy*

Please note that as a condition of publication PLOS' data policy (http://journals.plos.org/plosbiology/s/data-availability) requires that you make available all data used to draw the conclusions arrived at in your manuscript. If you have not already done so, you must include any data used in your manuscript either in appropriate repositories, within the body of the manuscript, or as supporting information (N.B. this includes any numerical values that were used to generate graphs, histograms etc.). For an example see here: http://www.plosbiology.org/article/info%3Adoi%2F10.1371%2Fjournal.pbio.1001908#s5

*Blot and Gel Data Policy*

We require the original, uncropped and minimally adjusted images supporting all blot and gel results reported in an article's figures or Supporting Information files. We will require these files before a manuscript can be accepted so please prepare them now, if you have not already uploaded them. Please carefully read our guidelines for how to prepare and upload this data: https://journals.plos.org/plosbiology/s/figures#loc-blot-and-gel-reporting-requirements

*Protocols deposition*

To enhance the reproducibility of your results, we recommend that if applicable you deposit your laboratory protocols in protocols.io, where a protocol can be assigned its own identifier (DOI) such that it can be cited independently in the future. Additionally, PLOS ONE offers an option for publishing peer-reviewed Lab Protocol articles, which describe protocols hosted on protocols.io. Read more information on sharing protocols at https://plos.org/protocols?utm_medium=editorial-email&utm_source=authorletters&utm_campaign=protocols

Thank you again for your submission to our journal. We hope that our editorial process has been constructive thus far, and we welcome your feedback at any time. Please don't hesitate to contact us if you have any questions or comments.

Best regards,

Richard

Richard Hodge, PhD

Senior Editor, PLOS Biology

rhodge@plos.org

------------------------------------

REVIEWS:

Reviewer #1 (Jake Leyhr, identifies himself): In this manuscript, Ragle et al present the large-scale tagging of 102 C.elegans cuticular collagens and other aECM components, and describe their expression and localization in different substructures of the cuticle. After optimizing CRISPR methods and generating novel modular knock-in constructs, they selected cuticular collagens and other aECM components, prioritizing tagging those with known phenotypic information and high expression levels. Following this impressive tagging effort, they use Airyscan imaging to describe in beautiful detail the highly distinct localization of each protein. Most of the tagged strains described in the manuscript are already available from the CGC, and when the remaining ones become available, they represent a powerful advance for the ECM community.

Main comments:

*The authors should correct the order of the numbered references to follow the order of the manuscript sections. For example, the very first reference in the introduction is currently numbered (5), while reference (1) first appears in the results section. I suggest the authors double-check all the references in case other references are out of order or misplaced. For example, the sentence "Estimates of the total number of protein species of the ECM, i.e. the matrisome,(11, 12) range from the hundreds to over 1000, many of which are subject to complex processing and post-translational modification." cites references 11 and 12, but reference 10 (Teuscher et al. 2019) is also highly relevant here, but doesn't appear for the first time until 3 paragraphs later.

*The authors describe a novel modular tagging system, introducing specific sgRNA sites on either side of the knock-in sequence so it can be efficiently swapped out for other tags by subsequent CRISPR injections. Two recent preprints have developed different modular tagging techniques (Han et al 2026; doi: 10.64898/2026.01.16.700006, Hefel et al 2026; doi: 10.64898/2026.01.15.699741) - the authors should discuss the pros and cons of their modular method in comparison with these.

*The manuscript would benefit from additional schematic diagrams that show the anatomy of the cuticle layers. Figures 2F and 5F are helpful, but especially for understanding the distinction between WT and bli(0) cortical/basal morphologies, a 3D diagram showing the relative positioning of the cortical and basal layers in WT and bli(0) worms would be helpful to understand Figure 5B,C. Otherwise, the basal layers in bli(0) worms shown in Figure 5B could be mistaken for alae.

*"In contrast, COL-80::mNG strut localization was abolished in bli-1(0) mutants and the remaining COL-80:mNG variably localized to annuli or furrows." - This should be shown in a panel of Figure 5 or a supplementary figure.

*102 genes were successfully tagged for this project - can the authors include any targeted genes that were unsuccessful in generating viable knock-in strains, if there were any? It would be useful to guide future tagging efforts.

*The authors may wish to consider publishing some form of their imaging dataset alongside this manuscript. Table 1 is valuable, but even better would be a web resource that hosts many of the high-quality images the authors obtained to prepare it.

Minor comments:

*In the sentence "Such resources have also established the importance of studying ECM components using endogenous knock-in tags, consistent fluorescent protein (XFP) tags and a modular editing pipeline for stable and reproducible analysis of protein localization." XFP is defined but never used again in the manuscript - consider removing it.

*In Figure S1A, the y-axis label is partially obscured.

*In Figure S1B, is 2:3:1 supposed to read 2:1?

*The authors should elaborate on the sentence "In contrast a pan-muscle mlc-1p::RFP co-injection marker enriched for edits (Figure S1F,G)" as it's not immediately clear to me how the data in S1F,G support it.

*"We successfully swapped mNG::FLAG to 2xOLLAS in 10 strains." should read "mSc::2xOLLAS"

*In Figure 1, presumably the gray/blue/black boxes are the linker, FLAG, and OLLAS sequences, but this should be made clearer either in the figure itself or in the caption.

*"we first examined a set of 27 cuticle genes initially defined by mutant phenotypes (17)." Table 1 should be referenced here, but it has 26 genes in bold, not 27. Reference 17 is cited for these 27 cuticle genes, but the genes bolded in Table 1 include some genes not mentioned in reference 17. Additional references should be cited to support the mutant phenotypes of col-121, lon-8, and ram-1.

*In Figure 4B, the quantification labels V, VL, LD, DL, D are too close together and difficult to read. The abbreviations should also be explained in the caption.

*The first panel in Figure 4D should have the text "DPY-5::mNG"

*The caption for Figure 5A mentions col-91 and col-125, but not the other panels - col-130, col-149, and rol-8. Is this deliberate?

*"Notably, we got efficient editing with 35 nt homology arms with our ssDNA templates, whereas a previous report used 59-134 nt homology arms." - Cite this previous report.

*"We then ran on an annealing program on a thermocycler" should read "We then ran an annealing program on a thermocycler"

*In Table 1, the "puncta" note for acn-1 is in a cell that appears merged with bli-5 below it.

*The first sentence of the Molecular biology methods section is confusing. The authors describe 36bp "homology arms", but then say these encode flexible linkers that are both 13aa long. 36bp encodes 12aa not 13aa. Is the 36bp referring to the homology arms for Gibson assembly, and a different 39bp sequence encodes the linkers?

*In Supplemental Table 1, the strain PHX3691 is described as sqt-1::mNG(syb3691) II. However, in the CGC, the strain PHX3691 is listed as being sqt-3(syb3691[sqt-3::mNG(int)]) V. Which is correct - sqt-1 or sqt-3?

Reviewer #2: Via the tagging of a large number of apical ECM proteins, Ragle et al. report here in the very interesting paper the generation of a very useful and rich resource that will be very important to the ECM field. The strains and patterns reported here represent an invaluable starting point for future studies. In more general terms, the manuscript very nicely illustrates how protein tagging has the potential to reveal new biology and to pave the path for future discovery science.

While I'm generally very enthusiastic about this manuscript, I do have one major request: To fully exploit and unleash the potential of this manuscript, the authors need to show more images of their protein localization patterns. As it stands the authors are quite focused on proteins that localize to the skin (perhaps not surprisingly, because that's what they are generally interested in), but it would be very useful - and, in fact, perhaps foundational for the research efforts. of others - if the authors were to better report and document the non-skin patterns. I'm specifically referring to the "interstitial" and "other" category in Table 1. The authors presently only give the reader a very limited taste of such patterns in Figure 6, but they really should be showing more. I'm not requesting what, in fact, other papers with large surveys of expression patterns have done - to show every single one of their expression patterns in the Supp, but the author should really consider showing one representative for each pattern.

(1) I really would want to see the "other" patterns in the pharynx, gut, uterus and coloemocytes (9 reporters). Please show all patterns, except those where two proteins show the exact same.

(2) The interstitial patterns: those are generally very poorly described ("etc." as in the Table legend doesn't cut it). I totally understand that the authors may not be able to make "sense" of some of the patterns - but that's even more of a reason to show these patterns. As I said, many of these patterns have the potential to serve as very valuable tools for others in the community and may reveal novel biology. For example, folks studying the excretory system, would love to see the patterns for the excretory pore/duct. As it stands, any read of the manuscript would need to order >20 strains to see for themselves which of the interstitial pattern is in the duct/pore, which a strong, specific, etc. It's also not presently clear whether any of the patterns is specific just for one specific locale (i.e. are their patterns that are exclusive to, e.g., the deirids; or the pores).

Again, the authors to show all the patterns, except if two proteins show the exact same localization pattern.

For all of the images above - and also for the images in Fig.6 as they currently are - it would be good to see a low mag overview image of an entire worm so that the reader can see the specificity of the expression pattern. In my understanding PLoS Biology is very flexible with Figure number, so the authors should not shy away from going double digit with Figure numbers.

A few additional comments:

- Is Table 1 incomplete? I haven't checked systematically, but I don't see col-34 listed, for example.

- please consider perhaps ordering the data in Table 1 in a different way. Right now it's alphabetical - it may be more useful to sort by pattern?

- Since much of the manuscript is about collagens, more "visual" background about their structural features may be useful. The authors describe some background on collagens in the Methods, but I would recommend to morph this into something more visual, into a Supp Fig.

- One very obvious question is which cells generate these proteins. For many patterns it's quite obvious (hypodermal cells), for other it's not quite as obvious (i.e. the interstitial patterns). Have the authors looked at scRNA databases to make some educated guesses/speculation about the source of the protein?

Reviewer #3 (David Fay, signs review): The manuscript by Ragle et al., describes a major undertaking to endogenously tag and categorize the expression and localization of scores of apical extracellular matrix proteins in C. elegans, most of which correspond to the larval and adult worm cuticle. In addition to the immediate findings, this work represents a valuable resource to the community for future studies, specifically for those trying to understand the orchestration that underlies the deposition and remodeling of complex extracellular matrices. The tagging methods used will allow for the further modification of the generated strains, such as color swapping, and the studies provide some useful methodological data and strategies for the use of CRISPR in C. elegans. The strains and dataset also provide a useful comparator to several recent transcriptional studies implicating several thousand genes in the molting cycle, including many secreted components or regulators of the aECM. Collectively, this work represents a substantive advancement in itself and will facilitate the advancement of the ECM field. I am therefore enthusiastic about its publication.

My comments relate almost entirely to presentation and are organized by Figure and section. Although well written, I was confused in several sections, and I think that there could be improvements in the overall organization that will better enable readers to find relevant information. This is admittedly not the easiest dataset to organize, and I think the authors tried their best and had some good ideas, but the large number and similarity of gene names underscores the need to optimizing this aspect of the paper. My general suggestions are to increase the number of figures (PLoS Biology doesn't have a specific limit), to further subdivide the proteins and include sub-tables of relevant proteins in most figures, and to ensure that all descriptive statements in the text are appropriately supported by either figure panels or easily accessible sub-tables within each section.

1) Fig 1 and associated.

a) I wasn't very clear on some aspects of the CRISPR strategy descriptions and believe this section would benefit from some additional clarifications (Fig 1 and S1). There is some very good information and tools in this section so it's important that readers can grasp this easily and is consistent with the overall intent of the paper as a community resource.

b) For the legend for Fig 1 it would be helpful and consistent to move the (B) and (C) designations to the beginning of the relevant statements.

c) The transition from testing edit efficiency by targeting on GLH-1 versus the collagens (page 7 top) was abrupt or hard to follow.

d) There may also be some typos in Fig S1 or in the legend or it may just need more information; I was most confused when trying to parse results in S1E, F, and G. State what the dots represent etc.

e) Some experiments (color swaps) were carried out using short homology arm PCR templates whereas others used plasmids with longer arms. I think this needs to be spelled out more clearly.

2) Fig 2 and associated.

a) At the bottom of page 9 some comment about how the markers may have some effect on protein function as suggested by the double-marked strain would be appropriate here, although I realize that it is mentioned later.

b) The panels in Fig 2 weren't always precisely aligned and the lower images assigned to lower 2B could be made clearer.

c) In the legend, should DPY-13 be included in reference to panel C?

d) I was confused about "but it is unclear whether they also localize to the cortical layer" since the diagram and other data indicates furrow collagens localizing to the cortical layer.

e) I was unclear about the reason for the statement here that: "furrow' collagen localization does not precisely correlate with morphological furrows and instead may define distinct subregions within furrows.

f) "Two other collagen knock-ins, DPY-5::mNG and DPY-13::mNG, were enriched in annuli…" Is it more accurate to say that "Two other collagen knock-ins… were enriched in the fibrous layers underlying the annuli…? Is it still part of the annuli if it is below the strut? Fig 2F seems to indicate annuli and furrows being present in the cortical layer specifically.

g) Fig S4 indicate gut granules in DPY-5 L1?

h) Throughout this section, such as on page 11, a number of localization/expression patterns are mentioned without a reference to a figure or table. Generally, if something is mentioned in this context one expects a corresponding figure or table reference, usually with an image, to accompany the statement. Conversely, from a statement on page 11, I was expecting DPY-17 to be shown in Fig 2E.

i) Fig S5A. I would suggest not doubling the intensities of the hets prior to plotting as I think most readers won't expect this and may misinterpret the data (as I did) prior to reading the legend.

3) Fig 3 and associated.

a) As for comment 2h, I found there to be some mildly confusing elements to this figure and text. From the text I thought that COL-149 would be shown in either Fig 3 or S6, but that was not the case. In the Fig 3 legend, it refers to Fig S3, but this reference was seemingly incorrect. The authors will want to go through all the figures and text to ensure consistency and correctness, but also to consider organizing the figures somewhat differently to make accessing the information as straightforward as possible.

b) In the same vein, I kept going back to Table 1, which is a good summary, but the table spanned two pages, so I wasn't able to see the top row key on the second page. It would be helpful to have more smaller tables throughout (e.g., 5E) that group genes according to localization. This type of study could warrant 8-10 regular figures, versus the current six, which might help to distribute the data into bite-sized chunks and would allow the inclusion of more images that are referred to in the text.

c) Here (Fig 3F) and elsewhere, consider avoiding red/green images and text.

4) Fig 4 and associated.

a) On page 16 COL-71 is referenced to Fig S8, but this figure shows COL-20 instead.

b) In 4A indicate inside panels which is inner and outer. Anything to reduce reliance on the legend, which didn't explicitly state which was which either, is helpful.

c) Define V, D, VL, DL, LD, etc. in 4B. Also decompress the text.

Fig 5 and associated.

a) I like the idea of the table in 5E but was puzzled that quite a few of the proteins (COL-130, ROL-8, etc.) in the Fig 5 panels were not listed in 5E. For organization, it would be helpful to group these accordingly.

b) From the text statement on page 18 I was expecting some accompanying figure images regarding COL-80 expression in bli-1(0) mutants. As mentioned above, there were a number of instances where it seemed there could or should have been images following statements, but this was not always the case.

c) Correlating localization with reported transcriptional timing is an interesting component of the work. Consider showing this in graphical form using the x axis for timing (earliest expression) and color coding the proteins by dots based on where they are expressed. This would highlight interesting anomalies as well.

Fig 6, associated, and Discussion.

a) On page 20 "RAM-1::mNG" seems to be missing from the sentence that beings with the word "localized".

b) On page 24, I wasn't familiar with the meaning of "cluster D" classification to understand the point. Also, when looking up this reference elsewhere I noticed that it was missing on page 3.

c) Any thoughts regarding the relative activities of fluorescent proteins in the cuticle? For example, there used to be some idea that superfolder GFP worked better than regular GFP, but it seems like the mNG works well too.

As for all my reviews this is signed by David Fay.

Reviewer #4: C. elegans has a collagen-based cuticle that is organized into multiple layers and three-dimensional substructures. By tagging many components of this apical matrix, Ragle et al provide a view of its amazing structural complexity and an explanation of why there are so many different cuticle collagens (180!) - each is expressed in different stages, tissues, and spatial patterns. Major cuticle substructures such as furrows, alae, and struts are shown to contain numerous different collagens that mark distinct nano-structures within (or adjacent) to them. Various orifices where tubes open to the outside environment also contain unique collagens. Components of the deep fibrous layers and of more cortical cuticle layers are also identified. This is a landmark study that contains many fascinating observations and provides tools that will be transformative for the C. elegans research community. The data also hint at levels of complexity that could be present in other apical ECMs and in various other collagen-based matrices.

Major comments

1. I appreciate that the authors are trying to stick to a high level summary style and not get bogged down in details, but there are many statements in the text that need to be more clearly backed up with data in the figures/legends/methods. e.g.

-Table 1 summary has some omissions/errors - information there based on imaging of how many animals per stage/tissue?

-Claimed differences in localization of different furrow Dpy collagens need firmer evidence

-Use of blistered mutants to infer layer organization is hard to understand from the provided figure and has some caveats that should be acknowledged

-Various statements about specific collagens are made without citing a figure panel or table where the data are found (see detailed comments below)

2. The data suggest some possible inconsistencies with prevailing models, but do not clearly resolve these issues. While it's not necessary (and probably not feasible) to resolve all of these issues here, it is important that the text and summary cartoons not imply evidence that doesn't exist. Instead, highlight possible inconsistencies and questions that will require follow-up. e.g.

i. Do the 5 furrow collagens extend through both basal and cortical layers? Or are some exclusively basal and others cortical? (Text and figures imply different results, which is confusing, but the data seem inconclusive. Two color imaging might address this. Avoid drawing cartoons in a way that is inconsistent with your data)

ii. Do Roller mutations disrupt fibril orientations in the basal fibrous layers, as classically proposed? (not in any obvious way but needs further analysis, text handles this issue well)

iii. Are cortical collagens the first or last to be secreted and added to the cuticle? Or a mix of these? This relates to a central dogma about layered aECM - the idea that layers are built sequentially from the outermost to the innermost. I personally do not believe this dogma is correct. (Text leans towards dogma, expression data lean the other way, actual experiments not done but should be quite easy with the available fusions)

3. The authors could consider reorganizing some figures in a way that would allow summary cartoons to be more complete and accurate. e.g. it would be nice to have a complete summary cartoon indicating which collagens are in which basal fibrous layer (instead of just an incomplete cartoon in Fig. 2F before more data are shown in Fig. 4), another for furrows and furrow flanking patterns, another for struts. The existing summary cartoons in Figs. 5 and 6 are very effective but perhaps not complete.

4. To be broadly accessible to non-experts and those outside of the C. elegans community, it would be helpful to provide a little more up-front background/explanation and to compare the worm collagens to mammalian collagens

-an early cartoon of cuticle layers and substructures (to be cited in Introduction) would help put subsequent text and figures in context

-an early cartoon of a generic cuticle collagen would help introduce relevant domains/cleavage sites and justify that C-terminal tagging is a reasonable approach (whereas it wouldn't be in the most highly studied mammalian type I collagen)

-the worm must build at least most of its new cuticle before shedding the old one at the molt, so most cuticle assembly occurs during the prior stage (this is mentioned in the Discussion, but needed earlier in order to understand the whole discussion about cortical collagens).

-the worm has 180 cuticle collagens, how many collagens do humans have? there are parallels in the tissue specificity and the fibril type distinctions

Specific comments, in order of Figures and text appearance

5. Table 1 RELATED TO POINT 1. Be clearer about the strength of the evidence supporting this table. Because I see some errors/omissions, I'm not confident that white boxes are truly evidence of absence (rather than just absence of data).

sqt-3::mNG is expressed in the embryo (Birnbaum et al 2023) and found in the cuticle of all stages - why does Table show it as adult-specific?

dpy-6 is expressed in various interfacial tubes, including buccal cavity (Mazzoli et al biorxiv).

Some distinction between cuticle and pre-cuticle would be helpful here

6. Fig. 1A would benefit from clearer labels and/or legend - blue box = FLAG, grey boxes = linkers, black box = 3' UTR?

Mention the rationale for using mNeonGreen? I didn't see any citation for this fluorophore.

7. Fig. 1B,C,Dcould be moved to Fig. S1 with the rest of related methods information

8. Fig. 2A (furrow collagens). RELATED TO POINT 1. I'm not seeing a clear difference between DPY-2 and DPY-3 here, despite claims in the text - both have some shorter lines and some longer ones. DPY-9 does look potentially different in extending less close to the seam, but this could be a focal plan difference. Any claimed difference here needs some more detail and numbers associated with it.

9. Fig. 2E (DPY-14) It's worth pointing out that, like SQT-3 and DPY-17, this basal layer collagen is being secreted very early into the space between the embryo and the eggshell. Mazzoli et al (biorxiv) see a similar result with the furrow collagens, so this appears to be a widespread trend - L1 cuticle collagens are dumped into a slurry long before specific patterns emerge.

10. Fig. 2F RELATED TO POINTS 1 AND 2. It is confusing that the legend (and Fig. 5E) says there is evidence that furrow collagens are basal (citation needed here), yet the cartoon here very explicitly indicates them as cortical. The striped pattern observed with the basal fibrous layer collagens is consistent with furrows or struts extending down into those layers (but that is not how it is drawn in the cartoon). It would be useful to do some 2-color imaging of furrow collagens with cortical/medial/basal collagens to resolve the relationships and redraw the cartoon accordingly. Or if you aren't going to address it, then maybe don't put the furrow collagen images in a figure that is emphasizing conclusions about layers!

11. Fig. 2F RELATED TO POINTS 1,2,3. It is confusing that the text and legend say that SQT-2 is non-fibrous, but the cartoon indicates it being in a fibrous layer - why? Explain how you determined that a collagen is in the "outer fibrous" or "inner fibrous" layer. Features of these layers are not explained until p. 16 and Fig. 4. I kept having to go back and forth between these sections.

12. Fig. 3B and 3C are showing an intriguing alae defect in dpy-3 mutants - worth mentioning, especially given that this furrow collagen is not itself observed in the alae.

13. Fig. 3 legend citation to Figure S3 is an error, I think you mean S6

14. Fig. 4B Define the abbreviations at graph bottom. Legend statement about chirality should cite Fig. S8 and ref. 57.

15. Fig. 5B-D RELATED TO POINT 1. It's very hard to understand how the collagen patterns relate to blisters since we can't see the blisters in any of these images. e.g. how can you tell that COL-91 is cortical from this image?? Please provide bright field images if available.

16. Fig. 5 As the authors point out, struts may do more than simply hold the cortical and basal layers together - they may actually influence layer organization or integrity. In that case, one could be misled about normal layer localization by inferring it in these mutant backgrounds. Another independent confirmation of the layer assignments would be to do two color imaging of putative cortical vs. basal components. In the absence of that, at least acknowledge caveats.

17. Fig. 5E RELATED TO POINT 2. Please indicate the Meeuse molt time on this table (or in legend) - it will run right through the middle of the cortical collagen phases. Leaving this out is misleading as people will assume that molt is at the bottom of the table. The data don't fit the prevailing model of cortical collagens being the earliest secreted, but that is one of things that is exciting here and needs follow-up.

Several of the listed cortical collagens appear to be expressed only in adult according to Meeuse et al 2020, which would mean they must be added on top of the existing cuticle. This should be easy to confirm or refute by imaging your tagged collagen strains at the molt, and would be a very important result!

18. Fig. 5F RELATED TO POINT 3. Why are several collagens listed as cortical in 5E but then not shown here in 5F? (col-12, col-63, col-80, col-81, col-98) - what is the basis for inclusion/exclusion?

19. Fig. S9 This should be a main figure!

20. Fig. S10 This is a cool result but it is very hard to understand what we are looking at here. The fluorescent images need accompanying bright field images or at least some cartoons to help orient us.

21. Figure scale bars are sometimes missing, sometimes labelled with sizes, sometimes not, sometimes explained in legend, sometimes not. (panels without scale information include 2D, 3F, 5B)

22. p. 4 In addition to ref 26 (which is about glial sockets), other interfacial tube matrix citations include ref. 61 or Cohen et al 2020 (vulva), Kelley et al 2015 (buccal cavity), and Gill et al 2016 (excretory duct/pore).

23. p. 5 Here we report a protein-tagging resource of knock-in strains for 102 aECM components…

For select genes, we generated an additional mScarlet(mSc) red FP-tagged versions to facilitate co-localization studies - citeTable S1

24. p. 8 We next prioritized collagens based on expression as described above. The above text is a dense description of methods that doesn't say anything about collagen expression as far as I could see. The text vaguely implies, but should say more explicitly, that you screened for edits based on visible fluorescence.

25. p. 9 To test whether aECM components tolerate mNG tagging we first examined a set of 27cuticle genes initially defined by mutant phenotypes (17). Knock-ins for four of these had been previously reported (e.g. bli-1, bli-2, bli-6, dpy-13) (7) - actually should be 6 since knock-ins of SQT-3 and DPY-17 were reported in Birnbaum et al 2023, though not analyzed for adult patterns there.

26. p. 9 Knock-in strains for such genetically defined cuticle genes were morphologically normal and displayed bright fluorescence in the cuticle. This is not entirely true according to later paragraph p. 12 (at least 4 strains had mutant phenotypes, more if you count genetic interactions).

27. RELATED TO POINT 1. p. 9 Within this set, individual patterns differed in detail. DPY-2::mNG showed the most consistent labeling, extending around the circumference to the lateral midline or lateral alae, without breaks (Figure 2A). DPY-3::mNG furrows often did not extend to the lateral midline. Clarify what is the evidence behind these statements, because this distinction is not apparent from the images shown. In both strains I see some furrows that don't extend to the mid-line.

28. RELATED TO POINT 1. p. 9, Interestingly, BIS-1/COL-121, identified on the basis of permeability barrier defects (56),also localized to furrows specifically in embryonic and L1 stages - Table or Figure citation for these data?

29. RELATED TO POINT 4. p. 11-12 mentions dibasic cleavage sites completely lacking any context; the fact that worm collagens are cleaved at a dibasic site isn't introduced until the next paragraph.

30. RELATED TO POINT 4. p. 12 An important question at the outset was whether collagens readily tolerate tagging. Yes, so why not discuss it at the outset? Many readers will find it surprising that you are tagging collagens at their C-terminus, since C-terminal cleavage is such an important part of human Type I collagen regulation.

31. p. 12 C.elegans cuticle collagens undergo N-terminal cleavage by subtilisin proteases... Citations needed for this statement. I don't see a citation for Thacker et al 1995 anywhere in the manuscript.

32. RELATED TO POINT 1. p. 12. Several other collagens we tagged contain similar C-terminal cleavage sites, however C-terminal knock-ins displayed cuticle localization. Which other collagens specifically are you referring to? Citation of a methods section, figure or table for these data?

33. p. 12 Of the [27] cuticle collagens defined by mutant phenotype we detected phenotypes for only three [FOUR or FIVE?] knock-in strains. The DPY-4::mNG and DPY-8::mNG KI strains displayed mild Dpy phenotypes whereas RAM-4/COL-43::mNG and RAM-2/COL-82::mNG KI males displayed Ram phenotypes, suggesting these insertions affect gene function....

34. RELATED TO POINT 1. p. 18. In contrast, COL-80::mNG strut localization was abolished in bli-1(0) mutants and the remaining COL-80:mNG variably localized to annuli or furrows. Figure or Table citation for these data?

35. p. 18 Thus, in some cases, disruption of struts may affect cuticle organization beyond separation of cortical and basal layers. If struts influence layer organization/integrity, then one could be misled about normal layer localization by inferring it in these mutant backgrounds.

36. RELATED TO POINTS 2, 4. pp 19-20: This timing would suggest the cortical collagens are expressed either just before the molt, presumably for construction of the cuticle early in the next larval stage, or right after the molt. As it is unclear how cortical collagens would be assembled outside basal layers, we favor the interpretation that the cortical collagen genes are expressed very early in the cyclic synthesis of each aECM. Then how do you explain col-63 and col-81, which don't fit this model? Meeuse puts ecdysis around 135˚, which would suggest some cortical collagens are expressed very early but others are expressed very late. This section feels like you are trying to fit the data to the dogma instead of acknowledging inconsistencies.

37. p. 20: [ ? collagen name missing?] localized exclusively to male tail rays,

38. p. 20: in addition to not seeing the proposed intestinal expression of cuticle collagens, do you care to comment on recent claims for distal tip cell expression of such collagens? https://pubmed.ncbi.nlm.nih.gov/41720312/

39. RELATED TO POINT 2. p. 24 Nevertheless, our analysis suggests that cortically localized proteins tend to have early transcript peaks whereas basal proteins have later peaks. I disagree strongly with this assessment. Several of the listed cortical collagens appear to be expressed only in adult according to Meeuse et al 2020, which would mean they must be added on top of the existing cuticle. This should be easy to confirm or refute by imaging your tagged collagen strains.

40. p. 24 Tagging of Hedgehog-related proteins has also revealed specific localization to substructures of the cuticle or precuticle (79). Ref 26 also relevant here, along with Chiyoda et al https://pubmed.ncbi.nlm.nih.gov/33872306/

41. p. 30 citation to Figure 1, specify panel(s) and or move to Fig. S1. Description of SAP Trap methods should cite Fig. S3.

42. p. 32 Description of unc-119 selection method should cite Fig. S2.

43. p. 33 citations to Figure 2 appear to be in error - data are in Fig. 1 and Fig. S1

44. RELATED TO POINT 1. p. 33 Approximately 35 collagens have potential C-terminal cleavage sites resembling that of SQT-3 (DGG motif) including BLI-6, DPY-4, DPY-13, ROL-1, and LON-3. Provide a citation or data (e.g. a sequence alignment) to back up this statement.

# Author comment: Author response to Decision Letter 2

**DOI:** 10.1371/journal.pbio.3004059.r003

4 Sep 2026

Attachment Submitted filename: Response to reviewers.docx

# Editor report: Decision Letter 2

**DOI:** 10.1371/journal.pbio.3004059.r004

**Authors:** Richard Hodge

21 Sep 2026

Dear Dr Ward,

Thank you for your patience while we considered your revised manuscript "Multiscale patterning of a model apical extracellular matrix revealed by systematic endogenous protein tagging" for publication as a Methods and Resources Article at PLOS Biology. This revised version of your manuscript has been evaluated by the PLOS Biology editors and the Academic Editor.

Based on our Academic Editor's assessment of your revision, we are likely to accept this manuscript for publication, provided you address the following data and other policy-related requests that I have provided below (A-E). Please also ensure that you double check the figure legends, experimental methods and the supplemental material to ensure everything is correct at this stage.

(A) We routinely suggest changes to titles to ensure maximum accessibility for a broad, non-specialist readership. In this case, we suggest a minor edit to the title, as follows, to further bring out the Resource aspect of the study. Please ensure you change both the manuscript file and the online submission system, as they need to match for final acceptance:

“A systematic fluorescently tagged protein toolkit for dissecting apical extracellular matrix patterning in C. elegans”

(B) You may be aware of the PLOS Data Policy, which requires that all data be made available without restriction: http://journals.plos.org/plosbiology/s/data-availability. For more information, please also see this editorial: http://dx.doi.org/10.1371/journal.pbio.1001797

Note that we do not require all raw data. Rather, we ask that all individual quantitative observations that underlie the data summarized in the figures and results of your paper be made available in one of the following forms:

-Supplementary files (e.g., excel). Please ensure that all data files are uploaded as 'Supporting Information' and are invariably referred to (in the manuscript, figure legends, and the Description field when uploading your files) using the following format verbatim: S1 Data, S2 Data, etc. Multiple panels of a single or even several figures can be included as multiple sheets in one excel file that is saved using exactly the following convention: S1_Data.xlsx (using an underscore).

-Deposition in a publicly available repository. Please also provide the accession code or a reviewer link so that we may view your data before publication.

Regardless of the method selected, please ensure that you provide the individual numerical values that underlie the summary data displayed in the following figure panels as they are essential for readers to assess your analysis and to reproduce it:

Figure 6B, S1A-H, S6A-B

NOTE: the numerical data provided should include all replicates AND the way in which the plotted mean and errors were derived (it should not present only the mean/average values).

(C) Please also ensure that each of the relevant figure legends in your manuscript include information on *WHERE THE UNDERLYING DATA CAN BE FOUND*, and ensure your supplemental data file/s has a legend.

(D) Per journal policy, if you have generated any custom code during the course of this investigation, please make it available without restrictions. Please ensure that the code is sufficiently well documented and reusable, and that your Data Statement in the Editorial Manager submission system accurately describes where your code can be found. More information on our Code Policy, what and how to share can be found here: https://journals.plos.org/plosbiology/s/code-availability

Please note that we cannot accept sole deposition of code in GitHub, as this could be changed after publication. However, you can archive this version of your publicly available GitHub code to Zenodo. Once you do this, it will generate a DOI number, which you will need to provide in the Data Accessibility Statement (you are welcome to also provide the GitHub access information). See the process for doing this here: https://docs.github.com/en/repositories/archiving-a-github-repository/referencing-and-citing-content

(E) Please ensure that you are using best practice for statistical reporting and data presentation. These are our guidelines https://journals.plos.org/plosbiology/s/best-practices-in-research-reporting#loc-statistical-reporting and a useful resource on data presentation https://journals.plos.org/plosbiology/article?id=10.1371/journal.pbio.1002128

- If you are reporting experiments where n ≤ 5, please plot each individual data point.

------------------------------------------------------------------------

As you address these items, please take this last chance to review your reference list to ensure that it is complete and correct. If you have cited papers that have been retracted, please include the rationale for doing so in the manuscript text, or remove these references and replace them with relevant current references. Any changes to the reference list should be mentioned in the cover letter that accompanies your revised manuscript.

In addition to these revisions, you may need to complete some formatting changes, which you will receive in a follow up email. A member of our team will be in touch with a set of requests shortly. If you do not receive a separate email within a few days, please assume that checks have been completed, and no additional changes are required.

We expect to receive your revised manuscript within two weeks.

To submit your revision, please go to https://www.editorialmanager.com/pbiology/ and log in as an Author. Click the link labelled 'Submissions Needing Revision' to find your submission record. Your revised submission must include the following:

- a cover letter that should detail your responses to any editorial requests, if applicable, and whether changes have been made to the reference list

- a Response to Reviewers file that provides a detailed response to the reviewers' comments (if applicable, if not applicable please do not delete your existing 'Response to Reviewers' file.)

- a track-changes file indicating any changes that you have made to the manuscript.

NOTE: If Supporting Information files are included with your article, note that these are not copyedited and will be published as they are submitted. Please ensure that these files are legible and of high quality (at least 300 dpi) in an easily accessible file format. For this reason, please be aware that any references listed in an SI file will not be indexed. For more information, see our Supporting Information guidelines:

https://journals.plos.org/plosbiology/s/supporting-information

*Published Peer Review History*

Please note that you may have the opportunity to make the peer review history publicly available. The record will include editor decision letters (with reviews) and your responses to reviewer comments. If eligible, we will contact you to opt in or out. Please see here for more details:

https://plos.org/published-peer-review-history/

*Press*

Should you, your institution's press office or the journal office choose to press release your paper, please ensure you have opted out of Early Article Posting on the submission form. We ask that you notify us as soon as possible if you or your institution is planning to press release the article.

*Protocols deposition*

To enhance the reproducibility of your results, we recommend that if applicable you deposit your laboratory protocols in protocols.io, where a protocol can be assigned its own identifier (DOI) such that it can be cited independently in the future. Additionally, PLOS ONE offers an option for publishing peer-reviewed Lab Protocol articles, which describe protocols hosted on protocols.io. Read more information on sharing protocols at https://plos.org/protocols?utm_medium=editorial-email&utm_source=authorletters&utm_campaign=protocols

Please do not hesitate to contact me should you have any questions.

Best wishes,

Richard

Richard Hodge, PhD

Senior Editor, PLOS Biology

rhodge@plos.org

PLOS

Empowering researchers to transform science

Carlyle House, Carlyle Road, Cambridge, CB4 3DN, United Kingdom

California (U.S.) corporation #C2354500, based in San Francisco

# Author comment: Author response to Decision Letter 3

**DOI:** 10.1371/journal.pbio.3004059.r005

29 Sep 2026

Attachment Submitted filename: 260926 Editorial review response.docx

# Editor report: Decision Letter 3

**DOI:** 10.1371/journal.pbio.3004059.r006

**Authors:** Richard Hodge

30 Sep 2026

Dear Jordan,

On behalf of my colleagues and the Academic Editor, Cody Smith, I am pleased to say that we can accept your manuscript for publication, provided you address any remaining formatting and reporting issues. These will be detailed in an email you should receive within 2-3 business days from our colleagues in the journal operations team; no action is required from you until then. Please note that we will not be able to formally accept your manuscript and schedule it for publication until you have completed any requested changes.

Please take a minute to log into Editorial Manager at http://www.editorialmanager.com/pbiology/, click the "Update My Information" link at the top of the page, and update your user information to ensure an efficient production process.

PRESS

We frequently collaborate with press offices. If your institution or institutions have a press office, please notify them about your upcoming paper at this point, to enable them to help maximise its impact. If the press office is planning to promote your findings, we would be grateful if they could coordinate with biologypress@plos.org. If you have previously opted in to the early version process, we ask that you notify us immediately of any press plans so that we may opt out on your behalf.

We also ask that you take this opportunity to read our Embargo Policy regarding the discussion, promotion and media coverage of work that is yet to be published by PLOS. As your manuscript is not yet published, it is bound by the conditions of our Embargo Policy. Please be aware that this policy is in place both to ensure that any press coverage of your article is fully substantiated and to provide a direct link between such coverage and the published work. For full details of our Embargo Policy, please visit http://www.plos.org/about/media-inquiries/embargo-policy/.

Thank you again for choosing PLOS Biology for publication and supporting Open Access publishing. We look forward to publishing your study.

Best wishes,

Richard

Richard Hodge, PhD

Senior Editor, PLOS Biology

rhodge@plos.org

PLOS

Empowering researchers to transform science

Carlyle House, Carlyle Road, Cambridge, CB4 3DN, United Kingdom

California (U.S.) corporation #C2354500, based in San Francisco
